# Supplementary material for: How Deep Can the Endophytic Mycobiome Go? A Case Study on Six Woody Species from the Brazilian Cerrado
Source: J Fungi (Basel). 2023 Apr 25;9(5):508. doi: 10.3390/jof9050508 (PMC10219290; doi:10.3390/jof9050508)
Supplement: Supplementary file 1 [file jof-09-00508-s001.zip › Supplementary Figure S1.pdf]

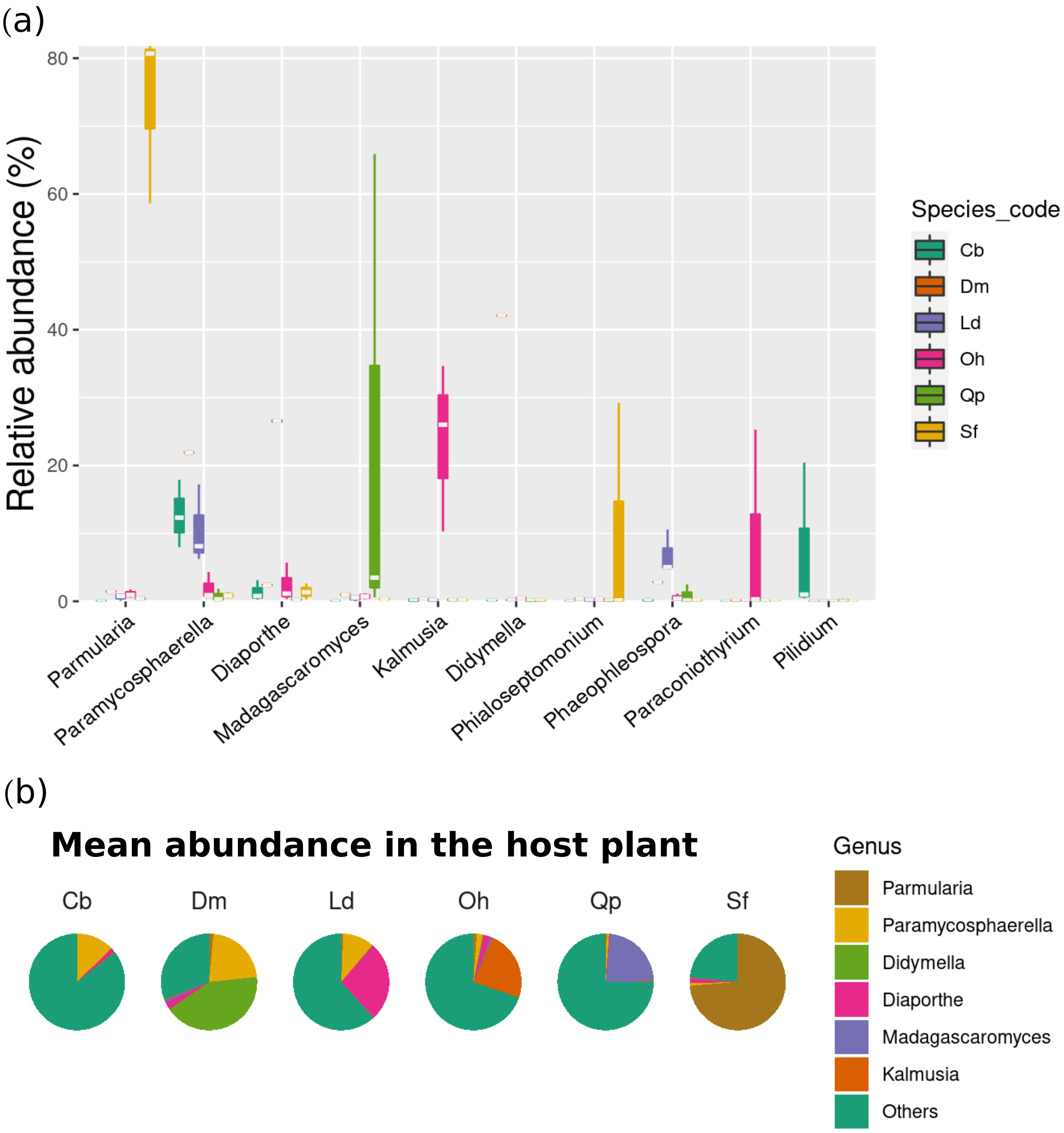

Figure S1. Taxonomic composition of the leaf endophytic fungal community from six host plant species at the genus level.
