# Supplementary material for: How Deep Can the Endophytic Mycobiome Go? A Case Study on Six Woody Species from the Brazilian Cerrado
Source: J Fungi (Basel). 2023 Apr 25;9(5):508. doi: 10.3390/jof9050508 (PMC10219290; doi:10.3390/jof9050508)
Supplement: Supplementary file 1 [file jof-09-00508-s001.zip › Supplementary Figure S2.pdf]

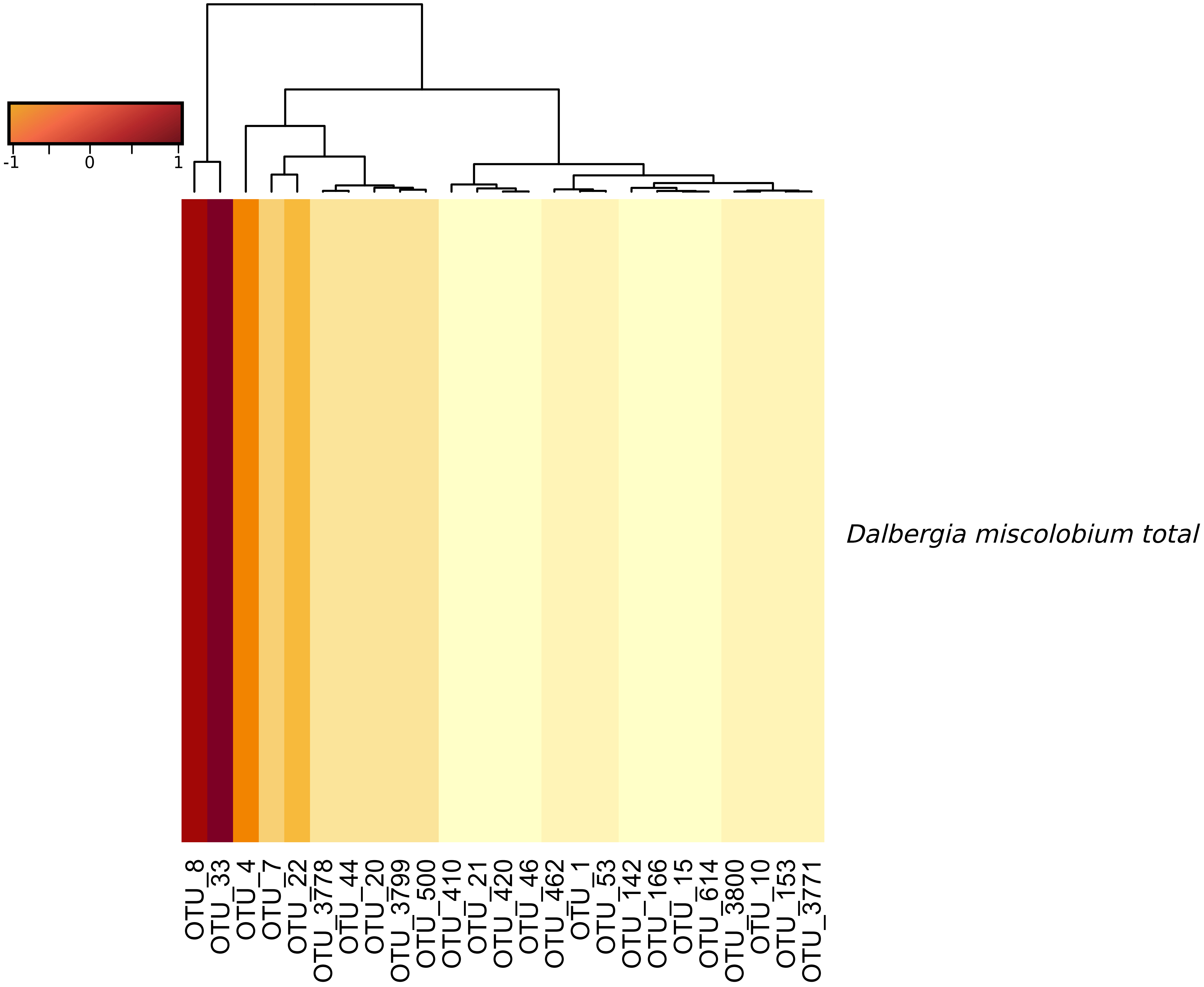


**Legend**

| OTU | Family | Genus |
|---|---|---|
| OTU_1 | Parmulariaceae | *Parmularia* |
| OTU_4 | Unclassified_ Ascomycota | Unclassified_ Ascomycota |
| OTU_7 | Unclassified_ Ascomycota | Unclassified_ Ascomycota |
| OTU_8 | Mycosphaerellaceae | *Paramycosphaerella* |
| OTU_10 | Diaporthaceae | *Diaporthe* |
| OTU_15 | Parmulariaceae | *Parmularia* |
| OTU_20 | Mycosphaerellaceae | *Paramycosphaerella* |
| OTU_21 | Mycosphaerellaceae | *Madagascaromyces* |
| OTU_22 | Mycosphaerellaceae | *Phaeophleospora* |
| OTU_33 | Didymellaceae | *Didymella* |
| OTU_44 | Teratosphaeriaceae | *Pseudoteratosphaeria* |
| OTU_46 | Unclassified_ Ascomycota | Unclassified_ Ascomycota |
| OTU_53 | Teratosphaeriaceae | *Pseudoteratosphaeria* |
| OTU_142 | Teratosphaeriaceae | *Neophaeothecoidea* |
| OTU_153 | *Teratosphaeriaceae* | *Neophaeothecoidea* |
| OTU_166 | Phyllostictaceae | *Phyllosticta* |
| OTU_410 | Unclassified_ Ascomycota | Unclassified_ Ascomycota |
| OTU_420 | Teratosphaeriaceae | *Neophaeothecoidea* |
| OTU_462 | Teichosporaceae | *Teichospora* |
| OTU_500 | Unclassified_ Ascomycota | Unclassified_ Ascomycota |
| OTU_614 | Unclassified_ Ascomycota | Unclassified_ Ascomycota |
| OTU_3771 | *Aureobasidiaceae* | *Aureobasidium* |
| OTU_3778 | Diaporthaceae | *Diaporthe* |
| OTU_3799 | Unclassified_ Ascomycota | Unclassified_ Ascomycota |
| OTU_3800 | Unclassified_ Ascomycota | Unclassified_ Ascomycota |

Figure S2. Heatmap depicting the relative abundance of the 25 most abundant OTUs in *Dalbergia miscolobium*.
