# Supplementary material for: How Deep Can the Endophytic Mycobiome Go? A Case Study on Six Woody Species from the Brazilian Cerrado
Source: J Fungi (Basel). 2023 Apr 25;9(5):508. doi: 10.3390/jof9050508 (PMC10219290; doi:10.3390/jof9050508)
Supplement: Supplementary file 1 [file jof-09-00508-s001.zip › Supplementary Table S1.pdf]

Table S1. Taxonomic assignment of OTUs.

Phylum	Class	Order	Family	Genus	Species	CB1629C	CB1765C	CB996C	DM18
Ascomycot	Dothideom	Asterinales	Parmularia	Parmularia		0	69	0	297
Ascomycot	Dothideom	Capnodiale	Mycosphae	Paramycosphaerella		8318	12268	20159	7260
Ascomycot	Dothideom	Pleosporal	Didymosph	Kalmusia		51	493	119	124
Ascomycot	Dothideom	Capnodiales				53	34	3124	80
Ascomycot	Sordariom	Diaporthal	Diaporthac	Diaporthe		82	733	38	109
Ascomycot	Sordariom	Hypocreale	Nectriacea	Phialoseptomonium		0	8	0	142
Ascomycot	Dothideom	Capnodiale	Mycosphae	Madagascaromyces		0	0	0	154
Ascomycot	Dothideom	Capnodiale	Mycosphae	Madagascaromyces		0	0	0	102
Ascomycot	Sordariom	Diaporthal	Lamproconiaceae			0	0	0	61
Ascomycot	Sordariom	Diaporthal	Diaporthac	Diaporthe		166	1024	136	245
Ascomycot	Dothideom	Capnodiale	Mycosphae	Phaeophleospora		113	247	109	891
Ascomycot	Dothideom	Pleosporal	Didymosph	Paraconiothyrium		0	0	0	36
Ascomycot	Leotiomyc	Helotiales	Chaetomel	Pilidium		906	466	24675	0
Ascomycot	Eurotiomy	Eurotiales	Aspergillaceae			0	46	19	1882
Ascomycot	Leotiomyc	Helotiales	Pezizellace	Porodiplod	livistonae	0	0	0	66
Ascomycot	Dothideom	Capnodiale	Mycosphae	Madagascaromyces		0	0	0	101
Ascomycot	Dothideom	Pleosporal	Didymellac	Didymella		128	48	0	15059
Ascomycot	Dothideom	Capnodiale	Mycosphae	Xenosonderhenia		0	17	0	76
Ascomycot	Sordariom	Xylariales	Sporocada	Neopestalotiopsis		852	503	1218	140
Ascomycot	Dothideom	Pleosporal	Pleosporac	Alternaria		222	1172	130	67
Ascomycot	Sordariom	Diaporthal	Diaporthac	Diaporthe		185	181	18	26
Ascomycot	Dothideom	Capnodiale	Teratospha	Pseudoteratosphaeria		1532	2274	887	385
Ascomycot	Dothideom	Capnodiale	Cladospori	Cladosporium		405	744	566	154
Ascomycot	Dothideom	Pleosporal	Didymellac	Epicoccum		0	0	0	0
Ascomycot	Dothideom	Capnodiale	Mycosphaerellaceae			0	0	0	70
Ascomycot	Dothideom	Capnodiale	Mycosphae	Pseudocercospora		0	0	0	0
Ascomycot	Eurotiomy	Chaetothyı	Chaetothyı	Strelitziana		0	83	0	24
Ascomycot	Dothideom	Capnodiale	Mycosphae	Pseudocercospora		22	0	39	38
Ascomycot	Dothideom	Capnodiale	Mycosphae	Paramycosphaerella		965	763	1075	440
Ascomycot	Dothideom	Capnodiale	Mycosphae	Phaeophleospora		0	0	0	113
Ascomycot	Sordariom	Diaporthal	Diaporthac	Diaporthe		0	0	0	0
Ascomycot	Dothideom	Capnodiale	Mycosphae	Stomiopeltis		140	727	3606	0
Ascomycot	Eurotiomy	Eurotiales	Aspergillac	Aspergillus		0	0	0	14
Ascomycot	Dothideom	Pleosporal	Didymellac	Epicoccum		0	0	0	0
Ascomycot	Dothideom	Pleosporales				0	0	0	38
Ascomycot	Sordariom	Diaporthal	Diaporthac	Diaporthe		16	397	0	0
Ascomycot	Eurotiomy	Onygenale	Onygenale	Zeloasperisporium		0	0	201	29
Ascomycot	Dothideom	Asterinales	Parmularia	Parmularia		0	0	0	219
Ascomycot	Sordariom	Diaporthal	Diaporthac	Diaporthe		0	344	0	0
Ascomycot	Dothideom	Capnodiale	Teratospha	Pseudoteratosphaeria		384	274	614	303
Ascomycot	Dothideom	Capnodiale	Teratospha	Neophaeothecoidea		446	281	2243	166
Ascomycot	Sordariom	Chaetosphaeriales				0	13	0	35
Ascomycot	Sordariom	Diaporthal	Diaporthac	Diaporthe		0	68	0	24
Ascomycot	Sordariom	Chaetosph	Chaetosph	Dendrophoma		0	16	0	78
Ascomycot	Sordariom	Trichospha	Trichospha	Nigrospora		0	0	58	0
Ascomycot	Sordariom	Xylariales	Sporocada	Pestalotiopsis		145	97	252	26
Ascomycot	Dothideom	Capnodiale	Mycosphae	Phaeophleospora		0	0	0	0
Ascomycot	Sordariom	Trichospha	Trichospha	Nigrospora		256	176	139	0

Ascomycot	Sordariom	Diaporthal	Diaporthaceae		0	128	0	22
Ascomycot	Dothideom	Capnodiale	Mycosphaerellaceae		0	0	0	18
Ascomycot	Dothideom	Capnodiale	Mycosphae	Zasmidium	110	211	123	85
Ascomycot	Dothideom	Capnodiale	Mycosphae	Zasmidium	152	173	130	85
Ascomycot	Sordariom	Diaporthal	Diaporthac	Diaporthe	58	91	53	0
Ascomycot	Dothideom	Capnodiale	Mycosphae	Pseudocer neriicola	0	13	0	0
Ascomycot	Sordariom	Diaporthal	Diaporthac	Diaporthe	14	42	0	0
Ascomycot	Dothideom	Capnodiale	Mycosphae	Paramycosphaerella	0	0	0	0
Ascomycot	Sordariom	Diaporthal	Diaporthac	Diaporthe	0	122	0	0
Ascomycot	Dothideom	Botryospha	Botryospha	Lasiodiplodia	0	0	0	16
Ascomycot	Sordariomycetes				0	10	0	0
Ascomycot	Dothideom	Capnodiale	Mycosphae	Geastrumia	121	248	1565	0
Ascomycot	Dothideom	Capnodiales			41	133	142	88
Ascomycot	Sordariom	Chaetosph	Chaetosph	Dictyochaeta	0	0	0	10
Ascomycot	Dothideom	Capnodiale	Teratospha	Neophaeothecoidea	685	56	827	249
Ascomycot	Sordariom	Diaporthal	Diaporthac	Diaporthe	0	0	0	0
Ascomycot	Sordariom	Diaporthal	Diaporthac	Diaporthe	95	0	0	0
Ascomycot	Sordariom	Diaporthal	Diaporthac	Diaporthe	0	0	0	54
Ascomycot	Dothideom	Capnodiale	Cladospori	Cladosporium	0	18	0	10
Ascomycot	Dothideom	Capnodiale	Mycosphae	Xenosonderhenia	0	0	0	0
Ascomycot	Dothideom	Pleosporales			28	118	41	0
Ascomycot	Dothideom	Capnodiale	Mycosphae	Geastrumia	90	965	448	0
Ascomycot	Sordariom	Diaporthal	Diaporthac	Diaporthe	90	85	0	0
Ascomycot	Dothideom	Capnodiale	Mycosphaerellaceae		106	82	288	41
Ascomycot	Sordariom	Diaporthal	Diaporthac	Diaporthe	0	0	0	0
Ascomycot	Dothideom	Pleosporales			0	23	27	0
Ascomycot	Dothideom	Capnodiale	Mycosphae	Neoceratosperma	0	0	0	44
Ascomycot	Sordariom	Diaporthal	Diaporthac	Diaporthe	29	0	0	0
Ascomycot	Dothideom	Dothideom	Dothideom	Radulidium	431	668	67	0
Ascomycot	Dothideom	Capnodiale	Mycosphaerellaceae		0	6	0	0
Ascomycot	Eurotiomy	Onygenale	Onygenale	Zeloasperisporium	0	0	0	6
Ascomycot	Sordariom	Trichospha	Trichospha	Nigrospora	193	0	57	41
Ascomycot	Sordariom	Chaetosph	Chaetosphaeriaceae		0	326	121	6
Ascomycot	Dothideom	Capnodiale	Teratospha	Neophaeothecoidea	136	85	1031	0
Ascomycot	Dothideom	Capnodiale	Mycosphae	Septoria	104	169	197	35
Ascomycot	Dothideom	Capnodiale	Cladospori	Cladosporium	0	0	0	0
Ascomycot	Sordariom	Hypocreale	Nectriacea	Gibberella	153	119	59	31
Ascomycot	Sordariom	Diaporthal	Diaporthac	Diaporthe	0	0	0	0
Ascomycot	Sordariom	Hypocreale	Nectriacea	Gibberella	592	266	350	0
Ascomycot	Dothideom	Pleosporal	Didymellac	Neodidymella	0	0	0	37
Ascomycot	Sordariom	Chaetosphaeriales			0	0	0	0
Ascomycot	Dothideomycetes				22	79	1056	0
Ascomycot	Dothideom	Capnodiale	Mycosphae	Stomiopeltis	24	0	1131	0
Ascomycot	Dothideom	Botryospha	Phyllosticta	Phyllosticta	0	0	0	223
Ascomycot	Dothideom	Pleosporal	Didymellac	Epicoccum	42	379	40	32
Ascomycot	Dothideom	Pleosporal	Didymellaceae		0	0	0	0
Ascomycot	Dothideom	Capnodiale	Teratospha	Pseudoteratosphaeria	354	80	197	97
Ascomycot	Dothideom	Capnodiale	Mycosphae	Madagascaromyces	0	0	0	0
Ascomycot	Sordariom	Diaporthal	Diaporthac	Diaporthe	23	30	16	0
Ascomycot	Dothideom	Capnodiale	Teratosphaeriaceae		285	67	571	10

Ascomycot	Sordariom	Sordariales	Chaetomia	Botryotrichum		489	183	302	0
Ascomycot	Dothideom	Capnodiale	Mycosphae	Pseudocercospora		0	0	0	0
Ascomycot	Sordariom	Trichospha	Trichospha	Nigrospora		0	0	0	0
Ascomycot	Dothideom	Capnodiale	Mycosphae	Paramycosphaerella		0	0	0	0
Ascomycot	Dothideomycetes					30	154	719	0
Ascomycot	Dothideom	Capnodiale	Teratospha	Pseudoteratosphaeria		92	165	439	62
Ascomycot	Dothideom	Pleosporal	Teichospor	Teichospora		570	0	8	286
Ascomycot	Sordariom	Diaporthal	Diaporthac	Diaporthe	sackstonii	26	0	0	0
Ascomycot	Sordariom	Xylariales	Xylariales_	Liberomyces		9	795	11	0
Ascomycot	Sordariom	Diaporthal	Diaporthac	Diaporthe		0	0	0	0
Ascomycot	Sordariom	Glomerella	Plectospha	Paragibellulopsis		265	177	376	0
Ascomycot	Sordariom	Chaetosph	Chaetosphaeriaceae			0	0	0	0
Ascomycot	Dothideom	Capnodiale	Mycosphae	Stomiopeltis		0	0	803	0
Ascomycot	Dothideom	Botryospha	Phyllosticta	Phyllosticta		0	0	0	67
Ascomycot	Dothideom	Pleosporal	Didymospł	Paracamarosporium		0	0	0	0
Ascomycot	Dothideom	Pleosporal	Pleosporac	Alternaria		0	0	0	0
Ascomycot	Sordariom	Diaporthal	Diaporthac	Diaporthe		0	0	0	0
Ascomycot	Sordariom	Diaporthal	Diaporthac	Diaporthe		0	0	0	0
Ascomycot	Dothideom	Pleosporal	Didymellac	Epicoccum		0	0	13	0
Ascomycot	Dothideom	Dothideale	Aureobasic	Aureobasidium		17	0	14	0
Ascomycot	Dothideom	Dothideale	Aureobasic	Aureobasidium		0	0	0	0
Ascomycot	Sordariom	Diaporthal	Diaporthaceae			0	0	0	0
Ascomycot	Pezizomyc	Pezizales	Pyronemat	Pseudombrophila		355	172	180	0
Ascomycot	Sordariom	Diaporthal	Diaporthac	Diaporthe		0	0	0	0
Ascomycot	Dothideom	Botryospha	Phyllosticta	Phyllosticta		5	5	6	18
Ascomycot	Eurotiomy	Chaetothyriales				15	0	680	0
Ascomycot	Dothideomycetes					0	0	0	0
Ascomycot	Sordariom	Hypocreale	Nectriacea	Gibberella		0	0	0	14
Ascomycot	Dothideom	Pleosporal	Pleosporac	Bipolaris		0	0	0	0
Ascomycot	Sordariom	Xylariales	Sporocada	Pestalotiopsis		94	63	42	0
Ascomycot	Sordariom	Trichospha	Trichospha	Nigrospora		0	668	0	0
Ascomycot	Leotiomyc	Helotiales	Hyaloscypł	Proliferodiscus		81	284	16	0
Ascomycot	Eurotiomy	Onygenale	Onygenale	Zeloasperisporium		0	0	0	33
Ascomycot	Sordariom	Glomerella	Glomerella	Colletotrichum		0	76	0	10
Ascomycot	Dothideom	Capnodiales				0	0	0	0
Ascomycot	Sordariom	Glomerella	Plectospha	Plectosphaerella		149	45	78	0
Ascomycot	Dothideom	Capnodiales				0	43	340	0
Ascomycot	Dothideom	Pleosporal	Didymospł	Xenocamarosporium		0	0	29	0
Ascomycot	Sordariom	Diaporthal	Valsaceae	Cytospora		0	0	0	0
Ascomycot	Sordariom	Hypocreale	Nectriacea	Phialoseptomonium		0	0	0	0
Ascomycot	Dothideom	Capnodiale	Mycosphae	Zasmidium		0	0	0	0
Ascomycot	Dothideom	Capnodiale	Teratospha	Neophaeothecoidea		0	0	187	203
Ascomycot	Sordariom	Diaporthal	Diaporthac	Diaporthe		0	0	0	0
Ascomycot	Dothideom	Capnodiale	Teratospha	Pseudoteratosphaeria		0	0	0	0
Ascomycot	Sordariom	Diaporthal	Diaporthac	Diaporthe		29	0	0	0
Ascomycot	Dothideom	Pleosporal	Phaeospha	Neosulcatispora		22	0	17	13
Ascomycot	Sordariom	Diaporthal	Diaporthac	Diaporthe		0	0	0	8
Ascomycot	Sordariom	Glomerella	Glomerella	Colletotrichum		36	68	0	0
Ascomycot	Eurotiomy	Chaetothyriales				0	0	556	0
Ascomycot	Sordariom	Sordariales	Chaetomia	Humicola	grisea	215	107	140	0

Ascomycot	Dothideom	Venturiale	Sympoven	Neocoleroa	98	390	40	0
Ascomycot	Sordariom	Microascal	Microasca	Lophotrichus	41	29	0	0
Ascomycot	Sordariom	Microascal	Microasca	Gamsia	260	181	101	0
Ascomycot	Sordariom	Hypocreale	Nectriacea	Gibberella	187	136	177	0
Ascomycot	Sordariom	Diaporthal	Diaporthac	Diaporthe	0	0	0	0
Ascomycot	Eurotiomy	Phaeomon	Phaeomon	Xenocylindrosporium	0	425	28	0
Ascomycot	Eurotiomy	Phaeomon	Phaeomon	Xenocylindrosporium	0	337	0	0
Ascomycot	Dothideom	Capnodiale	Teratospha	Pseudoteratosphaeria	0	0	0	0
Ascomycot	Eurotiomy	Phaeomoniellales			6	272	108	0
Ascomycot	Eurotiomy	Eurotiales	Aspergillac	Aspergillus	45	0	0	6
Ascomycot	Sordariom	Chaetosph	Chaetosphaeriaceae		65	425	0	0
Ascomycot	Sordariom	Diaporthal	Diaporthac	Diaporthe	0	0	0	0
Ascomycot	Dothideom	Capnodiale	Teratosphaeriaceae		0	0	0	8
Ascomycot	Sordariom	Hypocreale	Ophiocord	Tolypocladium	0	0	0	0
Ascomycot	Dothideom	Capnodiale	Mycospha	Exopassalora	0	0	432	0
Ascomycot	Dothideom	Capnodiale	Mycospha	Stomiopeltis	31	214	229	0
Ascomycot	Dothideom	Pleosporal	Didymospl	Paraconiothyrium	0	0	0	0
Ascomycot	Dothideom	Capnodiale	Teratospha	Pseudoteratosphaeria	0	82	0	0
Ascomycot	Dothideom	Capnodiale	Mycosphaerellaceae		0	0	0	0
Ascomycot	Sordariom	Diaporthal	Diaporthac	Diaporthe	0	0	0	0
Ascomycot	Sordariom	Diaporthal	Diaporthac	Diaporthe	0	0	0	0
Ascomycot	Dothideom	Pleosporal	Sporormia	Preussia	100	145	48	4
Ascomycot	Dothideom	Capnodiale	Mycosphaerellaceae		0	0	0	0
Ascomycot	Dothideom	Capnodiale	Mycosphaerellaceae		150	228	66	0
Ascomycot	Pezizomyc	Pezizales	Pyronemat	Geopora	0	0	0	0
Ascomycot	Dothideom	Capnodiale	Mycospha	Madagascaromyces	0	0	0	0
Ascomycot	Dothideom	Capnodiale	Teratosphaeriaceae		293	0	0	0
Ascomycot	Dothideom	Pleosporal	Didymellaceae		0	0	0	0
Ascomycot	Dothideom	Pleosporal	Didymellac	Epicoccum	18	0	0	0
Ascomycot	Sordariom	Glomerella	Glomerella	Colletotrichum	24	20	16	0
Ascomycot	Dothideom	Capnodiale	Mycosphaerellaceae		0	0	0	0
Ascomycot	Sordariom	Hypocreale	Nectriacea	Dactylonectria	196	134	96	0
Ascomycot	Dothideom	Capnodiale	Mycospha	Neoceratosperma	0	0	0	0
Ascomycot	Sordariom	Hypocreale	Nectriaceae		231	78	112	0
Ascomycot	Sordariomycetes				0	0	0	184
Ascomycot	Dothideom	Pleosporales			0	0	0	0
Ascomycot	Sordariom	Diaporthal	Diaporthac	Diaporthe	0	0	0	0
Ascomycot	Dothideom	Capnodiale	Mycospha	Zasmidium	98	275	0	0
Ascomycot	Dothideom	Pleosporal	Massarina	Helminthosporium	0	379	0	0
Ascomycot	Sordariom	Hypocreale	Nectriacea	Lasionectria	204	100	92	0
Ascomycot	Sordariom	Diaporthal	Diaporthac	Diaporthe	0	0	0	0
Ascomycot	Sordariom	Hypocreale	Nectriacea	Gibberella	161	73	109	0
Ascomycot	Sordariom	Diaporthal	Gnomoniaceae		0	0	0	5
Ascomycot	Dothideom	Capnodiale	Teratospha	Neophaeothecoidea	130	63	184	0
Ascomycot	Sordariom	Diaporthal	Diaporthac	Diaporthe	0	0	0	377
Ascomycot	Dothideom	Pleosporal	Pleosporac	Bipolaris	0	0	0	0
Ascomycot	Sordariom	Hypocreale	Nectriacea	Neonectria	223	73	73	0
Ascomycot	Dothideom	Capnodiale	Teratospha	Pseudoteratosphaeria	0	0	0	0
Ascomycot	Eurotiomy	Onygenale	Onygenale	Zeloasperisporium	0	0	0	0
Ascomycot	Dothideom	Pleosporal	Didymospl	Paraconiothyrium	0	0	0	0

Ascomycot	Dothideom	Dothideom	Dothideom	Radulidium	157	165	0	0
Ascomycot	Sordariom	Diaporthal	Diaporthac	Diaporthe	0	22	26	0
Ascomycot	Dothideom	Capnodiale	Mycosphaerellaceae		96	100	115	0
Ascomycot	Eurotiomy	Eurotiales			44	61	13	5
Ascomycot	Dothideom	Capnodiale	Mycosphae	Madagascaromyces	0	0	0	0
Ascomycot	Sordariom	Hypocreale	Nectriacea	Neocosmospora	92	28	47	0
Ascomycot	Dothideom	Pleosporal	Didymospl	Pseudopithomyces	0	0	28	0
Ascomycot	Sordariom	Sordariales	Chaetomia	Botryotrichum	185	65	87	0
Ascomycot	Sordariom	Diaporthal	Diaporthac	Diaporthe	0	0	0	0
Ascomycot	Dothideom	Capnodiale	Mycosphae	Zasmidium	0	0	0	0
Ascomycot	Dothideom	Capnodiale	Mycosphaerellaceae		0	0	0	0
Ascomycot	Dothideom	Capnodiale	Mycosphae	Madagascaromyces	0	0	0	0
Ascomycot	Dothideom	Pleosporal	Pleosporac	Curvularia	0	0	0	0
Ascomycot	Dothideom	Capnodiale	Mycosphaerellaceae		0	9	0	0
Ascomycot	Dothideom	Capnodiales			14	0	307	0
Ascomycot	Dothideom	Pleosporal	Lophiostomataceae		26	0	0	0
Ascomycot	Eurotiomy	Phaeomon	Phaeomon	Xenocylindrosporium	42	0	0	5
Ascomycot	Sordariom	Trichospha	Trichospha	Nigrospora	0	0	0	0
Ascomycot	Dothideom	Capnodiale	Mycosphae	Stomiopeltis	0	0	312	0
Ascomycot	Dothideom	Myriangial	Elsinoacea	Elsinoe	126	57	114	0
Ascomycot	Sordariom	Chaetosph	Chaetosph	Pseudodinemasporiur	0	0	0	0
Ascomycot	Dothideom	Asterinales	Parmularia	Parmularia	0	0	0	0
Ascomycot	Dothideom	Capnodiale	Mycosphae	Paramycosphaerella	0	0	167	136
Ascomycot	Sordariom	Trichospha	Trichospha	Nigrospora	201	28	73	0
Ascomycot	Dothideom	Capnodiale	Teratosphaeriaceae		0	0	302	0
Ascomycot	Leotiomyc	Helotiales	Chaetomel	Pilidium	249	0	38	0
Ascomycot	Sordariom	Sordariales	Chaetomia	Botryotrichum	110	99	88	0
Ascomycot	Sordariom	Diaporthal	Diaporthac	Diaporthe	0	0	0	0
Ascomycot	Dothideom	Pleosporal	Phaeospha	Phaeosphaeria	0	0	0	0
Ascomycot	Sordariom	Hypocreale	Nectriacea	Fusarium	105	49	139	0
Ascomycot	Dothideom	Capnodiale	Teratosphaeriaceae		0	0	286	0
Ascomycot	Dothideom	Pleosporal	Didymellac	Didymella	0	0	0	0
Ascomycot	Sordariom	Diaporthal	Diaporthac	Diaporthe	0	0	0	0
Ascomycot	Dothideom	Capnodiale	Teratosphaeriaceae		0	249	0	0
Ascomycot	Eurotiomy	Chaetothyr	Chaetothyr	Strelitziana	0	0	0	0
Ascomycot	Dothideomycetes				0	0	270	0
Ascomycot	Leotiomyc	Helotiales	Helotiacea	Tetracladium	111	115	41	0
Ascomycot	Eurotiomy	Chaetothyr	Chaetothyr	Strelitziana	28	0	143	0
Ascomycot	Leotiomyc	Thelebolal	Pseudeuro	Pseudogymnoascus	0	0	0	0
Ascomycot	Sordariom	Hypocreale	Nectriacea	Fusicolla	150	47	63	0
Ascomycot	Dothideom	Capnodiale	Mycosphae	Madagascaromyces	0	0	0	0
Ascomycot	Sordariom	Diaporthal	Diaporthac	Diaporthe	0	28	0	0
Ascomycot	Sordariom	Diaporthal	Cryphonec	Mastigosporella	0	0	0	0
Ascomycot	Leotiomyc	Helotiales	Helotiales_	Triposporium	0	167	0	0
Ascomycot	Dothideom	Dothideale	Aureobasic	Aureobasidium	0	0	0	251
Ascomycot	Dothideom	Capnodiales			0	0	250	0
Ascomycot	Sordariom	Diaporthal	Diaporthac	Diaporthe	0	0	0	0
Ascomycot	Sordariomycetes				0	0	0	0
Ascomycot	Sordariom	Diaporthal	Diaporthac	Diaporthe	0	0	0	0
Ascomycot	Dothideom	Pleosporal	Sporormia	Preussia	0	0	0	0

Ascomycot	Dothideom	Pleosporal	Didymosph	Paraphaeosphaeria		0	0	0	0
Ascomycot	Dothideom	Capnodiale	Mycosphae	Neoceratosperma		0	0	0	0
Ascomycot	Sordariom	Hypocreale	Hypocreac	Trichoderma		0	0	0	0
Ascomycot	Dothideom	Capnodiales				235	3	0	0
Ascomycot	Dothideom	Capnodiale	Cladospori	Cladosporium		0	0	0	0
Ascomycot	Leotiomyc	Helotiales	Hyaloscyph	Crucellisporiopsis		0	236	0	0
Ascomycot	Leotiomyc	Erysiphales	Erysiphace	Blumeria		0	0	0	0
Ascomycot	Dothideom	Capnodiale	Mycosphaerellaceae			0	0	0	0
Ascomycot	Dothideom	Pleosporal	Teichospor	Magnibotryascoma		31	0	63	0
Ascomycot	Dothideom	Venturiales	Sympovent	Neocoleroa		0	229	0	0
Ascomycot	Dothideom	Pleosporal	Didymellac	Didymella		0	0	0	0
Ascomycot	Sordariom	Hypocreale	Nectriacea	Gibberella		0	0	0	0
Ascomycot	Sordariom	Sordariales	Lasiosphae	Schizothecium		126	33	67	0
Ascomycot	Sordariom	Hypocreale	Bionectriac	Bullanockia		0	0	0	0
Ascomycot	Dothideom	Botryospha	Botryospha	Neofusicoccum		0	0	0	0
Ascomycot	Dothideom	Pleosporal	Massarinac	Helminthosporium		86	0	84	0
Ascomycot	Sordariom	Diaporthal	Diaporthac	Diaporthe		0	0	25	0
Ascomycot	Sordariom	Diaporthal	Diaporthac	Diaporthe		79	0	0	0
Ascomycot	Dothideom	Capnodiale	Dissoconia	Uwebraunia		2	0	0	0
Ascomycot	Sordariom	Diaporthal	Diaporthac	Diaporthe		0	0	0	0
Ascomycot	Archaeorhizomycetes					38	17	0	0
Ascomycot	Sordariom	Glomerella	Glomerella	Colletotrichum		0	0	0	0
Ascomycot	Leotiomyc	Helotiales				7	0	0	0
Ascomycot	Dothideom	Capnodiale	Mycosphaerellaceae			66	33	90	0
Ascomycota						0	0	0	0
Ascomycot	Dothideomycetes					0	0	188	0
Ascomycot	Dothideom	Capnodiale	Teratosphaeriaceae			0	152	32	0
Ascomycot	Dothideom	Pleosporal	Didymellac	Nothophoma		0	0	0	0
Ascomycot	Dothideom	Capnodiales				40	0	0	0
Ascomycot	Dothideom	Capnodiale	Dissoconia	Uwebraunia		0	0	0	0
Ascomycot	Dothideom	Capnodiales				0	0	0	0
Ascomycot	Dothideom	Capnodiale	Mycosphae	Zasmidium		0	0	175	0
Ascomycot	Eurotiomy	Eurotiales	Aspergillac	Penicillium		21	0	0	6
Ascomycot	Dothideom	Capnodiale	Mycosphae	Paramycosphaerella		0	0	168	0
Ascomycot	Eurotiomy	Chaetothyr	Chaetothyr	Strelitziana		0	0	14	0
Ascomycot	Sordariom	Diaporthal	Diaporthac	Diaporthe		0	0	0	0
Ascomycot	Dothideom	Capnodiales				38	27	101	0
Ascomycot	Dothideom	Capnodiale	Mycosphaerellaceae			0	0	0	149
Ascomycot	Dothideom	Capnodiale	Teratospha	Xenoteratosphaeria		61	0	54	0
Ascomycot	Dothideom	Pleosporal	Massarinac	Helminthosporium		103	0	42	0
Ascomycot	Leotiomyc	Helotiales	Helotiacea	Tetracladium		88	32	42	0
Ascomycot	Sordariom	Diaporthal	Diaporthac	Diaporthe		0	0	0	0
Ascomycot	Sordariom	Diaporthal	Diaporthac	Diaporthe		0	0	0	0
Ascomycot	Sordariom	Trichospha	Trichospha	Nigrospora		0	0	0	0
Ascomycot	Eurotiomy	Phaeomon	Phaeomoniellaceae			22	91	48	0
Ascomycot	Sordariom	Hypocreale	Nectriacea	Fusarium	solani	99	34	27	0
Ascomycot	Dothideom	Venturiales	Sympovent	Neocoleroa		54	106	0	0
Ascomycot	Eurotiomy	Onygenale	Onygenale	Zeloasperisporium		0	0	38	0
Ascomycot	Leotiomyc	Helotiales	Dermateac	Pezicula		154	0	0	0
Ascomycot	Dothideom	Capnodiale	Teratospha	Neophaeothecoidea		0	0	158	0

Ascomycot	Dothideom	Capnodiales			0	0	158	0
Ascomycot	Dothideom	Dothideale	Aureobasic	Aureobasidium	0	0	0	0
Ascomycot	Leotiomyc	Thelebola	Thelebolac	Thelebolus	49	70	38	0
Ascomycot	Dothideom	Capnodiale	Mycospha	Stomiopeltis	0	0	157	0
Ascomycot	Sordariom	Hypocreale	Nectriacea	Cylindrocarpon	85	38	33	0
Ascomycot	Sordariom	Diaporthal	Diaporthac	Diaporthe	0	0	0	0
Ascomycot	Dothideom	Capnodiale	Mycosphaerellaceae		0	0	0	0
Ascomycot	Dothideom	Pleosporal	Sporormia	Preussia	155	0	0	0
Ascomycot	Eurotiomy	Eurotiales			23	0	0	0
Ascomycot	Leotiomyc	Helotiales			151	0	0	0
Ascomycot	Dothideom	Capnodiale	Mycosphaerellaceae		18	46	77	0
Ascomycot	Dothideom	Pleosporal	Didymellac	Epicoccum	0	0	67	0
Ascomycot	Sordariom	Hypocreale	Hypocreale	Sarocladium	9	0	5	0
Ascomycot	Eurotiomy	Chaetothyı	Chaetothyı	Strelitziana	9	0	0	0
Ascomycot	Dothideom	Pleosporal	Phaeospha	Neosetophoma	6	59	0	0
Ascomycot	Dothideomycetes				0	150	0	0
Ascomycot	Dothideom	Capnodiale	Mycospha	Stomiopeltis	0	25	123	0
Ascomycot	Sordariom	Diaporthal	Diaporthac	Diaporthe	0	0	0	0
Ascomycot	Sordariom	Diaporthal	Diaporthac	Diaporthe	0	0	0	0
Ascomycot	Sordariom	Diaporthal	Diaporthac	Diaporthe	0	0	0	0
Ascomycot	Dothideom	Myriangiales			37	0	95	0
Ascomycot	Sordariom	Xylariales	Xylariacea	Hypoxylon	0	0	0	2
Ascomycot	Dothideom	Pleosporal	Phaeospha	Phaeosphaeria	0	0	0	0
Ascomycot	Sordariom	Glomerella	Plectospha	Plectosphaerella	56	47	0	9
Ascomycot	Sordariom	Hypocreale	Cordycipita	Simplicillium	0	0	0	0
Ascomycot	Dothideom	Pleosporal	Phaeosphaeriaceae		0	0	0	0
Ascomycot	Sordariom	Xylariales	Microdoch	Microdochium	47	57	35	0
Ascomycot	Sordariom	Diaporthal	Diaporthac	Diaporthe	0	0	0	0
Ascomycot	Sordariom	Microascal	Microasca	Canariomyces	0	0	0	0
Ascomycot	Dothideom	Capnodiale	Mycospha	Pallidocercospora	0	0	0	0
Ascomycot	Dothideom	Pleosporal	Didymellac	Epicoccum	89	0	0	0
Ascomycot	Eurotiomy	Eurotiales	Aspergillac	Penicillium	92	33	11	0
Ascomycot	Sordariom	Trichospha	Trichospha	Nigrospora	0	0	0	0
Ascomycot	Sordariomycetes				3	0	0	0
Ascomycot	Eurotiomy	Phaeomon	Phaeomoniellaceae		0	128	4	0
Ascomycot	Sordariom	Sordariales	Sordariace	Gelasinospora	0	131	0	0
Ascomycot	Dothideom	Pleosporal	Didymospł	Paraconiothyrium	0	0	0	0
Ascomycot	Dothideom	Capnodiale	Teratospha	Catenulostroma	22	0	13	0
Ascomycot	Dothideom	Capnodiale	Teratosphaeriaceae		0	0	0	0
Ascomycot	Sordariom	Xylariales	Xylariacea	Xylaria	0	0	0	0
Ascomycot	Sordariom	Sordariales	Chaetomiaceae		0	23	0	0
Ascomycot	Sordariom	Diaporthal	Diaporthac	Diaporthe	0	0	0	0
Ascomycot	Dothideom	Capnodiale	Mycosphaerellaceae		37	23	64	0
Ascomycot	Sordariom	Sordariales			0	0	0	0
Ascomycot	Dothideom	Capnodiale	Mycospha	Stomiopeltis	0	0	123	0
Ascomycot	Sordariom	Diaporthal	Diaporthaceae		0	0	0	0
Ascomycot	Sordariom	Glomerella	Glomerellaceae		57	0	0	0
Ascomycot	Eurotiomy	Eurotiales	Aspergillaceae		0	0	0	0
Ascomycot	Dothideom	Capnodiale	Mycospha	Geastrumia	0	95	22	0
Ascomycot	Leotiomyc	Thelebola	Pseudeuro	Pseudogymnoascus	70	21	25	0

Ascomycot	Leotiomyc	Helotiales	Helotiacea	Tetracladium	34	21	38	0
Ascomycot	Sordariom	Diaporthal	Diaporthac	Diaporthe	0	0	5	0
Ascomycot	Sordariom	Trichospha	Trichospha	Nigrospora	0	0	0	0
Ascomycot	Sordariom	Diaporthal	Diaporthac	Diaporthe	0	0	0	0
Ascomycot	Sordariom	Glomerella	Glomerella	Colletotrichum	0	67	0	0
Ascomycot	Sordariom	Diaporthal	Diaporthac	Diaporthe	0	0	0	0
Ascomycot	Sordariom	Diaporthales			0	0	0	0
Ascomycot	Dothideom	Capnodiale	Teratosphaeriaceae		0	0	0	0
Ascomycot	Dothideom	Pleosporal	Didymospl	Paracamarosporium	0	0	0	10
Ascomycot	Dothideom	Capnodiale	Mycosphaerellaceae		108	0	0	0
Ascomycot	Dothideom	Pleosporal	Didymellac	Neoascochyta	0	0	0	0
Ascomycot	Dothideom	Capnodiale	Mycosphaerellaceae		0	0	0	0
Ascomycot	Dothideom	Dothideale	Aureobasic	Aureobasidium	16	11	62	0
Ascomycot	Lecanorom	Ostropales	Stictidacea	Cyanodermella	11	87	0	0
Ascomycot	Sordariom	Diaporthal	Diaporthac	Diaporthe	0	28	0	0
Ascomycot	Sordariomycetes				0	0	0	8
Ascomycot	Eurotiomy	Onygenale	Onygenale	Zeloasperisporium	0	0	0	0
Ascomycot	Dothideom	Capnodiale	Mycospha	Zasmidium	0	105	0	0
Ascomycot	Dothideom	Pleosporal	Didymellaceae		0	0	0	0
Ascomycot	Sordariom	Trichospha	Trichospha	Nigrospora oryzae	0	0	0	0
Ascomycot	Eurotiomy	Eurotiales	Trichocom	Talaromyces	0	0	0	0
Ascomycot	Dothideom	Venturiales	Sympovent	Neocoleroa	80	0	22	0
Ascomycot	Dothideom	Capnodiale	Mycosphaerellaceae		0	0	0	0
Ascomycot	Leotiomyc	Helotiales	Helotiaceae		36	38	27	0
Ascomycot	Dothideom	Capnodiale	Mycospha	Zasmidium	30	40	0	0
Ascomycot	Dothideom	Capnodiale	Mycospha	Ramularia	0	0	0	0
Ascomycot	Dothideom	Asterinales	Parmularia	Parmularia	0	0	0	0
Ascomycot	Dothideom	Capnodiale	Capnodiale	Microcyclospora	16	10	73	0
Ascomycot	Sordariom	Trichospha	Trichospha	Nigrospora	0	0	0	0
Ascomycot	Sordariom	Hypocreale	Nectriacea	Neocosmospora	0	40	0	0
Ascomycot	Dothideom	Capnodiales			0	0	0	0
Ascomycot	Dothideom	Pleosporal	Didymellac	Epicoccum	0	0	0	0
Ascomycot	Sordariom	Diaporthal	Diaporthac	Diaporthe	0	0	0	0
Ascomycot	Dothideom	Capnodiale	Teratosphaeriaceae		76	0	9	0
Ascomycot	Dothideom	Venturiales	Sympovent	Ochroconis	0	0	0	0
Ascomycot	Dothideom	Pleosporal	Teichospor	Magnibotryascoma	0	0	0	0
Ascomycot	Sordariom	Diaporthal	Diaporthac	Diaporthe	0	0	0	0
Ascomycot	Dothideom	Capnodiale	Dissoconia	Uwebraunia	0	0	0	0
Ascomycot	Dothideom	Pleosporal	Didymospl	Kalmusia	0	0	0	0
Ascomycot	Sordariom	Diaporthal	Diaporthac	Diaporthe	0	0	0	0
Ascomycot	Sordariom	Diaporthal	Schizoparn	Coniella	35	13	44	0
Ascomycot	Dothideom	Capnodiale	Mycospha	Australosphaerella	14	0	53	0
Ascomycot	Dothideom	Pleosporal	Massarinac	Helminthosporium	0	92	0	0
Ascomycot	Dothideom	Capnodiale	Mycosphaerellaceae		0	0	0	0
Ascomycot	Sordariom	Diaporthal	Cryphonec	Mastigosporella	0	0	0	0
Ascomycot	Dothideomycetes				0	0	90	0
Ascomycot	Dothideom	Pleosporal	Lophiostomataceae		0	0	90	0
Ascomycot	Dothideom	Pleosporal	Cucurbitari	Pyrenochaetopsis	35	28	25	0
Ascomycot	Dothideom	Myriangial	Elsinoaceae		3	0	0	0
Ascomycot	Dothideom	Botryospha	Planistrom	Ramimonilia	21	0	59	0

Ascomycot	Sordariom	Xylariales	Xylariaceae	Muscodor	0	0	3	0
Ascomycot	Sordariom	Sordariales	Lasiosphae	Cladorrhinum	28	43	14	0
Ascomycot	Sordariom	Diaporthal	Diaporthac	Diaporthe	0	0	0	0
Ascomycot	Sordariom	Hypocreale	Nectriacea	Neocosmospora	47	16	19	0
Ascomycota					0	82	0	0
Ascomycot	Dothideom	Capnodiale	Teratospha	Pseudoteratosphaeria	0	0	0	0
Ascomycot	Dothideom	Capnodiale	Teratosphaeriaceae		0	0	0	0
Ascomycot	Dothideom	Asterinales	Parmularia	Parmularia	0	0	0	0
Ascomycot	Dothideom	Capnodiale	Teratospha	Euteratosphaeria	71	0	10	0
Ascomycot	Sordariom	Trichospha	Trichospha	Nigrospora	0	0	0	0
Ascomycot	Dothideom	Pleosporal	Didymellac	Epicoccum	0	0	0	0
Ascomycot	Sordariom	Glomerella	Plectosphaerellaceae		55	25	0	0
Ascomycot	Dothideom	Pleosporal	Didymellac	Didymella	0	0	0	0
Ascomycot	Eurotiomy	Onygenale	Onygenale	Zeloasperisporium	0	0	0	0
Ascomycot	Dothideom	Capnodiales			0	0	0	0
Ascomycot	Sordariom	Diaporthal	Diaporthac	Diaporthe	0	0	0	0
Ascomycot	Dothideom	Capnodiale	Teratosphaeriaceae		17	13	49	0
Ascomycot	Dothideom	Capnodiale	Mycosphae	Geastrumia	0	0	63	0
Ascomycot	Dothideom	Capnodiale	Teratospha	Penidiella	0	0	0	0
Ascomycot	Dothideom	Pleosporal	Didymosph	Kalmusia	0	0	0	0
Ascomycot	Sordariom	Hypocreale	Clavicipitac	Metarhiziu robertsii	66	4	8	0
Ascomycot	Eurotiomy	Chaetothyriales			0	21	28	0
Ascomycot	Dothideom	Capnodiale	Mycosphae	Paramycosphaerella	0	0	78	0
Ascomycot	Dothideom	Capnodiale	Mycosphae	Phaeophleospora	0	0	0	0
Ascomycot	Dothideom	Capnodiale	Mycosphaerellaceae		0	0	0	0
Ascomycot	Dothideom	Capnodiale	Mycosphae	Teratoramularia	0	0	0	0
Ascomycot	Sordariom	Sordariales			13	0	0	4
Ascomycot	Sordariom	Glomerella	Glomerella	Colletotrichum	0	0	0	0
Ascomycot	Leotiomyc	Helotiales			75	0	0	0
Ascomycot	Eurotiomy	Onygenale	Onygenale	Zeloasperisporium	0	0	5	70
Ascomycot	Dothideom	Pleosporal	Pleosporac	Curvularia	0	0	0	0
Ascomycot	Dothideom	Capnodiale	Teratospha	Pseudoteratosphaeria	0	0	0	0
Ascomycot	Sordariom	Glomerella	Glomerella	Colletotrichum	0	0	0	0
Ascomycot	Dothideom	Capnodiale	Mycosphae	Zasmidium	0	0	0	0
Ascomycot	Eurotiomy	Onygenale	Onygenale	Zeloasperisporium	0	0	0	0
Ascomycot	Dothideom	Hysteriales	Hysteriace	Gloniopsis	10	0	0	0
Ascomycot	Dothideom	Capnodiale	Mycosphae	Neoceratosperma	0	0	0	0
Ascomycot	Dothideom	Capnodiale	Mycosphae	Zasmidium	0	0	0	0
Ascomycot	Dothideom	Capnodiale	Teratosphaeriaceae		0	0	0	0
Ascomycot	Sordariom	Diaporthal	Cryphonectriaceae		0	0	0	0
Ascomycot	Dothideom	Capnodiales			0	36	0	0
Ascomycot	Dothideom	Capnodiales			0	0	4	0
Ascomycot	Sordariom	Diaporthal	Diaporthac	Diaporthe	0	0	0	0
Ascomycot	Sordariom	Diaporthal	Diaporthaceae		0	0	0	0
Ascomycot	Dothideom	Pleosporal	Didymosph	Paraconiothyrium	0	0	0	0
Ascomycot	Sordariom	Sordariales	Lasiosphae	Cercophora	60	11	0	0
Ascomycot	Sordariom	Sordariales	Lasiosphae	Apodus	56	6	9	0
Ascomycot	Sordariom	Hypocreale	Nectriaceae		42	16	13	0
Ascomycot	Leotiomyc	Helotiales	Sclerotinia	Moellerodiscus	0	71	0	0
Ascomycot	Dothideom	Botryospha	Planistrom	Ramimonilia	0	14	51	0

Ascomycot	Eurotiomy	Eurotiales	Aspergillac	Penicillium cairnsense	0	0	0	0
Ascomycot	Sordariom	Hypocreale	Hypocreac	Trichoderma	21	42	7	0
Ascomycot	Sordariom	Hypocreale	Nectriaceae		0	19	0	0
Ascomycot	Dothideom	Botryospha	Phyllostict	Phyllosticta	0	0	0	31
Ascomycot	Dothideom	Capnodiale	Mycosphae	Paramycosphaerella	0	0	0	0
Ascomycot	Sordariom	Hypocreale	Nectriacea	Neocosmospora	0	0	0	0
Ascomycot	Sordariom	Diaporthal	Diaporthac	Diaporthe	0	0	0	0
Ascomycot	Sordariom	Diaporthal	Diaporthac	Diaporthe	0	0	0	0
Ascomycot	Dothideom	Pleosporales			6	48	0	0
Ascomycot	Sordariom	Diaporthal	Diaporthaceae		0	0	0	0
Ascomycot	Sordariom	Xylariales	Xylariaceae	Daldinia	0	0	3	0
Ascomycot	Dothideom	Pleosporal	Didymellac	Didymella	0	0	0	0
Ascomycot	Pezizomyc	Pezizales	Pyronemataceae		0	66	0	0
Ascomycot	Dothideom	Pleosporal	Sporormia	Preussia	0	0	0	0
Ascomycot	Dothideom	Pleosporal	Didymellac	Boeremia	0	0	0	0
Ascomycot	Dothideom	Capnodiale	Mycosphae	Zasmidium	0	0	0	0
Ascomycot	Dothideom	Pleosporal	Pleosporal	Parapyrenochaeta	4	0	61	0
Ascomycot	Eurotiomy	Chaetothyr	Chaetothyr	Ceramothyrium	0	0	0	0
Ascomycot	Dothideom	Pleosporal	Didymellaceae		0	0	0	0
Ascomycot	Dothideom	Capnodiale	Teratospha	Catenulostroma	28	0	22	0
Ascomycota					0	64	0	0
Ascomycot	Dothideom	Capnodiale	Capnodiac	Capnodium	23	9	31	0
Ascomycot	Dothideom	Capnodiale	Cladospori	Cladosporium	10	0	0	0
Ascomycot	Dothideom	Pleosporal	Didymosph	Paraconiothyrium	0	0	0	0
Ascomycot	Dothideom	Capnodiales			62	0	0	0
Ascomycot	Eurotiomy	Chaetothyriales			62	0	0	0
Ascomycot	Dothideomycetes				0	62	0	0
Ascomycot	Sordariom	Hypocreale	Hypocreale	Trichothecium	0	0	0	0
Ascomycot	Sordariomycetes				61	0	0	0
Ascomycot	Dothideom	Capnodiale	Mycosphae	Geastrumia	21	8	32	0
Ascomycot	Eurotiomy	Chaetothyr	Chaetothyr	Strelitziana	0	54	0	7
Ascomycot	Sordariom	Xylariales	Amphispha	Amphisphaeria	0	9	0	0
Ascomycot	Sordariom	Diaporthal	Diaporthac	Diaporthe	0	0	0	0
Ascomycot	Sordariom	Hypocreale	Hypocreac	Trichoderma	15	3	0	0
Ascomycot	Dothideom	Pleosporal	Periconiac	Periconia	7	8	0	0
Ascomycot	Eurotiomy	Eurotiales	Trichocom	Talaromyces	0	37	0	11
Ascomycot	Sordariom	Chaetosphaeriales			0	26	0	0
Ascomycot	Dothideom	Pleosporal	Phaeospha	Setophoma	0	0	0	0
Ascomycot	Dothideom	Capnodiale	Mycosphae	Zasmidium	59	0	0	0
Ascomycot	Sordariom	Hypocreale	Stachybotr	Stachybotrys	24	11	24	0
Ascomycot	Dothideom	Dothideom	Dothideom	Gonatophragmium	0	59	0	0
Ascomycot	Leotiomyc	Helotiales	Hyaloscyph	Proliferodiscus	0	0	0	0
Ascomycot	Sordariom	Hypocreales			22	12	24	0
Ascomycot	Dothideom	Pleosporal	Massarinac	Helminthosporium	0	58	0	0
Ascomycot	Sordariom	Diaporthal	Diaporthac	Diaporthe	0	0	0	0
Ascomycot	Sordariom	Xylariales	Xylariaceae	Muscodor	0	0	0	0
Ascomycot	Eurotiomy	Phaeomon	Phaeomon	Neophaeomoniella	0	0	0	0
Ascomycot	Dothideom	Asterinales	Parmularia	Parmularia	0	0	0	0
Ascomycot	Dothideom	Tubeufiale	Tubeufiaceae		0	0	0	0
Ascomycot	Leotiomyc	Helotiales	Hyaloscyph	Crucellisporiopsis	57	0	0	0

Ascomycot	Dothideom	Botryosphа	Botryosphа	Pseudofusicoccum		0	14	0	0
Ascomycot	Sordariom	Diaporthal	Diaporthac	Diaporthe		0	0	0	0
Ascomycot	Dothideom	Dothideom	Muyocopro	Muyocopron		0	0	0	0
Ascomycot	Dothideom	Pleosporal	Teichospor	Magnibotryascoma		0	0	0	0
Ascomycot	Sordariom	Diaporthal	Diaporthac	Diaporthe		0	0	0	0
Ascomycot	Sordariom	Sordariales	Sordariace	Neurospora		0	0	0	0
Ascomycot	Dothideom	Capnodiale	Mycosphae	Neoceratosperma		0	0	0	0
Ascomycot	Dothideom	Capnodiale	Teratosphа	Xenophacidiella		33	0	22	0
Ascomycot	Dothideom	Capnodiale	Teratosphaeriaceae			15	14	0	0
Ascomycot	Sordariom	Diaporthal	Diaporthac	Diaporthe		0	0	0	0
Ascomycot	Pezizomyc	Pezizales	Pyronemat	Geopora		0	0	0	0
Ascomycot	Sordariom	Xylariales	Amphisphа	Lepteutypa		0	0	0	0
Ascomycot	Dothideom	Pleosporal	Pleosporal	Nigrograna		0	0	0	0
Ascomycot	Dothideom	Capnodiale	Teratosphа	Neophaeothecoidea		54	0	0	0
Ascomycot	Dothideom	Capnodiale	Teratosphа	Parapenidiella		28	9	17	0
Ascomycot	Eurotiomy	Eurotiales	Aspergillac	Aspergillus		15	0	0	0
Ascomycota						0	54	0	0
Ascomycot	Sordariom	Diaporthal	Diaporthac	Diaporthe		0	0	0	0
Ascomycot	Sordariom	Diaporthal	Diaporthac	Diaporthe		0	0	0	0
Ascomycot	Dothideom	Pleosporal	Teichospor	Magnibotryascoma		33	12	0	0
Ascomycot	Sordariom	Hypocreale	Nectriacea	Gibberella		32	0	21	0
Ascomycot	Archaeorhi	Archaeorhi	Archaeorhi	Archaeorhizomyces		14	10	0	0
Ascomycot	Dothideom	Pleosporal	Didymellac	Didymella		0	28	0	0
Ascomycot	Sordariom	Diaporthal	Diaporthac	Diaporthe		0	0	0	0
Ascomycot	Dothideom	Pleosporal	Didymospl	Kalmusia		0	0	0	0
Ascomycot	Sordariom	Hypocreale	Hypocreale	Sarocladium		0	0	0	0
Ascomycot	Dothideom	Capnodiale	Mycosphae	Madagascaromyces		0	0	0	0
Ascomycot	Dothideom	Capnodiale	Mycosphaerellaceae			0	0	0	0
Ascomycot	Sordariom	Diaporthal	Diaporthac	Diaporthe		0	0	0	0
Ascomycot	Dothideom	Pleosporal	Massarinac	Helminthosporium		0	52	0	0
Ascomycot	Dothideom	Capnodiale	Mycosphaerellaceae			0	0	52	0
Ascomycot	Eurotiomy	Chaetothyriales				0	0	29	0
Ascomycot	Dothideom	Capnodiales				0	0	17	0
Ascomycot	Sordariom	Diaporthal	Diaporthac	Diaporthe		0	0	0	0
Ascomycot	Sordariom	Glomerella	Plectospha	Paragibellulopsis		0	0	0	0
Ascomycot	Dothideom	Capnodiales				0	0	0	0
Ascomycot	Eurotiomy	Phaeomon	Phaeomon	Xenocylindrosporium		0	0	0	0
Ascomycot	Dothideom	Pleosporal	Leptosphae	Leptosphaeria		20	7	23	0
Ascomycot	Dothideom	Pleosporal	Pleosporac	Bipolaris	yamadae	11	0	0	0
Ascomycot	Sordariom	Diaporthal	Diaporthac	Diaporthe		0	5	0	17
Ascomycot	Sordariom	Diaporthal	Diaporthac	Diaporthe		0	0	0	0
Ascomycot	Eurotiomy	Eurotiales	Aspergillac	Aspergillus		0	0	0	0
Ascomycot	Dothideom	Capnodiale	Teratosphaeriaceae			0	0	0	0
Ascomycot	Sordariom	Diaporthal	Diaporthac	Diaporthe		0	0	0	0
Ascomycota						49	0	0	0
Ascomycot	Sordariom	Hypocreale	Cordycipita	Simplicillium		0	0	0	7
Ascomycot	Sordariom	Hypocreale	Hypocreales_fam_Incertae_sedis			0	0	0	0
Ascomycot	Sordariom	Diaporthal	Diaporthac	Diaporthe		0	0	0	0
Ascomycot	Sordariom	Microascal	Microascac	Cephalotri	nanum	40	8	0	0
Ascomycot	Dothideomycetes					0	48	0	0

Ascomycot	Dothideom	Pleosporal	Phaeospha	Setophoma		0	0	0	40
Ascomycot	Sordariom	Glomerella	Glomerellaceae			0	0	0	0
Ascomycot	Sordariom	Sordariales	Lasiosphae	Schizothecium		41	0	6	0
Ascomycot	Sordariom	Diaporthal	Diaporthac	Diaporthe		0	47	0	0
Ascomycot	Dothideom	Capnodiale	Mycosphae	Zasmidium		0	0	47	0
Ascomycot	Dothideom	Capnodiale	Mycosphaerellaceae			0	0	0	0
Ascomycot	Dothideom	Pleosporal	Lophiostomataceae			0	0	0	0
Ascomycot	Dothideom	Pleosporal	Pleosporac	Curvularia	intermedia	0	0	0	0
Ascomycot	Sordariom	Hypocreale	Ophiocord	Hirsutella		0	0	0	0
Ascomycot	Dothideom	Pleosporal	Teichospor	Magnibotryascoma		0	0	0	0
Ascomycot	Dothideom	Capnodiale	Mycosphaerellaceae			0	0	0	0
Ascomycot	Eurotiomy	Chaetothyr	Chaetothyr	Hermetothecium		4	0	42	0
Ascomycot	Sordariom	Diaporthal	Diaporthac	Diaporthe		0	0	0	0
Ascomycot	Dothideom	Botryospha	Phyllostict	Phyllosticta		0	0	0	0
Ascomycot	Sordariomycetes					0	0	0	0
Ascomycot	Dothideom	Capnodiale	Mycosphaerellaceae			0	0	0	0
Ascomycot	Sordariom	Diaporthal	Diaporthac	Diaporthe		0	0	0	0
Ascomycot	Dothideom	Pleosporal	Didymellaceae			45	0	0	0
Ascomycot	Dothideom	Capnodiale	Teratospha	Teratosphaericola		0	11	19	0
Ascomycot	Sordariom	Diaporthal	Diaporthac	Diaporthe		0	0	0	0
Ascomycot	Dothideom	Capnodiale	Mycosphaerellaceae			0	0	0	0
Ascomycot	Sordariom	Glomerella	Glomerella	Colletotrichum		0	0	0	0
Ascomycot	Eurotiomy	Chaetothyr	Chaetothyr	Strelitziana		0	0	0	0
Ascomycot	Dothideom	Pleosporal	Didymospl	Kalmusia		0	0	0	0
Ascomycot	Eurotiomy	Eurotiales	Aspergillac	Monascus		6	0	0	0
Ascomycot	Lecanorom	Ostropales	Stictidaceae			0	0	44	0
Ascomycot	Dothideom	Pleosporal	Pleosporac	Curvularia		0	0	0	0
Ascomycot	Sordariom	Trichospha	Trichospha	Nigrospora		0	0	0	0
Ascomycot	Dothideom	Botryospha	Botryospha	Neofusicoccum		0	0	0	0
Ascomycot	Dothideom	Pleosporales				0	0	0	0
Ascomycot	Dothideom	Capnodiale	Teratosphaeriaceae			43	0	0	0
Ascomycot	Sordariom	Sordariales	Chaetomiaceae			18	18	7	0
Ascomycot	Sordariom	Diaporthal	Diaporthac	Diaporthe		0	43	0	0
Ascomycot	Eurotiomy	Chaetothyr	Trichomeri	Trichomerium		0	0	43	0
Ascomycot	Dothideom	Capnodiale	Teratosphaeriaceae			0	0	0	43
Ascomycot	Dothideom	Pleosporal	Pleosporac	Alternaria		0	0	0	0
Ascomycot	Dothideom	Pleosporal	Massarinac	Helminthosporium		0	0	0	0
Ascomycot	Dothideomycetes					42	0	0	0
Ascomycot	Sordariom	Diaporthal	Diaporthac	Diaporthe		0	0	0	0
Ascomycot	Eurotiomy	Chaetothyr	Herpotrich	Exophiala		25	0	0	0
Ascomycot	Sordariom	Glomerella	Plectospha	Verticillium		15	15	11	0
Ascomycot	Sordariom	Sordariales				0	0	0	0
Ascomycot	Sordariom	Diaporthal	Diaporthac	Diaporthe		0	0	0	0
Ascomycot	Dothideom	Capnodiale	Teratospha	Xenoteratosphaeria		0	0	0	0
Ascomycot	Sordariom	Hypocreale	Hypocreale	Acremonium		0	0	0	0
Ascomycot	Sordariom	Hypocreale	Ophiocordycipitaceae			0	0	0	0
Ascomycot	Sordariom	Xylariales	Diatrypace	Peroneutypa		0	0	0	0
Ascomycot	Eurotiomy	Chaetothyr	Chaetothyr	Strelitziana		0	0	0	0
Ascomycot	Leotiomyc	Thelebolal	Pseudeuro	Pseudogymnoascus		17	0	22	0
Ascomycot	Saccharom	Saccharom	Saccharom	Issatchenki	orientalis	5	0	0	0

Ascomycot	Eurotiomy	Chaetothyı	Chaetothyriales_fam_Incertae_se		0	11	28	0
Ascomycot	Sordariom	Diaporthal	Diaporthac	Diaporthe	0	0	0	0
Ascomycot	Sordariomycetes				0	0	0	0
Ascomycot	Dothideom	Capnodiale	Cladospori	Cladosporium	0	0	0	0
Ascomycot	Dothideom	Capnodiale	Teratospha	Neophaeothecoidea	0	0	0	0
Ascomycot	Dothideom	Capnodiale	Teratosphaeriaceae		0	0	0	0
Ascomycot	Eurotiomy	Eurotiales	Aspergillaceae		0	0	0	0
Ascomycot	Dothideom	Capnodiale	Mycosphaerellaceae		0	0	0	0
Ascomycot	Sordariom	Trichospha	Trichospha	Nigrospora	38	0	0	0
Ascomycot	Sordariom	Sordariales	Chaetomia	Humicola	12	0	0	0
Ascomycot	Sordariom	Chaetosph	Chaetosphaeriaceae		0	0	0	35
Ascomycot	Sordariom	Diaporthal	Diaporthac	Diaporthe	0	0	0	0
Ascomycot	Eurotiomy	Eurotiales	Trichocom	Talaromyces	0	0	0	0
Ascomycot	Sordariom	Chaetosph	Chaetosph	Thozetella	0	0	0	0
Ascomycot	Eurotiomy	Chaetothyriales			0	0	0	0
Ascomycot	Dothideom	Pleosporales			0	0	0	0
Ascomycot	Dothideom	Capnodiale	Mycosphaerellaceae		0	0	0	0
Ascomycot	Dothideom	Capnodiale	Mycosphaerellaceae		0	0	0	0
Ascomycot	Eurotiomy	Chaetothyı	Chaetothyı	Strelitziana	0	0	0	0
Ascomycot	Eurotiomy	Eurotiales	Trichocom	Talaromyces	37	0	0	0
Ascomycot	Eurotiomy	Eurotiales	Aspergillac	Aspergillus	25	0	0	0
Ascomycot	Sordariom	Sordariales	Chaetomia	Botryotrichum	20	0	17	0
Ascomycot	Dothideom	Pleosporales			10	0	13	0
Ascomycot	Sordariom	Xylariales	Xylariacea	Hypoxylon	0	14	0	0
Ascomycot	Eurotiomy	Chaetothyı	Herpotrich	Veronaea	0	0	0	31
Ascomycota					0	0	0	0
Ascomycot	Sordariom	Diaporthal	Diaporthac	Diaporthe	0	0	0	0
Ascomycot	Dothideom	Pleosporal	Didymellac	Didymella	0	0	0	0
Ascomycot	Leotiomyc	Helotiales	Hyaloscypl	Proliferodiscus	0	0	0	0
Ascomycot	Dothideom	Pleosporales			0	0	0	0
Ascomycot	Dothideom	Capnodiale	Mycospha	Geastrumia	15	0	21	0
Ascomycota					0	36	0	0
Ascomycot	Saccharom	Saccharom	Debaryom	Meyerozyma	0	7	7	0
Ascomycot	Sordariom	Hypocreale	Nectriacea	Fusarium	0	0	0	0
Ascomycot	Sordariom	Chaetosph	Chaetosphaeriaceae		0	0	0	0
Ascomycot	Eurotiomy	Phaeomon	Phaeomon	Neophaeomoniella	0	0	0	0
Ascomycot	Dothideom	Capnodiale	Dissoconia	Uwebraunia	0	0	0	0
Ascomycot	Sordariom	Hypocreale	Bionectria	Clonostach sp	35	0	0	0
Ascomycot	Dothideom	Pleosporales			23	12	0	0
Ascomycot	Sordariom	Hypocreale	Nectriaceae		15	7	7	0
Ascomycot	Eurotiomy	Chaetothyı	Chaetothyı	Strelitziana	0	20	15	0
Ascomycot	Dothideom	Capnodiale	Schizothyri	Johansonia	0	0	35	0
Ascomycot	Dothideom	Capnodiale	Teratospha	Xenopenidiella	0	0	0	0
Ascomycot	Eurotiomy	Eurotiales	Aspergillac	Aspergillus	0	0	0	0
Ascomycot	Dothideom	Capnodiale	Teratospha	Lapidomyces	0	0	0	0
Ascomycot	Dothideom	Capnodiales			30	0	4	0
Ascomycota					0	34	0	0
Ascomycot	Dothideom	Capnodiales			0	0	34	0
Ascomycot	Sordariom	Diaporthal	Diaporthac	Diaporthe	0	0	0	0
Ascomycot	Eurotiomy	Onygenale	Onygenale	Zeloasperisporium	0	0	0	0

Ascomycot	Dothideom	Dothideom	Dothideom	Radulidium	19	0	14	0
Ascomycot	Dothideom	Pleosporal	Teichospor	Magnibotryascoma	0	33	0	0
Ascomycot	Dothideomycetes				0	33	0	0
Ascomycot	Sordariom	Xylariales			0	8	0	0
Ascomycot	Dothideom	Pleosporal	Phaeospha	Phaeosphaeria	0	0	0	0
Ascomycot	Sordariom	Xylariales	Xylariaceae	Muscodor	0	0	0	0
Ascomycot	Dothideom	Capnodiale	Teratosphaeriaceae		0	0	0	0
Ascomycot	Dothideom	Capnodiales			0	0	0	0
Ascomycot	Sordariom	Diaporthal	Diaporthac	Diaporthe	0	0	0	0
Ascomycot	Dothideom	Capnodiale	Teratospha	Xenoteratosphaeria	32	0	0	0
Ascomycot	Dothideom	Capnodiale	Mycosphaerellaceae		32	0	0	0
Ascomycot	Eurotiomy	Chaetothyriales			32	0	0	0
Ascomycot	Sordariom	Sordariales	Lasiosphaeriaceae		28	0	0	0
Ascomycot	Dothideom	Capnodiale	Mycosphae	Geastrumia	0	32	0	0
Ascomycot	Sordariom	Hypocreale	Clavicipitaceae		0	0	0	0
Ascomycot	Sordariom	Xylariales	Xylariaceae	Muscodor	0	0	0	0
Ascomycot	Sordariom	Hypocreale	Hypocreac	Trichoderma	0	0	0	0
Ascomycot	Eurotiomy	Eurotiales	Aspergillac	Penicillium	0	0	0	0
Ascomycot	Dothideom	Pleosporal	Phaeospha	Phaeosphaeria	0	0	0	0
Ascomycot	Dothideom	Capnodiale	Teratospha	Xenoteratosphaeria	0	0	0	0
Ascomycot	Sordariom	Glomerella	Plectospha	Lectera	0	31	0	0
Ascomycot	Sordariom	Diaporthal	Diaporthaceae		0	0	0	0
Ascomycot	Leotiomyc	Erysiphales	Erysiphace	Blumeria	0	0	0	0
Ascomycot	Dothideom	Capnodiales			0	0	0	0
Ascomycot	Sordariom	Diaporthal	Diaporthac	Diaporthe	0	0	0	0
Ascomycot	Eurotiomy	Eurotiales	Trichocom	Talaromyces	0	30	0	0
Ascomycot	Dothideom	Pleosporal	Didymellac	Nothophoma	0	30	0	0
Ascomycot	Dothideom	Dothideale	Aureobasic	Aureobasidium	0	11	0	0
Ascomycot	Sordariom	Diaporthal	Diaporthac	Diaporthe	0	0	0	0
Ascomycot	Dothideom	Capnodiale	Teratospha	Xenoteratosphaeria	0	0	0	0
Ascomycot	Dothideom	Capnodiale	Cladospori	Cladosporium	0	0	0	0
Ascomycot	Dothideom	Capnodiale	Mycosphae	Ramularia	0	0	0	0
Ascomycot	Dothideom	Dothideom	Dothideom	Radulidium	0	0	0	0
Ascomycot	Sordariom	Chaetosph	Chaetosph	Dendrophoma	0	0	0	0
Ascomycot	Pezizomyc	Pezizales	Ascodesmi	Cephalioph sp	14	10	5	0
Ascomycot	Dothideomycetes				0	0	29	0
Ascomycot	Dothideom	Capnodiale	Teratosphaeriaceae		0	0	29	0
Ascomycot	Sordariom	Glomerella	Glomerella	Colletotrichum	0	0	0	0
Ascomycot	Sordariom	Diaporthal	Diaporthac	Diaporthe	0	0	0	0
Ascomycot	Dothideom	Capnodiale	Cladospori	Cladosporium	0	0	0	0
Ascomycot	Dothideom	Pleosporal	Didymellac	Neoascochyta	0	0	0	0
Ascomycot	Dothideom	Capnodiale	Mycosphaerellaceae		0	0	0	0
Ascomycot	Dothideom	Pleosporal	Coniothyriaceae		0	0	0	0
Ascomycot	Dothideom	Pleosporal	Didymosph	Pseudopithomyces	0	0	0	0
Ascomycot	Leotiomyc	Helotiales	Dermateac	Pezicula	0	0	0	0
Ascomycot	Sordariom	Glomerella	Glomerella	Colletotricl conoides	0	0	0	0
Ascomycot	Sordariom	Hypocreale	Stachybotr	Paramyrothecium	23	5	0	0
Ascomycot	Dothideom	Pleosporal	Didymellac	Epicoccum	0	28	0	0
Ascomycot	Eurotiomy	Chaetothyi	Trichomeri	Trichomerium	0	26	0	0
Ascomycot	Eurotiomy	Phaeomon	Phaeomon	Neophaeomoniella	0	0	28	0

Ascomycot	Lecanorom	Ostropales	Gomphillac	Corticifraga	0	0	28	0
Ascomycot	Dothideom	Pleosporal	Pleosporac	Curvularia	0	0	0	0
Ascomycot	Dothideom	Capnodiale	Teratosphaeriaceae		0	0	0	0
Ascomycot	Sordariom	Hypocreale	Stachybotr	Paramyrothecium	0	0	0	0
Ascomycot	Sordariom	Diaporthal	Diaporthac	Diaporthe	0	0	0	0
Ascomycot	Dothideom	Pleosporal	Didymospl	Kalmusia	0	0	0	0
Ascomycot	Dothideom	Capnodiale	Capnodiale	Pseudoramichloridium	0	0	0	0
Ascomycot	Eurotiomy	Eurotiales	Aspergillac	Monascus	0	0	0	0
Ascomycot	Eurotiomy	Chaetothyı	Chaetothyı	Strelitziana	27	0	0	0
Ascomycot	Dothideom	Capnodiales			27	0	0	0
Ascomycot	Sordariom	Magnapor	Magnapor	Arxiella	13	0	14	0
Ascomycot	Eurotiomy	Chaetothyı	Chaetothyı	Hermetothecium	10	0	15	0
Ascomycot	Dothideom	Myriangiales			0	27	0	0
Ascomycota					0	27	0	0
Ascomycot	Dothideom	Capnodiale	Teratosphа	Parapenidiella	0	0	0	0
Ascomycot	Dothideom	Capnodiale	Mycosphaerellaceae		0	0	0	0
Ascomycot	Dothideom	Capnodiale	Teratosphа	Penidiella	0	0	0	0
Ascomycot	Dothideom	Pleosporales			0	0	0	0
Ascomycot	Dothideom	Capnodiales			0	0	0	0
Ascomycot	Dothideom	Pleosporal	Didymospl	Pseudopithomyces	0	0	0	0
Ascomycot	Dothideom	Capnodiale	Mycosphaerellaceae		0	0	0	0
Ascomycot	Dothideom	Tubeufiale	Tubeufiaceae		0	0	0	0
Ascomycot	Eurotiomy	Chaetothyı	Trichomeri	Brycekendrickomyces	0	0	0	0
Ascomycot	Pezizomyc	Pezizales	Ascodesmi	Cephaliophora	26	0	0	0
Ascomycot	Sordariom	Hypocreale	Nectriacea	Fusicolla	26	0	0	0
Ascomycot	Archaeorhi	Archaeorhi	Archaeorhi	Archaeorhizomyces	9	17	0	0
Ascomycot	Eurotiomy	Chaetothyı	Trichomeri	Trichomerium	0	0	26	0
Ascomycot	Eurotiomy	Chaetothyı	Trichomeri	Trichomerium	0	0	26	0
Ascomycot	Dothideom	Pleosporal	Cucurbitari	Pyrenochaeta	0	0	0	0
Ascomycot	Sordariom	Diaporthal	Diaporthac	Diaporthe	0	0	0	0
Ascomycot	Dothideom	Capnodiale	Mycosphaerellaceae		0	0	0	0
Ascomycot	Dothideom	Pleosporal	Didymellac	Stagonosporopsis	0	0	0	0
Ascomycot	Dothideom	Capnodiales			0	0	0	0
Ascomycot	Dothideom	Capnodiale	Mycosphaerellaceae		0	0	0	0
Ascomycot	Sordariom	Hypocreale	Nectriacea	Neocosmospora	0	0	0	0
Ascomycot	Dothideom	Pleosporal	Didymellaceae		0	0	0	0
Ascomycot	Dothideom	Pleosporal	Didymospl	Pseudopithomyces	0	0	0	0
Ascomycot	Dothideom	Asterinales	Morenoina	Morenoina	0	0	0	0
Ascomycot	Dothideom	Capnodiale	Mycosphae	Paramycosphaerella	0	25	0	0
Ascomycot	Dothideom	Pleosporal	Torulaceae	Dendryphion	0	25	0	0
Ascomycot	Dothideom	Capnodiale	Teratosphaeriaceae		0	25	0	0
Ascomycot	Eurotiomy	Chaetothyı	Chaetothyı	Chaetothyrium	0	0	25	0
Ascomycot	Dothideom	Capnodiale	Teratosphaeriaceae		0	0	25	0
Ascomycot	Dothideom	Pleosporal	Phaeospha	Setophoma	0	0	11	0
Ascomycot	Dothideom	Capnodiale	Mycosphae	Phaeophleospora	0	0	0	0
Ascomycot	Sordariom	Sordariales	Chaetomiaceae		0	0	0	0
Ascomycot	Eurotiomy	Eurotiales	Aspergillac	Aspergillus	0	0	0	0
Ascomycot	Dothideom	Pleosporal	Didymellac	Didymella	0	0	0	0
Ascomycot	Sordariomycetes				0	0	0	0
Ascomycot	Dothideom	Capnodiale	Mycosphae	Zasmidium	0	0	0	0

Ascomycot	Dothideom	Capnodiales			0	0	0	0
Ascomycot	Sordariom	Diaporthal	Diaporthac	Diaporthe	0	0	0	0
Ascomycot	Dothideom	Pleosporal	Phaeospha	Setophoma	14	10	0	0
Ascomycot	Eurotiomy	Onygenale	Onygenace	Polytolypa	0	15	9	0
Ascomycot	Sordariom	Hypocreale	Hypocreale	Emericellopsis	0	8	5	0
Ascomycot	Dothideom	Capnodiale	Teratosphaeriaceae		0	0	24	0
Ascomycot	Eurotiomy	Eurotiales	Aspergillac	Aspergillus	0	0	0	0
Ascomycot	Sordariom	Diaporthal	Diaporthac	Diaporthe	0	0	0	0
Ascomycot	Sordariom	Branch06			0	0	0	0
Ascomycot	Saccharom	Saccharomycetales			0	0	0	0
Ascomycot	Dothideom	Capnodiale	Cladospori	Cladosporium	0	0	0	0
Ascomycot	Dothideom	Pleosporal	Lophiotren	Atrocalyx	0	0	0	0
Ascomycot	Dothideom	Capnodiale	Teratospha	Xenopenidiella	0	0	0	0
Ascomycot	Leotiomyc	Thelebolal	Pseudeuro	Pseudogymnoascus	23	0	0	0
Ascomycot	Dothideom	Capnodiale	Mycosphae	Zasmidium	23	0	0	0
Ascomycot	Sordariom	Sordariales	Chaetomia	Retroconis	11	0	12	0
Ascomycot	Eurotiomy	Chaetothyı	Chaetothyı	Hermetothecium	0	0	23	0
Ascomycot	Dothideom	Pleosporal	Didymellac	Allophoma	0	0	10	0
Ascomycot	Dothideom	Pleosporal	Pleosporac	Alternaria	0	0	0	0
Ascomycot	Leotiomyc	Thelebolal	Pseudeuro	Pseudeurotium	0	0	0	0
Ascomycot	Dothideom	Capnodiale	Mycosphae	Ramularia	0	0	0	0
Ascomycot	Eurotiomy	Eurotiales	Aspergillac	Penicillium	0	0	0	0
Ascomycot	Dothideom	Capnodiale	Teratospha	Devriesia	0	0	0	0
Ascomycota					0	0	0	0
Ascomycot	Eurotiomy	Onygenale	Onygenale	Zeloasperisporium	0	0	0	0
Ascomycot	Eurotiomy	Eurotiales	Aspergillac	Penicillium	0	0	0	0
Ascomycota					0	0	0	0
Ascomycot	Dothideom	Venturiales	Sympovent	Neocoleroa	22	0	0	0
Ascomycot	Dothideom	Pleosporal	Thyridariac	Roussoella	0	17	5	0
Ascomycot	Dothideom	Capnodiale	Mycosphae	Pseudozasmidium	0	0	0	22
Ascomycot	Dothideom	Capnodiale	Teratosphaeriaceae		0	0	0	0
Ascomycot	Sordariom	Diaporthal	Diaporthac	Diaporthe	0	0	0	0
Ascomycot	Dothideom	Pleosporal	Pleosporac	Alternaria	0	0	0	0
Ascomycot	Dothideom	Capnodiale	Teratospha	Parateratosphaeria	0	0	0	0
Ascomycot	Eurotiomy	Eurotiales	Trichocom	Talaromyces	0	0	0	0
Ascomycot	Dothideom	Pleosporal	Pleosporac	Curvularia buchloes	0	0	0	0
Ascomycot	Eurotiomy	Phaeomon	Phaeomon	Neophaeomoniella	0	0	0	0
Ascomycot	Sordariom	Glomerella	Glomerellaceae		0	0	0	0
Ascomycot	Leotiomyc	Helotiales	Hyaloscypl	Crucellisporiopsis	21	0	0	0
Ascomycot	Sordariomycetes				8	0	13	0
Ascomycot	Sordariom	Xylariales	Amphisphaeriaceae		4	17	0	0
Ascomycota					0	21	0	0
Ascomycot	Sordariomycetes				0	12	0	0
Ascomycot	Dothideom	Venturiales	Sympovent	Neocoleroa	0	0	21	0
Ascomycot	Dothideom	Capnodiale	Mycosphaerellaceae		0	0	21	0
Ascomycot	Lecanoromycetes				0	0	0	0
Ascomycot	Dothideom	Pleosporal	Teichospor	Teichospora	0	0	0	0
Ascomycot	Dothideom	Capnodiale	Mycosphae	Septoria	0	0	0	0
Ascomycot	Sordariom	Diaporthales			0	0	0	0
Ascomycot	Dothideom	Capnodiale	Mycosphae	Phaeophleospora	0	0	0	0

Ascomycot	Sordariomy	Diaporthal	Diaporthac	Diaporthe	0	0	0	0
Ascomycot	Sordariomy	Diaporthal	Diaporthac	Diaporthe	0	0	0	0
Ascomycot	Dothideom	Pleosporal	Pleosporac	Alternaria	0	0	0	0
Ascomycot	Dothideom	Capnodiale	Teratospha	Catenulostroma	0	0	0	0
Ascomycot	Dothideom	Capnodiale	Mycospha	Zasmidium	0	0	0	0
Ascomycot	Dothideom	Pleosporal	Didymellaceae		0	0	0	0
Ascomycot	Dothideom	Capnodiale	Teratospha	Devriesia	0	0	0	0
Ascomycot	Sordariomy	Sordariales	Lasiosphaeriaceae		0	0	0	0
Ascomycota					0	0	0	0
Ascomycot	Sordariomy	Togniniales	Togniniace	Phaeoacremonium	0	0	0	0
Ascomycot	Sordariomy	Diaporthal	Diaporthac	Diaporthe	20	0	0	0
Ascomycot	Dothideom	Pleosporal	Phaeospha	Ophiosphaerella	14	6	0	0
Ascomycot	Sordariomy	Hypocreale	Clavicipitaceae		8	12	0	0
Ascomycot	Pezizomyc	Pezizales	Pezizaceae	Iodophanus	0	20	0	0
Ascomycot	Sordariomy	Diaporthal	Valsaceae	Cytospora	0	16	4	0
Ascomycot	Dothideomycetes				0	0	20	0
Ascomycot	Sordariomy	Diaporthal	Diaporthac	Diaporthe	0	0	0	0
Ascomycot	Pezizomyc	Pezizales	Pyronemat	Geopora	0	0	0	0
Ascomycota					0	0	0	0
Ascomycot	Eurotiomy	Phaeomon	Phaeomon	Xenocylindrosporium	0	0	0	0
Ascomycot	Sordariomy	Xylariales	Amphispha	Amphisphaeria	0	0	0	0
Ascomycot	Sordariomycetes				0	0	0	0
Ascomycot	Dothideom	Capnodiales			0	0	0	0
Ascomycot	Dothideom	Dothideom	Eremomyc	Arthrographis	0	0	0	0
Ascomycot	Leotiomyc	Helotiales	Hyaloscyph	Crucellisporiopsis	0	0	0	0
Ascomycot	Dothideom	Capnodiale	Teratospha	Xenoteratosphaeria	0	0	0	0
Ascomycot	Dothideom	Capnodiale	Teratosphaeriaceae		19	0	0	0
Ascomycot	Sordariomy	Hypocreale	Cordycipita	Lecanicillium	19	0	0	0
Ascomycot	Dothideom	Capnodiales			19	0	0	0
Ascomycot	Sordariomy	Hypocreale	Nectriacea	Stephanonectria	13	6	0	0
Ascomycot	Sordariomy	Sordariales	Sordariace	Gelasinospora	0	19	0	0
Ascomycot	Dothideom	Capnodiales			0	19	0	0
Ascomycot	Saccharom	Saccharom	Phaffomyc	Wickerhamomyces	0	9	0	0
Ascomycot	Dothideom	Capnodiale	Teratosphaeriaceae		0	0	19	0
Ascomycot	Dothideom	Pleosporal	Didymellac	Boeremia	0	0	0	0
Ascomycot	Dothideom	Pleosporal	Coniothyri	Coniothyrium	0	0	0	0
Ascomycot	Dothideom	Capnodiale	Teratospha	Xenoteratosphaeria	0	0	0	0
Ascomycot	Dothideomycetes				0	0	0	0
Ascomycot	Dothideom	Capnodiales			0	0	0	0
Ascomycot	Dothideom	Capnodiale	Mycosphaerellaceae		0	0	0	0
Ascomycot	Sordariomy	Hypocreale	Hypocreale	Acremonium	0	0	0	0
Ascomycot	Dothideom	Capnodiale	Cladospori	Cladosporium	0	0	0	0
Ascomycot	Dothideom	Pleosporal	Didymellaceae		0	0	0	0
Ascomycot	Dothideom	Pleosporal	Cucurbitari	Pyrenochaetopsis	0	0	0	0
Ascomycot	Dothideom	Pleosporal	Dictyospor	Pseudocoleophoma	0	0	0	0
Ascomycot	Dothideom	Capnodiale	Teratospha	Hyweljonesia	0	0	0	0
Ascomycot	Sordariomy	Sordariales	Chaetomiaceae		18	0	0	0
Ascomycot	Dothideom	Pleosporales			13	0	5	0
Ascomycot	Eurotiomy	Chaetothyr	Chaetothyr	Strelitziana	0	18	0	0
Ascomycot	Dothideom	Capnodiale	Teratosphaeriaceae		0	18	0	0

Ascomycot	Leotiomyc	Helotiales				0	4	0	0
Ascomycot	Dothideom	Capnodiales				0	0	18	0
Ascomycot	Dothideomycetes					0	0	18	0
Ascomycot	Dothideom	Capnodiales				0	0	18	0
Ascomycot	Eurotiomy	Chaetothyı	Chaetothyı	Strelitziana		0	0	18	0
Ascomycot	Dothideom	Capnodiale	Mycosphae	Madagascaromyces		0	0	0	0
Ascomycot	Lecanorom	Ostropales	Graphidace	Leiorreuma		0	0	0	0
Ascomycot	Dothideom	Venturiales	Sympoven	Ochroconis		0	0	0	0
Ascomycot	Sordariom	Diaporthal	Diaporthac	Diaporthe		0	0	0	0
Ascomycot	Dothideom	Capnodiale	Teratospha	Xenoteratosphaeria		0	0	0	0
Ascomycot	Sordariom	Hypocreale	Nectriacea	Ilyonectria		0	0	0	0
Ascomycot	Sordariom	Chaetosph	Chaetosphaeriaceae			0	0	0	0
Ascomycot	Sordariom	Diaporthal	Diaporthac	Diaporthe		0	0	0	0
Ascomycot	Dothideom	Pleosporal	Periconiac	Periconia		0	0	0	0
Ascomycota						0	0	0	0
Ascomycot	Dothideom	Pleosporal	Didymellac	Didymella		0	0	0	0
Ascomycot	Sordariom	Hypocreale	Nectriacea	Neocosmospora		0	0	0	0
Ascomycot	Dothideom	Capnodiale	Mycosphae	Exopassalora		0	0	0	0
Ascomycot	Eurotiomy	Chaetothyriales				0	0	0	0
Ascomycot	Sordariomycetes					0	0	0	0
Ascomycot	Dothideom	Pleosporal	Didymellac	Epicoccum		0	0	0	0
Ascomycota						0	0	0	0
Ascomycot	Eurotiomy	Eurotiales	Trichocom	Talaromyces		0	0	0	0
Ascomycot	Dothideom	Tubeufiale	Tubeufiaceae			0	0	0	0
Ascomycot	Sordariom	Diaporthal	Diaporthac	Diaporthe		0	0	0	0
Ascomycot	Dothideom	Capnodiale	Teratosphaeriaceae			17	0	0	0
Ascomycot	Eurotiomy	Chaetothyı	Chaetothyı	Strelitziana		17	0	0	0
Ascomycot	Eurotiomy	Onygenale	Onygenace	Polytolypa		17	0	0	0
Ascomycot	Dothideom	Pleosporal	Morosphae	Acrocalymma		15	0	0	0
Ascomycot	Sordariom	Hypocreales				9	0	8	0
Ascomycot	Dothideom	Capnodiales				0	17	0	0
Ascomycot	Orbiliomyc	Orbiliales				0	17	0	0
Ascomycot	Dothideom	Capnodiales				0	3	14	0
Ascomycot	Eurotiomy	Chaetothyriales				0	0	17	0
Ascomycot	Dothideomycetes					0	0	17	0
Ascomycot	Dothideom	Capnodiales				0	0	17	0
Ascomycot	Sordariom	Microascal	Microascac	Gamsia		0	0	17	0
Ascomycot	Sordariom	Chaetosphaeriales				0	0	0	17
Ascomycot	Dothideom	Capnodiale	Teratospha	Xenoteratosphaeria		0	0	0	0
Ascomycot	Dothideom	Capnodiales				0	0	0	0
Ascomycot	Sordariom	Hypocreale	Nectriacea	Volutella		0	0	0	0
Ascomycot	Leotiomyc	Helotiales	Helotiacea	Scytalidium		0	0	0	0
Ascomycot	Dothideom	Capnodiale	Neodevries	Neodevriesia		0	0	0	0
Ascomycot	Leotiomyc	Erysiphales	Erysiphace	Microsphaera		0	0	0	0
Ascomycot	Eurotiomy	Eurotiales	Trichocom	Thermomy	lanuginosu	0	0	0	0
Ascomycot	Eurotiomy	Chaetothyı	Herpotrich	Exophiala		0	0	0	0
Ascomycot	Dothideom	Pleosporal	Sporormia	Preussia		0	0	0	0
Ascomycot	Eurotiomy	Onygenale	Gymnoasca	Leucothecium		0	0	0	0
Ascomycot	Sordariom	Sordariales	Chaetomia	Humicola		0	0	0	0
Ascomycot	Sordariom	Diaporthal	Diaporthaceae			0	0	0	0

Ascomycot	Dothideom	Tubeufiale	Tubeufiaceae		0	0	0	0
Ascomycot	Sordariom	Glomerella	Plectospha	Lectera	16	0	0	0
Ascomycot	Dothideom	Capnodiale	Mycosphae	Geastrumia	16	0	0	0
Ascomycota					13	0	0	0
Ascomycot	Sordariom	Myrmecrid	Myrmecrid	Myrmecridium	4	0	0	0
Ascomycot	Sordariom	Diaporthal	Cryphonectriaceae		0	16	0	0
Ascomycot	Sordariom	Xylariales			0	16	0	0
Ascomycot	Dothideom	Pleosporal	Sporormia	Preussia	0	16	0	0
Ascomycot	Sordariom	Xylariales	Xylariaceae	Xylaria	0	7	9	0
Ascomycota					0	0	16	0
Ascomycot	Sordariom	Hypocreale	Nectriacea	Neocosmospora	0	0	0	16
Ascomycot	Dothideom	Dothideale	Aureobasic	Aureobasidium	0	0	0	7
Ascomycot	Sordariom	Diaporthal	Diaporthac	Diaporthe	0	0	0	0
Ascomycot	Sordariom	Diaporthal	Diaporthac	Diaporthe	0	0	0	0
Ascomycot	Sordariom	Diaporthal	Diaporthac	Diaporthe	0	0	0	0
Ascomycot	Dothideom	Capnodiale	Teratosphaeriaceae		0	0	0	0
Ascomycot	Lecanorom	Lecanorale	Ramalinace	Bacidina	0	0	0	0
Ascomycot	Dothideom	Capnodiale	Mycosphae	Pallidocercospora	0	0	0	0
Ascomycot	Dothideom	Pleosporal	Didymospl	Kalmusia	0	0	0	0
Ascomycot	Sordariom	Hypocreale	Cordycipita	Simplicillium	0	0	0	0
Ascomycot	Dothideom	Capnodiale	Mycosphae	Xenosonderhenia	0	0	0	0
Ascomycot	Dothideom	Capnodiale	Teratospha	Lapidomyces	0	0	0	0
Ascomycot	Dothideom	Tubeufiale	Tubeufiaceae		0	0	0	0
Ascomycot	Dothideom	Capnodiale	Mycosphae	Exutisphaerella	0	0	0	0
Ascomycot	Dothideom	Capnodiale	Mycosphaerellaceae		0	0	0	0
Ascomycot	Sordariomycetes				0	0	0	0
Ascomycot	Dothideom	Capnodiale	Teratosphaeriaceae		0	0	0	0
Ascomycot	Sordariom	Diaporthal	Valsaceae	Cytospora	15	0	0	0
Ascomycot	Sordariom	Sordariales	Sordariales	Ramophialophora	15	0	0	0
Ascomycot	Sordariom	Hypocreale	Clavicipitac	Metarhizium	8	7	0	0
Ascomycot	Sordariom	Xylariales	Amphispha	Lepteutypa	5	0	0	0
Ascomycot	Archaeorhizomycetes				0	15	0	0
Ascomycot	Dothideom	Pleosporal	Phaeospha	Neosulcatispora	0	15	0	0
Ascomycot	Dothideom	Capnodiale	Cladospori	Toxicocladosporium	0	15	0	0
Ascomycot	Dothideom	Capnodiale	Mycosphaerellaceae		0	0	15	0
Ascomycot	Dothideom	Dothideales			0	0	15	0
Ascomycot	Sordariom	Xylariales	Sporocada	Pestalotiopsis	0	0	0	15
Ascomycot	Dothideom	Pleosporal	Teichosporaceae		0	0	0	8
Ascomycot	Dothideom	Pleosporal	Pleosporac	Curvularia	0	0	0	0
Ascomycot	Sordariom	Sordariales	Chaetomiaceae		0	0	0	0
Ascomycot	Dothideom	Capnodiale	Mycosphae	Geastrumia	0	0	0	0
Ascomycot	Leotiomyc	Helotiales	Dermateac	Mollisia	0	0	0	0
Ascomycot	Sordariom	Sordariales	Sordariales	Rhodoveronaea	0	0	0	0
Ascomycot	Dothideom	Pleosporal	Pleosporac	Curvularia	0	0	0	0
Ascomycot	Dothideom	Pleosporal	Didymellaceae		0	0	0	0
Ascomycot	Sordariom	Branch06			0	0	0	0
Ascomycot	Sordariom	Sordariales	Lasiosphae	Podospora	0	0	0	0
Ascomycot	Dothideom	Capnodiale	Mycosphaerellaceae		0	0	0	0
Ascomycot	Saccharom	Saccharom	Saccharom	Kazachstania	0	0	0	0
Ascomycot	Dothideom	Capnodiale	Mycosphae	Ramularia	0	0	0	0

Ascomycot	Dothideom	Capnodiale	Teratospha	Xenoteratosphaeria	0	0	0	0
Ascomycot	Dothideom	Capnodiale	Teratosphaeriaceae		0	0	0	0
Ascomycot	Dothideom	Capnodiale	Teratospha	Penidiella	0	0	0	0
Ascomycot	Leotiomyce	Helotiales	Hyaloscyphaceae		14	0	0	0
Ascomycot	Sordariomy	Sordariales			14	0	0	0
Ascomycot	Dothideom	Pleosporales			14	0	0	0
Ascomycot	Dothideom	Pleosporale	Pleosporac	Curvularia	14	0	0	0
Ascomycot	Dothideom	Pleosporale	Teichospor	Teichospora	12	0	2	0
Ascomycot	Dothideom	Capnodiales			11	3	0	0
Ascomycot	Sordariomycetes				6	8	0	0
Ascomycot	Sordariomy	Chaetosphaeriales			6	0	4	4
Ascomycot	Sordariomy	Sordariales	Lasiosphaeriaceae		0	14	0	0
Ascomycot	Pezizomyce	Pezizales	Pyronemat	Scutellinia	0	14	0	0
Ascomycot	Sordariomy	Xylariales	Amphisphaeriaceae		0	7	3	0
Ascomycot	Dothideom	Pleosporale	Massarinac	Helminthosporium	0	0	14	0
Ascomycot	Sordariomy	Diaporthal	Diaporthac	Diaporthe	0	0	14	0
Ascomycot	Dothideom	Capnodiale	Teratosphaeriaceae		0	0	14	0
Ascomycot	Dothideom	Capnodiale	Mycosphae	Clypeosphaerella	0	0	0	0
Ascomycot	Dothideom	Capnodiale	Teratosphaeriaceae		0	0	0	0
Ascomycot	Sordariomy	Xylariales	Amphispha	Amphisphaeria	0	0	0	0
Ascomycot	Sordariomy	Xylariales			0	0	0	0
Ascomycot	Sordariomy	Hypocreale	Stachybotr	Stachybotrys	0	0	0	0
Ascomycot	Dothideom	Capnodiale	Cladospori	Cladosporium	0	0	0	0
Ascomycot	Dothideom	Pleosporale	Phaeospha	Phaeosphaeria	0	0	0	0
Ascomycot	Sordariomycetes				0	0	0	0
Ascomycot	Eurotiomyc	Chaetothyr	Chaetothyr	Strelitziana	0	0	0	0
Ascomycot	Sordariomy	Sordariales	Chaetomia	Acrophialophora	0	0	0	0
Ascomycot	Sordariomy	Sordariales			0	0	0	0
Ascomycot	Sordariomy	Diaporthal	Cryphonec	Aurantiosacculus	0	0	0	0
Ascomycot	Eurotiomyc	Chaetothyr	Herpotrichiellaceae		0	0	0	0
Ascomycot	Eurotiomyc	Eurotiales	Trichocom	Talaromyces	0	0	0	0
Ascomycot	Sordariomycetes				0	0	0	0
Ascomycot	Sordariomy	Sordariales	Lasiosphaeriaceae		0	0	0	0
Ascomycot	Leotiomyce	Thelebolale	Pseudeuro	Pseudeurotium	0	0	0	0
Ascomycot	Dothideom	Capnodiale	Mycosphaerellaceae		0	0	0	0
Ascomycota					0	0	0	0
Ascomycot	Eurotiomyc	Eurotiales	Aspergillac	Penicillium	0	0	0	0
Ascomycot	Dothideom	Pleosporale	Didymosph	Kalmusia	0	0	0	0
Ascomycot	Dothideom	Capnodiale	Cladospori	Cladosporium	0	0	0	0
Ascomycot	Sordariomy	Xylariales	Fasciatispo	Fasciatispora	0	0	0	0
Ascomycot	Dothideom	Pleosporale	Pleosporac	Bipolaris	0	0	0	0
Ascomycot	Sordariomy	Sordariales	Lasiosphaeriaceae		0	0	0	0
Ascomycot	Sordariomy	Diaporthal	Cryphonectriaceae		0	0	0	0
Ascomycot	Sordariomy	Diaporthal	Valsaceae	Cytospora	0	0	0	0
Ascomycot	Dothideom	Capnodiale	Mycosphaerellaceae		0	0	0	0
Ascomycot	Dothideom	Capnodiale	Mycosphae	Madagascaromyces	0	0	0	0
Ascomycot	Eurotiomyc	Onygenale	Onygenale	Zeloasperisporium	0	0	0	0
Ascomycot	Dothideom	Pleosporale	Sporormia	Preussia	13	0	0	0
Ascomycot	Sordariomy	Hypocreale	Cordycipita	Simplicillium	13	0	0	0
Ascomycot	Leotiomyce	Helotiales	Helotiales_	Triposporium	13	0	0	0

Ascomycot	Eurotiomy	Chaetothyı	Trichomeri	Trichomerium	13	0	0	0
Ascomycot	Eurotiomy	Eurotiales	Aspergillac	Penicillium	13	0	0	0
Ascomycota					13	0	0	0
Ascomycot	Dothideomycetes				8	5	0	0
Ascomycot	Dothideom	Pleosporal	Phaeospha	Paraphoma	7	0	6	0
Ascomycot	Sordariom	Xylariales	Amphisphа	Amphisphaeria	0	13	0	0
Ascomycot	Sordariom	Diaporthales			0	13	0	0
Ascomycot	Dothideom	Pleosporal	Didymospł	Paraphaeosphaeria	0	0	13	0
Ascomycot	Eurotiomy	Chaetothyriales			0	0	13	0
Ascomycot	Dothideom	Capnodiale	Mycosphae	Pallidocercospora	0	0	13	0
Ascomycot	Dothideom	Capnodiales			0	0	4	0
Ascomycot	Dothideom	Capnodiale	Teratospha	Xenoteratosphaeria	0	0	0	13
Ascomycot	Dothideom	Capnodiale	Teratosphaeriaceae		0	0	0	0
Ascomycot	Eurotiomy	Chaetothyı	Trichomeri	Knufia	0	0	0	0
Ascomycot	Leotiomyc	Helotiales			0	0	0	0
Ascomycot	Dothideom	Pleosporal	Leptospha	Acicuseptoria	0	0	0	0
Ascomycot	Eurotiomy	Chaetothyı	Chaetothyı	Chaetothyrium	0	0	0	0
Ascomycot	Dothideom	Capnodiales			0	0	0	0
Ascomycot	Sordariom	Magnapor	Magnapor	Arxiella	0	0	0	0
Ascomycot	Eurotiomy	Onygenale	Onygenale	Zeloasperisporium	0	0	0	0
Ascomycot	Archaeorhi	Archaeorhi	Archaeorhi	Archaeorhizomyces	0	0	0	0
Ascomycot	Sordariom	Hypocreale	Nectriacea	Ilyonectria	0	0	0	0
Ascomycot	Sordariom	Hypocreale	Nectriacea	Penicillifer	0	0	0	0
Ascomycot	Dothideom	Pleosporal	Didymosphaeriaceae		0	0	0	0
Ascomycot	Dothideom	Pleosporal	Phaeospha	Phaeosphaeria	0	0	0	0
Ascomycot	Sordariom	Diaporthal	Cryphonec	Mastigosporella	0	0	0	0
Ascomycot	Lecanorom	Ostropales	Gomphillac	Corticifraga	0	0	0	0
Ascomycot	Dothideom	Capnodiale	Mycosphaerellaceae		0	0	0	0
Ascomycot	Eurotiomy	Eurotiales	Aspergillaceae		12	0	0	0
Ascomycot	Sordariom	Hypocreale	Hypocreale	Emericellopsis	12	0	0	0
Ascomycot	Dothideom	Capnodiale	Teratosphaeriaceae		12	0	0	0
Ascomycot	Dothideom	Capnodiale	Cladospori	Toxicocladosporium	12	0	0	0
Ascomycot	Dothideom	Capnodiales			12	0	0	0
Ascomycot	Sordariom	Glomerella	Glomerella	Colletotrichum	6	6	0	0
Ascomycot	Dothideom	Botryosphа	Botryosphа	Diplodia	2	0	0	0
Ascomycot	Archaeorhi	Archaeorhi	Archaeorhi	Archaeorhizomyces	0	12	0	0
Ascomycot	Sordariomycetes				0	12	0	0
Ascomycot	Dothideom	Pleosporal	Tetraplosphaeriaceae		0	12	0	0
Ascomycot	Sordariom	Sordariales	Sordariales	Rhodoveronaea	0	6	0	0
Ascomycot	Dothideom	Capnodiale	Teratosphaeriaceae		0	0	12	0
Ascomycot	Leotiomyc	Erysiphales	Erysiphace	Podosphaera	0	0	7	0
Ascomycot	Dothideom	Pleosporal	Pleosporal	Parapyrenochaeta	0	0	0	12
Ascomycota					0	0	0	0
Ascomycota					0	0	0	0
Ascomycot	Sordariom	Sordariales			0	0	0	0
Ascomycot	Eurotiomy	Eurotiales	Trichocom	Talaromyces	0	0	0	0
Ascomycot	Dothideom	Capnodiale	Schizothyri	Johansonia	0	0	0	0
Ascomycot	Dothideom	Venturiales	Sympoveni	Ochroconis	0	0	0	0
Ascomycot	Sordariom	Diaporthal	Diaporthac	Diaporthe	0	0	0	0
Ascomycot	Archaeorhi	Archaeorhi	Archaeorhi	Archaeorhizomyces	0	0	0	0

Ascomycot	Archaeorhi	Archaeorhi	Archaeorhi	Archaeorhizomyces	0	0	0	0
Ascomycot	Sordariom	Hypocreale	Nectriacea	Volutella	0	0	0	0
Ascomycot	Dothideom	Pleosporal	Phaeospha	Neosetophoma	0	0	0	0
Ascomycot	Sordariom	Hypocreale	Nectriacea	Fusarium	0	0	0	0
Ascomycot	Archaeorhizomycetes				0	0	0	0
Ascomycot	Leotiomyc	Helotiales	Dermateaceae		0	0	0	0
Ascomycot	Sordariom	Sordariales			0	0	0	0
Ascomycot	Eurotiomy	Chaetothyı	Chaetothyı	Strelitziana	0	0	0	0
Ascomycot	Archaeorhi	Archaeorhi	Archaeorhi	Archaeorhizomyces	0	0	0	0
Ascomycot	Sordariom	Conioscypl	Conioscypl	Conioscypha	0	0	0	0
Ascomycot	Dothideom	Capnodiale	Mycospha	Phaeophleospora	11	0	0	0
Ascomycot	Dothideom	Pleosporal	Lophiotremataceae		11	0	0	0
Ascomycot	Sordariom	Sordariales	Lasiosphaeriaceae		0	11	0	0
Ascomycot	Sordariom	Glomerella	Glomerella	Colletotrichum	0	11	0	0
Ascomycot	Eurotiomy	Chaetothyı	Trichomeri	Trichomerium	0	0	11	0
Ascomycot	Dothideomycetes				0	0	3	6
Ascomycot	Sordariom	Hypocreale	Nectriaceae		0	0	0	11
Ascomycot	Eurotiomy	Eurotiales	Aspergillac	Penicillium	0	0	0	0
Ascomycot	Sordariom	Hypocreale	Nectriacea	Thelonectria	0	0	0	0
Ascomycot	Dothideom	Pleosporal	Phaeospha	Phaeosphaeria	0	0	0	0
Ascomycot	Sordariom	Hypocreale	Nectriacea	Neocosmospora	0	0	0	0
Ascomycot	Sordariom	Hypocreale	Nectriacea	Mariannaea	0	0	0	0
Ascomycot	Sordariom	Chaetosph	Chaetosph	Chloridium	0	0	0	0
Ascomycot	Eurotiomy	Eurotiales	Trichocom	Talaromyces	0	0	0	0
Ascomycot	Eurotiomy	Eurotiales	Aspergillac	Penicillium	0	0	0	0
Ascomycot	Sordariom	Diaporthal	Diaporthac	Diaporthe	0	0	0	0
Ascomycot	Dothideomycetes				0	0	0	0
Ascomycot	Sordariom	Sordariales	Lasiosphae	Schizothecium	0	0	0	0
Ascomycot	Dothideom	Capnodiale	Teratosphaeriaceae		0	0	0	0
Ascomycot	Eurotiomy	Chaetothyı	Chaetothyı	Strelitziana	0	0	0	0
Ascomycota					0	0	0	0
Ascomycot	Sordariom	Hypocreale	Nectriacea	Neocosmospora	0	0	0	0
Ascomycota					0	0	0	0
Ascomycot	Dothideom	Capnodiale	Cladospori	Rachicladosporium	0	0	0	0
Ascomycot	Sordariom	Sordariales	Chaetomia	Zopfiella	0	0	0	0
Ascomycot	Eurotiomy	Onygenale	Onygenale	Zeloasperisporium	0	0	0	0
Ascomycot	Dothideom	Pleosporal	Didymellaceae		0	0	0	0
Ascomycot	Sordariomycetes				0	0	0	0
Ascomycot	Sordariom	Glomerella	Plectosphaerellaceae		0	0	0	0
Ascomycot	Dothideom	Capnodiale	Teratosphaeriaceae		0	0	0	0
Ascomycot	Dothideom	Capnodiale	Mycospha	Ramulariopsis	0	0	0	0
Ascomycot	Sordariom	Sordariales	Sordariace	Gelasinospora	0	0	0	0
Ascomycota					0	0	0	0
Ascomycot	Archaeorhi	Archaeorhi	Archaeorhi	Archaeorhizomyces	10	0	0	0
Ascomycot	Archaeorhizomycetes				10	0	0	0
Ascomycot	Sordariom	Hypocreale	Nectriacea	Fusicolla	10	0	0	0
Ascomycot	Saccharom	Saccharom	Dipodasca	Dipodascus	10	0	0	0
Ascomycot	Eurotiomy	Eurotiales	Aspergillac	Penicillium	10	0	0	0
Ascomycot	Dothideom	Pleosporal	Phaeospha	Phaeosphaeria	10	0	0	0
Ascomycot	Pezizomyc	Pezizales	Sarcosomataceae		10	0	0	0

Ascomycot	Sordariom	Xylariales	Xylariaceae		0	10	0	0
Ascomycot	Dothideom	Pleosporal	Pleosporac	Curvularia	0	10	0	0
Ascomycot	Eurotiomy	Eurotiales	Aspergillac	Penicillium	0	10	0	0
Ascomycot	Dothideom	Capnodiales			0	10	0	0
Ascomycot	Sordariom	Hypocreale	Nectriacea	Gibberella	0	10	0	0
Ascomycot	Sordariom	Sordariales	Chaetomia	Retroconis	0	10	0	0
Ascomycot	Dothideom	Capnodiale	Teratosphaeriaceae		0	0	10	0
Ascomycota					0	0	10	0
Ascomycot	Eurotiomy	Chaetothyi	Herpotrichiellaceae		0	0	3	0
Ascomycot	Sordariom	Hypocreale	Nectriacea	Cylindrocarpon	0	0	0	10
Ascomycot	Sordariom	Hypocreale	Nectriacea	Neocosmospora	0	0	0	10
Ascomycot	Sordariom	Diaporthales			0	0	0	0
Ascomycot	Eurotiomy	Eurotiales	Aspergillac	Penicillium	0	0	0	0
Ascomycot	Dothideom	Pleosporal	Parabambu	Multiseptospora	0	0	0	0
Ascomycot	Sordariom	Sordariales			0	0	0	0
Ascomycot	Eurotiomy	Eurotiales	Aspergillac	Penicillium	0	0	0	0
Ascomycot	Dothideom	Pleosporal	Cucurbitari	Pyrenochaetopsis	0	0	0	0
Ascomycot	Saccharom	Saccharom	Phaffomyc	Wickerhamomyces	0	0	0	0
Ascomycot	Sordariom	Glomerella	Plectosphaerellaceae		0	0	0	0
Ascomycot	Dothideom	Capnodiales			0	0	0	0
Ascomycot	Sordariom	Sordariales	Lasiosphae	Cladorrhinum	0	0	0	0
Ascomycot	Saccharom	Saccharom	Debaryom	Meyerozyma	0	0	0	0
Ascomycot	Dothideom	Capnodiale	Mycosphae	Paramycosphaerella	0	0	0	0
Ascomycot	Dothideom	Pleosporal	Periconiac	Periconia	0	0	0	0
Ascomycot	Dothideom	Capnodiale	Mycosphae	Zymoseptoria	0	0	0	0
Ascomycot	Sordariom	Hypocreale	Nectriacea	Gibberella	0	0	0	0
Ascomycot	Dothideom	Pleosporal	Periconiac	Periconia	0	0	0	0
Ascomycot	Eurotiomy	Chaetothyi	Chaetothyi	Strelitziana	0	0	0	0
Ascomycot	Dothideom	Pleosporal	Pleosporac	Curvularia	0	0	0	0
Ascomycot	Dothideom	Hysteriales	Hysteriace	Gloniopsis	0	0	0	0
Ascomycot	Dothideom	Pleosporal	Didymellac	Paraboeremia	0	0	0	0
Ascomycot	Eurotiomy	Phaeomoniellales			0	0	0	0
Ascomycota					0	0	0	0
Ascomycot	Sordariom	Hypocreale	Hypocreac	Trichoderma	0	0	0	0
Ascomycot	Dothideom	Pleosporal	Cucurbitariaceae		0	0	0	0
Ascomycot	Eurotiomy	Chaetothyriales			0	0	0	0
Ascomycot	Eurotiomy	Eurotiales	Aspergillac	Penicillium	0	0	0	0
Ascomycot	Sordariom	Xylariales	Xylariales_	Liberomyces	0	0	0	0
Ascomycot	Dothideom	Pleosporal	Cucurbitari	Pyrenochaeta	0	0	0	0
Ascomycot	Leotiomyc	Helotiales	Vibrisseace	Phialoceph humicola	0	0	0	0
Ascomycot	Dothideom	Dothideale	Aureobasic	Aureobasidium	0	0	0	0
Ascomycot	Eurotiomy	Chaetothyi	Chaetothyi	Chaetothyrium	9	0	0	0
Ascomycot	Pezizomyc	Pezizales	Pyronemat	Aleuria	9	0	0	0
Ascomycot	Sordariom	Hypocreale	Nectriacea	Ophionectria	9	0	0	0
Ascomycot	Sordariom	Sordariales	Lasiosphae	Cercophora	0	9	0	0
Ascomycot	Pezizomyc	Pezizales	Pyronemat	Geopora	0	9	0	0
Ascomycot	Sordariom	Microascal	Microascac	Canariomyces	0	9	0	0
Ascomycot	Dothideomycetes				0	9	0	0
Ascomycot	Dothideom	Pleosporal	Lophiotren	Atrocalyx	0	9	0	0
Ascomycot	Leotiomyc	Helotiales	Helotiacea	Tetracladium	0	0	9	0

Ascomycot	Sordariom	Hypocreale	Bionectriac	Bionectria		0	0	4	0
Ascomycot	Sordariom	Sordariales	Chaetomia	Chaetomium		0	0	0	0
Ascomycot	Dothideom	Pleosporal	Torulaceae	Torula		0	0	0	0
Ascomycot	Eurotiomy	Eurotiales	Aspergillac	Penicillium		0	0	0	0
Ascomycot	Eurotiomy	Eurotiales				0	0	0	0
Ascomycot	Eurotiomy	Eurotiales	Aspergillaceae			0	0	0	0
Ascomycot	Sordariom	Microascal	Microascac	Microascus		0	0	0	0
Ascomycot	Sordariom	Chaetosph	Chaetosphaeriaceae			0	0	0	0
Ascomycot	Dothideom	Pleosporal	Pleosporal	Fusculina		0	0	0	0
Ascomycot	Dothideom	Hysteriales	Hysteriace	Gloniopsis		0	0	0	0
Ascomycot	Sordariom	Hypocreale	Hypocreale	Acremonium		0	0	0	0
Ascomycot	Sordariom	Coniochae	Coniochae	Coniochaeta		0	0	0	0
Ascomycot	Dothideom	Capnodiales				0	0	0	0
Ascomycot	Dothideom	Pleosporales				0	0	0	0
Ascomycot	Leotiomyc	Helotiales	Pezizellace	Porodiplodia		0	0	0	0
Ascomycot	Sordariom	Hypocreale	Nectriacea	Penicillifer		0	0	0	0
Ascomycot	Sordariom	Xylariales	Xylariales_	Hansfordia		0	0	0	0
Ascomycot	Sordariom	Hypocreales				0	0	0	0
Ascomycot	Sordariom	Xylariales				0	0	0	0
Ascomycot	Dothideom	Capnodiale	Mycosphaerellaceae			0	0	0	0
Ascomycot	Sordariom	Hypocreale	Bionectriac	Geosmithia		0	0	0	0
Ascomycot	Sordariom	Hypocreale	Bionectriac	Gliomastix		0	0	0	0
Ascomycot	Sordariom	Hypocreale	Hypocreac	Trichoderma		0	0	0	0
Ascomycot	Dothideom	Pleosporal	Pleosporac	Curvularia		0	0	0	0
Ascomycot	Dothideom	Pleosporal	Didymellaceae			0	0	0	0
Ascomycot	Dothideom	Myriangiales				0	0	0	0
Ascomycot	Dothideom	Tubeufiale	Tubeufiaceae			0	0	0	0
Ascomycot	Sordariom	Xylariales	Amphispha	Amphisphaeria		0	0	0	0
Ascomycot	Sordariom	Xylariales	Xylariaceae	Hypoxylon		0	0	0	0
Ascomycot	Archaeorhi	Archaeorhi	Archaeorhi	Archaeorhizomyces		8	0	0	0
Ascomycot	Sordariom	Hypocreale	Nectriacea	Paracremonium		8	0	0	0
Ascomycot	Dothideom	Capnodiale	Mycosphae	Paramycosphaerella		8	0	0	0
Ascomycot	Sordariom	Xylariales				8	0	0	0
Ascomycot	Pezizomyc	Pezizales	Ascobolace	Ascobolus		8	0	0	0
Ascomycot	Saccharom	Saccharomycetales				8	0	0	0
Ascomycota						8	0	0	0
Ascomycot	Pezizomyc	Pezizales	Ascobolace	Ascobolus		4	4	0	0
Ascomycot	Dothideom	Pleosporal	Sporormia	Preussia		0	8	0	0
Ascomycot	Dothideom	Pleosporal	Phaeospha	Paraphoma		0	8	0	0
Ascomycot	Sordariom	Sordariales	Lasiosphae	Apiosordaria		0	8	0	0
Ascomycot	Pezizomyc	Pezizales	Ascodesmi	Cephaliophora		0	8	0	0
Ascomycot	Dothideom	Pleosporal	Pleosporal	Fusculina		0	8	0	0
Ascomycot	Dothideom	Pleosporal	Didymosph	Paraphaeosphaeria		0	8	0	0
Ascomycot	Dothideom	Pleosporal	Torulaceae	Torula		0	8	0	0
Ascomycot	Dothideom	Pleosporal	Pleosporac	Cochliobolus		0	8	0	0
Ascomycot	Sordariom	Branch06				0	8	0	0
Ascomycot	Dothideom	Pleosporal	Periconiac	Periconia		0	0	8	0
Ascomycot	Dothideom	Capnodiale	Teratospha	Xenoteratosphaeria		0	0	0	0
Ascomycot	Sordariom	Hypocreale	Hypocreac	Trichodern	longibrach	0	0	0	0
Ascomycot	Sordariom	Xylariales	Sporocada	Seiridium		0	0	0	0

Ascomycot	Sordariom	Diaporthal	Diaporthac	Diaporthe		0	0	0	0
Ascomycot	Dothideom	Pleosporal	Periconiac	Periconia		0	0	0	0
Ascomycot	Sordariom	Sordariales				0	0	0	0
Ascomycot	Archaeorhizomycetes					0	0	0	0
Ascomycot	Dothideom	Pleosporal	Cucurbitari	Pyrenochaeta		0	0	0	0
Ascomycot	Sordariom	Sordariales	Chaetomia	Corynascus		0	0	0	0
Ascomycot	Sordariom	Hypocreale	Bionectriac	Hydropisphaera		0	0	0	0
Ascomycot	Dothideom	Asterinales	Parmularia	Parmularia		0	0	0	0
Ascomycot	Sordariom	Coniochae	Coniochae	Coniochaeta		0	0	0	0
Ascomycot	Saccharom	Saccharom	Dipodascaceae			0	0	0	0
Ascomycot	Pezizomyc	Pezizales	Ascobolace	Ascobolus		0	0	0	0
Ascomycot	Dothideom	Myriangiales				0	0	0	0
Ascomycot	Eurotiomy	Eurotiales	Trichocom	Talaromyces		0	0	0	0
Ascomycot	Dothideom	Myriangial	Elsinoacea	Elsinoe		0	0	0	0
Ascomycot	Sordariom	Hypocreale	Nectriacea	Fusicolla		0	0	0	0
Ascomycot	Dothideom	Pleosporal	Sporormia	Westerdykella		0	0	0	0
Ascomycot	Sordariom	Hypocreale	Nectriacea	Paracremo	binnewijze	0	0	0	0
Ascomycot	Dothideom	Pleosporal	Didymellac	Pseudoascochyta		0	0	0	0
Ascomycot	Sordariom	Glomerella	Plectospha	Lectera		0	0	0	0
Ascomycot	Dothideom	Pleosporal	Pleosporac	Bipolaris		0	0	0	0
Ascomycot	Dothideom	Pleosporal	Cucurbitari	Pyrenochaetopsis		0	0	0	0
Ascomycot	Eurotiomy	Chaetothyı	Herpotrich	Veronaea		0	0	0	0
Ascomycot	Dothideom	Pleosporal	Phaeospha	Neosulcatispora		0	0	0	0
Ascomycot	Eurotiomy	Chaetothyriales				7	0	0	0
Ascomycot	Archaeorhi	Archaeorhi	Archaeorhi	Archaeorhizomyces		7	0	0	0
Ascomycot	Dothideom	Venturiales	Sympoven	Neocoleroa		7	0	0	0
Ascomycot	Leotiomycetes					7	0	0	0
Ascomycot	Dothideom	Venturiales	Sympoven	Neocoleroa		7	0	0	0
Ascomycot	Sordariom	Xylariales	Microdoch	Microdochium		7	0	0	0
Ascomycot	Sordariom	Microascales				7	0	0	0
Ascomycot	Eurotiomy	Chaetothyı	Herpotrich	Exophiala		7	0	0	0
Ascomycot	Sordariom	Branch06				7	0	0	0
Ascomycot	Eurotiomy	Eurotiales	Aspergillac	Aspergillus		7	0	0	0
Ascomycot	Sordariom	Coniochae	Coniochae	Coniochaeta		5	0	2	0
Ascomycot	Eurotiomy	Chaetothyriales				4	0	0	0
Ascomycot	Saccharom	Saccharom	Dipodasca	Dipodascus		0	7	0	0
Ascomycot	Saccharom	Saccharom	Saccharom	Candida		0	7	0	0
Ascomycota						0	7	0	0
Ascomycot	Dothideom	Pleosporal	Lophiotren	Atrocalyx		0	7	0	0
Ascomycot	Dothideom	Capnodiales				0	7	0	0
Ascomycot	Dothideom	Capnodiale	Mycospha	Exopassalora		0	2	5	0
Ascomycot	Sordariom	Sordariales	Sordariales	Ramophialophora		0	0	7	0
Ascomycot	Saccharom	Saccharom	Saccharom	Diutina	catenulata	0	0	3	0
Ascomycota						0	0	0	7
Ascomycot	Dothideom	Pleosporal	Didymospł	Paraconiothyrium		0	0	0	7
Ascomycot	Dothideom	Pleosporal	Pleosporales_fam_Incertae_sedis			0	0	0	0
Ascomycot	Dothideom	Acrosperm	Acrosperm	Leptodiscella		0	0	0	0
Ascomycot	Orbiliomyc	Orbiliales				0	0	0	0
Ascomycot	Leotiomyc	Helotiales				0	0	0	0
Ascomycot	Leotiomyc	Helotiales	Helotiacea	Articulospora		0	0	0	0

Ascomycot	Sordariom	Hypocreale	Hypocreale	Acremonium	0	0	0	0
Ascomycota					0	0	0	0
Ascomycot	Saccharom	Saccharom	Saccharom	Candida	0	0	0	0
Ascomycot	Sordariom	Xylariales	Xylariales_	Castanediella	0	0	0	0
Ascomycot	Eurotiomy	Eurotiales	Aspergillac	Penicillium	0	0	0	0
Ascomycot	Sordariom	Hypocreale	Hypocreale	Acremonium	0	0	0	0
Ascomycot	Pezizomyc	Pezizales	Pyronemat	Sphaerosporella	0	0	0	0
Ascomycot	Eurotiomy	Eurotiales	Aspergillac	Aspergillus	0	0	0	0
Ascomycot	Dothideom	Pleosporal	Pleosporac	Curvularia	0	0	0	0
Ascomycot	Dothideom	Pleosporal	Pleosporal	Longipedicellata	0	0	0	0
Ascomycot	Sordariom	Hypocreale	Hypocreac	Trichoderma	0	0	0	0
Ascomycot	Sordariom	Hypocreale	Nectriaceae		0	0	0	0
Ascomycot	Archaeorhizomycetes				0	0	0	0
Ascomycot	Dothideom	Pleosporal	Cucurbitari	Pyrenochaetopsis	0	0	0	0
Ascomycot	Sordariom	Glomerella	Glomerellaceae		0	0	0	0
Ascomycota					0	0	0	0
Ascomycot	Eurotiomy	Eurotiales	Aspergillac	Aspergillus	0	0	0	0
Ascomycot	Dothideom	Capnodiales			0	0	0	0
Ascomycot	Dothideom	Capnodiale	Mycosphaerellaceae		0	0	0	0
Ascomycot	Dothideom	Capnodiale	Teratosphaeriaceae		0	0	0	0
Ascomycot	Leotiomycetes				0	0	0	0
Ascomycota					0	0	0	0
Ascomycot	Eurotiomy	Eurotiales	Aspergillac	Aspergillus japonicus	0	0	0	0
Ascomycot	Pezizomyc	Pezizomyc	Pezizomyc	Kramasamuha	0	0	0	0
Ascomycot	Sordariom	Xylariales	Xylariaceae	Daldinia	0	0	0	0
Ascomycot	Dothideom	Pleosporales			0	0	0	0
Ascomycot	Sordariom	Hypocreale	Nectriacea	Penicillifer	0	0	0	0
Ascomycot	Dothideom	Pleosporal	Didymospl	Xenocamarosporium	0	0	0	0
Ascomycot	Sordariom	Sordariales	Chaetomia	Condenascus	0	0	0	0
Ascomycot	Dothideom	Capnodiale	Mycosphaerellaceae		0	0	0	0
Ascomycot	Sordariom	Xylariales	Xylariales_	Phialemoniopsis	0	0	0	0
Ascomycot	Leotiomyc	Helotiales			0	0	0	0
Ascomycot	Archaeorhizomycetes				6	0	0	0
Ascomycot	Dothideom	Capnodiale	Mycospha	Paramycosphaerella	6	0	0	0
Ascomycot	Pezizomyc	Pezizales	Pyronemat	Pseudombrophila	6	0	0	0
Ascomycot	Sordariom	Hypocreale	Nectriacea	Xenocylindrocladium	6	0	0	0
Ascomycota					6	0	0	0
Ascomycot	Sordariom	Hypocreale	Stachybotr	Striatibotrys	6	0	0	0
Ascomycot	Dothideom	Capnodiale	Mycosphaerellaceae		6	0	0	0
Ascomycot	Dothideom	Pleosporal	Didymospl	Paracamarosporium	6	0	0	0
Ascomycot	Dothideom	Myriangiales			0	6	0	0
Ascomycot	Dothideom	Hysteriales			0	6	0	0
Ascomycot	Sordariom	Xylariales	Amphispha	Lepteutypa	0	6	0	0
Ascomycot	Saccharom	Saccharom	Pichiaceae	Pichia	0	6	0	0
Ascomycot	Dothideom	Capnodiale	Cladospori	Toxicocladosporium	0	6	0	0
Ascomycot	Dothideom	Capnodiale	Mycosphaerellaceae		0	0	6	0
Ascomycot	Dothideom	Pleosporal	Sporormia	Preussia	0	0	6	0
Ascomycot	Dothideom	Capnodiale	Mycosphaerellaceae		0	0	6	0
Ascomycot	Dothideom	Pleosporal	Teichospor	Magnibotryascoma	0	0	6	0
Ascomycot	Dothideom	Capnodiale	Mycospha	Exutisphaerella	0	0	0	6

Ascomycot	Dothideom	Capnodiale	Teratosphaeriaceae		0	0	0	0
Ascomycot	Dothideom	Capnodiale	Teratospha	Acidiella	0	0	0	0
Ascomycot	Sordariom	Microascal	Microascac	Canariomyces	0	0	0	0
Ascomycot	Sordariom	Hypocreale	Nectriacea	Neocosmospora	0	0	0	0
Ascomycot	Dothideom	Pleosporal	Didymellaceae		0	0	0	0
Ascomycot	Sordariom	Glomerella	Plectosphaerellaceae		0	0	0	0
Ascomycot	Dothideom	Pleosporal	Phaeospha	Neosetophoma	0	0	0	0
Ascomycot	Dothideom	Capnodiale	Mycosphaerellaceae		0	0	0	0
Ascomycot	Sordariom	Hypocreales			0	0	0	0
Ascomycot	Dothideom	Pleosporal	Pleosporal	Fusculina	0	0	0	0
Ascomycot	Sordariom	Hypocreale	Nectriacea	Gibberella	0	0	0	0
Ascomycot	Leotiomyc	Helotiales	Helotiacea	Articulospora	0	0	0	0
Ascomycot	Dothideom	Capnodiale	Mycosphae	Ramularia	0	0	0	0
Ascomycot	Eurotiomy	Eurotiales	Aspergillac	Phialomyces	0	0	0	0
Ascomycot	Dothideom	Pleosporales			0	0	0	0
Ascomycot	Dothideom	Capnodiale	Mycosphae	Phaeophleospora	0	0	0	0
Ascomycot	Sordariom	Microascales			0	0	0	0
Ascomycot	Sordariom	Sordariales	Chaetomiaceae		0	0	0	0
Ascomycot	Leotiomyc	Helotiales	Hyaloscypl	Hyaloscypha	0	0	0	0
Ascomycot	Eurotiomycetes				0	0	0	0
Ascomycot	Dothideom	Pleosporal	Didymospl	Paraphaeosphaeria	0	0	0	0
Ascomycot	Dothideom	Capnodiale	Mycosphae	Ramularia	0	0	0	0
Ascomycot	Sordariom	Hypocreale	Cordycipita	Simplicillium	0	0	0	0
Ascomycot	Sordariom	Hypocreale	Hypocreale	Sarocladium	0	0	0	0
Ascomycot	Sordariomycetes				0	0	0	0
Ascomycot	Eurotiomy	Phaeomon	Phaeomoniellaceae		0	0	0	0
Ascomycot	Eurotiomy	Eurotiales	Trichocom	Talaromyces	0	0	0	0
Ascomycot	Sordariom	Xylariales	Apiosporaceae		0	0	0	0
Ascomycot	Dothideom	Capnodiale	Mycosphae	Paramycosphaerella	0	0	0	0
Ascomycot	Sordariom	Sordariales	Helmintho	Spadicoides	0	0	0	0
Ascomycot	Eurotiomy	Chaetothyı	Herpotrich	Exophiala	0	0	0	0
Ascomycot	Sordariom	Hypocreales			0	0	0	0
Ascomycot	Dothideom	Pleosporal	Pleosporac	Stemphylium	0	0	0	0
Ascomycot	Sordariom	Hypocreale	Nectriacea	Gliocephalotrichum	0	0	0	0
Ascomycot	Sordariom	Hypocreale	Nectriacea	Ilyonectria	0	0	0	0
Ascomycot	Archaeorhi	Archaeorhi	Archaeorhi	Archaeorhizomyces	0	0	0	0
Ascomycot	Sordariom	Hypocreale	Bionectriaceae		0	0	0	0
Ascomycot	Sordariom	Sordariales			0	0	0	0
Ascomycot	Dothideom	Capnodiale	Teratosphaeriaceae		0	0	0	0
Ascomycot	Eurotiomy	Chaetothyriales			0	0	0	0
Ascomycot	Sordariom	Hypocreale	Cordycipita	Simplicillium	0	0	0	0
Ascomycot	Sordariom	Diaporthal	Diaporthac	Diaporthe	0	0	0	0
Ascomycot	Saccharom	Saccharom	Debaryom	Hyphopichia	5	0	0	0
Ascomycot	Dothideom	Capnodiales			5	0	0	0
Ascomycot	Sordariom	Xylariales			5	0	0	0
Ascomycot	Dothideom	Pleosporal	Cucurbitari	Neocucurbitaria	5	0	0	0
Ascomycot	Sordariom	Xylariales	Coniocessi	Coniocessia	5	0	0	0
Ascomycot	Dothideomycetes				5	0	0	0
Ascomycot	Sordariom	Hypocreale	Bionectriac	Gliomastix	5	0	0	0
Ascomycot	Sordariom	Hypocreales			2	0	3	0

Ascomycot	Dothideom	Capnodiale	Mycosphaerellaceae		0	5	0	0
Ascomycot	Dothideom	Pleosporales			0	5	0	0
Ascomycot	Sordariom	Branch06			0	5	0	0
Ascomycot	Pezizomyc	Pezizales	Pyronemat	Aleuria	0	5	0	0
Ascomycot	Sordariom	Xylariales	Hypoxylace	Hypomontagnella	0	5	0	0
Ascomycot	Sordariom	Hypocreale	Nectriacea	Bisifusarium	0	5	0	0
Ascomycot	Sordariom	Hypocreale	Hypocreale	Chlamydocillium	0	5	0	0
Ascomycot	Sordariom	Hypocreales			0	5	0	0
Ascomycot	Eurotiomy	Eurotiales	Aspergillac	Penicillium	0	5	0	0
Ascomycot	Leotiomyc	Helotiales	Chaetomel	Pilidium	0	5	0	0
Ascomycot	Eurotiomy	Eurotiales	Aspergillac	Penicillium	0	5	0	0
Ascomycot	Sordariom	Sordariales	Chaetomiaceae		0	5	0	0
Ascomycot	Sordariom	Hypocreale	Cordycipita	Engyodontium	0	5	0	0
Ascomycot	Dothideom	Pleosporal	Didymosphaeriaceae		0	0	5	0
Ascomycot	Eurotiomy	Chaetothyı	Chaetothyı	Strelitziana	0	0	5	0
Ascomycot	Arthoniom	Lichenostig	Phaeococc	Phaeococcomyces	0	0	5	0
Ascomycot	Eurotiomy	Chaetothyriales			0	0	5	0
Ascomycot	Dothideom	Pleosporal	Lophiostor	Pseudolophiostoma	0	0	5	0
Ascomycot	Dothideom	Capnodiale	Teratosphaeriaceae		0	0	5	0
Ascomycot	Dothideom	Botryosphą	Aplosporel	Aplosporella	0	0	5	0
Ascomycot	Leotiomyc	Helotiales			0	0	3	0
Ascomycot	Eurotiomy	Eurotiales	Aspergillac	Penicillium	0	0	0	5
Ascomycot	Sordariom	Hypocreale	Nectriacea	Ilyonectria	0	0	0	5
Ascomycot	Orbiliomyc	Orbiliales	Orbiliaceae	Orbilia	0	0	0	5
Ascomycot	Sordariom	Xylariales	Microdochiaceae		0	0	0	5
Ascomycot	Dothideom	Capnodiale	Teratospha	Hyweljonesia	0	0	0	0
Ascomycot	Dothideom	Pleosporal	Periconiace	Periconia	0	0	0	0
Ascomycot	Leotiomycetes				0	0	0	0
Ascomycot	Sordariom	Xylariales	Xylariaceae	Anthostomella	0	0	0	0
Ascomycot	Sordariom	Diaporthal	Diaporthac	Diaporthe	0	0	0	0
Ascomycot	Dothideom	Pleosporal	Pleosporac	Stemphylium	0	0	0	0
Ascomycot	Dothideom	Capnodiales			0	0	0	0
Ascomycot	Sordariom	Hypocreale	Hypocreac	Trichodern citrinovirid	0	0	0	0
Ascomycot	Dothideom	Pleosporal	Leptospha	Ophiobolus	0	0	0	0
Ascomycot	Sordariom	Microascal	Halosphae	Remispora	0	0	0	0
Ascomycot	Leotiomyc	Helotiales	Helotiales_	Cadophora	0	0	0	0
Ascomycot	Sordariom	Chaetosph	Chaetosphaeriaceae		0	0	0	0
Ascomycot	Dothideom	Pleosporal	Teichospor	Magnibotryascoma	0	0	0	0
Ascomycot	Dothideom	Pleosporal	Didymospł	Paraconiothyrium	0	0	0	0
Ascomycot	Sordariom	Hypocreales			0	0	0	0
Ascomycot	Sordariom	Sordariales			0	0	0	0
Ascomycot	Sordariom	Branch06			0	0	0	0
Ascomycot	Pezizomyc	Pezizales	Ascobolace	Ascobolus	0	0	0	0
Ascomycot	Eurotiomy	Chaetothyı	Chaetothyı	Ceramothyrium	0	0	0	0
Ascomycot	Dothideom	Pleosporal	Phaeospha	Setophoma	0	0	0	0
Ascomycot	Eurotiomy	Onygenale	Onygenale	Zeloasperisporium	0	0	0	0
Ascomycot	Dothideomycetes				0	0	0	0
Ascomycot	Leotiomyc	Helotiales	Dermateac	Mollisia	0	0	0	0
Ascomycot	Eurotiomy	Chaetothyriales			0	0	0	0
Ascomycot	Dothideom	Capnodiale	Mycosphaerellaceae		0	0	0	0

Ascomycot	Sordariom	Xylariales	Sporocada	Pestalotiopsis	0	0	0	0
Ascomycot	Eurotiomy	Eurotiales	Aspergillac	Aspergillus	0	0	0	0
Ascomycot	Dothideom	Pleosporal	Phaeospha	Wojnowiciella	0	0	0	0
Ascomycot	Leotiomyc	Helotiales	Tricladiace	Mycofalcella	0	0	0	0
Ascomycot	Dothideom	Pleosporal	Phaeospha	Leptospora	0	0	0	0
Ascomycot	Sordariom	Xylariales	Xylariaceae	Xylaria	0	0	0	0
Ascomycot	Eurotiomy	Eurotiales	Trichocom	Talaromyces	0	0	0	0
Ascomycot	Dothideom	Pleosporal	Phaeospha	Leptospora	0	0	0	0
Ascomycot	Leotiomyc	Helotiales	Helotiacea	Scytalidium	0	0	0	0
Ascomycot	Sordariom	Hypocreale	Hypocreale	Emericellopsis	0	0	0	0
Ascomycot	Eurotiomy	Chaetothyr	Chaetothyr	Strelitziana	0	0	0	0
Ascomycot	Archaeorhi	Archaeorhi	Archaeorhi	Archaeorhizomyces	0	0	0	0
Ascomycot	Sordariom	Hypocreale	Ophiocord	Ophiocordyceps	0	0	0	0
Ascomycot	Sordariom	Hypocreales			0	0	0	0
Ascomycot	Dothideom	Capnodiale	Mycospha	Phaeophleospora	0	0	0	0
Ascomycot	Sordariom	Sordariales	Chaetomia	Botryotrichum	0	0	0	0
Ascomycot	Dothideom	Capnodiale	Teratosphaeriaceae		0	0	0	0
Ascomycot	Sordariom	Sordariales	Lasiosphae	Schizothecium	0	0	0	0
Ascomycot	Sordariom	Microascal	Microasca	Scopulariopsis	0	0	0	0
Ascomycot	Dothideom	Capnodiale	Mycospha	Neoceratosperma	0	0	0	0
Ascomycota					0	0	0	0
Ascomycot	Dothideom	Capnodiales			4	0	0	0
Ascomycot	Sordariom	Microascal	Microasca	Lophotrichus	4	0	0	0
Ascomycot	Archaeorhizomycetes				4	0	0	0
Ascomycot	Dothideom	Pleosporal	Melanommataceae		4	0	0	0
Ascomycot	Dothideom	Pleosporal	Pleosporac	Alternaria	4	0	0	0
Ascomycot	Sordariom	Diaporthal	Cryphonectriaceae		4	0	0	0
Ascomycot	Dothideom	Pleosporal	Massarina	Stagonospora	4	0	0	0
Ascomycot	Dothideom	Pleosporal	Cucurbitari	Pyrenochaeta	4	0	0	0
Ascomycot	Sordariom	Glomerella	Glomerella	Colletotrichum	4	0	0	0
Ascomycot	Eurotiomy	Eurotiales	Aspergillac	Aspergillus	4	0	0	0
Ascomycot	Dothideom	Dothideale	Dothiorace	Hormonema	4	0	0	0
Ascomycot	Xylonomyc	GS34			4	0	0	0
Ascomycot	Dothideom	Botryosph	Botryosph	Neofusicoccum	0	4	0	0
Ascomycot	Dothideom	Dothideom	Dothideom	Peltaster	0	4	0	0
Ascomycot	Eurotiomy	Chaetothyr	Chaetothyriaceae		0	4	0	0
Ascomycot	Dothideom	Capnodiale	Teratosphaeriaceae		0	4	0	0
Ascomycot	Sordariom	Hypocreale	Hypocreac	Trichoderma	0	4	0	0
Ascomycot	Saccharom	Saccharom	Trichomon	Blastobotrys	0	4	0	0
Ascomycot	Dothideom	Venturiales	Sympoven	Neocoleroa	0	4	0	0
Ascomycot	Lecanorom	Ostropales			0	2	0	0
Ascomycot	Dothideom	Pleosporales			0	0	4	0
Ascomycot	Orbiliomyc	Orbiliales	Orbiliaceae	Orbilia	0	0	4	0
Ascomycot	Dothideom	Dothideale	Dothiorace	Hormonema	0	0	4	0
Ascomycot	Dothideom	Capnodiale	Teratosphaeriaceae		0	0	4	0
Ascomycot	Dothideom	Pleosporal	Pleosporac	Alternaria	0	0	4	0
Ascomycot	Dothideom	Botryosph	Botryosph	Pseudofusicoccum	0	0	4	0
Ascomycot	Dothideom	Pleosporal	Didymosph	Pseudopithomyces	0	0	4	0
Ascomycot	Sordariom	Sordariales			0	0	0	4
Ascomycot	Sordariom	Hypocreale	Nectriacea	Mariannaea	0	0	0	4

Ascomycot	Sordariom	Hypocreale	Nectriacea	Cylindrocladiella	0	0	0	4
Ascomycot	Dothideom	Pleosporal	Didymellac	Neoascochyta	0	0	0	4
Ascomycot	Lecanorom	Ostropales	Gomphillaceae		0	0	0	4
Ascomycot	Dothideom	Capnodiale	Mycosphaerellaceae		0	0	0	4
Ascomycot	Eurotiomy	Onygenale	Onygenace	Aphanoascus	0	0	0	0
Ascomycot	Dothideom	Pleosporal	Cucurbitari	Pyrenochaetopsis	0	0	0	0
Ascomycot	Dothideom	Capnodiales			0	0	0	0
Ascomycot	Sordariom	Xylariales	Apiosporac	Apiospora	0	0	0	0
Ascomycot	Dothideom	Venturiales	Sympovent	Neocoleroa	0	0	0	0
Ascomycot	Eurotiomy	Chaetothyı	Chaetothyı	Strelitziana	0	0	0	0
Ascomycot	Eurotiomy	Chaetothyriales			0	0	0	0
Ascomycot	Sordariomycetes				0	0	0	0
Ascomycot	Eurotiomy	Eurotiales	Aspergillac	Aspergillus	0	0	0	0
Ascomycota					0	0	0	0
Ascomycot	Leotiomyc	Helotiales	Chaetomellaceae		0	0	0	0
Ascomycot	Dothideom	Pleosporal	Morosphaeriaceae		0	0	0	0
Ascomycot	Sordariom	Xylariales	Amphisphaeriaceae		0	0	0	0
Ascomycot	Saccharom	Saccharom	Saccharom	Kazachstania	0	0	0	0
Ascomycot	Sordariom	Hypocreale	Bionectriac	Bionectria	0	0	0	0
Ascomycot	Dothideom	Pleosporal	Leptosphae	Leptosphaeria	0	0	0	0
Ascomycot	Sordariom	Togniniales	Togniniace	Phaeoacremonium	0	0	0	0
Ascomycot	Saccharom	Saccharom	Debaryom	Hyphopichia	0	0	0	0
Ascomycot	Dothideom	Asterinales	Parmularia	Parmularia	0	0	0	0
Ascomycot	Dothideom	Capnodiale	Mycosphae	Geastrumia	0	0	0	0
Ascomycot	Dothideom	Dothideale	Aureobasic	Aureobasidium	0	0	0	0
Ascomycot	Eurotiomy	Chaetothyı	Chaetothyriaceae		0	0	0	0
Ascomycot	Dothideom	Pleosporales			0	0	0	0
Ascomycot	Leotiomyc	Rhytismata	Rhytismataceae		0	0	0	0
Ascomycot	Sordariom	Hypocreale	Hypocreac	Trichoderma	0	0	0	0
Ascomycot	Dothideom	Capnodiale	Teratospha	Penidiella	0	0	0	0
Ascomycot	Archaeorhi	Archaeorhi	Archaeorhi	Archaeorhizomyces	0	0	0	0
Ascomycot	Archaeorhi	Archaeorhi	Archaeorhi	Archaeorhizomyces	0	0	0	0
Ascomycot	Dothideom	Capnodiale	Neodevries	Neodevriesia	0	0	0	0
Ascomycot	Archaeorhi	GS31			0	0	0	0
Ascomycot	Sordariom	Diaporthal	Diaporthac	Diaporthe	0	0	0	0
Ascomycot	Sordariom	Microascal	Microascac	Canariomyces	0	0	0	0
Ascomycota					0	0	0	0
Ascomycot	Dothideom	Capnodiale	Mycosphae	Phaeophleospora	0	0	0	0
Ascomycot	Sordariom	Xylariales	Diatrypace	Peroneutypa	0	0	0	0
Ascomycot	Dothideom	Pleosporal	Pleosporaceae		0	0	0	0
Ascomycot	Sordariomycetes				0	0	0	0
Ascomycot	Sordariomycetes				0	0	0	0
Ascomycot	Sordariomycetes				0	0	0	0
Ascomycot	Dothideom	Pleosporal	Pleosporal	Parapyrenochaeta	0	0	0	0
Ascomycot	Dothideom	Capnodiale	Mycosphae	Madagascaromyces	0	0	0	0
Ascomycot	Sordariom	Hypocreale	Bionectriac	Hydropisphaera	0	0	0	0
Ascomycot	Sordariom	Chaetosphaeriales			0	0	0	0
Ascomycot	Sordariom	Hypocreales			0	0	0	0
Ascomycot	Dothideom	Capnodiale	Mycosphae	Ramularia	0	0	0	0
Ascomycot	Sordariom	Sordariales	Chaetomia	Humicola	0	0	0	0

Ascomycot	Dothideom	Capnodiale	Mycosphae	Ramularia	0	0	0	0
Ascomycot	Dothideom	Capnodiales			0	0	0	0
Ascomycot	Sordariomycetes				0	0	0	0
Ascomycot	Eurotiomy	Phaeomon	Phaeomoniellaceae		0	0	0	0
Ascomycot	Sordariom	Xylariales			0	0	0	0
Ascomycot	Sordariom	Hypocreale	Nectriacea	Fusicolla	0	0	0	0
Ascomycot	Dothideom	Capnodiale	Teratospha	Catenulostroma	0	0	0	0
Ascomycot	Dothideom	Pleosporal	Melanomn	Melanodiplodia	3	0	0	0
Ascomycot	Sordariom	Hypocreales			3	0	0	0
Ascomycot	Sordariom	Xylariales	Xylariaceae	Xylaria	3	0	0	0
Ascomycot	Pezizomyc	Pezizales	Pezizales_f	Trichobolus	3	0	0	0
Ascomycot	Orbiliomyc	Orbiliales	Orbiliaceae		3	0	0	0
Ascomycot	Eurotiomy	Onygenale	Gymnoasc	Gymnoascus	3	0	0	0
Ascomycot	Dothideom	Pleosporales			3	0	0	0
Ascomycot	Eurotiomycetes				3	0	0	0
Ascomycot	Eurotiomy	Chaetothyriales			3	0	0	0
Ascomycot	Leotiomyc	Helotiales			3	0	0	0
Ascomycot	Dothideomycetes				3	0	0	0
Ascomycot	Sordariom	Hypocreale	Ophiocord	Hirsutella	3	0	0	0
Ascomycot	Dothideom	Pleosporal	Phaeospha	Phaeodothis	3	0	0	0
Ascomycot	Sordariom	Sordariales			3	0	0	0
Ascomycot	Sordariom	Xylariales	Xylariaceae		3	0	0	0
Ascomycot	Dothideom	Capnodiale	Teratospha	Pseudoteratosphaeria	3	0	0	0
Ascomycot	Sordariom	Chaetosph	Chaetosphaeriaceae		3	0	0	0
Ascomycot	Sordariom	Xylariales	Xylariaceae	Xylaria	3	0	0	0
Ascomycot	Sordariom	Hypocreales			3	0	0	0
Ascomycot	Dothideom	Pleosporal	Didymellac	Neoascochyta	3	0	0	0
Ascomycot	Saccharom	Saccharom	Saccharom	Candida	3	0	0	0
Ascomycot	Sordariomycetes				3	0	0	0
Ascomycota					0	3	0	0
Ascomycot	Dothideom	Capnodiales			0	3	0	0
Ascomycot	Eurotiomy	Onygenale	Onygenale	Spiromastix	0	3	0	0
Ascomycot	Sordariom	Sordariales	Sordariales	Ramophialophora	0	3	0	0
Ascomycot	Leotiomyc	Helotiales	Hyaloscyphaceae		0	3	0	0
Ascomycot	Sordariomycetes				0	3	0	0
Ascomycot	Dothideom	Capnodiale	Capnodiale	Arthrocatena	0	3	0	0
Ascomycot	Dothideom	Pleosporal	Teichosporaceae		0	3	0	0
Ascomycot	Sordariom	Sordariales	Chaetomia	Stolonocarpus	0	3	0	0
Ascomycot	Dothideom	Pleosporal	Teichospor	Magnibotryascoma	0	3	0	0
Ascomycot	Dothideom	Capnodiale	Teratospha	Penidiella	0	3	0	0
Ascomycot	Dothideom	Capnodiale	Mycosphaerellaceae		0	3	0	0
Ascomycot	Sordariom	Sordariales	Chaetomia	Botryotrichum	0	3	0	0
Ascomycot	Sordariom	Xylariales	Sporocadaceae		0	3	0	0
Ascomycot	Dothideom	Pleosporal	Pleosporac	Curvularia	0	3	0	0
Ascomycot	Pezizomyc	Pezizales	Ascodesmi	Cephaliophora	0	3	0	0
Ascomycot	Sordariom	Sordariales	Chaetomia	Retroconis	0	3	0	0
Ascomycot	Sordariom	Hypocreale	Nectriaceae		0	3	0	0
Ascomycot	Dothideom	Pleosporal	Didymellaceae		0	3	0	0
Ascomycot	Taphrinom	Taphrinale	Taphrinace	Taphrina	0	0	3	0
Ascomycot	Eurotiomy	Chaetothy	Herpotrich	Exophiala	0	0	3	0

Ascomycot	Sordariom	Xylariales			0	0	3	0
Ascomycot	Dothideom	Capnodiales			0	0	3	0
Ascomycot	Sordariom	Chaetosph	Chaetosphaeriaceae		0	0	3	0
Ascomycot	Dothideom	Venturiales	Sympovent	Ochroconis	0	0	3	0
Ascomycot	Dothideom	Pleosporal	Pleosporac	Curvularia	0	0	3	0
Ascomycot	Leotiomyc	Helotiales	Sclerotinia	Sclerotinia	0	0	3	0
Ascomycot	Dothideom	Capnodiale	Mycosphaerellaceae		0	0	3	0
Ascomycot	Arthoniom	Lichenostig	Phaeococc	Phaeococcomyces	0	0	3	0
Ascomycota					0	0	3	0
Ascomycot	Dothideom	Pleosporal	Periconiac	Periconia	0	0	3	0
Ascomycot	Dothideom	Capnodiale	Teratospha	Devriesia	0	0	0	3
Ascomycot	Sordariom	Coniochae	Coniochae	Coniochaeta	0	0	0	3
Ascomycot	Eurotiomy	Eurotiales	Aspergillac	Penicillium	0	0	0	0
Ascomycot	Leotiomyc	Helotiales			0	0	0	0
Ascomycot	Dothideom	Capnodiale	Teratosphaeriaceae		0	0	0	0
Ascomycot	Sordariom	Glomerella	Plectospha	Lectera	0	0	0	0
Ascomycot	Sordariom	Sordariales			0	0	0	0
Ascomycot	Dothideom	Pleosporal	Phaeospha	Neosetophoma	0	0	0	0
Ascomycot	Leotiomyc	Helotiales	Helotiales_	Mirandina	0	0	0	0
Ascomycot	Sordariom	Xylariales	Sporocada	Neopestalotiopsis	0	0	0	0
Ascomycot	Dothideom	Capnodiale	Teratospha	Pseudoteratosphaeria	0	0	0	0
Ascomycot	Sordariom	Diaporthal	Diaporthac	Diaporthe	0	0	0	0
Ascomycot	Eurotiomy	Chaetothyriales			0	0	0	0
Ascomycot	Dothideom	Capnodiales			0	0	0	0
Ascomycot	Laboulbeni	Pyxidiophorales			0	0	0	0
Ascomycota					0	0	0	0
Ascomycot	Archaeorhizomycetes				0	0	0	0
Ascomycot	Dothideom	Capnodiales			0	0	0	0
Ascomycot	Sordariom	Glomerella	Glomerella	Colletotrichum	0	0	0	0
Ascomycot	Sordariomycetes				0	0	0	0
Ascomycot	Dothideom	Capnodiale	Mycosphaerellaceae		0	0	0	0
Ascomycot	Dothideom	Capnodiale	Cladospori	Cladosporium	0	0	0	0
Ascomycot	Dothideom	Asterinales	Parmularia	Parmularia	0	0	0	0
Ascomycot	Sordariom	Hypocreale	Nectriacea	Phialoseptomonium	0	0	0	0
Ascomycot	Dothideom	Capnodiale	Mycosphae	Madagascaromyces	0	0	0	0
Ascomycot	Sordariom	Hypocreales			0	0	0	0
Ascomycot	Leotiomyc	Helotiales	Sclerotinia	Botryotinia	0	0	0	0
Ascomycot	Saccharom	Saccharom	Dipodasca	Dipodascus	0	0	0	0
Ascomycot	Sordariom	Xylariales	Xylariaceae		0	0	0	0
Ascomycot	Dothideom	Capnodiales			0	0	0	0
Ascomycot	Leotiomyc	Helotiales	Helotiales_	Leohumicola	0	0	0	0
Ascomycot	Dothideom	Pleosporales			0	0	0	0
Ascomycot	Dothideom	Capnodiales			0	0	0	0
Ascomycot	Archaeorhi	Archaeorhi	Archaeorhi	Archaeorhizomyces	0	0	0	0
Ascomycot	Sordariomycetes				0	0	0	0
Ascomycot	Saccharom	Saccharom	Saccharom	Hanseniaspora	0	0	0	0
Ascomycot	Dothideom	Pleosporal	Didymospl	Paracamarosporium	0	0	0	0
Ascomycot	Sordariom	Sordariales	Lasiosphae	Apiosordaria	0	0	0	0
Ascomycot	Dothideom	Pleosporal	Didymosphaeriaceae		0	0	0	0
Ascomycot	Dothideom	Asterinales	Parmularia	Parmularia	0	0	0	0

Ascomycota						0	0	0	0
Ascomycot	Pezizomyc	Pezizales	Ascobolace	Ascobolus		0	0	0	0
Ascomycot	Sordariom	Hypocreale	Bionectriaceae			0	0	0	0
Ascomycot	Archaeorhi	Archaeorhi	Archaeorhi	Archaeorhizomyces		0	0	0	0
Ascomycot	Archaeorhi	Archaeorhi	Archaeorhi	Archaeorhizomyces		0	0	0	0
Ascomycot	Dothideom	Pleosporal	Phaeospha	Neosulcatispora		0	0	0	0
Ascomycot	Sordariom	Coniochae	Coniochae	Coniochaeta		0	0	0	0
Ascomycot	Sordariom	Xylariales				0	0	0	0
Ascomycot	Sordariom	Xylariales	Xylariacea	Xylaria		0	0	0	0
Ascomycot	Dothideom	Pleosporal	Didymellac	Neodidymella		0	0	0	0
Ascomycot	Sordariom	Diaporthales				0	0	0	0
Ascomycot	Dothideom	Pleosporal	Cucurbitari	Pyrenochaetopsis		0	0	0	0
Ascomycot	Dothideomycetes					0	0	0	0
Ascomycot	Saccharom	Saccharom	Saccharom	Candida		0	0	0	0
Ascomycot	Saccharom	Saccharom	Dipodascaceae			0	0	0	0
Ascomycot	Dothideom	Dothideom	Dothideom	Radulidium		0	0	0	0
Ascomycot	Sordariom	Xylariales	Microdoch	Microdochium		0	0	0	0
Ascomycot	Sordariom	Diaporthales				0	0	0	0
Ascomycot	Dothideom	Capnodiale	Mycosphaerellaceae			0	0	0	0
Ascomycota						0	0	0	0
Ascomycot	Dothideom	Capnodiale	Mycosphaerellaceae			0	0	0	0
Ascomycot	Sordariom	Xylariales	Amphispha	Amphisphaeria		0	0	0	0
Ascomycot	Dothideom	Capnodiales				0	0	0	0
Ascomycot	Sordariom	Xylariales	Xylariaceae			0	0	0	0
Ascomycot	Dothideom	Pleosporal	Didymospł	Paraconiothyrium		0	0	0	0
Ascomycot	Sordariom	Diaporthal	Diaporthac	Diaporthe		0	0	0	0
Ascomycot	Sordariom	Hypocreale	Nectriaceae			0	0	0	0
Ascomycot	Dothideom	Asterinales	Parmularia	Parmularia		0	0	0	0
Ascomycot	Eurotiomy	Eurotiales	Trichocom	Talaromyces		0	0	0	0
Ascomycot	Dothideom	Asterinales	Parmularia	Parmularia		0	0	0	0
Ascomycot	Sordariom	Hypocreale	Nectriacea	Neocosmospora		0	0	0	0
Ascomycot	Dothideom	Capnodiales				0	0	0	0
Ascomycot	Eurotiomy	Chaetothyı	Herpotrichiellaceae			0	0	0	0
Ascomycot	Dothideom	Tubeufiale	Wiesnerior	Spissiomyces		0	0	0	0
Ascomycot	Archaeorhi	Archaeorhi	Archaeorhi	Archaeorhizomyces		0	0	0	0
Ascomycot	Sordariom	Hypocreale	Hypocreac	Monocillium		0	0	0	0
Ascomycot	Saccharom	Saccharom	Phaffomyc	Wickerhamomyces		0	0	0	0
Ascomycot	Sordariom	Hypocreale	Clavicipitaceae			2	0	0	0
Ascomycot	Eurotiomy	Chaetothyı	Herpotrich	Coniosporium		2	0	0	0
Ascomycot	Leotiomyc	Helotiales				2	0	0	0
Ascomycot	Dothideom	Pleosporal	Phaeospha	Leptospora		2	0	0	0
Ascomycot	Lecanorom	Lecanorale	Ramboldia	Ramboldia		2	0	0	0
Ascomycot	Dothideom	Capnodiale	Teratosphaeriaceae			2	0	0	0
Ascomycot	Sordariom	Branch06				2	0	0	0
Ascomycot	Sordariom	Xylariales	Microdoch	Selenodriella		2	0	0	0
Ascomycot	Leotiomyc	Helotiales	Hyaloscypł	Proliferodiscus		2	0	0	0
Ascomycot	Dothideom	Pleosporal	Neomassaı	Neomassaı	thailandica	2	0	0	0
Ascomycot	Dothideom	Capnodiales				2	0	0	0
Ascomycot	Sordariom	Xylariales	Sporocada	Neopestalotiopsis		2	0	0	0
Ascomycot	Dothideom	Capnodiales				2	0	0	0

Ascomycot	Sordariom	Hypocreales			2	0	0	0
Ascomycot	Dothideom	Capnodiale	Mycosphae	Stomiopeltis	2	0	0	0
Ascomycot	Pezizomyc	Pezizales	Pyronemat	Scutellinia	2	0	0	0
Ascomycot	Dothideom	Capnodiale	Mycosphaerellaceae		0	2	0	0
Ascomycot	Dothideom	Myriangiales			0	2	0	0
Ascomycot	Dothideom	Pleosporal	Lophiostor	Flabellascoma	0	2	0	0
Ascomycot	Eurotiomy	Eurotiales	Trichocom	Thermomyces	0	2	0	0
Ascomycot	Eurotiomy	Chaetothyriales			0	2	0	0
Ascomycot	Leotiomyc	Helotiales	Helotiacea	Articulospora	0	2	0	0
Ascomycot	Dothideom	Capnodiale	Teratosphaeriaceae		0	2	0	0
Ascomycot	Dothideom	Capnodiale	Mycosphaerellaceae		0	2	0	0
Ascomycot	Sordariom	Sordariales	Chaetomiaceae		0	2	0	0
Ascomycot	Eurotiomy	Chaetothyriales			0	2	0	0
Ascomycot	Dothideom	Asterinales	Parmularia	Parmularia	0	2	0	0
Ascomycot	Pezizomyc	Pezizales	Ascodesmi	Cephaliophora	0	2	0	0
Ascomycot	Sordariom	Hypocreale	Nectriacea	Volutella	0	2	0	0
Ascomycota					0	2	0	0
Ascomycot	Sordariom	Sordariales			0	2	0	0
Ascomycot	Eurotiomy	Onygenale	Gymnoasc	Leucothecium	0	2	0	0
Ascomycot	Sordariom	Diaporthales			0	2	0	0
Ascomycot	Sordariom	Xylariales	Sporocadaceae		0	2	0	0
Ascomycot	Sordariom	Sordariales	Chaetomia	Collariella	0	2	0	0
Ascomycot	Dothideom	Pleosporal	Sporormia	Preussia	0	2	0	0
Ascomycot	Dothideom	Capnodiale	Mycosphaerellaceae		0	2	0	0
Ascomycot	Dothideom	Pleosporal	Teichospor	Magnibotryascoma	0	0	2	0
Ascomycot	Dothideomycetes				0	0	2	0
Ascomycot	Sordariom	Sordariales	Sordariace	Gelasinospora	0	0	2	0
Ascomycot	Sordariom	Magnapor	Magnapor	Magnaporthe	0	0	2	0
Ascomycot	Eurotiomy	Onygenale	Ajellomycetaceae		0	0	2	0
Ascomycot	Sordariom	Glomerella	Plectospha	Paragibellulopsis	0	0	2	0
Ascomycot	Lecanorom	Caliciales	Caliciaceae		0	0	2	0
Ascomycot	Dothideom	Capnodiales			0	0	2	0
Ascomycota					0	0	2	0
Ascomycot	Dothideom	Capnodiale	Teratosphaeriaceae		0	0	2	0
Ascomycot	Dothideom	Hysteriales	Hysteriace	Rhytidhysteron	0	0	2	0
Ascomycot	Dothideom	Capnodiale	Mycosphae	Ramularia	0	0	2	0
Ascomycot	Dothideom	Capnodiale	Teratospha	Devriesia	0	0	2	0
Ascomycot	Orbiliomyc	Orbiliales	Orbiliacea	Arthrobotrys	0	0	2	0
Ascomycot	Dothideomycetes				0	0	2	0
Ascomycot	Dothideom	Capnodiale	Teratosphaeriaceae		0	0	2	0
Ascomycot	Dothideom	Myriangial	Elsinoaceae		0	0	2	0
Ascomycot	Dothideom	Pleosporal	Didymospl	Montagnula	0	0	2	0
Ascomycot	Sordariom	Hypocreale	Cordycipita	Lecanicillium	0	0	2	0
Ascomycot	Dothideom	Capnodiale	Teratosphaeriaceae		0	0	2	0
Ascomycot	Sordariom	Hypocreale	Hypocreac	Cladobotryum	0	0	0	2
Ascomycot	Saccharom	Saccharom	Pichiaceae	Pichia	0	0	0	2
Ascomycot	Dothideom	Myriangiales			0	0	0	2
Ascomycot	Sordariom	Chaetosph	Chaetosphaeriaceae		0	0	0	2
Ascomycot	Dothideom	Capnodiale	Mycosphae	Exopassalora	0	0	0	2
Ascomycot	Sordariom	Hypocreale	Clavicipita	Metarhizium	0	0	0	2

Ascomycot	Dothideom	Capnodiale	Schizothyri	Johansonia		0	0	0	0
Ascomycot	Sordariom	Hypocreale	Bionectriac	Valsonectria		0	0	0	0
Ascomycot	Dothideom	Capnodiale	Mycosphae	Phaeophleospora		0	0	0	0
Ascomycot	Sordariom	Diaporthales				0	0	0	0
Ascomycot	Eurotiomy	Chaetothyi	Herpotrich	Exophiala	equina	0	0	0	0
Ascomycot	Sordariomycetes					0	0	0	0
Ascomycot	Dothideomycetes					0	0	0	0
Ascomycot	Sordariom	Xylariales	Xylariaceae			0	0	0	0
Ascomycot	Dothideomycetes					0	0	0	0
Ascomycot	Sordariom	Diaporthal	Diaporthac	Diaporthe		0	0	0	0
Ascomycot	Dothideom	Capnodiale	Teratosphaeriaceae			0	0	0	0
Ascomycot	Dothideom	Pleosporal	Didymellaceae			0	0	0	0
Ascomycot	Sordariom	Xylariales	Sporocada	Pestalotiopsis		0	0	0	0
Ascomycot	Dothideom	Capnodiale	Mycosphae	Phaeophleospora		0	0	0	0
Ascomycot	Eurotiomy	Chaetothyriales				0	0	0	0
Ascomycot	Archaeorhizomycetes					0	0	0	0
Ascomycot	Saccharom	Saccharom	Dipodasca	Dipodascus		0	0	0	0
Ascomycot	Dothideom	Pleosporal	Amorosiac	Angustimassarina		0	0	0	0
Ascomycot	Leotiomyc	Thelebolal	Pseudeuro	Gymnostellatospora		0	0	0	0
Ascomycot	Dothideomycetes					0	0	0	0
Ascomycot	Dothideom	Capnodiale	Mycosphaerellaceae			0	0	0	0
Ascomycot	Sordariom	Xylariales	Xylariaceae	Xylaria		0	0	0	0
Ascomycot	Sordariom	Hypocreales				0	0	0	0
Ascomycot	Dothideom	Pleosporal	Didymospl	Spegazzinia		0	0	0	0
Ascomycot	Sordariomycetes					0	0	0	0
Ascomycot	Sordariom	Xylariales				0	0	0	0
Ascomycot	Sordariom	Diaporthales				0	0	0	0
Ascomycot	Leotiomycetes					0	0	0	0
Ascomycot	Sordariom	Sordariales				0	0	0	0
Ascomycot	Eurotiomy	Eurotiales	Trichocom	Talaromyces		0	0	0	0
Ascomycot	Leotiomyc	Helotiales	Helotiacea	Articulospora		0	0	0	0
Ascomycot	Eurotiomycetes					0	0	0	0
Ascomycot	Eurotiomy	Eurotiales	Aspergillac	Penicillium		0	0	0	0
Ascomycot	Sordariom	Diaporthales				0	0	0	0
Ascomycot	Dothideom	Capnodiale	Cladospori	Verrucocladosporium		0	0	0	0
Ascomycot	Leotiomyc	Helotiales				0	0	0	0
Ascomycot	Sordariom	Hypocreale	Hypocreaceae			0	0	0	0
Ascomycot	Sordariom	Boliniales	Boliniales_fam_Incertae_sedis			0	0	0	0
Ascomycot	Sordariom	Xylariales				0	0	0	0
Ascomycot	Sordariom	Xylariales	Xylariaceae			0	0	0	0
Ascomycot	Sordariom	Calosphaei	Calosphaei	Jattaea		0	0	0	0
Ascomycot	Sordariom	Hypocreales				0	0	0	0
Ascomycot	Eurotiomy	Chaetothyi	Cyphellopł	Cyphellopł	fusarioides	0	0	0	0
Ascomycot	Eurotiomy	Eurotiales	Aspergillac	Penicillium		0	0	0	0
Ascomycot	Saccharom	Saccharom	Trichomon	Blastobotrys		0	0	0	0
Ascomycot	Dothideom	Asterinales	Parmularia	Parmularia		0	0	0	0
Ascomycot	Sordariom	Xylariales	Xylariaceae	Anthostomella		0	0	0	0
Ascomycot	Dothideom	Pleosporal	Phaeospha	Leptospora		0	0	0	0
Ascomycot	Dothideom	Pleosporales				0	0	0	0
Ascomycot	Saccharom	Saccharom	Pichiaceae	Pichia		0	0	0	0

Ascomycot	Sordariom	Xylariales	Xylariaceae	Muscodor		0	0	0	0
Ascomycot	Sordariom	Hypocreale	Nectriacea	Neocosmospora		0	0	0	0
Ascomycot	Sordariom	Hypocreale	Nectriacea	Ilyonectria		0	0	0	0
Ascomycot	Dothideom	Capnodiale	Mycosphae	Phaeophleospora		0	0	0	0
Ascomycot	Sordariom	Glomerella	Plectospha	Paragibellulopsis		0	0	0	0
Ascomycota						0	0	0	0
Ascomycot	Dothideom	Capnodiale	Mycosphaerellaceae			0	0	0	0
Ascomycot	Sordariom	Xylariales	Amphispha	Amphisphaeria		0	0	0	0
Ascomycot	Sordariom	Diaporthales				0	0	0	0
Ascomycot	Dothideom	Capnodiale	Mycosphaerellaceae			0	0	0	0
Ascomycot	Dothideom	Botryospha	Phyllosticta	Phyllosticta		0	0	0	0
Ascomycot	Saccharom	Saccharom	Debaryom	Debaryomyces		0	0	0	0
Ascomycot	Dothideom	Capnodiale	Teratosphaeriaceae			0	0	0	0
Ascomycot	Sordariom	Diaporthales				0	0	0	0
Ascomycot	Dothideom	Capnodiale	Teratospha	Devriesia		0	0	0	0
Ascomycot	Sordariom	Hypocreale	Bionectriaceae			0	0	0	0
Ascomycot	Eurotiomy	Onygenale	Onygenace	Auxarthron		0	0	0	0
Ascomycot	Dothideom	Asterinales	Parmularia	Parmularia		0	0	0	0
Ascomycota						0	0	0	0
Ascomycot	Eurotiomy	Chaetothyr	Cyphelloph	Cyphellophora		0	0	0	0
Ascomycot	Eurotiomy	Chaetothyriales				0	0	0	0
Ascomycot	Dothideom	Pleosporal	Corynespo	Corynespora		0	0	0	0
Ascomycot	Sordariomycetes					0	0	0	0
Ascomycot	Leotiomyc	Helotiales	Dermateac	Pezicula		0	0	0	0
Ascomycot	Sordariom	Hypocreale	Stachybotryaceae			0	0	0	0
Ascomycot	Dothideom	Venturiales				0	0	0	0
Ascomycot	Eurotiomy	Phaeomon	Phaeomon	Xenocylindrosporium		0	0	0	0
Ascomycot	Sordariom	Magnapor	Magnaporthaceae			0	0	0	0
Ascomycot	Dothideom	Botryospha	Botryospha	Diplodia		0	0	0	0
Ascomycota						0	0	0	0
Ascomycot	Sordariom	Hypocreale	Nectriacea	Phialoseptomonium		0	0	0	0
Ascomycot	Dothideom	Pleosporal	Didymosphaeriaceae			0	0	0	0
Ascomycot	Dothideom	Pleosporal	Didymellac	Similiphoma		0	0	0	0
Ascomycot	Dothideom	Pleosporal	Didymosphaeriaceae			0	0	0	0
Basidiomy	Tremellom	Tremellale	Bulleribasi	Vishniacozyma		26	32	6	38
Basidiomy	Tremellom	Tremellale	Bulleracea	Fonsecazyma		0	195	96	31
Basidiomy	Tremellom	Tremellales				0	0	0	0
Basidiomy	Tremellom	Tremellale	Rhynchoga	Papiliotrema		0	0	0	11
Basidiomycota						0	0	11	11
Basidiomy	Malassezic	Malassezia	Malassezia	Malassezia		0	0	0	0
Basidiomy	Tremellom	Tremellale	Bulleribasi	Vishniacozyma		0	0	0	0
Basidiomy	Tremellom	Tremellale	Rhynchoga	Papiliotren	frias	0	0	0	9
Basidiomy	Tremellom	Cystofiloba	Mrakiacea	Tausonia		277	244	158	0
Basidiomy	Microbotry	Microbotry	Chrysozym	Sampaiozyma		50	0	0	0
Basidiomy	Tremellom	Tremellale	Tremellale	Kwoniella		0	0	0	0
Basidiomy	Agaricomy	Thelephora	Thelephoraceae			32	36	6	0
Basidiomy	Agaricomy	Russulales	Russulacea	Russula	pectinatoic	0	14	0	0
Basidiomy	Tremellom	Tremellale	Bulleribasi	Hannaella		0	0	0	2
Basidiomy	Tremellom	Tremellale	Bulleribasi	Vishniacozyma		0	0	0	0
Basidiomy	Tremellom	Tremellale	Trimorphomycetaceae			0	0	0	0

Basidiomyc	Agaricomy	Cantharella	Ceratobasi	Waitea	0	0	0	0
Basidiomyc	Tremellom	Tremellale	Trimorpho	Saitozyma	166	38	26	16
Basidiomyc	Tremellom	Tremellale	Trimorpho	Saitozyma	0	0	0	11
Basidiomyc	Wallemion	Wallemiale	Wallemiac	Wallemia	47	0	35	14
Basidiomyc	Ustilaginor	Ustilaginal	Ustilaginac	Moesziomyces	0	0	0	0
Basidiomyc	Malassezio	Malassezia	Malassezia	Malassezia	0	0	0	0
Basidiomyc	Microbotry	Sporidiobo	Sporidiobo	Rhodotorula	0	0	0	0
Basidiomyc	Tremellom	Filobasidia	Piskurozym	Solicoccozyma	99	112	63	0
Basidiomyc	Tremellom	Filobasidia	Filobasidia	Filobasidium	49	22	18	3
Basidiomyc	Tremellom	Filobasidia	Filobasidia	Filobasidium	0	0	0	5
Basidiomyc	Malassezio	Malassezia	Malassezia	Malassezia	0	0	0	0
Basidiomyc	Agaricomy	Cantharella	Ceratobasi	Waitea	0	0	0	0
Basidiomyc	Agaricomy	Agaricales	Tricholoma	Clitocybula	0	0	0	0
Basidiomyc	Tremellom	Tremellale	Rhynchoga	Papiliotrema	0	0	0	0
Basidiomyc	Tremellom	Tremellale	Bulleribasi	Vishniacozyma	0	0	0	0
Basidiomyc	Agaricomy	Corticiales	Punctularia	Punctulariopsis	0	154	0	0
Basidiomyc	Tremellom	Tremellale	Bulleribasi	Vishniacozyma	0	0	0	0
Basidiomyc	Tremellom	Tremellale	Bulleribasi	Hannaella	0	0	0	0
Basidiomyc	Agaricomy	Cantharella	Ceratobasi	Waitea	0	0	0	0
Basidiomyc	Tremellom	Tremellale	Bulleribasi	Hannaella	19	3	0	0
Basidiomyc	Agaricomy	Polyporale	Polyporace	Funalia	0	0	0	0
Basidiomyc	Agaricomy	Auricularia	Exidiaceae		17	0	0	0
Basidiomyc	Tremellom	Tremellale	Bulleribasi	Hannaella	0	0	0	0
Basidiomyc	Tremellom	Tremellale	Bulleribasi	Vishniacozyma	0	0	0	0
Basidiomyc	Tremellom	Tremellale	Rhynchoga	Papiliotrema	0	0	0	0
Basidiomyc	Tremellom	Tremellale	Bulleribasi	Vishniacozyma	0	0	0	0
Basidiomyc	Tremellom	Filobasidia	Filobasidia	Filobasidium	0	0	0	0
Basidiomyc	Malassezio	Malassezia	Malassezia	Malassezia	0	0	0	0
Basidiomyc	Tremellom	Tremellale	Bulleribasi	Vishniacozyma	0	0	0	0
Basidiomyc	Agaricomy	Polyporale	Polyporace	Favolus	0	0	0	0
Basidiomyc	Tremellom	Tremellale	Bulleribasi	Hannaella	0	0	0	0
Basidiomyc	Agaricomy	Polyporales			0	0	0	0
Basidiomyc	Agaricomycetes				0	66	0	0
Basidiomyc	Microbotry	Sporidiobo	Sporidiobo	Rhodotorula	8	40	0	0
Basidiomyc	Tremellom	Tremellale	Tremellale	Kwoniella	0	0	0	0
Basidiomyc	Tremellom	Tremellales			30	0	27	0
Basidiomyc	Tremellom	Holterman	Holterman	Holtermanniella	0	0	0	0
Basidiomyc	Tremellom	Tremellale	Bulleribasi	Vishniacoz sp	0	0	0	0
Basidiomyc	Malassezio	Malassezia	Malassezia	Malassezia	0	0	0	0
Basidiomyc	Tremellom	Tremellale	Tremellace	Tremella	0	0	0	0
Basidiomyc	Tremellom	Tremellale	Trimorpho	Saitozyma	0	0	0	0
Basidiomyc	Pucciniomy	Septobasid	Septobasid	Auriculoscypha	41	0	0	0
Basidiomyc	Tremellom	Tremellale	Bulleribasi	Vishniacozyma	0	0	0	0
Basidiomyc	Tremellom	Tremellale	Bulleribasi	Hannaella	0	0	0	0
Basidiomyc	Tremellom	Tremellale	Bulleribasi	Vishniacozyma	0	0	0	0
Basidiomyc	Tremellom	Tremellale	Bulleribasi	Vishniacozyma	0	0	0	0
Basidiomyc	Agaricomy	Polyporale	Meruliacea	Phlebia	0	0	0	44
Basidiomyc	Tremellom	Tremellale	Tremellale	Kwoniella	0	0	0	0
Basidiomyc	Pucciniomy	Septobasid	Septobasid	Septobasidium	26	0	15	0
Basidiomyc	Agaricomy	Thelephora	Thelephoraceae		0	0	0	0

Basidiomy(	Malassezic	Malassezia	Malasseziaceae		0	0	0	0
Basidiomy(	Tremellom	Tremellale:	Bulleribasi(	Vishniacozyma	0	0	0	0
Basidiomy(	Tremellom	Tremellale:	Bulleribasi(	Vishniacozyma	0	0	0	0
Basidiomy(	Tremellom	Tremellale:	Bulleribasi(	Hannaella	0	0	0	0
Basidiomy(	Microbotry	Sporidiobo	Sporidiobo	Sporobolomyces	0	0	0	0
Basidiomy(	Tremellom	Tremellale:	Bulleribasi(	Vishniacozyma	0	0	0	0
Basidiomy(	Agaricomy	Russulales	Russulacea	Russula	0	0	0	0
Basidiomy(	Tremellom	Tremellale:	Bulleribasi(	Vishniacozyma	0	0	0	0
Basidiomy(	Agaricomy	Trechispor	Hydnodont	Trechispora	0	0	0	0
Basidiomy(	Tremellom	Tremellale:	Bulleribasi(	Vishniacozyma	0	0	0	0
Basidiomy(	Tremellom	Tremellale:	Bulleribasi(	Hannaella	0	0	0	0
Basidiomy(	Tremellom	Tremellale:	Tremellace	Cryptococcus	0	0	0	0
Basidiomy(	Tremellom	Tremellale:	Bulleribasi(	Vishniacozyma	0	0	0	0
Basidiomy(	Malassezic	Malassezia	Malassezia	Malassezia	0	0	0	0
Basidiomy(	Tremellom	Tremellale:	Rhynchoga	Papiliotrema	0	0	0	0
Basidiomy(	Tremellom	Tremellale:	Tremellace	Bulleromyces	0	0	3	0
Basidiomy(	Tremellom	Tremellale:	Tremellale:	Kwoniella	0	0	0	0
Basidiomy(	Cystobasid	Erythrobas	Erythrobas	Erythrobasidium	0	11	19	0
Basidiomy(	Malassezic	Malassezia	Malassezia	Malassezia	0	0	0	0
Basidiomy(	Tremellom	Tremellale:	Bulleribasi(	Vishniacozyma	0	0	0	0
Basidiomy(	Tremellom	Cystofiloba	Cystofiloba	Cystofilobasidium	0	0	0	0
Basidiomy(	Agaricomy	Sebacinale	Sebacinace	Sebacina	24	5	0	0
Basidiomy(	Pucciniomy	Septobasid	Septobasid	Septobasidium	0	0	29	0
Basidiomy(	Malassezic	Malassezia	Malassezia	Malassezia	0	0	0	0
Basidiomy(	Tremellom	Tremellale:	Bulleribasi(	Vishniacozyma	0	0	0	0
Basidiomy(	Agaricomy	Polyporale	Meruliacea	Mycoacia	0	0	0	0
Basidiomy(	Microbotry	Microbotry	Chrysozym	Sampaiozyma	0	0	0	2
Basidiomy(	Agaricomy	Auriculariales			0	0	0	0
Basidiomy(	Tremellom	Tremellale:	Tremellale:	Kwoniella	0	0	0	0
Basidiomy(	Agaricomy	Agaricales	Agaricacea	Lepiota	0	27	0	0
Basidiomy(	Malassezic	Malassezia	Malassezia	Malassezia	0	0	0	0
Basidiomy(	Tremellom	Tremellale:	Bulleribasi(	Vishniacozyma	0	0	0	0
Basidiomy(	Tremellom	Tremellale:	Bulleribasi(	Vishniacozyma	0	0	0	0
Basidiomy(	Tremellom	Tremellale:	Bulleribasi(	Vishniacozyma	0	0	0	0
Basidiomy(	Tremellom	Tremellale:	Bulleribasi(	Vishniacozyma	0	0	0	0
Basidiomy(	Microbotry	Sporidiobo	Sporidiobo	Sporobolomyces	0	0	0	0
Basidiomy(	Agaricomy	Russulales	Peniophora	Peniophora	25	0	0	0
Basidiomy(	Tremellom	Tremellale:	Rhynchoga	Papiliotrema	3	8	0	0
Basidiomy(	Tremellom	Tremellale:	Tremellace	Bulleromyces	0	3	0	0
Basidiomy(	Agaricomy	Agaricales	Psathyrella	Coprinopsis	24	0	0	0
Basidiomy(	Tremellom	Tremellale:	Tremellace	Cryptococcus	16	8	0	0
Basidiomy(	Pucciniomy	Septobasid	Septobasid	Septobasidium	0	0	24	0
Basidiomy(	Malassezic	Malassezia	Malassezia	Malassezia	0	0	0	0
Basidiomy(	Cystobasid	Cystobasid	Symmetros	Symmetrospora	0	0	0	0
Basidiomy(	Tremellom	Tremellale:	Bulleribasi(	Vishniacozyma	0	0	0	0
Basidiomy(	Agaricomy	Cantharella	Ceratobasi	Ceratobasidium	0	0	0	0
Basidiomy(	Tremellom	Tremellale:	Bulleribasi(	Vishniacozyma	0	0	0	0
Basidiomy(	Microbotry	Sporidiobo	Sporidiobo	Sporobolomyces	0	0	0	0
Basidiomy(	Tremellom	Tremellale:	Bulleribasi(	Hannaella	0	0	0	0
Basidiomy(	Agaricomy	Boletales	Gyroporac	Gyroporus	22	0	0	0

Basidiomy	Tremellom	Tremellale	Trimorpho	Saitozyma		0	22	0	0
Basidiomy	Microbotry	Sporidiobo	Sporidiobo	Sporobolomyces		0	22	0	0
Basidiomy	Classiculon	Classiculal	Classiculac	Classicula		0	0	21	0
Basidiomy	Tremellom	Tremellale	Cuniculitre	Kockovaella		0	0	0	0
Basidiomy	Agaricomy	Agaricales	Schizophyl	Schizophyllum		20	0	0	0
Basidiomy	Tremellom	Tremellale	Bulleribasi	Vishniacozyma		0	0	0	0
Basidiomy	Agaricomy	Russulales	Russulacea	Russula		0	0	0	0
Basidiomy	Tremellom	Tremellales				19	0	0	0
Basidiomy	Agaricomy	Agaricales	Typhulacea	Typhula		15	4	0	0
Basidiomy	Agaricomy	Russulales	Peniophoraceae			0	19	0	0
Basidiomy	Tremellom	Tremellale	Bulleribasi	Dioszegia		0	0	0	0
Basidiomy	Agaricomy	Thelephora	Thelephoraceae			0	0	0	0
Basidiomy	Agaricomy	Agaricales	Psathyrella	Coprinellus		0	0	0	0
Basidiomy	Agaricomy	Cantharella	Ceratobasi	Thanatephorus		0	0	0	0
Basidiomy	Agaricomy	Boletales	Gyroporac	Gyroporus		0	0	0	0
Basidiomy	Tremellom	Trichospor	Trichospor	Apiotrichum		17	0	0	0
Basidiomy	Microbotry	Microbotry	Chrysozym	Sampaiozyma		0	17	0	0
Basidiomy	Agaricomycetes					0	0	0	0
Basidiomy	Agaricomy	Boletales	Gyroporac	Gyroporus		0	0	0	0
Basidiomy	Tremellom	Tremellale	Trimorpho	Saitozyma		0	0	0	0
Basidiomy	Tremellom	Tremellale	Bulleribasi	Vishniacozyma		0	0	0	0
Basidiomy	Tremellom	Tremellale	Bulleribasi	Vishniacozyma		0	0	0	0
Basidiomy	Microbotry	Sporidiobo	Sporidiobo	Rhodotorula		0	0	0	0
Basidiomy	Tremellomycetes					0	0	0	0
Basidiomy	Agaricomy	Polyporale	Steccherin	Nigroporus		0	0	0	0
Basidiomy	Tremellom	Tremellale	Tremellace	Bulleromyces		0	0	0	0
Basidiomy	Agaricomy	Cantharella	Ceratobasi	Waitea		0	0	0	0
Basidiomy	Agaricomy	Hymenoch	Hymenoch	Hymenochaete		0	0	0	0
Basidiomy	Tremellom	Tremellale	Cuniculitre	Fellomyces		0	0	0	0
Basidiomy	Agaricomy	Thelephora	Thelephoraceae			0	0	0	0
Basidiomy	Wallemion	Wallemiale	Wallemiac	Wallemia		0	15	0	0
Basidiomy	Tremellom	Tremellale	Bulleribasi	Vishniacozyma		0	0	0	0
Basidiomy	Agaricomy	Polyporale	Meruliacea	Phlebia		0	0	0	0
Basidiomy	Agaricomy	Thelephora	Thelephoraceae			0	0	0	0
Basidiomy	Tremellom	Tremellale	Bulleribasi	Vishniacozyma		0	0	0	0
Basidiomy	Agaricomy	Polyporale	Polyporace	Trametes		13	0	0	0
Basidiomy	Tremellom	Tremellale	Trimorpho	Saitozyma		2	0	0	0
Basidiomy	Agaricomy	Corticiales	Punctularia	Punctulariopsis		0	0	6	0
Basidiomy	Exobasidio	Golubevial	Golubeviac	Golubevia		0	0	0	0
Basidiomy	Agaricomy	Agaricales	Schizophyl	Schizophyllum		0	0	0	0
Basidiomy	Microbotry	Sporidiobo	Sporidiobo	Rhodotorula		0	0	0	0
Basidiomy	Cystobasid	Cystobasid	Symmetros	Symmetrospora		0	0	0	0
Basidiomy	Tremellom	Trichospor	Trichospor	Apiotrichum		0	0	0	0
Basidiomy	Tremellom	Tremellale	Bulleribasi	Dioszegia	sp	0	0	0	0
Basidiomy	Agaricomy	Agaricales	Psathyrella	Coprinellus		0	0	0	0
Basidiomy	Agaricomy	Sebacinale	Serendipita	Serendipita		0	0	0	0
Basidiomy	Agaricomy	Agaricales	Agaricacea	Agaricus		0	0	0	0
Basidiomy	Tremellom	Tremellale	Bulleracea	Fonsecazyma		0	0	0	0
Basidiomy	Agaricomy	Cantharella	Ceratobasi	Ceratobasidium		0	0	0	12
Basidiomy	Cystobasid	Cystobasid	Symmetros	Symmetrospora		0	0	0	0

Basidiomy	Ustilaginor	Ustilaginal	Ustilaginac	Moesziom	antarcticus	0	0	0	0
Basidiomy	Tremellom	Cystofiloba	Cystofiloba	Cystofilobasidium		0	0	0	0
Basidiomy	Agaricomy	Sebacinales				11	0	0	0
Basidiomy	Agaricomy	Agaricales	Inocybaceae			11	0	0	0
Basidiomy	Agaricomy	Agaricales	Entolomat	Clitopilus		0	11	0	0
Basidiomy	Agaricomy	Polyporale	Fomitopsic	Cinereomyces		0	0	0	0
Basidiomy	Agaricomy	Hymenoch	Rickenellac	Sidera		0	0	0	0
Basidiomy	Agaricomy	Polyporale	Phaneroch	Bjerkandera		0	0	0	0
Basidiomy	Tremellom	Filobasidia	Filobasidia	Filobasidium		0	0	0	0
Basidiomy	Tremellom	Tremellales				0	0	0	0
Basidiomy	Agaricomy	Trechisporales				0	0	0	0
Basidiomy	Tremellom	Tremellale	Bulleribasi	Hannaella		0	0	0	0
Basidiomy	Agaricomy	Sebacinale	Sebacinace	Sebacina		0	10	0	0
Basidiomy	Agaricomy	Russulales	Russulacea	Russula		0	0	0	0
Basidiomy	Agaricomy	Agaricales				0	0	0	0
Basidiomy	Cystobasid	Cystobasid	Cystobasid	Occultifur		0	0	0	0
Basidiomy	Exobasidio	Golubevial	Golubeviac	Golubevia		0	0	0	0
Basidiomy	Cystobasid	Erythrobas	Erythrobas	Bannoa		0	0	0	0
Basidiomy	Agaricomy	Polyporale	Meruliacea	Phlebia		0	0	0	0
Basidiomy	Tremellom	Trichospor	Trichospor	Apiotrichum		0	0	0	9
Basidiomy	Tremellom	Tremellale	Bulleribasi	Vishniacozyma		0	0	0	9
Basidiomycota						0	0	0	0
Basidiomy	Agaricomy	Cantharell	Ceratobasidiaceae			0	0	0	0
Basidiomy	Tremellom	Tremellale	Bulleribasi	Vishniacozyma		0	0	0	0
Basidiomy	Malassezic	Malassezia	Malassezia	Malassezia		0	0	0	0
Basidiomy	Agaricomy	Thelephora	Thelephoraceae			0	0	0	0
Basidiomy	Tremellom	Tremellale	Rhynchoga	Papiliotrema		0	0	0	0
Basidiomycota						0	0	0	0
Basidiomy	Cystobasid	Erythrobas	Erythrobas	Bannoa		0	0	8	0
Basidiomy	Agaricomy	Sebacinale	Serendipita	Serendipita		0	0	0	0
Basidiomy	Agaricomy	Sebacinale	Serendipitaceae			0	0	0	0
Basidiomy	Agaricomy	Polyporale	Fomitopsic	Postia		0	0	0	0
Basidiomy	Agaricomy	Agaricales				0	0	0	0
Basidiomy	Cystobasid	Cystobasid	Symmetros	Symmetrospora		0	0	0	0
Basidiomy	Cystobasid	Erythrobas	Erythrobas	Erythrobasidium		0	0	0	0
Basidiomy	Agaricomy	Polyporale	Fomitopsic	Cinereomyces		7	0	0	0
Basidiomy	Agaricomy	Agaricales	Schizophyl	Schizophyllum		0	7	0	0
Basidiomy	Agaricomy	Agaricales	Tricholoma	Gerronema		0	7	0	0
Basidiomy	Pucciniomy	Septobasid	Septobasid	Septobasidium		0	7	0	0
Basidiomy	Agaricomy	Cantharell	Ceratobasi	Thanatephorus		0	0	7	0
Basidiomy	Tremellom	Tremellale	Bulleribasi	Vishniacozyma		0	0	0	0
Basidiomy	Agaricomy	Agaricales	Entolomat	Clitopilus		0	0	0	0
Basidiomy	Agaricomy	Agaricales	Agaricacea	Agaricus		0	0	0	0
Basidiomy	Agaricomy	Agaricales				0	0	0	0
Basidiomy	Tremellom	Tremellale	Bulleribasi	Vishniacozyma		6	0	0	0
Basidiomy	Agaricomycetes					6	0	0	0
Basidiomy	Agaricomy	Agaricales	Agaricacea	Agaricus		6	0	0	0
Basidiomy	Agaricomy	Agaricales				6	0	0	0
Basidiomy	Agaricomycetes					6	0	0	0
Basidiomy	Agaricomy	Agaricales	Agaricacea	Agaricus		0	3	0	0

Basidiomy‹	Agaricomycetes				0	0	6	0
Basidiomy‹	Agaricomy	Agaricales	Bolbitiacea	Conocybe	0	0	6	0
Basidiomy‹	Agaricostill	Agaricostill	Ruineniace	Ruinenia	0	0	6	0
Basidiomy‹	Tremellom	Trichospor	Trichospor	Apiotrichum	0	0	6	0
Basidiomy‹	Agaricomy	Cantharellales			0	0	0	0
Basidiomy‹	Agaricomy	Agaricales	Psathyrella	Coprinellus	0	0	0	0
Basidiomy‹	Agaricomy	Agaricales	Clavariacea	Clavulinopsis	0	0	0	0
Basidiomy‹	Malassezic	Malassezia	Malassezia	Malassezia	0	0	0	0
Basidiomy‹	Malassezic	Malassezia	Malassezia	Malassezia	0	0	0	0
Basidiomy‹	Agaricomy	Agaricales	Psathyrella	Coprinellus	0	0	0	0
Basidiomy‹	Agaricomycetes				0	0	0	0
Basidiomy‹	Agaricomy	Corticiales	Punctularia	Punctulariopsis	0	0	0	0
Basidiomy‹	Agaricomy	Cantharella	Ceratobasi	Ceratobasidium	5	0	0	0
Basidiomy‹	Agaricomy	Polyporale	Steccherin	Nigroporus	5	0	0	0
Basidiomy‹	Agaricostill	Agaricostill	Chionosph	Kurtzmanomyces	5	0	0	0
Basidiomy‹	Pucciniomy	Septobasid	Septobasid	Septobasidium	0	5	0	0
Basidiomy‹	Microbotry	Sporidiobo	Sporidiobo	Rhodotorula	0	0	0	0
Basidiomy‹	Agaricomy	Sebacinale	Sebacinace	Sebacina	0	0	0	0
Basidiomy‹	Agaricomy	Agaricales			0	0	0	0
Basidiomy‹	Agaricomy	Polyporales			0	0	0	0
Basidiomy‹	Agaricomy	Russulales	Russulacea	Russula	0	0	0	0
Basidiomy‹	Microbotry	Sporidiobo	Sporidiobo	Rhodotorula	0	0	0	0
Basidiomy‹	Agaricomy	Russulales	Russulacea	Russula	0	0	0	0
Basidiomy‹	Malassezic	Malassezia	Malassezia	Malassezia	0	0	0	0
Basidiomy‹	Cystobasid	Cystobasid	Microsporomycetaceae		0	0	0	0
Basidiomy‹	Agaricomy	Polyporale	Steccherin	Steccherinum	0	0	0	0
Basidiomy‹	Microbotry	Sporidiobo	Sporidiobo	Rhodosporidiobolus	0	0	0	0
Basidiomy‹	Tremellom	Tremellale	Tremellale	Kwoniella	0	0	0	0
Basidiomy‹	Tremellom	Tremellale	Tremellale	Kwoniella	0	0	0	0
Basidiomy‹	Agaricomycetes				4	0	0	0
Basidiomy‹	Tremellom	Tremellale	Bulleribasi	Hannaella	0	4	0	0
Basidiomy‹	Agaricomy	Corticiales			0	4	0	0
Basidiomy‹	Agaricomy	Auricularia	Exidiaceae		0	4	0	0
Basidiomy‹	Agaricomycetes				0	4	0	0
Basidiomy‹	Agaricomy	Agaricales	Lyophyllac	Fibulochlar chilensis	0	4	0	0
Basidiomy‹	Tremellom	Tremellale	Bulleribasi	Vishniacozyma	0	4	0	0
Basidiomy‹	Agaricomy	Russulales			0	0	4	0
Basidiomy‹	Tremellom	Tremellale	Tremellale	Kwoniella	0	0	0	4
Basidiomy‹	Agaricomy	Polyporale	Podoscyph	Hypochnicium	0	0	0	0
Basidiomy‹	Tremellom	Tremellale	Bulleracea	Fonsecazyma	0	0	0	0
Basidiomy‹	Agaricomycetes				0	0	0	0
Basidiomy‹	Microbotry	Microbotry	Chrysozym	Udeniozyma	0	0	0	0
Basidiomy‹	Tremellom	Tremellale	Bulleribasi	Bulleribasi oberjocher	0	0	0	0
Basidiomy‹	Atractiellor	Atractiellal	Hoehnelon	Atractiella	0	0	0	0
Basidiomy‹	Ustilaginor	Ustilaginal	Ustilaginac	Macalpinomyces	0	0	0	0
Basidiomy‹	Agaricomy	Agaricales	Entolomat	Entoloma	0	0	0	0
Basidiomy‹	Ustilaginor	Ustilaginal	Ustilaginac	Macalpinomyces	0	0	0	0
Basidiomy‹	Tremellom	Tremellales			0	0	0	0
Basidiomy‹	Tremellom	Tremellale	Bulleribasi	Dioszegia	0	0	0	0
Basidiomy‹	Cystobasid	Cystobasid	Symmetros	Symmetrospora	0	0	0	0

Basidiomy	Tremellom	Tremellale	Bulleribasi	Vishniacozyma	0	0	0	0
Basidiomy	Tremellom	Trichospor	Trichospor	Cutaneotrichosporon	0	0	0	0
Basidiomy	Microbotry	Sporidiobo	Sporidiobo	Rhodosporidiobolus	0	0	0	0
Basidiomy	Agaricomy	Thelephora	Thelephoraceae		0	0	0	0
Basidiomy	Agaricomy	Agaricales	Stropharia	Kuehneromyces	0	0	0	0
Basidiomy	Agaricomy	Agaricales	Agaricales_	Uncobasidium	3	0	0	0
Basidiomy	Tremellom	Filobasidia	Piskurozym	Solicoccozyma	3	0	0	0
Basidiomy	Agaricomy	Agaricales	Stephanosporaceae		3	0	0	0
Basidiomy	Agaricomy	Agaricales	Clavariacea	Clavaria	3	0	0	0
Basidiomy	Agaricostil	Agaricostil	Kondoacea	Kondoa	3	0	0	0
Basidiomy	Agaricomy	Cantharella	Ceratobasi	Thanatephorus	3	0	0	0
Basidiomy	Agaricomy	Agaricales	Schizophyl	Schizophyllum	3	0	0	0
Basidiomy	Agaricomy	Polyporale	Fomitopsic	Skeletocutis	3	0	0	0
Basidiomy	Agaricomy	Agaricales	Bolbitiacea	Panaeolus	0	3	0	0
Basidiomy	Agaricomy	Agaricales	Psathyrella	Coprinellus	0	3	0	0
Basidiomy	Agaricomy	Agaricales	Clavariacea	Clavaria	0	3	0	0
Basidiomy	Agaricomy	Agaricales	Tricholoma	Tricholoma	0	0	0	0
Basidiomy	Agaricomycetes				0	0	0	0
Basidiomy	Tremellom	Filobasidia	Filobasidia	Goffeauzyma	0	0	0	0
Basidiomy	Tremellom	Trichospor	Trichospor	Trichosporon	0	0	0	0
Basidiomy	Exobasidio	Entylomata	Entylomata	Tilletiopsis	0	0	0	0
Basidiomy	Ustilaginor	Ustilaginal	Ustilaginac	Moesziomyces	0	0	0	0
Basidiomy	Agaricomy	Agaricales	Cortinariac	Cortinarius	0	0	0	0
Basidiomy	Tremellomycetes				0	0	0	0
Basidiomy	Agaricomy	Cantharella	Clavulinaceae		0	0	0	0
Basidiomy	Agaricomy	Agaricales	Psathyrella	Coprinellus	0	0	0	0
Basidiomy	Agaricomy	Agaricales			0	0	0	0
Basidiomy	Tremellom	Filobasidia	Filobasidia	Naganishia	0	0	0	0
Basidiomy	Agaricomy	Sebacinale	Serendipitaceae		0	0	0	0
Basidiomy	Agaricomy	Agaricales	Entolomata	Clitopilus	0	0	0	0
Basidiomy	Agaricomy	Sebacinales			0	0	0	0
Basidiomy	Tremellom	Tremellales			0	0	0	0
Basidiomy	Agaricomy	Russulales	Russulacea	Russula	2	0	0	0
Basidiomy	Agaricomy	Polyporale	Phaneroch	Phanerochaete	2	0	0	0
Basidiomy	Agaricomy	Trechispor	Hydnodon	Trechispora	2	0	0	0
Basidiomy	Agaricomy	Auriculariales			2	0	0	0
Basidiomycota					0	2	0	0
Basidiomy	Tremellom	Filobasidia	Piskurozym	Solicoccozyma	0	2	0	0
Basidiomy	Agaricomy	Agaricales	Psathyrella	Coprinopsis	0	2	0	0
Basidiomy	Agaricomy	Sebacinale	Serendipita	Serendipita	0	2	0	0
Basidiomy	Agaricomy	Agaricales	Clavariacea	Clavulinopsis	0	0	2	0
Basidiomycota					0	0	2	0
Basidiomy	Agaricomy	Boletales	Gyroporac	Gyroporus	0	0	2	0
Basidiomy	Agaricomy	Trechispor	Hydnodontaceae		0	0	0	0
Basidiomy	Pucciniomy	Septobasid	Septobasid	Septobasidium	0	0	0	0
Basidiomy	Tremellom	Tremellale	Bulleribasi	Vishniacozyma	0	0	0	0
Basidiomy	Malassezic	Malassezia	Malassezia	Malassezia	0	0	0	0
Basidiomy	Agaricomy	Polyporale	Podoscyph	Rickiopora	0	0	0	0
Basidiomy	Agaricomy	Auricularia	Exidiaceae		0	0	0	0
Basidiomy	Agaricomy	Auriculariales			0	0	0	0

Basidiomy(	Exobasidio	Exobasidiales			0	0	0	0
Basidiomy(	Tremellom	Tremellales			0	0	0	0
Basidiomy(	Agaricomy	Russulales	Russulacea	Russula	0	0	0	0
Basidiomy(	Agaricomy	Agaricales	Schizophyl	Schizophyllum	0	0	0	0
Basidiomy(	Tremellom	Cystofiloba	Cystofiloba	Cystofilobasidium	0	0	0	0
Basidiomy(	Agaricomy	Polyporale	Ganoderm	Ganoderma	0	0	0	0
Chytridiom	Rhizophlyc	Rhizophlyc	Rhizophlyctidaceae		24	35	70	0
Chytridiom	Spizellomy	Spizellomycetales			56	35	0	0
Chytridiomycota					3	60	10	0
Chytridiom	Rhizophlyc	Rhizophlyc	Rhizophlyc	Rhizophlyctis	0	0	0	0
Chytridiom	Rhizophydi	Rhizophydi	Alphamyce	Betamyces	0	0	0	0
Chytridiom	Rhizophydi	Rhizophydiales			0	14	0	0
Chytridiom	Rhizophydi	Rhizophydiales			12	0	0	0
Chytridiom	Spizellomy	Spizellomy	Spizellomy	Spizellomyces	0	0	9	0
Chytridiomycota					7	0	0	0
Chytridiom	Rhizophydi	Rhizophydiales			6	0	0	0
Chytridiomycota					0	0	0	0
Chytridiomycota					4	0	0	0
Chytridiomycota					0	3	0	0
Chytridiomycota					0	0	3	0
Chytridiom	Spizellomy	Spizellomy	Powellomycetaceae		0	2	0	0
Chytridiom	Spizellomy	Spizellomy	Powellomycetaceae		0	0	2	0
Chytridiomycota					0	0	0	0
Chytridiomycota					0	0	0	0
Fungi					0	30	0	586
Fungi					0	0	0	29
Fungi					14141	11095	279	0
Fungi					16613	6536	0	0
Fungi					0	18983	281	0
Fungi					0	0	0	109
Fungi					0	0	0	51
Fungi					0	0	0	0
Fungi					5154	2294	3074	0
Fungi					159	69	7260	0
Fungi					0	0	0	19
Fungi					0	0	0	0
Fungi					0	0	0	0
Fungi					0	0	0	0
Fungi					0	0	0	0
Fungi					0	0	0	0
Fungi					1556	724	884	0
Fungi					0	0	3137	0
Fungi					1320	520	701	0
Fungi					1405	504	711	0
Fungi					2614	0	0	0
Fungi					47	29	2501	0
Fungi					401	136	1896	0
Fungi					0	0	0	0
Fungi					0	0	0	0
Fungi					0	0	0	0

Fungi	1142	368	712	0
Fungi	1267	353	524	0
Fungi	0	0	0	0
Fungi	1311	398	210	0
Fungi	0	0	0	167
Fungi	896	620	206	0
Fungi	0	896	0	220
Fungi	37	15	1454	0
Fungi	893	179	331	0
Fungi	1196	236	0	0
Fungi	0	0	0	0
Fungi	810	303	259	0
Fungi	660	345	193	0
Fungi	1151	25	27	0
Fungi	21	15	1127	0
Fungi	1161	0	0	0
Fungi	574	175	387	0
Fungi	517	193	233	0
Fungi	0	0	945	0
Fungi	0	0	0	0
Fungi	184	96	643	0
Fungi	0	0	0	0
Fungi	0	0	0	0
Fungi	0	0	0	9
Fungi	0	0	0	0
Fungi	504	64	166	0
Fungi	0	0	0	0
Fungi	0	0	0	0
Fungi	41	438	118	0
Fungi	0	0	46	4
Fungi	0	0	695	0
Fungi	100	6	12	8
Fungi	0	0	0	0
Fungi	224	173	231	0
Fungi	0	0	0	0
Fungi	0	0	0	0
Fungi	0	0	0	0
Fungi	11	480	10	0
Fungi	346	80	80	0
Fungi	0	0	0	0
Fungi	319	71	112	0
Fungi	0	0	502	0
Fungi	0	409	0	0
Fungi	0	0	0	0
Fungi	0	0	0	0
Fungi	0	0	457	0
Fungi	0	0	0	0
Fungi	0	0	0	419
Fungi	0	0	0	0
Fungi	291	110	0	0

Fungi	0	0	0	399
Fungi	94	13	288	0
Fungi	0	0	0	0
Fungi	44	22	297	0
Fungi	391	0	0	0
Fungi	0	0	0	0
Fungi	294	91	0	0
Fungi	0	0	384	0
Fungi	303	0	73	0
Fungi	0	22	21	0
Fungi	360	0	0	0
Fungi	169	114	77	0
Fungi	0	0	0	0
Fungi	87	73	194	0
Fungi	0	0	0	0
Fungi	0	0	0	0
Fungi	320	0	0	0
Fungi	0	0	0	0
Fungi	229	0	87	0
Fungi	0	0	316	0
Fungi	0	0	0	0
Fungi	0	0	0	0
Fungi	0	0	308	0
Fungi	0	0	0	0
Fungi	0	0	0	0
Fungi	0	289	0	0
Fungi	0	0	0	0
Fungi	0	0	0	0
Fungi	282	0	0	0
Fungi	148	92	41	0
Fungi	180	91	0	0
Fungi	151	119	0	0
Fungi	133	83	47	0
Fungi	0	263	0	0
Fungi	262	0	0	0
Fungi	0	0	0	0
Fungi	0	0	0	0
Fungi	0	0	0	0
Fungi	0	0	0	244
Fungi	76	101	66	0
Fungi	236	0	0	0
Fungi	182	16	36	0
Fungi	0	0	0	0
Fungi	32	79	96	0
Fungi	196	0	22	0
Fungi	0	0	0	0
Fungi	0	0	215	0
Fungi	0	0	0	0
Fungi	196	0	0	0
Fungi	38	73	0	0

Fungi					9	6	176	0
Fungi					162	0	27	0
Fungi					0	0	0	0
Fungi					179	0	0	0
Fungi					0	178	0	0
Fungi					0	0	0	0
Fungi					0	0	174	0
Fungi					172	0	0	0
Fungi					59	91	6	0
Fungi					0	0	0	0
Fungi					0	0	0	0
Fungi					0	166	0	0
Fungi					0	0	0	0
Fungi					159	0	0	0
Fungi					33	0	126	0
Fungi					8	7	137	0
Fungi					0	0	159	0
Fungi					0	0	0	0
Fungi					156	0	0	0
Fungi					0	0	0	0
Fungi					151	0	0	0
Fungi					151	0	0	0
Fungi					150	0	0	0
Fungi					0	150	0	0
Fungi					6	10	6	0
Fungi					19	22	107	0
Fungi					146	0	0	0
Fungi					0	144	0	0
Fungi					0	0	0	143
Fungi					140	0	0	0
Fungi					57	83	0	0
Fungi					136	0	0	0
Fungi					135	0	0	0
Fungi					135	0	0	0
Fungi					134	0	0	0
Fungi					0	0	134	0
Fungi					0	0	0	0
Fungi					0	0	0	0
Fungi					0	0	0	0
Fungi					114	16	0	0
Glomerom	Glomerom	Glomerale:	Glomerace	Funneliformis	0	42	0	0
Glomerom	Glomerom	Glomerale:	Glomerace	Rhizophagus	0	0	0	0
Monoblepl	Monoblepl	Monoblepharidales			0	0	0	0
Mortierellc	Mortierellc	Mortierella	Mortierella	Mortierella	172	129	102	0
Mortierellc	Mortierellc	Mortierella	Mortierella	Mortierella	170	133	88	0
Mortierellc	Mortierellc	Mortierella	Mortierella	Mortierella	112	94	78	0
Mortierellc	Mortierellc	Mortierella	Mortierella	Mortierella	146	70	58	0
Mortierellc	Mortierellc	Mortierella	Mortierella	Mortierella	68	72	47	0
Mortierellc	Mortierellc	Mortierella	Mortierella	Mortierella	74	66	30	0
Mortierellc	Mortierellc	Mortierella	Mortierella	Mortierella	57	48	22	0

Mortierell‹	Mortierell‹	Mortierella	Mortierella	Mortierella		69	0	36	0
Mortierell‹	Mortierell‹	Mortierella	Mortierella	Mortierella		0	0	0	0
Mortierell‹	Mortierell‹	Mortierella	Mortierella	Mortierella	gamsii	31	33	0	0
Mortierell‹	Mortierell‹	Mortierella	Mortierella	Mortierella		21	15	0	0
Mortierell‹	Mortierell‹	Mortierella	Mortierella	Mortierella		0	0	0	0
Mortierell‹	Mortierell‹	Mortierella	Mortierella	Mortierella		24	0	11	0
Mortierell‹	Mortierell‹	Mortierella	Mortierella	Mortierella		0	0	0	25
Mortierell‹	Mortierell‹	Mortierella	Mortierella	Mortierella		0	21	0	0
Mortierell‹	Mortierell‹	Mortierella	Mortierella	Mortierella		16	0	0	0
Mortierell‹	Mortierell‹	Mortierella	Mortierella	Mortierella		0	0	0	0
Mortierell‹	Mortierell‹	Mortierella	Mortierella	Mortierella		0	0	0	0
Mortierellomycota						0	0	0	0
Mortierell‹	Mortierell‹	Mortierellales				0	0	0	0
Mortierell‹	Mortierell‹	Mortierella	Mortierella	Mortierella		0	0	12	0
Mortierell‹	Mortierell‹	Mortierella	Mortierella	Mortierella		0	11	0	0
Mortierell‹	Mortierell‹	Mortierella	Mortierella	Mortierella		10	0	0	0
Mortierell‹	Mortierell‹	Mortierella	Mortierella	Mortierella		0	0	0	0
Mortierell‹	Mortierell‹	Mortierella	Mortierella	Mortierella		0	0	9	0
Mortierell‹	Mortierell‹	Mortierella	Mortierella	Mortierella		6	0	0	0
Mortierell‹	Mortierell‹	Mortierella	Mortierella	Mortierella		0	0	0	6
Mortierell‹	Mortierell‹	Mortierellales				0	0	0	0
Mortierell‹	Mortierell‹	Mortierella	Mortierella	Mortierella		0	0	0	0
Mortierell‹	Mortierell‹	Mortierellales				5	0	0	0
Mortierell‹	Mortierell‹	Mortierella	Mortierella	Mortierella		0	0	0	0
Mortierell‹	Mortierell‹	Mortierella	Mortierellaceae			0	0	0	0
Mortierell‹	Mortierell‹	Mortierella	Mortierella	Mortierella		0	0	0	0
Mortierell‹	Mortierell‹	Mortierella	Mortierella	Mortierella		3	0	0	0
Mortierell‹	Mortierell‹	Mortierella	Mortierella	Mortierella		0	3	0	0
Mortierell‹	Mortierell‹	Mortierella	Mortierella	Mortierella		0	0	0	0
Mortierell‹	Mortierell‹	Mortierella	Mortierella	Mortierella		0	0	0	0
Olpidiomy‹	Olpidiomy‹	Olpidiales	Olpidiacea	Olpidium		20	0	7	0
Olpidiomy‹	Olpidiomy‹	Olpidiales	Olpidiacea	Olpidium		23	4	17	0
Rozellomy‹	Rozellomy‹	Branch03				0	0	0	0
Rozellomycota						0	0	0	0
Rozellomycota						0	0	0	0
Rozellomycota						0	0	0	0
Rozellomycota						0	0	0	0
Rozellomycota						0	0	0	0
Rozellomycota						0	0	0	0
Rozellomycota						0	0	0	0
Rozellomycota						0	5	0	0
Rozellomycota						0	0	0	0
Rozellomy‹	Rozellomy‹	GS11				0	3	0	0
Zoopagomycota						7	0	0	0
Zoopagomycota						4	0	0	0
Zoopagom	Zoopagom	Zoopagales	Piptocepha	Syncephalis		0	0	3	0
Zoopagom	Zoopagom	Zoopagales	Piptocepha	Syncephalis		2	0	0	0
Zoopagomycota						0	2	0	0
Zoopagom	Zoopagom	Zoopagales	Piptocepha	Syncephalis		0	0	2	0
Fungi						0	0	0	0

Fungi	0	121	0	0
Fungi	0	0	121	0
Fungi	0	0	120	0
Fungi	119	0	0	0
Fungi	0	118	0	0
Fungi	0	0	0	0
Fungi	0	0	0	0
Fungi	115	0	0	0
Fungi	115	0	0	0
Fungi	0	0	0	4
Fungi	114	0	0	0
Fungi	0	114	0	0
Fungi	69	44	0	0
Fungi	0	0	0	113
Fungi	112	0	0	0
Fungi	0	0	0	0
Fungi	0	0	0	0
Fungi	108	0	0	0
Fungi	106	0	0	0
Fungi	106	0	0	0
Fungi	0	106	0	0
Fungi	0	0	0	0
Fungi	0	0	104	0
Fungi	0	0	0	0
Fungi	57	0	45	0
Fungi	0	102	0	0
Fungi	0	101	0	0
Fungi	0	100	0	0
Fungi	99	0	0	0
Fungi	0	0	0	99
Fungi	98	0	0	0
Fungi	0	97	0	0
Fungi	0	97	0	0
Fungi	0	97	0	0
Fungi	0	0	0	0
Fungi	0	62	0	0
Fungi	0	0	96	0
Fungi	0	0	10	0
Fungi	95	0	0	0
Fungi	95	0	0	0
Fungi	95	0	0	0
Fungi	0	0	0	0
Fungi	94	0	0	0
Fungi	0	93	0	0
Fungi	0	93	0	0
Fungi	0	0	93	0
Fungi	92	0	0	0
Fungi	0	0	0	0
Fungi	90	0	0	0
Fungi	46	43	0	0

Fungi	0	89	0	0
Fungi	0	89	0	0
Fungi	0	0	3	0
Fungi	0	0	0	0
Fungi	0	88	0	0
Fungi	0	0	0	0
Fungi	0	0	0	0
Fungi	0	86	0	0
Fungi	0	86	0	0
Fungi	0	86	0	0
Fungi	0	86	0	0
Fungi	84	0	0	0
Fungi	84	0	0	0
Fungi	84	0	0	0
Fungi	0	84	0	0
Fungi	0	84	0	0
Fungi	83	0	0	0
Fungi	83	0	0	0
Fungi	49	34	0	0
Fungi	0	83	0	0
Fungi	0	0	83	0
Fungi	0	0	0	0
Fungi	0	82	0	0
Fungi	0	0	82	0
Fungi	81	0	0	0
Fungi	2	0	79	0
Fungi	0	81	0	0
Fungi	80	0	0	0
Fungi	5	0	75	0
Fungi	0	80	0	0
Fungi	0	0	0	0
Fungi	79	0	0	0
Fungi	0	0	0	0
Fungi	0	0	78	0
Fungi	0	0	0	0
Fungi	0	77	0	0
Fungi	0	77	0	0
Fungi	76	0	0	0
Fungi	0	76	0	0
Fungi	0	75	0	0
Fungi	0	75	0	0
Fungi	0	0	0	0
Fungi	74	0	0	0
Fungi	74	0	0	0
Fungi	74	0	0	0
Fungi	51	0	23	0
Fungi	39	13	22	0
Fungi	0	74	0	0
Fungi	0	74	0	0
Fungi	0	0	74	0

Fungi	73	0	0	0
Fungi	0	73	0	0
Fungi	72	0	0	0
Fungi	72	0	0	0
Fungi	22	0	15	0
Fungi	0	0	0	0
Fungi	0	70	0	0
Fungi	0	70	0	0
Fungi	0	0	0	0
Fungi	69	0	0	0
Fungi	69	0	0	0
Fungi	69	0	0	0
Fungi	69	0	0	0
Fungi	50	19	0	0
Fungi	46	0	23	0
Fungi	0	69	0	0
Fungi	0	0	0	0
Fungi	68	0	0	0
Fungi	68	0	0	0
Fungi	68	0	0	0
Fungi	68	0	0	0
Fungi	0	68	0	0
Fungi	0	0	0	0
Fungi	0	0	0	0
Fungi	0	67	0	0
Fungi	0	67	0	0
Fungi	46	20	0	0
Fungi	0	0	66	0
Fungi	65	0	0	0
Fungi	65	0	0	0
Fungi	65	0	0	0
Fungi	65	0	0	0
Fungi	64	0	0	0
Fungi	64	0	0	0
Fungi	64	0	0	0
Fungi	0	64	0	0
Fungi	0	64	0	0
Fungi	0	64	0	0
Fungi	0	64	0	0
Fungi	63	0	0	0
Fungi	63	0	0	0
Fungi	63	0	0	0
Fungi	63	0	0	0
Fungi	0	63	0	0
Fungi	0	0	0	0
Fungi	9	13	40	0
Fungi	0	62	0	0
Fungi	61	0	0	0
Fungi	61	0	0	0
Fungi	0	61	0	0

Fungi	60	0	0	0
Fungi	0	60	0	0
Fungi	0	60	0	0
Fungi	0	60	0	0
Fungi	0	60	0	0
Fungi	0	0	0	0
Fungi	0	0	59	0
Fungi	58	0	0	0
Fungi	58	0	0	0
Fungi	58	0	0	0
Fungi	58	0	0	0
Fungi	58	0	0	0
Fungi	58	0	0	0
Fungi	0	58	0	0
Fungi	0	58	0	0
Fungi	0	58	0	0
Fungi	0	0	0	0
Fungi	57	0	0	0
Fungi	57	0	0	0
Fungi	57	0	0	0
Fungi	57	0	0	0
Fungi	57	0	0	0
Fungi	7	10	6	0
Fungi	0	57	0	0
Fungi	0	57	0	0
Fungi	56	0	0	0
Fungi	56	0	0	0
Fungi	25	31	0	0
Fungi	0	56	0	0
Fungi	0	56	0	0
Fungi	0	0	56	0
Fungi	55	0	0	0
Fungi	49	0	6	0
Fungi	30	25	0	0
Fungi	0	55	0	0
Fungi	0	55	0	0
Fungi	0	0	0	0
Fungi	54	0	0	0
Fungi	54	0	0	0
Fungi	54	0	0	0
Fungi	54	0	0	0
Fungi	0	54	0	0
Fungi	0	54	0	0
Fungi	0	54	0	0
Fungi	0	54	0	0
Fungi	0	54	0	0
Fungi	0	54	0	0
Fungi	0	54	0	0
Fungi	53	0	0	0
Fungi	53	0	0	0

Fungi	53	0	0	0
Fungi	53	0	0	0
Fungi	53	0	0	0
Fungi	0	53	0	0
Fungi	0	53	0	0
Fungi	0	53	0	0
Fungi	0	0	0	53
Fungi	0	0	0	0
Fungi	52	0	0	0
Fungi	52	0	0	0
Fungi	0	52	0	0
Fungi	0	52	0	0
Fungi	0	52	0	0
Fungi	0	9	4	0
Fungi	0	0	0	0
Fungi	0	0	0	0
Fungi	51	0	0	0
Fungi	0	51	0	0
Fungi	0	51	0	0
Fungi	0	51	0	0
Fungi	0	0	51	0
Fungi	0	0	51	0
Fungi	0	0	0	0
Fungi	50	0	0	0
Fungi	50	0	0	0
Fungi	50	0	0	0
Fungi	0	50	0	0
Fungi	0	50	0	0
Fungi	0	0	50	0
Fungi	0	0	50	0
Fungi	0	0	0	50
Fungi	0	0	0	0
Fungi	0	0	0	0
Fungi	49	0	0	0
Fungi	49	0	0	0
Fungi	49	0	0	0
Fungi	49	0	0	0
Fungi	49	0	0	0
Fungi	49	0	0	0
Fungi	49	0	0	0
Fungi	0	49	0	0
Fungi	0	49	0	0
Fungi	0	49	0	0
Fungi	0	0	0	0
Fungi	48	0	0	0
Fungi	48	0	0	0
Fungi	48	0	0	0
Fungi	48	0	0	0
Fungi	48	0	0	0
Fungi	0	48	0	0

Fungi	0	48	0	0
Fungi	0	48	0	0
Fungi	0	48	0	0
Fungi	0	48	0	0
Fungi	0	48	0	0
Fungi	0	48	0	0
Fungi	0	0	0	0
Fungi	47	0	0	0
Fungi	47	0	0	0
Fungi	47	0	0	0
Fungi	47	0	0	0
Fungi	47	0	0	0
Fungi	30	17	0	0
Fungi	0	47	0	0
Fungi	0	47	0	0
Fungi	0	47	0	0
Fungi	0	0	0	0
Fungi	46	0	0	0
Fungi	46	0	0	0
Fungi	46	0	0	0
Fungi	0	46	0	0
Fungi	0	46	0	0
Fungi	0	46	0	0
Fungi	0	0	46	0
Fungi	0	0	0	0
Fungi	45	0	0	0
Fungi	45	0	0	0
Fungi	45	0	0	0
Fungi	45	0	0	0
Fungi	45	0	0	0
Fungi	45	0	0	0
Fungi	45	0	0	0
Fungi	0	45	0	0
Fungi	0	45	0	0
Fungi	0	45	0	0
Fungi	0	45	0	0
Fungi	0	45	0	0
Fungi	0	45	0	0
Fungi	0	41	0	0
Fungi	44	0	0	0
Fungi	44	0	0	0
Fungi	44	0	0	0
Fungi	0	44	0	0
Fungi	0	44	0	0
Fungi	0	44	0	0
Fungi	43	0	0	0
Fungi	43	0	0	0
Fungi	43	0	0	0
Fungi	43	0	0	0
Fungi	43	0	0	0

Fungi	0	43	0	0
Fungi	0	43	0	0
Fungi	0	43	0	0
Fungi	0	43	0	0
Fungi	0	0	0	0
Fungi	0	0	0	0
Fungi	42	0	0	0
Fungi	42	0	0	0
Fungi	0	42	0	0
Fungi	0	42	0	0
Fungi	0	42	0	0
Fungi	0	0	17	0
Fungi	0	0	0	0
Fungi	41	0	0	0
Fungi	41	0	0	0
Fungi	41	0	0	0
Fungi	41	0	0	0
Fungi	41	0	0	0
Fungi	41	0	0	0
Fungi	19	7	15	0
Fungi	0	41	0	0
Fungi	0	41	0	0
Fungi	0	41	0	0
Fungi	0	41	0	0
Fungi	0	0	41	0
Fungi	0	0	41	0
Fungi	0	0	0	0
Fungi	40	0	0	0
Fungi	40	0	0	0
Fungi	40	0	0	0
Fungi	40	0	0	0
Fungi	0	40	0	0
Fungi	0	40	0	0
Fungi	0	40	0	0
Fungi	0	40	0	0
Fungi	0	40	0	0
Fungi	0	40	0	0
Fungi	0	0	40	0
Fungi	0	0	0	0
Fungi	39	0	0	0
Fungi	39	0	0	0
Fungi	39	0	0	0
Fungi	39	0	0	0
Fungi	6	10	0	0
Fungi	0	39	0	0
Fungi	0	39	0	0
Fungi	0	39	0	0
Fungi	0	39	0	0
Fungi	0	39	0	0
Fungi	0	39	0	0

Fungi	0	0	39	0
Fungi	38	0	0	0
Fungi	38	0	0	0
Fungi	38	0	0	0
Fungi	38	0	0	0
Fungi	38	0	0	0
Fungi	38	0	0	0
Fungi	38	0	0	0
Fungi	0	38	0	0
Fungi	0	38	0	0
Fungi	0	38	0	0
Fungi	0	38	0	0
Fungi	0	38	0	0
Fungi	0	38	0	0
Fungi	0	0	0	0
Fungi	0	0	0	0
Fungi	37	0	0	0
Fungi	37	0	0	0
Fungi	37	0	0	0
Fungi	37	0	0	0
Fungi	37	0	0	0
Fungi	37	0	0	0
Fungi	0	37	0	0
Fungi	0	37	0	0
Fungi	0	37	0	0
Fungi	0	0	37	0
Fungi	0	0	0	0
Fungi	0	0	0	0
Fungi	36	0	0	0
Fungi	36	0	0	0
Fungi	36	0	0	0
Fungi	36	0	0	0
Fungi	36	0	0	0
Fungi	36	0	0	0
Fungi	36	0	0	0
Fungi	36	0	0	0
Fungi	27	0	0	0
Fungi	0	36	0	0
Fungi	0	36	0	0
Fungi	0	36	0	0
Fungi	0	36	0	0
Fungi	0	0	36	0
Fungi	0	0	0	0
Fungi	35	0	0	0
Fungi	35	0	0	0
Fungi	35	0	0	0
Fungi	35	0	0	0
Fungi	35	0	0	0
Fungi	35	0	0	0
Fungi	35	0	0	0

Fungi	35	0	0	0
Fungi	35	0	0	0
Fungi	35	0	0	0
Fungi	35	0	0	0
Fungi	35	0	0	0
Fungi	0	35	0	0
Fungi	0	35	0	0
Fungi	0	35	0	0
Fungi	0	35	0	0
Fungi	0	0	0	0
Fungi	0	0	0	0
Fungi	0	0	0	0
Fungi	34	0	0	0
Fungi	34	0	0	0
Fungi	34	0	0	0
Fungi	34	0	0	0
Fungi	34	0	0	0
Fungi	34	0	0	0
Fungi	34	0	0	0
Fungi	34	0	0	0
Fungi	34	0	0	0
Fungi	8	0	0	0
Fungi	6	0	0	0
Fungi	0	34	0	0
Fungi	0	0	0	34
Fungi	0	0	0	0
Fungi	0	0	0	0
Fungi	33	0	0	0
Fungi	33	0	0	0
Fungi	33	0	0	0
Fungi	33	0	0	0
Fungi	33	0	0	0
Fungi	33	0	0	0
Fungi	33	0	0	0
Fungi	33	0	0	0
Fungi	33	0	0	0
Fungi	33	0	0	0
Fungi	33	0	0	0
Fungi	0	33	0	0
Fungi	0	33	0	0
Fungi	0	33	0	0
Fungi	0	5	13	0
Fungi	0	0	33	0
Fungi	0	0	0	0
Fungi	0	0	0	0
Fungi	0	0	0	0
Fungi	32	0	0	0
Fungi	32	0	0	0
Fungi	32	0	0	0
Fungi	32	0	0	0

Fungi	32	0	0	0
Fungi	32	0	0	0
Fungi	32	0	0	0
Fungi	32	0	0	0
Fungi	32	0	0	0
Fungi	32	0	0	0
Fungi	32	0	0	0
Fungi	32	0	0	0
Fungi	32	0	0	0
Fungi	32	0	0	0
Fungi	32	0	0	0
Fungi	0	32	0	0
Fungi	0	32	0	0
Fungi	0	32	0	0
Fungi	0	0	5	0
Fungi	0	0	0	0
Fungi	31	0	0	0
Fungi	31	0	0	0
Fungi	31	0	0	0
Fungi	31	0	0	0
Fungi	31	0	0	0
Fungi	31	0	0	0
Fungi	31	0	0	0
Fungi	31	0	0	0
Fungi	31	0	0	0
Fungi	31	0	0	0
Fungi	31	0	0	0
Fungi	31	0	0	0
Fungi	31	0	0	0
Fungi	0	31	0	0
Fungi	0	31	0	0
Fungi	0	31	0	0
Fungi	0	31	0	0
Fungi	0	0	31	0
Fungi	0	0	0	0
Fungi	0	0	0	0
Fungi	30	0	0	0
Fungi	30	0	0	0
Fungi	30	0	0	0
Fungi	30	0	0	0
Fungi	30	0	0	0
Fungi	0	30	0	0
Fungi	0	30	0	0
Fungi	0	30	0	0
Fungi	0	30	0	0
Fungi	0	30	0	0
Fungi	0	30	0	0
Fungi	0	30	0	0
Fungi	0	10	0	0
Fungi	0	2	0	0

Fungi	0	0	30	0
Fungi	0	0	0	0
Fungi	0	0	0	0
Fungi	0	0	0	0
Fungi	0	0	0	0
Fungi	29	0	0	0
Fungi	29	0	0	0
Fungi	29	0	0	0
Fungi	29	0	0	0
Fungi	29	0	0	0
Fungi	29	0	0	0
Fungi	29	0	0	0
Fungi	29	0	0	0
Fungi	12	17	0	0
Fungi	0	29	0	0
Fungi	0	29	0	0
Fungi	0	29	0	0
Fungi	0	29	0	0
Fungi	0	29	0	0
Fungi	0	29	0	0
Fungi	0	0	29	0
Fungi	0	0	0	0
Fungi	0	0	0	0
Fungi	0	0	0	0
Fungi	0	0	0	0
Fungi	28	0	0	0
Fungi	28	0	0	0
Fungi	28	0	0	0
Fungi	28	0	0	0
Fungi	28	0	0	0
Fungi	28	0	0	0
Fungi	28	0	0	0
Fungi	28	0	0	0
Fungi	28	0	0	0
Fungi	28	0	0	0
Fungi	0	28	0	0
Fungi	0	28	0	0
Fungi	0	28	0	0
Fungi	0	28	0	0
Fungi	0	28	0	0
Fungi	0	28	0	0
Fungi	0	28	0	0
Fungi	0	28	0	0
Fungi	0	0	0	0
Fungi	0	0	0	0
Fungi	0	0	0	0
Fungi	0	0	0	0
Fungi	0	0	0	0
Fungi	27	0	0	0
Fungi	27	0	0	0

Fungi	27	0	0	0
Fungi	27	0	0	0
Fungi	27	0	0	0
Fungi	27	0	0	0
Fungi	27	0	0	0
Fungi	27	0	0	0
Fungi	27	0	0	0
Fungi	27	0	0	0
Fungi	27	0	0	0
Fungi	27	0	0	0
Fungi	27	0	0	0
Fungi	27	0	0	0
Fungi	27	0	0	0
Fungi	27	0	0	0
Fungi	0	27	0	0
Fungi	0	27	0	0
Fungi	0	27	0	0
Fungi	0	27	0	0
Fungi	0	27	0	0
Fungi	0	27	0	0
Fungi	0	27	0	0
Fungi	0	27	0	0
Fungi	0	27	0	0
Fungi	0	0	27	0
Fungi	0	0	0	0
Fungi	0	0	0	0
Fungi	0	0	0	0
Fungi	26	0	0	0
Fungi	26	0	0	0
Fungi	26	0	0	0
Fungi	26	0	0	0
Fungi	26	0	0	0
Fungi	26	0	0	0
Fungi	26	0	0	0
Fungi	26	0	0	0
Fungi	26	0	0	0
Fungi	26	0	0	0
Fungi	26	0	0	0
Fungi	26	0	0	0
Fungi	0	26	0	0
Fungi	0	26	0	0
Fungi	0	26	0	0
Fungi	0	26	0	0
Fungi	0	26	0	0
Fungi	0	26	0	0
Fungi	0	26	0	0
Fungi	0	26	0	0
Fungi	0	26	0	0
Fungi	0	26	0	0
Fungi	0	0	26	0

Fungi	0	0	26	0
Fungi	0	0	0	0
Fungi	25	0	0	0
Fungi	25	0	0	0
Fungi	25	0	0	0
Fungi	25	0	0	0
Fungi	25	0	0	0
Fungi	25	0	0	0
Fungi	25	0	0	0
Fungi	25	0	0	0
Fungi	25	0	0	0
Fungi	25	0	0	0
Fungi	25	0	0	0
Fungi	25	0	0	0
Fungi	25	0	0	0
Fungi	0	25	0	0
Fungi	0	25	0	0
Fungi	0	25	0	0
Fungi	0	25	0	0
Fungi	0	25	0	0
Fungi	0	25	0	0
Fungi	0	25	0	0
Fungi	0	25	0	0
Fungi	0	0	25	0
Fungi	0	0	0	0
Fungi	0	0	0	0
Fungi	0	0	0	0
Fungi	24	0	0	0
Fungi	24	0	0	0
Fungi	24	0	0	0
Fungi	24	0	0	0
Fungi	24	0	0	0
Fungi	24	0	0	0
Fungi	24	0	0	0
Fungi	24	0	0	0
Fungi	24	0	0	0
Fungi	24	0	0	0
Fungi	24	0	0	0
Fungi	24	0	0	0
Fungi	24	0	0	0
Fungi	24	0	0	0
Fungi	24	0	0	0
Fungi	24	0	0	0
Fungi	0	24	0	0
Fungi	0	24	0	0
Fungi	0	24	0	0
Fungi	0	24	0	0
Fungi	0	24	0	0
Fungi	0	24	0	0
Fungi	0	24	0	0

Fungi	0	24	0	0
Fungi	0	24	0	0
Fungi	0	24	0	0
Fungi	0	24	0	0
Fungi	0	0	24	0
Fungi	0	0	0	0
Fungi	23	0	0	0
Fungi	23	0	0	0
Fungi	23	0	0	0
Fungi	23	0	0	0
Fungi	23	0	0	0
Fungi	23	0	0	0
Fungi	23	0	0	0
Fungi	23	0	0	0
Fungi	23	0	0	0
Fungi	23	0	0	0
Fungi	23	0	0	0
Fungi	23	0	0	0
Fungi	23	0	0	0
Fungi	23	0	0	0
Fungi	23	0	0	0
Fungi	23	0	0	0
Fungi	23	0	0	0
Fungi	0	23	0	0
Fungi	0	23	0	0
Fungi	0	23	0	0
Fungi	0	23	0	0
Fungi	0	23	0	0
Fungi	0	23	0	0
Fungi	0	23	0	0
Fungi	0	23	0	0
Fungi	0	23	0	0
Fungi	0	0	23	0
Fungi	0	0	23	0
Fungi	0	0	23	0
Fungi	0	0	0	0
Fungi	22	0	0	0
Fungi	22	0	0	0
Fungi	22	0	0	0
Fungi	22	0	0	0
Fungi	22	0	0	0
Fungi	22	0	0	0
Fungi	22	0	0	0
Fungi	22	0	0	0
Fungi	22	0	0	0
Fungi	22	0	0	0
Fungi	22	0	0	0
Fungi	22	0	0	0
Fungi	22	0	0	0
Fungi	22	0	0	0

Fungi	22	0	0	0
Fungi	8	0	0	0
Fungi	0	22	0	0
Fungi	0	22	0	0
Fungi	0	22	0	0
Fungi	0	22	0	0
Fungi	0	22	0	0
Fungi	0	22	0	0
Fungi	0	22	0	0
Fungi	0	22	0	0
Fungi	0	22	0	0
Fungi	0	22	0	0
Fungi	0	14	8	0
Fungi	0	0	22	0
Fungi	0	0	0	0
Fungi	0	0	0	0
Fungi	0	0	0	0
Fungi	0	0	0	0
Fungi	0	0	0	0
Fungi	21	0	0	0
Fungi	21	0	0	0
Fungi	21	0	0	0
Fungi	21	0	0	0
Fungi	21	0	0	0
Fungi	21	0	0	0
Fungi	21	0	0	0
Fungi	21	0	0	0
Fungi	21	0	0	0
Fungi	21	0	0	0
Fungi	21	0	0	0
Fungi	21	0	0	0
Fungi	21	0	0	0
Fungi	21	0	0	0
Fungi	21	0	0	0
Fungi	21	0	0	0
Fungi	21	0	0	0
Fungi	21	0	0	0
Fungi	10	4	0	0
Fungi	0	21	0	0
Fungi	0	21	0	0
Fungi	0	21	0	0
Fungi	0	21	0	0
Fungi	0	21	0	0
Fungi	0	21	0	0
Fungi	0	0	21	0
Fungi	0	0	0	0
Fungi	0	0	0	0
Fungi	0	0	0	0
Fungi	20	0	0	0
Fungi	20	0	0	0

Fungi	20	0	0	0
Fungi	20	0	0	0
Fungi	20	0	0	0
Fungi	20	0	0	0
Fungi	20	0	0	0
Fungi	20	0	0	0
Fungi	20	0	0	0
Fungi	20	0	0	0
Fungi	20	0	0	0
Fungi	20	0	0	0
Fungi	20	0	0	0
Fungi	20	0	0	0
Fungi	20	0	0	0
Fungi	20	0	0	0
Fungi	20	0	0	0
Fungi	20	0	0	0
Fungi	20	0	0	0
Fungi	20	0	0	0
Fungi	20	0	0	0
Fungi	0	20	0	0
Fungi	0	20	0	0
Fungi	0	20	0	0
Fungi	0	20	0	0
Fungi	0	20	0	0
Fungi	0	20	0	0
Fungi	0	20	0	0
Fungi	0	0	20	0
Fungi	0	0	20	0
Fungi	0	0	20	0
Fungi	0	0	20	0
Fungi	0	0	0	20
Fungi	0	0	0	0
Fungi	0	0	0	0
Fungi	0	0	0	0
Fungi	0	0	0	0
Fungi	0	0	0	0
Fungi	19	0	0	0
Fungi	19	0	0	0
Fungi	19	0	0	0
Fungi	19	0	0	0
Fungi	19	0	0	0
Fungi	19	0	0	0
Fungi	19	0	0	0
Fungi	19	0	0	0
Fungi	19	0	0	0
Fungi	19	0	0	0
Fungi	19	0	0	0
Fungi	19	0	0	0
Fungi	19	0	0	0
Fungi	19	0	0	0

Fungi	19	0	0	0
Fungi	19	0	0	0
Fungi	19	0	0	0
Fungi	19	0	0	0
Fungi	19	0	0	0
Fungi	19	0	0	0
Fungi	19	0	0	0
Fungi	19	0	0	0
Fungi	19	0	0	0
Fungi	4	15	0	0
Fungi	0	19	0	0
Fungi	0	0	0	0
Fungi	0	0	0	0
Fungi	18	0	0	0
Fungi	18	0	0	0
Fungi	18	0	0	0
Fungi	18	0	0	0
Fungi	18	0	0	0
Fungi	18	0	0	0
Fungi	18	0	0	0
Fungi	18	0	0	0
Fungi	18	0	0	0
Fungi	18	0	0	0
Fungi	18	0	0	0
Fungi	18	0	0	0
Fungi	18	0	0	0
Fungi	18	0	0	0
Fungi	18	0	0	0
Fungi	0	18	0	0
Fungi	0	18	0	0
Fungi	0	18	0	0
Fungi	0	18	0	0
Fungi	0	18	0	0
Fungi	0	18	0	0
Fungi	0	6	12	0
Fungi	0	0	18	0
Fungi	0	0	12	0
Fungi	0	0	0	0
Fungi	0	0	0	0
Fungi	0	0	0	0
Fungi	0	0	0	0
Fungi	0	0	0	0
Fungi	17	0	0	0
Fungi	17	0	0	0
Fungi	17	0	0	0
Fungi	17	0	0	0
Fungi	17	0	0	0
Fungi	17	0	0	0
Fungi	17	0	0	0
Fungi	17	0	0	0

Fungi	17	0	0	0
Fungi	17	0	0	0
Fungi	17	0	0	0
Fungi	17	0	0	0
Fungi	17	0	0	0
Fungi	17	0	0	0
Fungi	17	0	0	0
Fungi	17	0	0	0
Fungi	17	0	0	0
Fungi	0	17	0	0
Fungi	0	17	0	0
Fungi	0	17	0	0
Fungi	0	17	0	0
Fungi	0	17	0	0
Fungi	0	17	0	0
Fungi	0	17	0	0
Fungi	0	17	0	0
Fungi	0	17	0	0
Fungi	0	0	17	0
Fungi	0	0	17	0
Fungi	0	0	17	0
Fungi	0	0	0	0
Fungi	0	0	0	0
Fungi	0	0	0	0
Fungi	0	0	0	0
Fungi	0	0	0	0
Fungi	16	0	0	0
Fungi	16	0	0	0
Fungi	16	0	0	0
Fungi	16	0	0	0
Fungi	16	0	0	0
Fungi	16	0	0	0
Fungi	16	0	0	0
Fungi	16	0	0	0
Fungi	16	0	0	0
Fungi	16	0	0	0
Fungi	16	0	0	0
Fungi	16	0	0	0
Fungi	16	0	0	0
Fungi	16	0	0	0
Fungi	16	0	0	0
Fungi	16	0	0	0
Fungi	16	0	0	0
Fungi	16	0	0	0
Fungi	16	0	0	0
Fungi	16	0	0	0
Fungi	9	7	0	0
Fungi	7	0	0	0
Fungi	0	16	0	0
Fungi	0	16	0	0

Fungi	0	16	0	0
Fungi	0	16	0	0
Fungi	0	16	0	0
Fungi	0	16	0	0
Fungi	0	16	0	0
Fungi	0	16	0	0
Fungi	0	16	0	0
Fungi	0	0	16	0
Fungi	0	0	0	0
Fungi	0	0	0	0
Fungi	0	0	0	0
Fungi	0	0	0	0
Fungi	15	0	0	0
Fungi	15	0	0	0
Fungi	15	0	0	0
Fungi	15	0	0	0
Fungi	15	0	0	0
Fungi	15	0	0	0
Fungi	15	0	0	0
Fungi	15	0	0	0
Fungi	15	0	0	0
Fungi	15	0	0	0
Fungi	15	0	0	0
Fungi	15	0	0	0
Fungi	15	0	0	0
Fungi	15	0	0	0
Fungi	15	0	0	0
Fungi	15	0	0	0
Fungi	0	15	0	0
Fungi	0	15	0	0
Fungi	0	15	0	0
Fungi	0	15	0	0
Fungi	0	15	0	0
Fungi	0	9	0	0
Fungi	0	0	0	0
Fungi	0	0	0	0
Fungi	0	0	0	0
Fungi	0	0	0	0
Fungi	14	0	0	0
Fungi	14	0	0	0
Fungi	14	0	0	0
Fungi	14	0	0	0
Fungi	14	0	0	0
Fungi	14	0	0	0
Fungi	14	0	0	0
Fungi	14	0	0	0
Fungi	14	0	0	0
Fungi	14	0	0	0
Fungi	14	0	0	0
Fungi	14	0	0	0

Fungi	10	0	0	0
Fungi	6	0	0	0
Fungi	0	14	0	0
Fungi	0	14	0	0
Fungi	0	14	0	0
Fungi	0	14	0	0
Fungi	0	14	0	0
Fungi	0	14	0	0
Fungi	0	14	0	0
Fungi	0	14	0	0
Fungi	0	14	0	0
Fungi	0	0	14	0
Fungi	0	0	0	14
Fungi	0	0	0	0
Fungi	0	0	0	0
Fungi	0	0	0	0
Fungi	0	0	0	0
Fungi	0	0	0	0
Fungi	0	0	0	0
Fungi	0	0	0	0
Fungi	0	0	0	0
Fungi	0	0	0	0
Fungi	0	0	0	0
Fungi	13	0	0	0
Fungi	13	0	0	0
Fungi	13	0	0	0
Fungi	13	0	0	0
Fungi	13	0	0	0
Fungi	13	0	0	0
Fungi	0	13	0	0
Fungi	0	13	0	0
Fungi	0	13	0	0
Fungi	0	13	0	0
Fungi	0	13	0	0
Fungi	0	13	0	0
Fungi	0	13	0	0
Fungi	0	13	0	0
Fungi	0	0	0	0
Fungi	0	0	0	0
Fungi	0	0	0	0
Fungi	0	0	0	0
Fungi	0	0	0	0
Fungi	0	0	0	0
Fungi	0	0	0	0
Fungi	0	0	0	0
Fungi	0	0	0	0
Fungi	0	0	0	0
Fungi	0	0	0	0
Fungi	0	0	0	0
Fungi	12	0	0	0

Fungi	12	0	0	0
Fungi	12	0	0	0
Fungi	12	0	0	0
Fungi	12	0	0	0
Fungi	12	0	0	0
Fungi	12	0	0	0
Fungi	12	0	0	0
Fungi	12	0	0	0
Fungi	12	0	0	0
Fungi	12	0	0	0
Fungi	12	0	0	0
Fungi	12	0	0	0
Fungi	12	0	0	0
Fungi	12	0	0	0
Fungi	0	12	0	0
Fungi	0	12	0	0
Fungi	0	12	0	0
Fungi	0	12	0	0
Fungi	0	12	0	0
Fungi	0	6	0	0
Fungi	0	0	12	0
Fungi	0	0	0	0
Fungi	0	0	0	0
Fungi	0	0	0	0
Fungi	0	0	0	0
Fungi	0	0	0	0
Fungi	0	0	0	0
Fungi	0	0	0	0
Fungi	0	0	0	0
Fungi	0	0	0	0
Fungi	0	0	0	0
Fungi	0	0	0	0
Fungi	0	0	0	0
Fungi	0	0	0	0
Fungi	0	0	0	0
Fungi	11	0	0	0
Fungi	11	0	0	0
Fungi	11	0	0	0
Fungi	11	0	0	0
Fungi	11	0	0	0
Fungi	11	0	0	0
Fungi	11	0	0	0
Fungi	11	0	0	0
Fungi	11	0	0	0
Fungi	11	0	0	0
Fungi	11	0	0	0
Fungi	0	11	0	0
Fungi	0	11	0	0
Fungi	0	11	0	0

Fungi	0	11	0	0
Fungi	0	0	11	0
Fungi	0	0	11	0
Fungi	0	0	0	0
Fungi	0	0	0	0
Fungi	0	0	0	0
Fungi	0	0	0	0
Fungi	0	0	0	0
Fungi	0	0	0	0
Fungi	0	0	0	0
Fungi	0	0	0	0
Fungi	0	0	0	0
Fungi	0	0	0	0
Fungi	0	0	0	0
Fungi	10	0	0	0
Fungi	10	0	0	0
Fungi	10	0	0	0
Fungi	10	0	0	0
Fungi	10	0	0	0
Fungi	10	0	0	0
Fungi	10	0	0	0
Fungi	10	0	0	0
Fungi	10	0	0	0
Fungi	10	0	0	0
Fungi	10	0	0	0
Fungi	10	0	0	0
Fungi	10	0	0	0
Fungi	10	0	0	0
Fungi	5	5	0	0
Fungi	0	10	0	0
Fungi	0	10	0	0
Fungi	0	10	0	0
Fungi	0	10	0	0
Fungi	0	10	0	0
Fungi	0	0	0	0
Fungi	0	0	0	0
Fungi	0	0	0	0
Fungi	0	0	0	0
Fungi	0	0	0	0
Fungi	0	0	0	0
Fungi	0	0	0	0
Fungi	0	0	0	0
Fungi	0	0	0	0
Fungi	0	0	0	0
Fungi	0	0	0	0
Fungi	9	0	0	0
Fungi	9	0	0	0
Fungi	9	0	0	0
Fungi	9	0	0	0
Fungi	9	0	0	0

Fungi	9	0	0	0
Fungi	9	0	0	0
Fungi	9	0	0	0
Fungi	9	0	0	0
Fungi	9	0	0	0
Fungi	9	0	0	0
Fungi	9	0	0	0
Fungi	6	0	3	0
Fungi	0	9	0	0
Fungi	0	9	0	0
Fungi	0	9	0	0
Fungi	0	9	0	0
Fungi	0	3	0	0
Fungi	0	0	9	0
Fungi	0	0	9	0
Fungi	0	0	9	0
Fungi	0	0	9	0
Fungi	0	0	9	0
Fungi	0	0	4	0
Fungi	0	0	0	0
Fungi	0	0	0	0
Fungi	0	0	0	0
Fungi	0	0	0	0
Fungi	0	0	0	0
Fungi	0	0	0	0
Fungi	0	0	0	0
Fungi	0	0	0	0
Fungi	0	0	0	0
Fungi	0	0	0	0
Fungi	0	0	0	0
Fungi	0	0	0	0
Fungi	0	0	0	0
Fungi	0	0	0	0
Fungi	8	0	0	0
Fungi	8	0	0	0
Fungi	8	0	0	0
Fungi	8	0	0	0
Fungi	8	0	0	0
Fungi	8	0	0	0
Fungi	8	0	0	0
Fungi	8	0	0	0
Fungi	8	0	0	0
Fungi	8	0	0	0
Fungi	0	8	0	0
Fungi	0	8	0	0
Fungi	0	8	0	0
Fungi	0	0	8	0
Fungi	0	0	8	0
Fungi	0	0	2	0
Fungi	0	0	0	0

Fungi	0	0	0	0
Fungi	0	0	0	0
Fungi	0	0	0	0
Fungi	0	0	0	0
Fungi	0	0	0	0
Fungi	0	0	0	0
Fungi	0	0	0	0
Fungi	0	0	0	0
Fungi	0	0	0	0
Fungi	0	0	0	0
Fungi	0	0	0	0
Fungi	0	0	0	0
Fungi	0	0	0	0
Fungi	0	0	0	0
Fungi	0	0	0	0
Fungi	0	0	0	0
Fungi	0	0	0	0
Fungi	7	0	0	0
Fungi	7	0	0	0
Fungi	7	0	0	0
Fungi	7	0	0	0
Fungi	7	0	0	0
Fungi	7	0	0	0
Fungi	7	0	0	0
Fungi	7	0	0	0
Fungi	7	0	0	0
Fungi	0	7	0	0
Fungi	0	7	0	0
Fungi	0	7	0	0
Fungi	0	7	0	0
Fungi	0	7	0	0
Fungi	0	0	7	0
Fungi	0	0	0	0
Fungi	0	0	0	0
Fungi	0	0	0	0
Fungi	0	0	0	0
Fungi	0	0	0	0
Fungi	0	0	0	0
Fungi	0	0	0	0
Fungi	0	0	0	0
Fungi	0	0	0	0
Fungi	0	0	0	0
Fungi	0	0	0	0
Fungi	0	0	0	0
Fungi	0	0	0	0
Fungi	0	0	0	0
Fungi	0	0	0	0
Fungi	0	0	0	0
Fungi	0	0	0	0
Fungi	0	0	0	0

Fungi	0	0	0	0
Fungi	0	0	0	0
Fungi	6	0	0	0
Fungi	6	0	0	0
Fungi	6	0	0	0
Fungi	0	6	0	0
Fungi	0	6	0	0
Fungi	0	6	0	0
Fungi	0	3	3	0
Fungi	0	0	6	0
Fungi	0	0	6	0
Fungi	0	0	0	0
Fungi	0	0	0	0
Fungi	0	0	0	0
Fungi	0	0	0	0
Fungi	0	0	0	0
Fungi	0	0	0	0
Fungi	0	0	0	0
Fungi	0	0	0	0
Fungi	0	0	0	0
Fungi	0	0	0	0
Fungi	0	0	0	0
Fungi	0	0	0	0
Fungi	0	0	0	0
Fungi	0	0	0	0
Fungi	0	0	0	0
Fungi	0	0	0	0
Fungi	0	0	0	0
Fungi	0	0	0	0
Fungi	0	0	0	0
Fungi	0	0	0	0
Fungi	0	0	0	0
Fungi	0	0	0	0
Fungi	0	0	0	0
Fungi	0	0	0	0
Fungi	5	0	0	0
Fungi	5	0	0	0
Fungi	5	0	0	0
Fungi	5	0	0	0
Fungi	5	0	0	0
Fungi	5	0	0	0
Fungi	5	0	0	0
Fungi	5	0	0	0
Fungi	5	0	0	0
Fungi	0	5	0	0
Fungi	0	5	0	0
Fungi	0	5	0	0
Fungi	0	5	0	0
Fungi	0	0	5	0
Fungi	0	0	5	0

Fungi	0	0	0	0
Fungi	0	0	0	0
Fungi	0	0	0	0
Fungi	0	0	0	0
Fungi	0	0	0	0
Fungi	0	0	0	0
Fungi	0	0	0	0
Fungi	0	0	0	0
Fungi	0	0	0	0
Fungi	0	0	0	0
Fungi	0	0	0	0
Fungi	0	0	0	0
Fungi	0	0	0	0
Fungi	0	0	0	0
Fungi	0	0	0	0
Fungi	0	0	0	0
Fungi	0	0	0	0
Fungi	0	0	0	0
Fungi	4	0	0	0
Fungi	4	0	0	0
Fungi	4	0	0	0
Fungi	4	0	0	0
Fungi	2	0	0	0
Fungi	0	4	0	0
Fungi	0	4	0	0
Fungi	0	0	4	0
Fungi	0	0	4	0
Fungi	0	0	4	0
Fungi	0	0	4	0
Fungi	0	0	0	0
Fungi	0	0	0	0
Fungi	0	0	0	0
Fungi	0	0	0	0
Fungi	0	0	0	0
Fungi	0	0	0	0
Fungi	0	0	0	0
Fungi	0	0	0	0
Fungi	0	0	0	0
Fungi	0	0	0	0
Fungi	0	0	0	0
Fungi	0	0	0	0
Fungi	0	0	0	0
Fungi	0	0	0	0
Fungi	0	0	0	0
Fungi	0	0	0	0
Fungi	0	0	0	0
Fungi	0	0	0	0
Fungi	3	0	0	0
Fungi	3	0	0	0
Fungi	3	0	0	0

Fungi	3	0	0	0
Fungi	3	0	0	0
Fungi	3	0	0	0
Fungi	3	0	0	0
Fungi	3	0	0	0
Fungi	3	0	0	0
Fungi	3	0	0	0
Fungi	3	0	0	0
Fungi	3	0	0	0
Fungi	3	0	0	0
Fungi	3	0	0	0
Fungi	3	0	0	0
Fungi	3	0	0	0
Fungi	0	3	0	0
Fungi	0	3	0	0
Fungi	0	3	0	0
Fungi	0	3	0	0
Fungi	0	3	0	0
Fungi	0	3	0	0
Fungi	0	3	0	0
Fungi	0	3	0	0
Fungi	0	3	0	0
Fungi	0	0	3	0
Fungi	0	0	3	0
Fungi	0	0	3	0
Fungi	0	0	3	0
Fungi	0	0	3	0
Fungi	0	0	3	0
Fungi	0	0	0	0
Fungi	0	0	0	0
Fungi	0	0	0	0
Fungi	0	0	0	0
Fungi	0	0	0	0
Fungi	0	0	0	0
Fungi	0	0	0	0
Fungi	0	0	0	0
Fungi	0	0	0	0
Fungi	0	0	0	0
Fungi	0	0	0	0
Fungi	0	0	0	0
Fungi	0	0	0	0
Fungi	0	0	0	0
Fungi	0	0	0	0
Fungi	0	0	0	0
Fungi	0	0	0	0
Fungi	0	0	0	0
Fungi	0	0	0	0
Fungi	0	0	0	0
Fungi	0	0	0	0
Fungi	0	0	0	0

Fungi	0	0	0	0
Fungi	0	0	0	0
Fungi	0	0	0	0
Fungi	0	0	0	0
Fungi	0	0	0	0
Fungi	0	0	0	0
Fungi	0	0	0	0
Fungi	0	0	0	0
Fungi	0	0	0	0
Fungi	0	0	0	0
Fungi	0	0	0	0
Fungi	0	0	0	0
Fungi	0	0	0	0
Fungi	0	0	0	0
Fungi	0	0	0	0
Fungi	0	0	0	0
Fungi	0	0	0	0
Fungi	0	0	0	0
Fungi	0	0	0	0
Fungi	0	0	0	0
Fungi	0	0	0	0
Fungi	0	0	0	0
Fungi	0	0	0	0
Fungi	0	0	0	0
Fungi	0	0	0	0
Fungi	0	0	0	0
Fungi	0	0	0	0
Fungi	0	0	0	0
Fungi	2	0	0	0
Fungi	2	0	0	0
Fungi	2	0	0	0
Fungi	2	0	0	0
Fungi	2	0	0	0
Fungi	2	0	0	0
Fungi	2	0	0	0
Fungi	2	0	0	0
Fungi	2	0	0	0
Fungi	2	0	0	0
Fungi	2	0	0	0
Fungi	2	0	0	0
Fungi	2	0	0	0
Fungi	0	2	0	0
Fungi	0	2	0	0
Fungi	0	2	0	0
Fungi	0	2	0	0
Fungi	0	2	0	0
Fungi	0	2	0	0
Fungi	0	2	0	0
Fungi	0	2	0	0
Fungi	0	2	0	0

Fungi	0	0	2	0
Fungi	0	0	2	0
Fungi	0	0	2	0
Fungi	0	0	2	0
Fungi	0	0	2	0
Fungi	0	0	2	0
Fungi	0	0	2	0
Fungi	0	0	2	0
Fungi	0	0	2	0
Fungi	0	0	2	0
Fungi	0	0	2	0
Fungi	0	0	2	0
Fungi	0	0	2	0
Fungi	0	0	0	2
Fungi	0	0	0	2
Fungi	0	0	0	0
Fungi	0	0	0	0
Fungi	0	0	0	0
Fungi	0	0	0	0
Fungi	0	0	0	0
Fungi	0	0	0	0
Fungi	0	0	0	0
Fungi	0	0	0	0
Fungi	0	0	0	0
Fungi	0	0	0	0
Fungi	0	0	0	0
Fungi	0	0	0	0
Fungi	0	0	0	0
Fungi	0	0	0	0
Fungi	0	0	0	0
Fungi	0	0	0	0
Fungi	0	0	0	0
Fungi	0	0	0	0
Fungi	0	0	0	0
Fungi	0	0	0	0
Fungi	0	0	0	0
Fungi	0	0	0	0
Fungi	0	0	0	0
Fungi	0	0	0	0
Fungi	0	0	0	0
Fungi	0	0	0	0
Fungi	0	0	0	0
Fungi	0	0	0	0
Fungi	0	0	0	0
Fungi	0	0	0	0
Fungi	0	0	0	0
Fungi	0	0	0	0
Fungi	0	0	0	0
Fungi	0	0	0	0
Fungi	0	0	0	0

Fungi	0	0	0	0
Fungi	0	0	0	0
Fungi	0	0	0	0
Fungi	0	0	0	0
Fungi	0	0	0	0
Fungi	0	0	0	0
Fungi	0	0	0	0
Fungi	0	0	0	0
Fungi	0	0	0	0
Fungi	0	0	0	0
Fungi	0	0	0	0
Fungi	0	0	0	0
Fungi	0	0	0	0
Fungi	0	0	0	0
Fungi	0	0	0	0
Fungi	0	0	0	0
Fungi	0	0	0	0
Fungi	0	0	0	0
Fungi	0	0	0	0
Fungi	0	0	0	0
Fungi	0	0	0	0
Fungi	0	0	0	0
Fungi	0	0	0	0

LD2293	LD2299	LD2304	OH1078	OH2208	OH2226	QP1131	QP65	QP971	SF134
74	1088	452	578	328	1297	408	330	275	92686
22613	8478	8962	752	819	4310	322	2219	379	460
18	433	228	24765	38401	10940	243	507	183	107
35828	12365	14898	424	106	2347	185	181	116	60
11462	13329	10226	470	147	984	143	143	140	86
0	242	110	173	145	291	139	124	128	86
0	507	285	304	123	583	526	30010	3820	82
0	385	250	214	134	386	247	29014	218	73
0	140	473	8105	20980	449	169	69	152	55
9134	7359	6464	142	68	3890	87	180	68	0
11643	5919	4775	306	113	913	166	2385	197	93
43	74	42	71	47	26371	74	51	24	24
0	0	0	0	0	0	0	485	0	0
24	5947	4151	182	64	436	1005	2630	2191	1817
0	183	150	7516	13815	269	100	68	89	36
0	293	152	159	90	285	150	20289	117	54
0	255	147	349	105	470	110	93	95	53
0	266	158	156	84	291	137	80	128	11772
510	279	255	151	553	344	2274	99	2391	1516
2351	2943	1996	340	235	1354	43	208	53	34
3217	3276	2475	85	41	237	22	35	29	17
642	260	425	473	247	411	91	1434	279	54
531	364	402	1361	521	3151	49	208	35	35
1428	227	455	160	76	5936	57	65	46	17
0	206	121	147	69	194	255	6600	674	38
0	3789	22	175	100	95	974	33	906	0
0	61	35	1570	3966	82	31	25	17	10
170	258	101	102	57	208	127	905	90	0
379	265	389	49	0	157	0	56	54	0
2193	1039	733	85	46	174	0	525	0	0
1296	1652	1399	12	36	105	20	0	33	0
0	0	0	0	0	0	0	0	0	0
28	265	190	35	21	421	1478	92	1084	8
2450	729	866	0	0	271	0	0	0	0
151	6	5	1137	498	2350	0	3	0	0
1095	1424	948	0	0	78	39	0	0	0
4	19	27	610	872	1905	24	70	22	0
0	613	532	319	254	583	258	201	143	692
815	1046	1062	0	0	74	0	0	0	28
159	93	131	78	32	550	52	345	152	0
0	10	17	0	0	16	15	0	31	0
16	45	37	729	1059	528	46	501	58	0
848	974	1040	0	16	94	0	0	0	0
0	62	33	40	24	73	60	2231	34	20
1087	578	545	0	0	206	46	18	41	34
3	368	0	123	1174	323	25	19	14	0
1089	491	747	0	0	81	0	119	0	0
812	361	507	34	0	102	0	31	28	0

554	915	748	0	27	39	0	38	0	0
1056	404	601	33	13	233	23	23	22	0
325	209	368	236	59	139	37	238	0	31
368	212	217	249	107	212	0	309	55	0
697	714	476	55	0	109	0	24	19	0
0	1205	0	0	564	0	273	0	249	0
551	686	865	0	0	55	0	30	0	0
960	462	658	0	27	112	38	0	0	0
963	601	540	0	0	0	0	0	0	0
17	0	17	27	13	1972	14	9	12	0
43	41	48	888	927	43	27	18	24	6
0	13	0	21	0	10	0	5	0	0
310	181	219	35	25	179	42	374	114	7
0	34	25	735	827	169	30	12	22	0
0	0	0	0	0	0	21	0	0	0
466	842	431	0	0	43	32	0	0	0
371	609	544	0	22	0	0	0	0	0
546	463	558	50	22	54	0	0	0	0
0	13	0	1585	17	52	15	11	20	0
0	0	0	0	0	0	0	0	0	1596
23	66	58	0	27	97	0	0	0	0
0	0	0	0	0	0	0	0	0	0
492	368	335	0	0	0	0	0	0	65
98	0	46	37	10	137	0	75	24	136
494	371	441	0	0	40	0	0	0	0
1029	86	151	12	6	72	0	6	0	0
0	45	25	25	17	60	31	648	61	56
370	418	459	0	0	43	0	0	16	0
42	9	10	0	0	83	0	0	0	0
0	0	0	0	0	65	0	647	583	0
0	0	0	236	621	0	0	398	0	0
179	141	87	271	190	58	26	0	0	0
0	14	22	18	13	59	28	628	18	8
0	0	0	20	0	0	0	0	0	0
31	121	118	96	47	121	24	67	22	17
0	0	0	326	178	695	0	0	0	0
137	71	80	79	124	248	23	36	24	0
220	456	470	0	0	30	0	0	0	0
0	0	0	0	0	0	0	0	0	0
57	11	28	347	103	573	0	0	0	12
0	0	0	0	67	1048	13	42	0	22
0	0	0	0	0	0	0	8	4	0
0	0	0	0	0	0	0	0	0	0
0	0	326	311	178	8	42	0	43	16
372	40	235	0	0	0	0	0	0	0
400	223	414	0	0	97	0	0	0	0
112	21	0	36	14	60	21	54	74	0
0	0	0	0	0	0	0	1116	0	0
399	298	200	0	0	72	0	0	0	18
0	0	35	0	34	0	0	0	0	0

0	0	0	0	0	17	0	0	0	0
0	0	0	0	0	0	0	0	0	0
694	149	136	0	0	0	0	0	0	0
440	143	216	0	0	42	0	0	0	0
0	0	0	0	0	0	0	0	0	0
0	0	0	0	0	81	0	44	0	0
0	0	0	8	0	0	0	15	0	0
230	290	290	0	0	38	0	0	0	0
0	0	0	21	24	0	0	12	0	0
243	296	312	0	0	0	0	0	0	0
0	0	0	0	0	0	0	0	0	0
0	11	0	12	0	765	11	0	0	0
0	0	0	0	0	0	0	0	0	0
0	8	363	28	0	0	194	38	90	13
0	0	0	0	0	800	0	0	0	0
555	99	145	0	0	0	0	0	0	0
257	332	205	0	0	0	0	0	0	0
272	230	246	0	0	0	0	0	0	0
311	131	81	44	32	135	0	0	0	0
64	43	34	87	186	231	70	0	0	0
0	0	0	393	333	12	0	0	6	0
198	223	264	0	0	23	0	0	5	0
0	0	0	0	0	0	0	0	0	0
292	172	211	0	0	32	0	0	0	0
3	11	11	197	125	36	5	4	7	16
0	0	0	0	0	0	0	0	0	0
0	4	10	164	472	12	4	6	4	5
32	12	0	200	93	320	0	0	0	0
0	0	0	49	44	0	0	0	0	477
0	155	0	112	105	38	0	0	0	26
0	0	0	0	0	0	0	0	0	0
0	0	6	5	0	256	0	0	0	14
0	0	0	156	466	0	0	0	0	0
178	50	246	12	0	42	0	0	11	7
0	2	0	639	0	0	0	0	0	0
0	44	96	91	0	48	21	0	27	5
121	55	47	0	0	11	0	0	0	0
10	6	0	123	400	22	0	0	6	0
0	0	11	0	0	593	0	0	0	0
0	119	133	88	0	169	90	0	0	0
41	66	48	78	0	82	0	186	22	0
0	20	0	12	13	99	0	22	0	5
219	153	166	0	0	39	0	0	0	0
0	0	0	0	0	0	0	0	0	0
110	168	245	0	0	12	0	7	0	0
84	36	51	0	11	21	8	34	10	8
171	139	221	0	0	25	0	0	0	0
100	77	156	0	12	28	19	32	0	0
0	0	0	0	0	0	0	0	0	0
0	0	36	0	0	56	0	0	0	0

0	24	0	0	0	0	0	0	0	0
0	182	151	32	10	37	9	22	14	0
0	0	0	0	0	0	0	0	0	0
0	0	41	0	0	0	0	0	0	0
266	112	161	0	0	0	0	0	0	0
45	8	0	0	0	26	0	0	0	0
0	0	0	43	125	0	0	12	0	0
215	135	167	0	0	0	0	0	0	0
0	0	0	7	119	0	0	0	0	0
0	95	122	34	11	144	0	17	0	4
0	0	0	0	0	0	0	0	0	0
140	172	178	0	0	0	0	0	0	0
0	7	6	292	111	28	3	0	9	5
5	0	8	8	6	421	7	6	8	0
0	0	0	44	0	0	0	0	0	0
0	0	0	0	0	0	0	0	0	0
0	0	0	0	0	473	0	0	0	0
93	0	0	0	0	181	0	0	59	0
0	0	0	0	0	0	0	0	463	0
142	148	151	0	0	14	0	0	0	0
0	267	180	0	0	0	0	0	0	0
0	9	0	0	89	25	0	0	4	0
0	0	0	0	0	0	0	446	0	0
0	0	0	0	0	0	0	0	0	0
0	5	0	227	52	160	0	0	0	0
0	0	0	0	0	0	0	0	443	0
0	0	37	0	0	111	0	0	0	0
324	0	57	0	0	57	0	0	0	0
73	92	194	0	0	46	12	0	0	0
14	0	27	64	178	32	0	0	0	0
0	10	0	0	0	0	0	421	0	0
0	0	0	0	0	0	0	0	0	0
0	20	18	27	0	40	0	0	17	220
0	0	0	0	0	0	0	0	0	0
0	7	0	0	0	159	0	66	0	0
0	0	0	137	0	279	0	0	0	0
153	76	0	0	0	0	0	57	0	0
3	0	0	0	0	34	0	0	0	0
0	0	17	0	0	0	0	0	0	3
0	0	0	0	0	0	0	0	0	0
115	132	149	0	0	0	0	0	0	0
0	0	13	0	0	38	0	0	0	0
0	14	6	0	8	19	6	0	4	6
0	0	0	0	0	0	0	0	0	0
0	0	0	0	0	0	0	0	0	0
298	35	29	0	0	13	0	0	0	0
0	0	0	0	0	0	0	0	0	0
0	0	365	0	0	0	0	0	0	0
0	0	0	124	236	0	0	0	0	0
0	0	0	38	254	55	4	0	0	0

0	0	0	0	0	32	0	0	0	0
135	99	72	0	0	0	0	0	0	0
14	9	13	0	0	0	0	0	0	0
69	35	19	55	0	35	0	11	0	0
0	0	0	0	0	0	0	41	305	0
37	11	17	0	30	44	0	15	0	0
6	52	0	69	31	150	0	0	0	0
0	0	0	0	0	0	0	0	0	0
73	262	0	0	0	0	0	0	0	0
0	0	0	205	0	0	0	0	0	0
0	0	0	0	0	0	167	40	126	0
0	0	0	0	0	0	0	330	0	0
150	124	29	9	0	15	0	0	0	0
0	0	0	0	0	0	0	0	0	299
0	0	0	0	0	0	0	0	0	0
195	0	0	0	0	98	0	0	0	0
0	0	25	98	147	0	0	0	0	0
0	0	0	97	44	176	0	0	0	0
0	0	0	0	0	0	0	0	0	0
4	0	0	0	0	10	0	0	0	0
0	0	0	158	147	0	0	0	0	0
0	0	0	0	0	0	0	0	0	103
0	0	0	0	0	0	0	0	0	0
0	0	0	0	0	0	0	0	0	0
0	0	0	0	0	0	0	0	0	0
0	0	0	0	0	0	0	0	0	0
0	0	0	0	0	0	0	0	0	0
86	0	209	0	0	0	0	0	0	0
0	0	294	0	0	0	0	0	0	0
0	0	0	0	0	0	0	0	0	0
0	0	0	0	0	0	0	0	0	0
0	0	0	107	155	0	0	0	0	0
158	0	125	0	0	0	0	0	0	0
0	0	0	0	27	0	0	0	0	0
0	0	0	0	276	0	0	0	0	0
0	0	0	0	0	0	0	0	0	0
0	0	0	0	0	0	0	0	0	0
0	0	0	35	16	34	0	8	0	0
11	29	14	125	26	34	2	4	0	0
0	0	0	0	0	0	0	0	0	0
0	0	0	0	0	0	0	0	258	0
81	0	57	0	0	0	0	42	0	0
0	0	0	0	0	0	201	52	0	0
16	0	0	24	39	5	0	0	0	0
0	0	0	0	0	0	0	0	0	0
0	0	0	0	0	0	0	0	0	0
0	180	66	0	0	0	0	0	0	0
0	9	7	4	5	10	12	167	12	0
0	0	0	232	11	0	0	0	0	0
0	0	0	0	230	0	0	13	0	0

206	16	7	0	0	12	0	0	0	0
0	0	0	0	0	0	0	240	0	0
0	13	11	115	22	31	4	6	0	0
0	0	0	0	0	0	0	0	0	0
18	0	0	53	38	128	0	0	0	0
0	0	0	0	0	0	0	0	0	0
0	0	0	219	0	17	0	0	0	0
0	0	0	0	0	0	0	0	235	0
0	0	0	7	0	75	0	25	0	15
0	0	0	0	0	0	0	0	0	0
229	0	0	0	0	0	0	0	0	0
0	0	0	82	40	106	0	0	0	0
0	0	0	0	0	0	0	0	0	0
0	0	0	0	0	0	0	0	0	2
0	0	0	0	0	0	0	0	0	0
0	0	0	0	0	33	0	0	0	0
0	121	70	0	0	0	0	0	0	0
85	0	50	0	0	0	0	0	0	0
20	0	0	82	0	80	0	0	0	0
75	46	80	0	0	0	0	0	0	0
14	0	0	59	28	36	0	0	0	0
18	12	0	60	0	55	0	0	0	0
0	0	0	0	0	0	0	0	0	0
0	0	0	0	0	0	0	0	0	0
0	0	0	0	0	189	0	0	0	0
0	0	0	0	0	0	0	0	0	0
0	0	0	0	0	0	0	0	0	0
0	0	184	0	0	0	0	0	0	0
0	0	0	55	0	85	0	0	0	0
0	0	0	57	37	86	0	0	0	0
0	0	0	0	0	0	116	61	0	0
0	0	0	0	0	0	0	0	0	0
13	36	48	0	12	30	0	0	0	0
0	0	0	0	0	0	0	0	0	0
0	0	0	32	97	0	0	0	0	7
57	17	84	0	0	9	0	0	0	0
0	0	0	0	0	0	0	0	0	0
0	0	3	4	0	0	0	4	0	0
0	10	0	15	0	13	0	0	11	0
0	0	18	0	0	0	0	0	0	0
0	0	0	0	0	0	0	0	0	0
98	34	30	0	0	0	0	0	0	0
0	75	87	0	0	0	0	0	0	0
0	0	0	69	0	93	0	0	0	0
0	0	0	0	0	0	0	0	0	0
0	0	0	0	0	0	0	0	0	0
0	0	0	0	0	0	0	0	0	0
0	0	0	32	90	0	0	0	0	0
0	0	0	0	0	0	0	0	0	0
0	0	0	0	0	0	0	0	0	0

0	0	0	0	0	0	0	0	0	0
0	0	0	50	91	0	0	17	0	0
0	0	0	0	0	0	0	0	0	0
0	0	0	0	0	0	0	0	0	0
0	0	0	0	0	0	0	0	0	0
43	77	29	0	0	7	0	0	0	0
0	0	0	0	0	0	0	156	0	0
0	0	0	0	0	0	0	0	0	0
36	37	0	11	14	24	0	0	0	3
0	0	0	0	0	0	0	0	0	0
0	0	0	0	0	10	0	0	0	0
37	0	47	0	0	0	0	0	0	0
0	0	136	0	0	0	0	0	0	0
0	0	0	42	45	54	0	0	0	0
0	51	3	0	13	18	0	0	0	0
0	0	0	0	0	0	0	0	0	0
0	0	0	0	0	0	0	0	0	0
0	84	64	0	0	0	0	0	0	0
58	23	66	0	0	0	0	0	0	0
0	0	147	0	0	0	0	0	0	0
0	9	0	0	0	0	0	4	0	0
0	0	3	21	33	43	0	0	4	0
0	0	0	55	23	65	0	0	0	0
0	0	0	0	30	0	0	0	0	0
13	30	48	0	0	38	7	0	0	0
0	0	5	0	0	136	0	0	0	0
0	0	0	0	0	0	0	0	0	0
0	0	92	0	0	0	0	0	0	0
0	0	0	120	18	0	0	0	0	0
0	0	0	0	0	26	0	112	0	0
0	0	0	48	0	0	0	0	0	0
0	0	0	0	0	0	0	0	0	0
0	0	0	0	0	133	0	0	0	0
0	19	83	3	8	0	0	7	0	3
0	0	0	0	0	0	0	0	0	0
0	0	0	0	0	0	0	0	0	0
0	0	2	3	0	123	0	0	2	0
0	10	0	14	0	61	0	0	0	8
0	0	0	0	0	127	0	0	0	0
0	0	0	126	0	0	0	0	0	0
19	33	25	0	9	16	0	0	0	0
34	36	55	0	0	0	0	0	0	0
0	0	0	0	0	0	0	0	0	0
0	0	0	101	20	0	0	0	3	0
0	0	0	0	0	0	0	0	0	0
50	24	44	0	0	5	0	0	0	0
0	0	9	0	0	0	0	0	5	0
0	59	59	0	0	0	0	0	0	0
0	0	0	0	0	0	0	0	0	0
0	0	0	0	0	0	0	0	0	0

0	0	0	9	0	13	0	0	0	0
45	0	54	3	0	5	2	0	0	0
101	0	0	0	0	12	0	0	0	0
57	0	55	0	0	0	0	0	0	0
0	0	6	0	38	0	0	0	0	0
45	65	0	0	0	0	0	0	0	0
0	67	43	0	0	0	0	0	0	0
0	12	0	22	0	0	0	57	0	0
10	26	0	0	0	63	0	0	0	0
0	0	0	0	0	0	0	0	0	0
0	0	0	89	0	15	0	0	0	0
0	0	0	0	0	0	0	108	0	0
0	0	18	0	0	0	0	0	0	0
0	0	0	0	0	9	0	0	0	0
0	0	0	0	0	0	0	0	0	0
0	0	6	3	57	0	6	0	10	13
0	0	0	0	0	107	0	0	0	0
0	0	0	0	0	0	0	0	0	0
16	30	0	0	0	0	31	0	27	0
53	0	50	0	0	0	0	0	0	0
0	49	37	0	0	0	5	7	0	0
0	0	0	0	0	0	0	0	0	0
0	0	0	0	0	0	0	0	102	0
0	0	0	0	0	0	0	0	0	0
13	0	0	0	0	17	0	0	0	0
0	9	0	84	0	7	0	0	0	0
0	0	0	0	0	0	0	0	0	0
0	0	0	0	0	0	0	0	0	0
0	0	0	59	40	0	0	0	0	0
0	19	0	39	0	0	0	0	0	0
0	3	4	51	35	2	0	3	0	0
0	0	98	0	0	0	0	0	0	0
0	0	0	0	0	0	0	0	0	0
12	0	0	0	0	0	0	0	0	0
0	0	0	0	0	0	0	0	0	0
19	77	0	0	0	0	0	0	0	0
28	34	33	0	0	0	0	0	0	0
0	0	0	0	39	56	0	0	0	0
0	0	0	56	0	38	0	0	0	0
28	65	0	0	0	0	0	0	0	0
0	0	0	0	0	0	0	0	0	0
0	0	0	0	0	25	0	0	0	0
0	0	0	0	0	0	0	0	0	0
0	0	0	0	0	0	0	91	0	0
0	0	0	0	0	0	0	0	91	0
0	0	0	0	0	0	0	0	0	0
0	0	0	0	0	0	0	0	0	0
0	0	0	0	0	0	0	0	0	0
4	0	0	0	0	80	0	0	0	0
0	0	0	0	0	6	0	0	0	0

0	0	0	57	21	0	0	0	5	0
0	0	0	0	0	0	0	0	0	0
35	0	50	0	0	0	0	0	0	0
0	0	0	0	0	0	0	0	0	0
0	0	0	0	0	0	0	0	0	0
0	0	82	0	0	0	0	0	0	0
0	0	0	53	29	0	0	0	0	0
0	0	0	0	0	0	0	0	0	82
0	0	0	0	0	0	0	0	0	0
0	0	0	81	0	0	0	0	0	0
0	0	0	42	0	39	0	0	0	0
0	0	0	0	0	0	0	0	0	0
0	49	0	0	0	0	18	0	13	0
0	0	0	0	80	0	0	0	0	0
0	0	0	0	0	80	0	0	0	0
0	0	0	0	0	0	0	0	0	80
0	0	0	0	0	0	0	0	0	0
0	0	0	16	0	0	0	0	0	0
26	34	0	0	0	19	0	0	0	0
0	0	0	79	0	0	0	0	0	0
0	0	0	0	0	0	0	0	0	0
0	0	0	29	0	0	0	0	0	0
0	0	0	0	0	0	0	0	0	0
0	78	0	0	0	0	0	0	0	0
37	15	25	0	0	0	0	0	0	0
0	0	0	20	11	46	0	0	0	0
0	15	24	2	0	0	0	7	7	0
27	15	18	0	0	16	0	0	0	0
0	0	0	0	0	0	0	0	0	0
0	0	0	0	0	0	0	0	0	0
67	0	0	0	0	8	0	0	0	0
15	6	22	0	0	0	4	6	22	0
10	0	0	0	0	65	0	0	0	0
0	0	0	75	0	0	0	0	0	0
0	0	0	75	0	0	0	0	0	0
0	0	0	0	0	0	0	60	0	0
0	0	0	0	0	0	0	0	0	0
73	0	0	0	0	0	0	0	0	0
73	0	0	0	0	0	0	0	0	0
6	67	0	0	0	0	0	0	0	0
0	0	0	36	0	0	0	0	0	0
0	0	0	68	0	0	0	0	0	0
0	0	72	0	0	0	0	0	0	0
0	0	72	0	0	0	0	0	0	0
0	0	0	0	0	72	0	0	0	0
0	0	0	0	0	0	0	0	0	0
0	0	0	0	0	0	0	0	0	0
0	0	0	0	0	0	0	0	0	0
0	0	0	0	0	0	0	0	0	0
0	0	0	0	0	0	0	6	0	0

0	0	0	0	0	71	0	0	0	0
0	0	0	0	0	0	0	0	0	0
0	0	0	51	0	0	0	0	0	0
0	0	0	0	0	0	0	39	0	0
0	0	0	70	0	0	0	0	0	0
42	8	14	0	0	0	0	0	0	0
0	69	0	0	0	0	0	0	0	0
0	0	69	0	0	0	0	0	0	0
0	14	0	0	0	0	0	0	0	0
0	68	0	0	0	0	0	0	0	0
0	16	2	0	10	36	0	0	0	0
0	0	0	23	8	36	0	0	0	0
0	0	0	0	0	0	0	0	0	0
0	59	0	0	0	7	0	0	0	0
0	0	13	53	0	0	0	0	0	0
0	0	0	0	0	0	0	0	0	0
0	0	0	0	0	0	0	0	0	0
0	0	0	65	0	0	0	0	0	0
0	0	0	0	65	0	0	0	0	0
7	0	0	0	0	7	0	0	0	0
0	0	0	0	0	0	0	0	0	0
0	0	0	0	0	0	0	0	0	0
0	0	0	0	0	42	0	11	0	0
0	37	0	0	26	0	0	0	0	0
0	0	0	0	0	0	0	0	0	0
0	0	0	0	0	0	0	0	0	0
0	0	0	0	0	0	0	0	0	0
15	21	13	0	0	9	0	0	0	0
0	0	0	0	0	0	0	0	0	0
0	0	0	0	0	0	0	0	0	0
0	0	0	0	0	0	0	0	0	0
0	0	0	0	0	52	0	0	0	0
0	32	29	0	0	0	0	0	0	0
0	0	20	22	0	0	0	0	0	0
0	0	0	11	0	27	5	0	0	0
0	0	12	0	0	0	0	0	0	0
0	0	0	0	0	0	0	34	0	0
0	0	0	0	0	60	0	0	0	0
0	0	0	0	0	0	0	0	0	0
0	0	0	0	0	0	0	0	0	0
0	0	0	0	0	0	0	0	0	0
0	0	0	0	0	0	35	0	24	0
0	0	0	0	0	0	0	0	0	0
0	0	0	0	0	0	0	0	0	0
24	34	0	0	0	0	0	0	0	0
0	37	12	0	0	9	0	0	0	0
0	0	0	0	2	56	0	0	0	0
0	0	0	0	0	0	0	0	0	0
0	0	0	0	0	0	0	0	0	0
0	0	0	0	0	0	0	0	0	0

0	0	0	0	0	8	0	0	0	0
0	36	21	0	0	0	0	0	0	0
0	0	0	8	49	0	0	0	0	0
0	0	0	0	0	57	0	0	0	0
30	26	0	0	0	0	0	0	0	0
0	0	15	0	3	34	0	4	0	0
0	0	0	0	0	0	0	0	0	0
0	0	0	0	0	0	0	0	0	0
0	0	7	0	0	0	0	19	0	0
0	0	55	0	0	0	0	0	0	0
0	0	0	55	0	0	0	0	0	0
0	0	0	3	0	52	0	0	0	0
0	0	0	0	0	10	0	0	0	0
0	0	0	0	0	0	0	0	0	0
0	0	0	0	0	0	0	0	0	0
0	0	0	0	25	8	0	0	0	0
0	0	0	0	0	0	0	0	0	0
29	25	0	0	0	0	0	0	0	0
0	0	54	0	0	0	0	0	0	0
0	0	0	0	0	0	0	0	0	0
0	0	0	0	0	0	0	0	0	0
2	0	10	0	2	15	0	0	0	0
25	0	0	0	0	0	0	0	0	0
0	36	17	0	0	0	0	0	0	0
0	0	0	53	0	0	0	0	0	0
0	0	0	22	4	27	0	0	0	0
0	0	0	0	0	0	0	53	0	0
0	0	0	0	0	0	0	53	0	0
0	0	0	0	0	0	0	0	0	0
0	0	0	0	0	0	0	0	0	0
0	0	0	0	0	0	0	0	0	0
5	0	0	18	0	0	0	0	0	0
0	0	12	0	0	5	0	0	2	0
26	26	0	0	0	0	0	0	0	0
0	0	11	22	0	19	0	0	0	0
0	0	0	51	0	0	0	0	0	0
0	0	0	0	51	0	0	0	0	0
0	0	0	0	0	0	0	0	0	0
0	0	0	39	0	0	0	0	0	0
0	0	24	0	0	0	0	4	0	0
50	0	0	0	0	0	0	0	0	0
0	4	0	46	0	0	0	0	0	0
0	0	0	0	0	50	0	0	0	0
0	0	0	0	0	0	0	0	0	0
0	0	0	0	0	0	0	0	0	0
0	0	14	0	5	23	0	0	0	0
0	12	22	10	0	5	0	0	0	0
0	0	0	39	10	0	0	0	0	0
0	0	0	0	0	0	0	0	0	0
0	0	0	0	0	0	0	0	0	0

0	0	0	8	0	0	0	0	0	0
0	0	0	0	9	39	0	0	0	0
0	0	0	0	0	0	0	0	0	0
0	0	0	0	0	0	0	0	0	0
0	0	0	0	0	0	0	0	0	0
18	20	9	0	0	0	0	0	0	0
0	25	20	0	0	0	2	0	0	0
0	0	38	0	0	9	0	0	0	0
0	0	0	41	0	2	0	0	4	0
0	0	0	0	47	0	0	0	0	0
0	0	0	0	0	0	0	47	0	0
0	0	0	0	0	0	0	0	0	0
0	0	46	0	0	0	0	0	0	0
0	0	46	0	0	0	0	0	0	0
0	0	0	0	43	0	0	0	3	0
0	0	0	0	0	0	0	46	0	0
0	0	0	0	0	0	0	0	0	0
0	0	0	0	0	0	0	0	0	0
15	0	0	0	0	0	0	0	0	0
45	0	0	0	0	0	0	0	0	0
20	10	10	0	0	5	0	0	0	0
0	0	0	0	45	0	0	0	0	0
0	0	0	0	0	45	0	0	0	0
0	0	0	0	0	43	2	0	0	0
0	0	36	0	2	0	0	0	0	0
0	0	0	0	0	0	0	0	0	0
44	0	0	0	0	0	0	0	0	0
0	44	0	0	0	0	0	0	0	0
0	0	6	0	0	0	0	0	0	7
0	0	0	0	0	0	0	41	3	0
0	0	0	0	0	0	0	0	0	0
0	0	0	0	0	0	0	0	0	0
0	0	0	0	0	0	0	0	0	0
0	0	0	0	0	0	0	0	0	0
0	0	0	0	0	0	0	0	0	0
0	0	0	43	0	0	0	0	0	0
0	0	0	0	43	0	0	0	0	0
0	0	0	0	0	0	0	0	0	0
0	42	0	0	0	0	0	0	0	0
0	0	0	16	0	0	0	0	0	0
0	0	0	0	0	0	0	0	0	0
0	0	0	38	0	0	0	0	3	0
40	0	0	0	0	0	0	0	0	0
25	0	0	0	0	15	0	0	0	0
15	17	0	0	0	8	0	0	0	0
3	0	0	12	5	20	0	0	0	0
0	0	0	33	0	7	0	0	0	0
0	0	0	0	0	40	0	0	0	0
0	0	0	0	0	0	0	0	0	0
5	0	8	17	0	0	0	0	0	0

0	0	0	0	0	0	0	0	0	0
39	0	0	0	0	0	0	0	0	0
0	0	0	39	0	0	0	0	0	0
0	0	0	39	0	0	0	0	0	0
0	0	0	36	0	0	0	0	0	0
0	0	0	0	39	0	0	0	0	0
0	0	0	0	0	39	0	0	0	0
0	0	0	0	0	0	0	0	39	0
0	0	0	0	0	0	0	0	0	0
0	5	0	21	0	0	0	0	0	0
0	0	0	0	0	0	0	0	0	0
38	0	0	0	0	0	0	0	0	0
0	23	15	0	0	0	0	0	0	0
0	0	0	38	0	0	0	0	0	0
0	0	0	38	0	0	0	0	0	0
0	0	0	38	0	0	0	0	0	0
0	0	0	0	0	0	0	38	0	0
0	0	0	0	0	0	0	0	38	0
0	0	0	0	0	0	0	0	0	0
0	0	0	0	0	0	0	0	0	0
0	0	12	0	0	0	0	0	0	0
0	0	0	0	0	0	0	0	0	0
14	0	0	0	0	0	0	0	0	0
0	0	17	0	0	6	0	0	0	0
0	0	0	0	0	3	0	3	0	0
26	0	11	0	0	0	0	0	0	0
0	37	0	0	0	0	0	0	0	0
0	15	22	0	0	0	0	0	0	0
0	0	37	0	0	0	0	0	0	0
0	0	0	0	0	37	0	0	0	0
0	0	0	0	0	0	0	0	0	0
0	0	0	0	0	0	0	0	0	0
0	10	0	7	5	0	0	0	0	0
0	0	0	36	0	0	0	0	0	0
0	0	0	36	0	0	0	0	0	0
0	0	0	0	0	36	0	0	0	0
0	0	0	0	0	36	0	0	0	0
0	0	0	0	0	0	0	0	0	0
0	0	0	0	0	0	0	0	0	0
0	0	6	0	0	0	0	0	0	0
0	0	0	0	0	0	0	0	0	0
0	0	0	0	0	0	0	0	0	0
16	19	0	0	0	0	0	0	0	0
0	0	35	0	0	0	0	0	0	0
0	0	0	35	0	0	0	0	0	0
0	0	0	0	0	0	0	0	0	0
0	0	0	0	0	0	0	0	0	0
0	0	0	0	0	0	0	0	0	0
0	15	19	0	0	0	0	0	0	0
0	0	0	0	0	34	0	0	0	0

0	0	0	0	0	0	0	0	0	0
0	0	0	0	0	0	0	0	0	0
0	0	0	0	0	0	0	0	0	0
0	0	0	25	0	0	0	0	0	0
0	0	0	33	0	0	0	0	0	0
0	0	0	0	33	0	0	0	0	0
0	0	0	0	0	33	0	0	0	0
0	0	0	0	0	0	0	33	0	0
0	0	0	0	0	0	0	0	0	0
0	0	0	0	0	0	0	0	0	0
0	0	0	0	0	0	0	0	0	0
0	0	0	0	0	0	0	0	0	0
4	0	0	0	0	0	0	0	0	0
0	0	0	0	0	0	0	0	0	0
23	0	0	0	0	9	0	0	0	0
7	13	0	0	0	0	0	0	0	0
3	0	0	29	0	0	0	0	0	0
0	0	0	28	0	0	4	0	0	0
0	0	0	0	0	32	0	0	0	0
0	0	0	0	0	0	0	32	0	0
0	0	0	0	0	0	0	0	0	0
0	31	0	0	0	0	0	0	0	0
0	0	0	31	0	0	0	0	0	0
0	0	0	31	0	0	0	0	0	0
0	0	0	0	0	0	0	0	0	31
0	0	0	0	0	0	0	0	0	0
0	0	0	0	0	0	0	0	0	0
0	0	0	0	0	19	0	0	0	0
0	0	30	0	0	0	0	0	0	0
0	0	26	0	0	0	0	0	0	4
0	0	0	30	0	0	0	0	0	0
0	0	0	27	0	0	0	3	0	0
0	0	0	0	0	30	0	0	0	0
0	0	0	0	0	0	0	30	0	0
0	0	0	0	0	0	0	0	0	0
0	0	0	0	0	0	0	0	0	0
0	0	0	0	0	0	0	0	0	0
23	6	0	0	0	0	0	0	0	0
13	0	16	0	0	0	0	0	0	0
0	0	0	29	0	0	0	0	0	0
0	0	0	29	0	0	0	0	0	0
0	0	0	29	0	0	0	0	0	0
0	0	0	15	6	8	0	0	0	0
0	0	0	0	0	29	0	0	0	0
0	0	0	0	0	0	0	0	26	3
0	0	0	0	0	0	0	0	0	0
0	0	0	0	0	0	0	0	0	0
0	0	0	0	0	0	0	0	0	0
0	0	0	2	0	0	0	0	0	0
0	0	0	0	0	0	0	0	0	0

0	0	0	0	0	0	0	0	0	0
17	0	11	0	0	0	0	0	0	0
14	7	0	0	0	7	0	0	0	0
0	24	4	0	0	0	0	0	0	0
0	0	28	0	0	0	0	0	0	0
0	0	0	28	0	0	0	0	0	0
0	0	0	0	28	0	0	0	0	0
0	0	0	0	0	24	0	0	0	0
0	0	0	0	0	0	0	0	0	0
0	0	0	0	0	0	0	0	0	0
0	0	0	0	0	0	0	0	0	0
0	0	0	2	0	0	0	0	0	0
0	0	0	0	0	0	0	0	0	0
0	0	0	0	0	0	0	0	0	0
4	0	16	0	0	7	0	0	0	0
0	27	0	0	0	0	0	0	0	0
0	0	27	0	0	0	0	0	0	0
0	0	0	27	0	0	0	0	0	0
0	0	0	27	0	0	0	0	0	0
0	0	0	12	0	15	0	0	0	0
0	0	0	0	0	0	0	27	0	0
0	0	0	0	0	0	0	0	0	12
0	0	0	0	0	0	0	0	0	0
0	0	0	0	0	0	0	0	0	0
0	0	0	0	0	0	0	0	0	0
0	0	0	0	0	0	0	0	0	0
0	0	0	0	0	0	0	0	0	0
0	0	0	0	0	0	0	0	0	0
0	26	0	0	0	0	0	0	0	0
0	26	0	0	0	0	0	0	0	0
0	26	0	0	0	0	0	0	0	0
0	0	0	26	0	0	0	0	0	0
0	0	0	26	0	0	0	0	0	0
0	0	0	7	19	0	0	0	0	0
0	0	0	0	0	26	0	0	0	0
0	0	0	0	0	26	0	0	0	0
0	0	0	0	0	26	0	0	0	0
0	0	0	0	0	0	0	26	0	0
0	0	0	0	0	0	0	0	0	0
0	0	0	0	0	0	0	0	0	0
0	0	0	0	0	0	0	0	0	0
0	0	0	0	0	0	0	0	0	0
0	0	0	0	0	0	0	0	0	0
8	0	0	0	6	0	0	0	0	0
14	0	0	0	0	0	0	11	0	0
0	25	0	0	0	0	0	0	0	0
0	25	0	0	0	0	0	0	0	0
0	0	0	25	0	0	0	0	0	0
0	0	0	25	0	0	0	0	0	0
0	0	0	0	25	0	0	0	0	0

0	0	0	0	0	0	0	25	0	0
0	0	0	0	0	0	0	0	0	0
0	0	0	0	0	0	0	0	0	0
0	0	0	0	0	0	0	0	0	0
0	0	11	0	0	0	0	0	0	0
0	0	0	0	0	0	0	0	0	0
24	0	0	0	0	0	0	0	0	0
0	24	0	0	0	0	0	0	0	0
0	24	0	0	0	0	0	0	0	0
0	3	3	0	0	15	3	0	0	0
0	0	24	0	0	0	0	0	0	0
0	0	11	0	11	0	0	2	0	0
0	0	0	0	0	24	0	0	0	0
0	0	0	0	0	0	0	0	0	0
0	0	0	0	0	0	0	0	0	0
0	0	0	0	0	0	0	0	0	0
0	0	0	0	0	0	0	0	0	0
0	13	0	0	0	0	0	0	0	0
0	23	0	0	0	0	0	0	0	0
0	14	9	0	0	0	0	0	0	0
0	0	0	23	0	0	0	0	0	0
0	0	0	23	0	0	0	0	0	0
0	0	0	23	0	0	0	0	0	0
0	0	0	23	0	0	0	0	0	0
0	0	0	0	23	0	0	0	0	0
0	0	0	0	0	23	0	0	0	0
0	0	0	0	0	23	0	0	0	0
0	0	0	0	0	0	0	0	0	0
0	0	0	0	0	0	0	0	0	0
0	0	0	0	0	0	0	0	0	0
22	0	0	0	0	0	0	0	0	0
22	0	0	0	0	0	0	0	0	0
0	22	0	0	0	0	0	0	0	0
0	0	0	0	22	0	0	0	0	0
0	0	0	0	0	22	0	0	0	0
0	0	0	0	0	22	0	0	0	0
0	0	0	0	0	0	8	0	14	0
0	0	0	0	0	0	0	0	0	0
0	0	0	0	0	0	0	0	0	0
0	0	0	0	0	0	0	0	0	0
0	0	0	0	0	0	0	0	0	0
0	0	0	0	0	0	0	0	0	0
0	0	0	0	0	0	0	0	0	0
0	0	0	0	0	0	0	0	0	0
0	0	0	0	0	0	0	0	0	0
21	0	0	0	0	0	0	0	0	0
21	0	0	0	0	0	0	0	0	0
21	0	0	0	0	0	0	0	0	0
21	0	0	0	0	0	0	0	0	0
21	0	0	0	0	0	0	0	0	0

0	21	0	0	0	0	0	0	0	0
0	21	0	0	0	0	0	0	0	0
0	0	21	0	0	0	0	0	0	0
0	0	21	0	0	0	0	0	0	0
0	0	21	0	0	0	0	0	0	0
0	0	9	12	0	0	0	0	0	0
0	0	0	21	0	0	0	0	0	0
0	0	0	21	0	0	0	0	0	0
0	0	0	0	0	0	0	0	0	0
0	0	0	0	0	0	0	0	0	0
0	0	0	0	0	0	0	0	0	0
0	0	0	0	0	0	0	0	0	0
0	0	0	0	0	0	0	0	0	0
0	0	0	0	0	0	0	0	0	0
0	0	0	0	0	0	0	0	0	0
0	0	0	0	0	0	0	0	0	0
20	0	0	0	0	0	0	0	0	0
20	0	0	0	0	0	0	0	0	0
20	0	0	0	0	0	0	0	0	0
20	0	0	0	0	0	0	0	0	0
4	6	6	0	0	4	0	0	0	0
0	0	16	0	4	0	0	0	0	0
0	0	0	20	0	0	0	0	0	0
0	0	0	0	0	20	0	0	0	0
0	0	0	0	0	17	0	0	0	0
0	0	0	0	0	0	0	20	0	0
0	0	0	0	0	0	0	0	0	0
0	0	0	0	0	0	0	0	0	0
0	0	0	0	0	0	0	0	0	0
0	0	0	0	0	0	0	0	0	0
0	0	0	0	0	0	0	0	0	0
0	0	0	0	0	0	0	0	0	0
0	0	0	0	0	10	0	0	0	0
0	0	0	0	0	0	0	0	0	0
0	19	0	0	0	0	0	0	0	0
0	4	9	0	3	0	0	0	3	0
0	0	19	0	0	0	0	0	0	0
0	0	19	0	0	0	0	0	0	0
0	0	4	15	0	0	0	0	0	0
0	0	0	19	0	0	0	0	0	0
0	0	0	19	0	0	0	0	0	0
0	0	0	19	0	0	0	0	0	0
0	0	0	19	0	0	0	0	0	0
0	0	0	0	0	19	0	0	0	0
0	0	0	0	0	19	0	0	0	0
0	0	0	0	0	0	0	19	0	0
0	0	0	0	0	0	0	0	0	0
0	0	0	0	0	0	0	0	0	0
0	0	0	0	0	0	0	0	0	0
0	0	0	0	0	0	0	0	0	0

0	14	0	0	0	0	0	0	0	0
0	0	0	0	0	0	0	0	0	0
0	0	0	0	0	0	0	0	0	0
0	0	0	0	0	0	0	0	0	0
0	0	0	0	0	0	0	0	0	0
18	0	0	0	0	0	0	0	0	0
4	14	0	0	0	0	0	0	0	0
0	18	0	0	0	0	0	0	0	0
0	18	0	0	0	0	0	0	0	0
0	18	0	0	0	0	0	0	0	0
0	18	0	0	0	0	0	0	0	0
0	7	11	0	0	0	0	0	0	0
0	6	12	0	0	0	0	0	0	0
0	0	18	0	0	0	0	0	0	0
0	0	18	0	0	0	0	0	0	0
0	0	0	18	0	0	0	0	0	0
0	0	0	18	0	0	0	0	0	0
0	0	0	18	0	0	0	0	0	0
0	0	0	18	0	0	0	0	0	0
0	0	0	16	0	0	2	0	0	0
0	0	0	0	0	18	0	0	0	0
0	0	0	0	0	18	0	0	0	0
0	0	0	0	0	18	0	0	0	0
0	0	0	0	0	0	0	0	0	0
0	0	0	0	0	0	0	0	0	0
0	0	0	0	0	0	0	0	0	0
0	0	0	0	0	0	0	0	0	0
0	0	0	0	0	0	0	0	0	0
2	0	0	0	0	0	0	0	0	0
0	0	0	0	0	0	0	0	0	0
0	0	0	0	0	0	0	0	0	0
0	0	0	0	0	0	0	0	0	0
0	0	0	0	0	0	0	0	0	0
0	0	0	0	0	0	0	0	0	0
0	0	0	0	0	0	0	0	0	0
0	0	0	0	0	0	0	0	0	0
0	0	0	0	0	0	0	0	0	0
0	0	0	0	0	0	0	0	0	0
17	0	0	0	0	0	0	0	0	0
13	0	0	0	0	4	0	0	0	0
3	0	0	0	14	0	0	0	0	0
0	17	0	0	0	0	0	0	0	0
0	17	0	0	0	0	0	0	0	0
0	17	0	0	0	0	0	0	0	0
0	0	15	0	0	0	0	0	0	0
0	0	10	0	7	0	0	0	0	0
0	0	0	2	0	15	0	0	0	0
0	0	0	0	0	17	0	0	0	0
0	0	0	0	0	17	0	0	0	0
0	0	0	0	0	0	0	0	0	0

0	0	0	0	0	0	0	0	0	0
0	0	0	0	0	0	0	0	0	0
0	0	0	0	0	0	0	0	0	0
0	0	0	0	3	0	0	0	0	0
0	0	3	4	0	5	0	0	0	0
0	0	0	0	0	0	0	0	0	0
0	0	0	0	0	0	0	0	0	0
0	0	0	0	0	0	0	0	0	0
0	0	0	0	0	0	0	0	0	0
0	0	0	0	0	0	0	0	0	0
0	0	0	0	0	0	0	0	0	0
0	0	0	0	0	9	0	0	0	0
16	0	0	0	0	0	0	0	0	0
16	0	0	0	0	0	0	0	0	0
0	16	0	0	0	0	0	0	0	0
0	16	0	0	0	0	0	0	0	0
0	16	0	0	0	0	0	0	0	0
0	0	0	16	0	0	0	0	0	0
0	0	0	16	0	0	0	0	0	0
0	0	0	16	0	0	0	0	0	0
0	0	0	4	0	0	0	0	0	12
0	0	0	0	16	0	0	0	0	0
0	0	0	0	0	14	0	0	0	0
0	0	0	0	0	0	0	16	0	0
0	0	0	0	0	0	0	0	0	0
0	0	0	0	0	0	0	0	0	0
0	0	0	0	0	0	0	0	0	0
0	0	0	0	0	0	0	0	0	0
0	0	0	0	0	0	0	0	0	0
0	0	0	0	0	0	0	0	0	0
6	0	0	0	0	4	0	0	0	0
0	0	0	0	0	0	0	0	0	0
0	0	0	0	0	0	0	0	0	0
0	0	0	0	0	0	0	0	0	0
0	0	0	0	0	0	0	0	0	0
0	0	0	0	0	0	0	0	0	0
0	0	0	0	0	0	0	0	0	0
0	0	0	0	0	0	0	0	0	0
15	0	0	0	0	0	0	0	0	0
11	0	0	0	4	0	0	0	0	0
9	6	0	0	0	0	0	0	0	0
5	4	6	0	0	0	0	0	0	0
2	10	0	0	0	3	0	0	0	0
0	15	0	0	0	0	0	0	0	0
0	15	0	0	0	0	0	0	0	0
0	15	0	0	0	0	0	0	0	0
0	15	0	0	0	0	0	0	0	0
0	0	15	0	0	0	0	0	0	0
0	0	15	0	0	0	0	0	0	0
0	0	0	15	0	0	0	0	0	0

0	0	0	15	0	0	0	0	0	0
0	0	0	0	0	0	0	0	15	0
0	0	0	0	0	0	0	0	0	15
0	0	0	0	0	0	0	0	0	0
0	0	0	0	0	0	0	0	0	0
0	0	0	0	0	0	0	0	0	0
0	0	0	0	0	0	0	0	0	0
0	0	0	0	0	0	0	0	0	0
0	0	0	0	0	0	0	0	0	0
0	0	0	0	0	0	0	0	0	0
0	0	0	0	0	0	0	0	0	0
0	0	0	0	0	0	0	0	0	0
0	0	0	0	0	0	0	0	0	0
0	0	0	0	0	0	0	0	0	0
0	0	0	0	0	0	0	0	0	0
0	0	0	0	0	0	0	0	0	0
0	0	0	0	0	0	0	0	0	0
0	0	0	0	0	0	0	0	0	0
14	0	0	0	0	0	0	0	0	0
0	14	0	0	0	0	0	0	0	0
0	14	0	0	0	0	0	0	0	0
0	3	11	0	0	0	0	0	0	0
0	0	14	0	0	0	0	0	0	0
0	0	14	0	0	0	0	0	0	0
0	0	14	0	0	0	0	0	0	0
0	0	14	0	0	0	0	0	0	0
0	0	14	0	0	0	0	0	0	0
0	0	0	14	0	0	0	0	0	0
0	0	0	14	0	0	0	0	0	0
0	0	0	14	0	0	0	0	0	0
0	0	0	14	0	0	0	0	0	0
0	0	0	14	0	0	0	0	0	0
0	0	0	14	0	0	0	0	0	0
0	0	0	14	0	0	0	0	0	0
0	0	0	0	5	9	0	0	0	0
0	0	0	0	0	14	0	0	0	0
0	0	0	0	0	14	0	0	0	0
0	0	0	0	0	14	0	0	0	0
0	0	0	0	0	14	0	0	0	0
0	0	0	0	0	14	0	0	0	0
0	0	0	0	0	14	0	0	0	0
0	0	0	0	0	14	0	0	0	0
0	0	0	0	0	14	0	0	0	0
0	0	0	0	0	0	0	14	0	0
0	0	0	0	0	0	0	14	0	0
0	0	0	0	0	0	0	0	14	0
0	0	0	0	0	0	0	0	14	0
0	0	0	0	0	0	0	0	0	0
0	0	0	0	0	0	0	0	0	0
0	0	0	0	0	0	0	0	0	0
0	0	0	0	0	0	0	0	0	0

0	0	0	0	0	0	0	0	0	0
0	0	0	0	0	0	0	0	0	0
0	0	0	0	0	0	0	0	0	0
0	0	0	0	0	0	0	0	0	0
0	0	0	0	0	0	0	0	0	0
0	0	0	0	0	0	0	0	0	0
0	0	0	0	0	0	0	0	0	0
0	0	0	0	0	0	0	0	0	0
0	0	0	0	0	0	0	0	0	0
0	0	0	0	0	0	0	0	0	0
0	0	0	0	9	0	0	0	0	0
0	0	0	0	0	0	0	0	0	0
13	0	0	0	0	0	0	0	0	0
0	13	0	0	0	0	0	0	0	0
0	0	13	0	0	0	0	0	0	0
0	0	0	13	0	0	0	0	0	0
0	0	0	13	0	0	0	0	0	0
0	0	0	13	0	0	0	0	0	0
0	0	0	11	0	2	0	0	0	0
0	0	0	0	13	0	0	0	0	0
0	0	0	0	0	13	0	0	0	0
0	0	0	0	0	13	0	0	0	0
0	0	0	0	0	13	0	0	0	0
0	0	0	0	0	13	0	0	0	0
0	0	0	0	0	13	0	0	0	0
0	0	0	0	0	0	0	13	0	0
0	0	0	0	0	0	0	0	0	13
0	0	0	0	0	0	0	0	0	0
0	0	0	0	0	0	0	0	0	0
0	0	0	0	0	0	0	0	0	0
0	0	0	0	0	0	0	0	0	0
0	0	0	0	0	0	0	0	0	0
0	0	0	0	0	0	0	0	0	0
0	0	0	0	0	0	0	0	0	0
0	0	0	10	0	0	0	0	0	0
0	0	0	0	0	0	0	0	0	0
0	0	0	0	0	0	0	0	0	0
0	0	0	0	0	0	0	0	0	0
0	0	6	0	0	0	0	0	0	0
0	0	0	0	0	0	0	0	0	0
0	0	0	0	0	5	0	0	0	0
0	0	0	0	0	0	0	0	0	0
12	0	0	0	0	0	0	0	0	0
3	0	9	0	0	0	0	0	0	0
3	0	6	0	0	3	0	0	0	0
0	12	0	0	0	0	0	0	0	0
0	12	0	0	0	0	0	0	0	0
0	0	12	0	0	0	0	0	0	0
0	0	12	0	0	0	0	0	0	0
0	0	12	0	0	0	0	0	0	0

0	0	2	10	0	0	0	0	0	0
0	0	0	12	0	0	0	0	0	0
0	0	0	12	0	0	0	0	0	0
0	0	0	12	0	0	0	0	0	0
0	0	0	12	0	0	0	0	0	0
0	0	0	12	0	0	0	0	0	0
0	0	0	5	0	0	0	0	7	0
0	0	0	0	0	0	0	0	0	0
0	0	0	0	0	0	0	0	0	0
0	0	0	0	0	0	0	0	0	0
0	0	0	0	0	0	0	0	0	0
0	0	0	0	0	0	0	0	0	0
0	0	0	0	0	0	0	0	0	0
0	0	0	0	0	0	0	0	0	0
0	0	0	0	0	0	0	0	0	0
0	0	0	0	0	0	0	0	0	0
0	0	0	0	0	0	0	0	0	0
0	11	0	0	0	0	0	0	0	0
0	11	0	0	0	0	0	0	0	0
0	11	0	0	0	0	0	0	0	0
0	11	0	0	0	0	0	0	0	0
0	7	0	4	0	0	0	0	0	0
0	6	0	0	0	0	0	0	0	0
0	0	11	0	0	0	0	0	0	0
0	0	11	0	0	0	0	0	0	0
0	0	11	0	0	0	0	0	0	0
0	0	11	0	0	0	0	0	0	0
0	0	11	0	0	0	0	0	0	0
0	0	9	0	0	2	0	0	0	0
0	0	0	11	0	0	0	0	0	0
0	0	0	11	0	0	0	0	0	0
0	0	0	11	0	0	0	0	0	0
0	0	0	3	0	0	0	8	0	0
0	0	0	0	4	7	0	0	0	0
0	0	0	0	0	11	0	0	0	0
0	0	0	0	0	11	0	0	0	0
0	0	0	0	0	11	0	0	0	0
0	0	0	0	0	11	0	0	0	0
0	0	0	0	0	11	0	0	0	0
0	0	0	0	0	0	0	11	0	0
0	0	0	0	0	0	0	11	0	0
0	0	0	0	0	0	0	0	0	0
0	0	0	0	0	0	0	0	0	0
0	0	0	0	0	0	0	0	0	0
0	0	0	0	0	0	0	0	0	0
0	0	0	0	0	0	0	0	0	0
0	0	0	0	0	0	0	0	0	0
0	0	0	0	0	0	0	0	0	0
0	0	0	0	0	0	0	0	0	0
0	0	0	0	0	0	0	0	0	0

0	0	0	0	0	0	0	0	0	0
0	0	0	0	0	0	0	0	0	0
0	0	0	0	0	0	0	0	0	0
0	0	0	0	0	0	0	0	0	0
0	0	0	0	0	0	0	0	0	0
0	0	0	0	0	0	0	0	0	0
0	0	0	0	0	0	0	0	0	0
0	0	0	0	0	0	0	0	0	0
0	0	0	0	0	7	0	0	0	0
0	0	0	0	0	0	0	0	0	0
0	0	0	0	0	0	0	0	0	0
7	0	0	0	0	0	0	0	0	3
0	10	0	0	0	0	0	0	0	0
0	10	0	0	0	0	0	0	0	0
0	10	0	0	0	0	0	0	0	0
0	10	0	0	0	0	0	0	0	0
0	10	0	0	0	0	0	0	0	0
0	10	0	0	0	0	0	0	0	0
0	10	0	0	0	0	0	0	0	0
0	8	2	0	0	0	0	0	0	0
0	0	10	0	0	0	0	0	0	0
0	0	10	0	0	0	0	0	0	0
0	0	10	0	0	0	0	0	0	0
0	0	10	0	0	0	0	0	0	0
0	0	0	10	0	0	0	0	0	0
0	0	0	5	0	5	0	0	0	0
0	0	0	4	0	6	0	0	0	0
0	0	0	0	10	0	0	0	0	0
0	0	0	0	7	0	0	0	0	0
0	0	0	0	0	10	0	0	0	0
0	0	0	0	0	10	0	0	0	0
0	0	0	0	0	10	0	0	0	0
0	0	0	0	0	10	0	0	0	0
0	0	0	0	0	10	0	0	0	0
0	0	0	0	0	10	0	0	0	0
0	0	0	0	0	10	0	0	0	0
0	0	0	0	0	10	0	0	0	0
0	0	0	0	0	10	0	0	0	0
0	0	0	0	0	0	0	10	0	0
0	0	0	0	0	0	0	10	0	0
0	0	0	0	0	0	0	0	0	0
0	0	0	0	0	0	0	0	0	0
0	0	0	0	0	0	0	0	0	0
0	0	0	0	0	0	0	0	0	0
0	0	0	0	0	0	0	0	0	0
0	0	0	0	0	0	0	0	0	0
0	0	0	0	0	0	0	0	0	0
0	0	0	0	0	0	0	0	0	0
0	0	0	0	0	0	0	0	0	0
0	0	0	0	0	0	0	0	0	0

0	0	0	0	0	5	0	0	0	0
0	9	0	0	0	0	0	0	0	0
0	9	0	0	0	0	0	0	0	0
0	9	0	0	0	0	0	0	0	0
0	0	9	0	0	0	0	0	0	0
0	0	9	0	0	0	0	0	0	0
0	0	9	0	0	0	0	0	0	0
0	0	9	0	0	0	0	0	0	0
0	0	9	0	0	0	0	0	0	0
0	0	9	0	0	0	0	0	0	0
0	0	9	0	0	0	0	0	0	0
0	0	6	0	0	0	0	0	0	0
0	0	0	9	0	0	0	0	0	0
0	0	0	9	0	0	0	0	0	0
0	0	0	9	0	0	0	0	0	0
0	0	0	5	0	0	0	4	0	0
0	0	0	0	9	0	0	0	0	0
0	0	0	0	9	0	0	0	0	0
0	0	0	0	9	0	0	0	0	0
0	0	0	0	0	9	0	0	0	0
0	0	0	0	0	9	0	0	0	0
0	0	0	0	0	9	0	0	0	0
0	0	0	0	0	9	0	0	0	0
0	0	0	0	0	9	0	0	0	0
0	0	0	0	0	9	0	0	0	0
0	0	0	0	0	0	9	0	0	0
0	0	0	0	0	0	0	0	0	0
0	0	0	0	0	0	0	0	0	0
0	0	0	0	0	0	0	0	0	0
0	0	0	0	0	0	0	0	0	0
0	0	0	0	0	0	0	0	0	0
0	0	0	0	0	0	0	0	0	0
0	0	0	0	0	0	0	0	0	0
0	0	0	0	0	0	0	0	0	0
0	0	0	0	0	0	0	0	0	0
0	0	0	0	0	0	0	0	0	0
0	0	0	0	0	0	0	0	0	0
0	0	0	0	0	0	0	0	0	0
0	0	0	0	0	0	0	0	0	0
0	0	0	0	0	0	0	0	0	0
0	0	0	0	0	0	0	0	0	0
0	0	0	0	0	0	0	0	0	0
0	0	0	0	0	0	0	0	0	0
0	0	0	0	0	0	0	0	0	0
0	0	0	0	0	0	0	0	0	0
0	0	0	0	0	0	0	0	0	0
0	0	0	0	0	0	0	0	0	0
8	0	0	0	0	0	0	0	0	0
3	0	0	0	0	0	0	5	0	0
0	8	0	0	0	0	0	0	0	0

0	8	0	0	0	0	0	0	0	0
0	8	0	0	0	0	0	0	0	0
0	0	8	0	0	0	0	0	0	0
0	0	8	0	0	0	0	0	0	0
0	0	8	0	0	0	0	0	0	0
0	0	8	0	0	0	0	0	0	0
0	0	8	0	0	0	0	0	0	0
0	0	0	8	0	0	0	0	0	0
0	0	0	8	0	0	0	0	0	0
0	0	0	8	0	0	0	0	0	0
0	0	0	0	8	0	0	0	0	0
0	0	0	0	8	0	0	0	0	0
0	0	0	0	8	0	0	0	0	0
0	0	0	0	0	8	0	0	0	0
0	0	0	0	0	8	0	0	0	0
0	0	0	0	0	8	0	0	0	0
0	0	0	0	0	8	0	0	0	0
0	0	0	0	0	8	0	0	0	0
0	0	0	0	0	8	0	0	0	0
0	0	0	0	0	0	0	8	0	0
0	0	0	0	0	0	0	0	0	0
0	0	0	0	0	0	0	0	0	0
0	0	0	0	0	0	0	0	0	0
0	0	0	0	0	0	0	0	0	0
0	0	0	0	0	0	0	0	0	0
0	0	0	0	0	0	0	0	0	0
0	0	0	0	0	0	0	0	0	0
0	0	0	0	0	0	0	0	0	0
0	0	0	0	0	0	0	0	0	0
0	0	0	0	0	0	0	0	0	0
0	0	0	0	0	0	0	0	0	0
0	0	0	0	0	0	0	0	0	0
0	0	0	0	0	0	0	0	0	0
0	0	0	0	0	0	0	0	0	0
0	0	0	0	0	0	0	0	0	3
0	0	0	0	0	0	0	0	0	0
0	0	0	0	0	0	0	0	0	0
0	0	0	0	0	0	0	0	0	0
0	0	0	0	0	0	0	0	0	0
0	0	0	0	0	0	0	0	0	0
0	0	0	0	0	0	0	0	0	0
0	0	0	0	0	0	0	0	0	0
0	0	0	0	0	4	0	0	0	0
0	0	0	0	0	0	0	0	0	0
0	0	0	0	0	0	0	0	0	0
5	0	0	0	0	2	0	0	0	0
5	0	0	0	0	2	0	0	0	0
0	7	0	0	0	0	0	0	0	0
0	7	0	0	0	0	0	0	0	0
0	7	0	0	0	0	0	0	0	0

0	7	0	0	0	0	0	0	0	0
0	7	0	0	0	0	0	0	0	0
0	7	0	0	0	0	0	0	0	0
0	7	0	0	0	0	0	0	0	0
0	7	0	0	0	0	0	0	0	0
0	5	0	2	0	0	0	0	0	0
0	0	7	0	0	0	0	0	0	0
0	0	7	0	0	0	0	0	0	0
0	0	7	0	0	0	0	0	0	0
0	0	7	0	0	0	0	0	0	0
0	0	7	0	0	0	0	0	0	0
0	0	7	0	0	0	0	0	0	0
0	0	7	0	0	0	0	0	0	0
0	0	7	0	0	0	0	0	0	0
0	0	7	0	0	0	0	0	0	0
0	0	0	7	0	0	0	0	0	0
0	0	0	7	0	0	0	0	0	0
0	0	0	7	0	0	0	0	0	0
0	0	0	0	7	0	0	0	0	0
0	0	0	0	7	0	0	0	0	0
0	0	0	0	0	7	0	0	0	0
0	0	0	0	0	7	0	0	0	0
0	0	0	0	0	7	0	0	0	0
0	0	0	0	0	7	0	0	0	0
0	0	0	0	0	7	0	0	0	0
0	0	0	0	0	7	0	0	0	0
0	0	0	0	0	7	0	0	0	0
0	0	0	0	0	0	7	0	0	0
0	0	0	0	0	0	0	7	0	0
0	0	0	0	0	0	0	0	7	0
0	0	0	0	0	0	0	0	0	0
0	0	0	0	0	0	0	0	0	0
0	0	0	0	0	0	0	0	0	0
0	0	0	0	0	0	0	0	0	0
0	0	0	0	0	0	0	0	0	0
0	0	0	0	0	0	0	0	0	0
0	0	0	0	0	0	0	0	0	0
0	0	0	0	0	0	0	0	0	0
0	0	0	0	0	0	0	0	0	0
0	0	0	0	0	0	0	0	0	0
0	0	0	0	0	0	0	0	0	0
0	0	0	0	0	0	0	0	0	0
0	0	0	0	0	0	0	0	0	0
0	0	0	0	0	0	0	0	0	0
0	0	0	0	0	0	0	0	0	0
0	0	0	0	0	0	0	0	0	0
0	0	0	0	0	0	0	0	0	0
0	0	0	0	0	0	0	0	0	0
0	0	0	0	0	0	0	0	0	0
0	0	0	0	0	0	0	0	0	0

6	0	0	0	0	0	0	0	0	0
6	0	0	0	0	0	0	0	0	0
6	0	0	0	0	0	0	0	0	0
0	6	0	0	0	0	0	0	0	0
0	6	0	0	0	0	0	0	0	0
0	6	0	0	0	0	0	0	0	0
0	6	0	0	0	0	0	0	0	0
0	6	0	0	0	0	0	0	0	0
0	6	0	0	0	0	0	0	0	0
0	0	6	0	0	0	0	0	0	0
0	0	6	0	0	0	0	0	0	0
0	0	6	0	0	0	0	0	0	0
0	0	6	0	0	0	0	0	0	0
0	0	6	0	0	0	0	0	0	0
0	0	6	0	0	0	0	0	0	0
0	0	6	0	0	0	0	0	0	0
0	0	6	0	0	0	0	0	0	0
0	0	3	3	0	0	0	0	0	0
0	0	2	4	0	0	0	0	0	0
0	0	0	6	0	0	0	0	0	0
0	0	0	6	0	0	0	0	0	0
0	0	0	0	6	0	0	0	0	0
0	0	0	0	6	0	0	0	0	0
0	0	0	0	6	0	0	0	0	0
0	0	0	0	6	0	0	0	0	0
0	0	0	0	6	0	0	0	0	0
0	0	0	0	6	0	0	0	0	0
0	0	0	0	0	6	0	0	0	0
0	0	0	0	0	6	0	0	0	0
0	0	0	0	0	6	0	0	0	0
0	0	0	0	0	6	0	0	0	0
0	0	0	0	0	6	0	0	0	0
0	0	0	0	0	6	0	0	0	0
0	0	0	0	0	6	0	0	0	0
0	0	0	0	0	6	0	0	0	0
0	0	0	0	0	6	0	0	0	0
0	0	0	0	0	0	0	6	0	0
0	0	0	0	0	0	0	6	0	0
0	0	0	0	0	0	0	6	0	0
0	0	0	0	0	0	0	4	2	0
0	0	0	0	0	0	0	0	0	0
0	0	0	0	0	0	0	0	0	0
0	0	0	0	0	0	0	0	0	0
0	0	0	0	0	0	0	0	0	0
0	0	0	0	0	0	0	0	0	0
0	0	0	0	0	0	0	0	0	0
0	0	0	0	0	0	0	0	0	0
0	0	0	0	0	0	0	0	0	0
0	0	0	0	0	0	0	0	0	0
0	0	0	0	0	0	0	0	0	0

0	0	0	0	0	0	0	0	0	0
0	0	0	0	0	0	0	0	0	0
0	0	0	0	0	0	0	0	0	0
0	0	0	0	0	0	0	0	0	0
0	0	0	0	0	0	0	0	0	0
0	0	0	0	0	0	0	0	0	0
0	0	0	0	0	0	0	0	0	0
0	0	0	0	0	0	0	0	0	0
0	0	0	0	0	0	0	0	0	0
0	0	0	0	0	0	0	0	0	0
0	0	0	0	0	0	0	0	0	0
0	0	0	0	0	0	0	0	0	0
0	0	0	0	0	0	0	0	0	0
0	0	0	0	0	0	0	0	0	0
0	0	0	0	0	0	0	0	0	0
0	0	0	0	0	0	0	0	0	0
0	0	0	0	0	0	0	0	0	0
0	0	0	0	0	0	0	0	0	0
0	0	0	0	0	0	0	0	0	0
0	0	0	0	0	0	0	0	0	0
0	0	0	0	0	0	0	0	2	0
0	0	0	0	0	0	0	0	0	0
0	0	0	0	0	0	0	0	0	0
0	0	0	0	0	0	0	0	0	0
0	0	0	0	0	0	0	0	0	0
5	0	0	0	0	0	0	0	0	0
0	5	0	0	0	0	0	0	0	0
0	5	0	0	0	0	0	0	0	0
0	5	0	0	0	0	0	0	0	0
0	5	0	0	0	0	0	0	0	0
0	5	0	0	0	0	0	0	0	0
0	5	0	0	0	0	0	0	0	0
0	5	0	0	0	0	0	0	0	0
0	5	0	0	0	0	0	0	0	0
0	5	0	0	0	0	0	0	0	0
0	5	0	0	0	0	0	0	0	0
0	5	0	0	0	0	0	0	0	0
0	0	5	0	0	0	0	0	0	0
0	0	5	0	0	0	0	0	0	0
0	0	5	0	0	0	0	0	0	0
0	0	5	0	0	0	0	0	0	0
0	0	0	5	0	0	0	0	0	0
0	0	0	5	0	0	0	0	0	0
0	0	0	5	0	0	0	0	0	0
0	0	0	5	0	0	0	0	0	0
0	0	0	5	0	0	0	0	0	0
0	0	0	5	0	0	0	0	0	0
0	0	0	5	0	0	0	0	0	0
0	0	0	5	0	0	0	0	0	0
0	0	0	5	0	0	0	0	0	0

0	0	0	0	5	0	0	0	0	0
0	0	0	0	5	0	0	0	0	0
0	0	0	0	0	5	0	0	0	0
0	0	0	0	0	5	0	0	0	0
0	0	0	0	0	5	0	0	0	0
0	0	0	0	0	5	0	0	0	0
0	0	0	0	0	5	0	0	0	0
0	0	0	0	0	5	0	0	0	0
0	0	0	0	0	5	0	0	0	0
0	0	0	0	0	5	0	0	0	0
0	0	0	0	0	5	0	0	0	0
0	0	0	0	0	5	0	0	0	0
0	0	0	0	0	2	0	3	0	0
0	0	0	0	0	0	0	5	0	0
0	0	0	0	0	0	0	5	0	0
0	0	0	0	0	0	0	5	0	0
0	0	0	0	0	0	0	5	0	0
0	0	0	0	0	0	0	5	0	0
0	0	0	0	0	0	0	5	0	0
0	0	0	0	0	0	0	0	0	5
0	0	0	0	0	0	0	0	0	0
0	0	0	0	0	0	0	0	0	0
0	0	0	0	0	0	0	0	0	0
0	0	0	0	0	0	0	0	0	0
0	0	0	0	0	0	0	0	0	0
0	0	0	0	0	0	0	0	0	0
0	0	0	0	0	0	0	0	0	0
0	0	0	0	0	0	0	0	0	0
0	0	0	0	0	0	0	0	0	0
0	0	0	0	0	0	0	0	0	0
0	0	0	0	0	0	0	0	0	0
0	0	0	0	0	0	0	0	0	0
0	0	0	0	0	0	0	0	0	0
0	0	0	0	0	0	0	0	0	0
0	0	0	0	0	0	0	0	0	0
0	0	0	0	0	0	0	0	0	0
0	0	0	0	0	0	0	0	0	0
0	0	0	0	0	0	0	0	0	0
0	0	0	0	0	0	0	0	0	0
0	0	0	0	0	0	0	0	0	0
0	0	0	0	2	0	0	0	0	0
0	0	0	0	0	0	0	0	0	0
0	0	0	0	0	0	0	0	0	0
0	0	0	0	0	0	0	0	0	0
0	0	0	0	0	0	0	0	0	0
0	0	0	0	0	0	0	0	0	0
0	0	0	0	0	0	0	0	0	0
0	0	0	0	0	0	0	0	0	0
0	0	0	0	0	0	0	0	0	0
0	0	0	0	0	0	0	0	0	0

0	0	0	0	0	0	0	0	0	0
0	0	0	0	0	0	0	0	0	0
0	0	0	0	0	0	0	0	0	0
0	0	0	0	0	0	0	0	0	0
4	0	0	0	0	0	0	0	0	0
4	0	0	0	0	0	0	0	0	0
4	0	0	0	0	0	0	0	0	0
4	0	0	0	0	0	0	0	0	0
4	0	0	0	0	0	0	0	0	0
4	0	0	0	0	0	0	0	0	0
4	0	0	0	0	0	0	0	0	0
2	0	0	0	0	2	0	0	0	0
0	4	0	0	0	0	0	0	0	0
0	4	0	0	0	0	0	0	0	0
0	4	0	0	0	0	0	0	0	0
0	4	0	0	0	0	0	0	0	0
0	4	0	0	0	0	0	0	0	0
0	0	4	0	0	0	0	0	0	0
0	0	4	0	0	0	0	0	0	0
0	0	4	0	0	0	0	0	0	0
0	0	4	0	0	0	0	0	0	0
0	0	2	0	0	0	0	0	0	2
0	0	0	4	0	0	0	0	0	0
0	0	0	4	0	0	0	0	0	0
0	0	0	4	0	0	0	0	0	0
0	0	0	4	0	0	0	0	0	0
0	0	0	0	4	0	0	0	0	0
0	0	0	0	4	0	0	0	0	0
0	0	0	0	4	0	0	0	0	0
0	0	0	0	4	0	0	0	0	0
0	0	0	0	0	4	0	0	0	0
0	0	0	0	0	4	0	0	0	0
0	0	0	0	0	4	0	0	0	0
0	0	0	0	0	4	0	0	0	0
0	0	0	0	0	4	0	0	0	0
0	0	0	0	0	4	0	0	0	0
0	0	0	0	0	4	0	0	0	0
0	0	0	0	0	4	0	0	0	0
0	0	0	0	0	4	0	0	0	0
0	0	0	0	0	4	0	0	0	0
0	0	0	0	0	4	0	0	0	0
0	0	0	0	0	4	0	0	0	0
0	0	0	0	0	4	0	0	0	0
0	0	0	0	0	0	2	2	0	0
0	0	0	0	0	0	0	4	0	0
0	0	0	0	0	0	0	4	0	0
0	0	0	0	0	0	0	0	4	0
0	0	0	0	0	0	0	0	4	0
0	0	0	0	0	0	0	0	4	0
0	0	0	0	0	0	0	0	4	0

0	0	0	0	0	0	0	0	0	4
0	0	0	0	0	0	0	0	0	4
0	0	0	0	0	0	0	0	0	4
0	0	0	0	0	0	0	0	0	0
0	0	0	0	0	0	0	0	0	0
0	0	0	0	0	0	0	0	0	0
0	0	0	0	0	0	0	0	0	0
0	0	0	0	0	0	0	0	0	0
0	0	0	0	0	0	0	0	0	0
0	0	0	0	0	0	0	0	0	0
0	0	0	0	0	0	0	0	0	0
0	0	0	0	0	0	0	0	0	0
0	0	0	0	0	0	0	0	0	0
0	0	0	0	0	0	0	0	0	0
0	0	0	0	0	0	0	0	0	0
0	0	0	0	0	0	0	0	0	0
0	0	0	0	0	0	0	0	0	0
0	0	0	0	0	0	0	0	0	0
0	0	0	0	0	0	0	0	0	0
0	0	0	0	0	0	0	0	0	0
0	0	0	0	0	0	0	0	0	0
0	0	0	0	0	0	0	0	0	0
0	0	0	0	0	0	0	0	0	0
0	0	0	0	0	0	0	0	0	0
0	0	0	0	0	0	0	0	0	0
0	0	0	0	0	0	0	0	0	0
0	0	0	0	0	0	0	0	0	0
0	0	0	0	0	0	0	0	0	0
0	0	0	0	0	0	0	0	0	0
0	0	0	0	0	0	0	0	0	0
0	0	0	0	0	0	0	0	0	0
0	0	0	0	0	0	0	0	0	0
0	0	0	0	0	0	0	0	0	0
0	0	0	0	0	0	0	0	0	0
0	0	0	0	0	0	0	0	0	0
0	0	0	0	0	0	0	0	0	0
0	0	0	0	0	0	0	0	0	0
0	0	0	0	0	0	0	0	0	0
0	0	0	0	0	0	0	0	0	0
0	0	0	0	0	0	0	0	0	0
0	0	0	0	0	0	0	0	0	0
0	0	0	0	0	0	0	0	0	0
0	0	0	0	0	0	0	0	0	0
0	0	0	0	0	0	0	0	0	0
0	0	0	0	0	0	0	0	0	0
0	0	0	0	0	0	0	0	0	0
0	0	0	0	0	0	0	0	0	0
0	0	0	0	0	0	0	0	0	0
0	0	0	0	0	0	0	0	0	0
0	0	0	0	0	0	0	0	0	0

0	0	0	0	0	0	0	0	0	0
0	0	0	0	0	0	0	0	0	0
0	0	0	0	0	0	0	0	0	0
0	0	0	0	0	0	0	0	0	0
0	0	0	0	0	0	0	0	0	0
0	0	0	0	0	0	0	0	0	0
0	0	0	0	0	0	0	0	0	0
0	0	0	0	0	0	0	0	0	0
0	0	0	0	0	0	0	0	0	0
0	0	0	0	0	0	0	0	0	0
0	0	0	0	0	0	0	0	0	0
0	0	0	0	0	0	0	0	0	0
3	0	0	0	0	0	0	0	0	0
3	0	0	0	0	0	0	0	0	0
3	0	0	0	0	0	0	0	0	0
3	0	0	0	0	0	0	0	0	0
3	0	0	0	0	0	0	0	0	0
0	3	0	0	0	0	0	0	0	0
0	3	0	0	0	0	0	0	0	0
0	3	0	0	0	0	0	0	0	0
0	3	0	0	0	0	0	0	0	0
0	3	0	0	0	0	0	0	0	0
0	3	0	0	0	0	0	0	0	0
0	0	3	0	0	0	0	0	0	0
0	0	3	0	0	0	0	0	0	0
0	0	3	0	0	0	0	0	0	0
0	0	3	0	0	0	0	0	0	0
0	0	0	3	0	0	0	0	0	0
0	0	0	3	0	0	0	0	0	0
0	0	0	3	0	0	0	0	0	0
0	0	0	3	0	0	0	0	0	0
0	0	0	3	0	0	0	0	0	0
0	0	0	3	0	0	0	0	0	0
0	0	0	3	0	0	0	0	0	0
0	0	0	3	0	0	0	0	0	0
0	0	0	3	0	0	0	0	0	0
0	0	0	0	3	0	0	0	0	0
0	0	0	0	3	0	0	0	0	0
0	0	0	0	3	0	0	0	0	0
0	0	0	0	3	0	0	0	0	0
0	0	0	0	3	0	0	0	0	0
0	0	0	0	3	0	0	0	0	0
0	0	0	0	3	0	0	0	0	0
0	0	0	0	0	3	0	0	0	0
0	0	0	0	0	3	0	0	0	0
0	0	0	0	0	3	0	0	0	0
0	0	0	0	0	3	0	0	0	0
0	0	0	0	0	3	0	0	0	0
0	0	0	0	0	3	0	0	0	0
0	0	0	0	0	3	0	0	0	0

0	0	0	0	0	3	0	0	0	0
0	0	0	0	0	3	0	0	0	0
0	0	0	0	0	3	0	0	0	0
0	0	0	0	0	3	0	0	0	0
0	0	0	0	0	3	0	0	0	0
0	0	0	0	0	3	0	0	0	0
0	0	0	0	0	3	0	0	0	0
0	0	0	0	0	3	0	0	0	0
0	0	0	0	0	3	0	0	0	0
0	0	0	0	0	3	0	0	0	0
0	0	0	0	0	0	3	0	0	0
0	0	0	0	0	0	3	0	0	0
0	0	0	0	0	0	0	3	0	0
0	0	0	0	0	0	0	3	0	0
0	0	0	0	0	0	0	3	0	0
0	0	0	0	0	0	0	3	0	0
0	0	0	0	0	0	0	3	0	0
0	0	0	0	0	0	0	0	3	0
0	0	0	0	0	0	0	0	3	0
0	0	0	0	0	0	0	0	3	0
0	0	0	0	0	0	0	0	0	3
0	0	0	0	0	0	0	0	0	3
0	0	0	0	0	0	0	0	0	0
0	0	0	0	0	0	0	0	0	0
0	0	0	0	0	0	0	0	0	0
0	0	0	0	0	0	0	0	0	0
0	0	0	0	0	0	0	0	0	0
0	0	0	0	0	0	0	0	0	0
0	0	0	0	0	0	0	0	0	0
0	0	0	0	0	0	0	0	0	0
0	0	0	0	0	0	0	0	0	0
0	0	0	0	0	0	0	0	0	0
0	0	0	0	0	0	0	0	0	0
0	0	0	0	0	0	0	0	0	0
0	0	0	0	0	0	0	0	0	0
0	0	0	0	0	0	0	0	0	0
0	0	0	0	0	0	0	0	0	0
0	0	0	0	0	0	0	0	0	0
0	0	0	0	0	0	0	0	0	0
0	0	0	0	0	0	0	0	0	0
0	0	0	0	0	0	0	0	0	0
0	0	0	0	0	0	0	0	0	0
0	0	0	0	0	0	0	0	0	0
0	0	0	0	0	0	0	0	0	0
0	0	0	0	0	0	0	0	0	0
0	0	0	0	0	0	0	0	0	0
0	0	0	0	0	0	0	0	0	0
0	0	0	0	0	0	0	0	0	0
0	0	0	0	0	0	0	0	0	0
0	0	0	0	0	0	0	0	0	0

0	0	0	0	0	0	0	0	0	0
0	0	0	0	0	0	0	0	0	0
0	0	0	0	0	0	0	0	0	0
0	0	0	0	0	0	0	0	0	0
0	0	0	0	0	0	0	0	0	0
0	0	0	0	0	0	0	0	0	0
0	0	0	0	0	0	0	0	0	0
0	0	0	0	0	0	0	0	0	0
0	0	0	0	0	0	0	0	0	0
0	0	0	0	0	0	0	0	0	0
0	0	0	0	0	0	0	0	0	0
0	0	0	0	0	0	0	0	0	0
0	0	0	0	0	0	0	0	0	0
0	0	0	0	0	0	0	0	0	0
0	0	0	0	0	0	0	0	0	0
0	0	0	0	0	0	0	0	0	0
0	0	0	0	0	0	0	0	0	0
0	0	0	0	0	0	0	0	0	0
0	0	0	0	0	0	0	0	0	0
0	0	0	0	0	0	0	0	0	0
0	0	0	0	0	0	0	0	0	0
0	0	0	0	0	0	0	0	0	0
0	0	0	0	0	0	0	0	0	0
0	0	0	0	0	0	0	0	0	0
0	0	0	0	0	0	0	0	0	0
0	0	0	0	0	0	0	0	0	0
0	0	0	0	0	0	0	0	0	0
0	0	0	0	0	0	0	0	0	0
0	0	0	0	0	0	0	0	0	0
0	0	0	0	0	0	0	0	0	0
0	0	0	0	0	0	0	0	0	0
0	0	0	0	0	0	0	0	0	0
0	0	0	0	0	0	0	0	0	0
0	0	0	0	0	0	0	0	0	0
0	0	0	0	0	0	0	0	0	0
0	0	0	0	0	0	0	0	0	0
0	0	0	0	0	0	0	0	0	0
0	0	0	0	0	0	0	0	0	0
0	0	0	0	0	0	0	0	0	0
0	0	0	0	0	0	0	0	0	0
0	0	0	0	0	0	0	0	0	0
0	0	0	0	0	0	0	0	0	0
0	0	0	0	0	0	0	0	0	0
0	0	0	0	0	0	0	0	0	0
0	0	0	0	0	0	0	0	0	0
0	0	0	0	0	0	0	0	0	0
0	0	0	0	0	0	0	0	0	0
0	0	0	0	0	0	0	0	0	0
0	0	0	0	0	0	0	0	0	0
0	0	0	0	0	0	0	0	0	0
0	0	0	0	0	0	0	0	0	0

2	0	0	0	0	0	0	0	0	0
2	0	0	0	0	0	0	0	0	0
0	2	0	0	0	0	0	0	0	0
0	2	0	0	0	0	0	0	0	0
0	2	0	0	0	0	0	0	0	0
0	2	0	0	0	0	0	0	0	0
0	2	0	0	0	0	0	0	0	0
0	2	0	0	0	0	0	0	0	0
0	2	0	0	0	0	0	0	0	0
0	2	0	0	0	0	0	0	0	0
0	2	0	0	0	0	0	0	0	0
0	2	0	0	0	0	0	0	0	0
0	2	0	0	0	0	0	0	0	0
0	0	2	0	0	0	0	0	0	0
0	0	2	0	0	0	0	0	0	0
0	0	2	0	0	0	0	0	0	0
0	0	2	0	0	0	0	0	0	0
0	0	2	0	0	0	0	0	0	0
0	0	0	2	0	0	0	0	0	0
0	0	0	2	0	0	0	0	0	0
0	0	0	2	0	0	0	0	0	0
0	0	0	2	0	0	0	0	0	0
0	0	0	2	0	0	0	0	0	0
0	0	0	2	0	0	0	0	0	0
0	0	0	2	0	0	0	0	0	0
0	0	0	2	0	0	0	0	0	0
0	0	0	2	0	0	0	0	0	0
0	0	0	2	0	0	0	0	0	0
0	0	0	0	2	0	0	0	0	0
0	0	0	0	2	0	0	0	0	0
0	0	0	0	2	0	0	0	0	0
0	0	0	0	0	2	0	0	0	0
0	0	0	0	0	2	0	0	0	0
0	0	0	0	0	2	0	0	0	0
0	0	0	0	0	2	0	0	0	0
0	0	0	0	0	2	0	0	0	0
0	0	0	0	0	2	0	0	0	0
0	0	0	0	0	2	0	0	0	0
0	0	0	0	0	2	0	0	0	0
0	0	0	0	0	2	0	0	0	0
0	0	0	0	0	2	0	0	0	0
0	0	0	0	0	2	0	0	0	0
0	0	0	0	0	2	0	0	0	0
0	0	0	0	0	2	0	0	0	0
0	0	0	0	0	2	0	0	0	0
0	0	0	0	0	2	0	0	0	0
0	0	0	0	0	2	0	0	0	0
0	0	0	0	0	2	0	0	0	0
0	0	0	0	0	2	0	0	0	0
0	0	0	0	0	0	2	0	0	0

0	0	0	0	0	0	2	0	0	0
0	0	0	0	0	0	2	0	0	0
0	0	0	0	0	0	2	0	0	0
0	0	0	0	0	0	2	0	0	0
0	0	0	0	0	0	2	0	0	0
0	0	0	0	0	0	0	2	0	0
0	0	0	0	0	0	0	2	0	0
0	0	0	0	0	0	0	0	2	0
0	0	0	0	0	0	0	0	2	0
0	0	0	0	0	0	0	0	2	0
0	0	0	0	0	0	0	0	2	0
0	0	0	0	0	0	0	0	2	0
0	0	0	0	0	0	0	0	2	0
0	0	0	0	0	0	0	0	2	0
0	0	0	0	0	0	0	0	2	0
0	0	0	0	0	0	0	0	0	2
0	0	0	0	0	0	0	0	0	2
0	0	0	0	0	0	0	0	0	0
0	0	0	0	0	0	0	0	0	0
0	0	0	0	0	0	0	0	0	0
0	0	0	0	0	0	0	0	0	0
0	0	0	0	0	0	0	0	0	0
0	0	0	0	0	0	0	0	0	0
0	0	0	0	0	0	0	0	0	0
0	0	0	0	0	0	0	0	0	0
0	0	0	0	0	0	0	0	0	0
0	0	0	0	0	0	0	0	0	0
0	0	0	0	0	0	0	0	0	0
0	0	0	0	0	0	0	0	0	0
0	0	0	0	0	0	0	0	0	0
0	0	0	1	0	0	0	0	0	0
0	0	0	1	0	0	0	0	0	0
0	0	0	0	0	1	0	0	0	0
0	0	0	0	0	1	0	0	0	0
0	65	100	9962	78	216	72	60	69	20
0	45	27	0	6833	1094	46	80	31	0
0	0	0	2841	0	0	0	0	0	21
0	47	37	853	1662	78	27	21	41	7
0	27	27	355	1287	59	20	17	28	8
29	65	0	712	101	43	0	0	0	0
0	0	0	839	0	0	0	0	0	0
0	14	10	554	0	41	10	10	16	0
0	0	0	0	0	0	0	0	0	0
0	242	151	22	0	88	0	21	6	0
0	7	6	58	447	0	5	0	4	0
42	0	4	206	103	85	0	0	0	0
12	4	0	329	47	93	0	2	0	0
20	0	0	131	37	264	0	0	0	0
0	0	0	419	0	0	0	0	0	0
0	0	0	246	162	0	0	0	0	0

0	0	0	401	0	0	0	0	0	0
4	15	25	0	20	51	6	10	4	0
12	0	0	128	36	182	0	0	0	0
0	13	16	49	17	140	6	5	0	2
4	21	0	114	65	119	0	0	0	0
0	0	0	291	22	0	0	0	0	0
0	0	0	0	0	0	0	298	0	0
0	0	0	0	0	0	0	0	0	0
0	57	42	44	9	18	0	0	0	0
0	3	0	213	0	16	0	0	0	0
9	34	14	132	0	15	0	0	0	0
0	0	0	183	0	16	0	0	0	0
187	0	0	0	0	11	0	0	0	0
0	16	10	62	26	80	0	0	0	0
0	0	0	159	0	30	0	0	0	0
0	0	0	0	0	0	0	0	0	0
0	0	0	150	0	0	0	0	0	0
0	0	0	46	0	96	0	0	0	0
0	0	0	131	0	0	0	0	0	0
0	0	0	0	26	63	0	0	0	0
0	0	103	0	0	0	0	0	0	0
0	0	0	78	0	0	0	0	0	0
2	0	0	42	0	50	0	0	0	0
0	0	0	90	0	0	0	0	0	0
0	0	0	19	14	47	0	0	0	0
0	0	0	83	0	0	0	0	0	0
0	0	0	76	0	0	0	0	0	0
16	0	0	59	0	0	0	0	0	0
0	0	0	73	0	0	0	0	0	0
0	0	0	0	71	0	0	0	0	0
0	0	0	35	0	33	0	0	0	0
35	10	22	0	0	0	0	0	0	0
0	0	0	0	0	0	0	0	0	0
0	0	0	11	0	5	0	0	0	0
0	0	0	0	62	0	0	0	0	0
0	0	0	0	0	0	0	0	0	0
0	0	0	52	0	5	0	0	0	0
0	0	0	56	0	0	0	0	0	0
0	0	0	54	0	0	0	0	0	0
0	0	0	11	0	0	15	0	26	0
0	0	0	17	0	34	0	0	0	0
0	0	0	0	0	0	0	0	0	0
0	0	0	50	0	0	0	0	0	0
2	0	7	15	5	20	0	0	0	0
0	0	0	49	0	0	0	0	0	0
0	0	0	49	0	0	0	0	0	0
0	0	0	0	0	0	0	0	0	0
0	0	0	0	43	0	0	0	0	0
0	0	0	0	0	0	0	0	0	0
0	0	0	0	0	41	0	0	0	0

4	0	3	29	4	0	0	0	0	0
0	0	0	40	0	0	0	0	0	0
0	0	0	39	0	0	0	0	0	0
0	0	0	0	16	23	0	0	0	0
0	0	0	38	0	0	0	0	0	0
0	0	0	38	0	0	0	0	0	0
0	0	0	37	0	0	0	0	0	0
0	0	0	0	0	37	0	0	0	0
7	20	9	0	0	0	0	0	0	0
0	0	0	36	0	0	0	0	0	0
0	0	0	15	0	19	0	0	0	0
2	0	0	31	0	0	0	0	0	0
0	0	0	33	0	0	0	0	0	0
0	8	0	24	0	0	0	0	0	0
0	0	0	0	29	0	0	0	3	0
0	28	0	0	0	0	0	0	0	0
0	0	0	31	0	0	0	0	0	0
0	0	0	0	0	0	0	0	0	0
10	20	0	0	0	0	0	0	0	0
0	0	0	30	0	0	0	0	0	0
0	0	0	30	0	0	0	0	0	0
0	0	0	0	0	0	0	0	0	0
0	0	0	0	0	0	0	0	0	0
0	0	0	29	0	0	0	0	0	0
0	0	0	29	0	0	0	0	0	0
0	0	0	26	0	3	0	0	0	0
0	0	0	0	26	0	0	0	0	0
3	0	0	0	25	0	0	0	0	0
0	0	0	0	28	0	0	0	0	0
0	0	0	0	0	0	0	0	0	0
27	0	0	0	0	0	0	0	0	0
0	0	0	27	0	0	0	0	0	0
0	0	0	27	0	0	0	0	0	0
0	0	0	27	0	0	0	0	0	0
0	0	0	16	0	5	4	0	0	0
0	12	0	12	2	0	0	0	0	0
0	0	0	0	0	0	0	0	0	0
0	0	0	0	14	0	0	0	0	0
0	0	0	0	3	19	0	0	0	0
0	0	0	0	0	0	0	0	0	0
0	0	0	0	0	0	0	0	0	0
0	0	0	0	0	0	0	0	0	0
0	24	0	0	0	0	0	0	0	0
0	0	24	0	0	0	0	0	0	0
0	0	0	24	0	0	0	0	0	0
0	0	0	24	0	0	0	0	0	0
0	0	0	0	0	24	0	0	0	0
0	0	0	0	0	24	0	0	0	0
0	0	0	0	0	23	0	0	0	0
0	0	0	0	0	0	0	0	0	0

0	0	0	0	0	0	0	0	0	0
0	0	0	0	0	0	0	0	0	0
0	0	0	0	0	0	0	0	0	0
0	0	0	0	18	3	0	0	0	0
0	0	0	0	0	0	0	0	0	0
0	0	0	20	0	0	0	0	0	0
0	0	0	20	0	0	0	0	0	0
0	0	0	0	0	0	0	0	0	0
0	0	0	0	0	0	0	0	0	0
0	0	0	0	0	0	0	0	0	0
0	0	0	19	0	0	0	0	0	0
0	0	0	19	0	0	0	0	0	0
0	0	0	0	0	19	0	0	0	0
0	0	0	0	18	0	0	0	0	0
0	0	0	0	0	18	0	0	0	0
0	0	0	0	0	0	0	0	0	0
0	0	0	0	0	0	0	0	0	0
11	0	0	0	0	6	0	0	0	0
0	0	17	0	0	0	0	0	0	0
0	0	0	17	0	0	0	0	0	0
0	0	0	17	0	0	0	0	0	0
0	0	0	17	0	0	0	0	0	0
0	0	0	0	17	0	0	0	0	0
0	0	0	0	10	2	5	0	0	0
0	0	0	0	0	17	0	0	0	0
0	0	16	0	0	0	0	0	0	0
0	0	0	16	0	0	0	0	0	0
0	0	0	10	6	0	0	0	0	0
0	0	0	0	16	0	0	0	0	0
0	0	0	0	0	16	0	0	0	0
0	0	0	0	0	0	0	0	0	0
0	0	0	15	0	0	0	0	0	0
0	0	0	15	0	0	0	0	0	0
0	14	0	0	0	0	0	0	0	0
0	0	0	14	0	0	0	0	0	0
0	0	0	0	0	0	0	0	0	0
0	3	0	0	8	0	0	0	0	0
0	0	0	0	0	7	0	0	0	0
0	13	0	0	0	0	0	0	0	0
0	0	13	0	0	0	0	0	0	0
0	0	9	0	0	4	0	0	0	0
0	0	0	13	0	0	0	0	0	0
0	0	0	13	0	0	0	0	0	0
0	0	0	13	0	0	0	0	0	0
0	0	0	0	9	0	0	0	0	0
0	0	0	0	0	13	0	0	0	0
0	0	0	0	0	13	0	0	0	0
0	0	0	0	0	13	0	0	0	0
0	0	0	0	0	0	0	0	0	0
0	12	0	0	0	0	0	0	0	0

0	0	0	12	0	0	0	0	0	0
0	0	0	12	0	0	0	0	0	0
0	0	0	0	0	0	0	0	0	0
0	0	0	0	0	0	0	0	0	0
0	0	0	0	0	0	0	0	0	0
11	0	0	0	0	0	0	0	0	0
0	9	0	0	2	0	0	0	0	0
0	0	11	0	0	0	0	0	0	0
0	0	0	11	0	0	0	0	0	0
0	0	0	11	0	0	0	0	0	0
0	0	0	0	11	0	0	0	0	0
0	0	0	0	11	0	0	0	0	0
0	0	0	0	0	0	0	0	0	0
0	10	0	0	0	0	0	0	0	0
0	7	3	0	0	0	0	0	0	0
0	0	0	10	0	0	0	0	0	0
0	0	0	0	4	6	0	0	0	0
0	0	0	0	0	10	0	0	0	0
0	0	0	0	0	0	0	10	0	0
0	0	0	0	0	0	0	0	0	0
0	0	0	0	0	0	0	0	0	0
0	9	0	0	0	0	0	0	0	0
0	0	9	0	0	0	0	0	0	0
0	0	0	6	0	3	0	0	0	0
0	0	0	0	9	0	0	0	0	0
0	0	0	0	9	0	0	0	0	0
0	0	0	0	0	9	0	0	0	0
0	0	0	0	0	9	0	0	0	0
0	0	0	0	0	0	0	0	0	0
0	8	0	0	0	0	0	0	0	0
0	8	0	0	0	0	0	0	0	0
0	8	0	0	0	0	0	0	0	0
0	0	8	0	0	0	0	0	0	0
0	0	0	8	0	0	0	0	0	0
0	0	0	0	8	0	0	0	0	0
0	0	0	0	0	0	0	0	0	0
0	0	0	0	0	0	0	0	0	0
0	0	0	0	0	0	0	0	0	0
0	0	0	0	0	0	0	0	0	0
0	0	0	0	0	0	0	0	0	0
7	0	0	0	0	0	0	0	0	0
0	7	0	0	0	0	0	0	0	0
0	7	0	0	0	0	0	0	0	0
0	0	7	0	0	0	0	0	0	0
0	0	0	0	0	0	0	0	0	0
0	0	0	0	0	0	0	0	0	0
0	0	0	0	0	0	0	0	0	0
0	0	0	0	0	0	0	0	0	0
0	0	0	0	0	0	0	0	0	0
3	0	0	0	0	0	0	0	0	0

0	0	0	0	0	0	0	0	0	0
0	0	0	0	0	0	0	0	0	0
0	0	0	0	0	0	0	0	0	0
0	0	0	0	0	0	0	0	0	0
0	6	0	0	0	0	0	0	0	0
0	6	0	0	0	0	0	0	0	0
0	0	6	0	0	0	0	0	0	0
0	0	0	0	6	0	0	0	0	0
0	0	0	0	6	0	0	0	0	0
0	0	0	0	0	6	0	0	0	0
0	0	0	0	0	0	0	6	0	0
0	0	0	0	0	0	0	0	0	0
0	0	0	0	0	0	0	0	0	0
0	0	0	0	0	0	0	0	0	0
0	0	0	0	0	0	0	0	0	0
0	0	0	0	0	0	0	0	0	0
0	5	0	0	0	0	0	0	0	0
0	5	0	0	0	0	0	0	0	0
0	5	0	0	0	0	0	0	0	0
0	0	5	0	0	0	0	0	0	0
0	0	5	0	0	0	0	0	0	0
0	0	5	0	0	0	0	0	0	0
0	0	5	0	0	0	0	0	0	0
0	0	0	0	0	5	0	0	0	0
0	0	0	0	0	5	0	0	0	0
0	0	0	0	0	5	0	0	0	0
0	0	0	0	0	5	0	0	0	0
0	0	0	0	0	5	0	0	0	0
0	0	0	0	0	0	0	5	0	0
0	0	0	0	0	0	0	0	0	0
0	0	0	0	0	0	0	0	0	0
0	0	0	0	0	0	0	0	0	0
0	0	0	0	0	0	0	0	0	0
0	0	0	0	0	0	0	0	0	0
0	0	0	0	0	0	0	0	0	0
0	0	0	0	0	0	0	0	0	0
0	0	0	0	0	0	0	0	0	0
0	0	0	0	0	0	0	0	0	0
4	0	0	0	0	0	0	0	0	0
4	0	0	0	0	0	0	0	0	0
0	4	0	0	0	0	0	0	0	0
0	4	0	0	0	0	0	0	0	0
0	2	2	0	0	0	0	0	0	0
0	0	4	0	0	0	0	0	0	0
0	0	4	0	0	0	0	0	0	0
0	0	4	0	0	0	0	0	0	0
0	0	0	4	0	0	0	0	0	0
0	0	0	4	0	0	0	0	0	0
0	0	0	0	4	0	0	0	0	0
0	0	0	0	4	0	0	0	0	0

0	0	0	0	4	0	0	0	0	0
0	0	0	0	0	4	0	0	0	0
0	0	0	0	0	4	0	0	0	0
0	0	0	0	0	0	0	0	0	0
0	0	0	0	0	0	0	0	0	0
0	0	0	0	0	0	0	0	0	0
0	0	0	0	0	0	0	0	0	0
0	0	0	0	0	0	0	0	0	0
0	0	0	0	0	0	0	0	0	0
0	0	0	0	0	0	0	0	0	0
0	0	0	0	0	0	0	0	0	0
0	0	0	0	0	0	0	0	0	0
0	0	0	0	0	0	0	0	0	0
0	0	0	0	0	0	0	0	0	0
0	0	0	0	0	0	0	0	0	0
0	0	0	0	0	0	0	0	0	0
0	0	0	0	0	0	0	0	0	0
3	0	0	0	0	0	0	0	0	0
3	0	0	0	0	0	0	0	0	0
3	0	0	0	0	0	0	0	0	0
0	3	0	0	0	0	0	0	0	0
0	3	0	0	0	0	0	0	0	0
0	0	3	0	0	0	0	0	0	0
0	0	3	0	0	0	0	0	0	0
0	0	3	0	0	0	0	0	0	0
0	0	3	0	0	0	0	0	0	0
0	0	0	3	0	0	0	0	0	0
0	0	0	3	0	0	0	0	0	0
0	0	0	0	3	0	0	0	0	0
0	0	0	0	3	0	0	0	0	0
0	0	0	0	0	3	0	0	0	0
0	0	0	0	0	0	0	3	0	0
0	0	0	0	0	0	0	0	3	0
0	0	0	0	0	0	0	0	0	0
0	0	0	0	0	0	0	0	0	0
0	0	0	0	0	0	0	0	0	0
0	0	0	0	0	0	0	0	0	0
0	0	0	0	0	0	0	0	0	0
0	0	0	0	0	0	0	0	0	0
0	0	0	0	0	0	0	0	0	0
0	0	0	0	0	0	0	0	0	0
0	0	0	0	0	0	0	0	0	0
0	0	0	0	0	0	0	0	0	0
0	0	0	0	0	0	0	0	0	0
0	2	0	0	0	0	0	0	0	0
0	2	0	0	0	0	0	0	0	0
0	2	0	0	0	0	0	0	0	0
0	0	0	2	0	0	0	0	0	0
0	0	0	0	0	2	0	0	0	0
0	0	0	0	0	2	0	0	0	0
0	0	0	0	0	2	0	0	0	0

0	0	0	0	0	2	0	0	0	0
0	0	0	0	0	0	2	0	0	0
0	0	0	0	0	0	0	0	0	2
0	0	0	0	0	0	0	0	0	0
0	0	0	0	0	0	0	0	0	0
0	0	0	0	0	0	0	0	0	0
0	0	0	0	0	0	0	0	0	0
0	0	0	0	0	0	0	0	0	0
0	0	0	0	0	0	0	0	0	0
0	20	0	0	0	0	0	0	0	0
0	0	0	0	0	18	0	0	0	0
0	0	0	0	0	0	0	0	0	0
0	0	0	0	0	0	0	0	0	0
0	0	0	0	0	0	0	0	0	0
0	0	0	0	0	0	0	0	0	0
0	0	0	0	0	0	0	0	0	0
0	6	0	0	0	0	0	0	0	0
0	0	0	0	0	0	0	0	0	0
0	0	0	0	0	0	0	0	0	0
0	0	0	0	0	0	0	0	0	0
0	0	0	0	0	0	0	0	0	0
0	0	0	0	0	0	0	0	0	0
0	0	0	2	0	0	0	0	0	0
0	0	0	0	0	2	0	0	0	0
8	1640	1228	1793	773	3031	107559	2699	89878	468
4535	38895	29132	32	20	217	43	144	19	14
65	0	0	0	0	657	0	0	0	0
0	0	0	0	0	0	0	0	0	0
0	0	0	0	0	0	0	0	0	0
0	254	168	204	115	358	2079	6666	7072	0
0	142	123	169	71	278	1466	188	13970	44
0	99	67	123	49	207	10291	145	1922	0
154	0	0	0	0	1212	0	0	0	0
0	0	0	0	0	0	0	0	0	0
4	59	49	43	21	66	5226	23	637	11
0	0	0	89	0	112	5003	0	509	0
0	53	43	74	26	108	387	47	4160	18
338	2412	1560	0	0	0	0	0	0	0
0	62	41	79	36	95	569	55	2491	25
0	0	0	0	0	0	3279	0	0	0
0	0	0	0	0	0	0	0	0	0
0	0	0	0	0	0	0	0	0	0
0	0	0	0	0	184	0	0	0	0
0	0	0	0	0	0	0	0	0	0
0	0	0	0	0	0	0	0	0	0
0	0	0	0	0	0	0	0	0	0
0	30	0	0	0	0	0	34	0	0
0	0	0	0	0	0	1890	115	447	0
118	1273	969	0	0	0	0	0	0	0
174	1332	797	0	0	0	0	0	0	0

0	0	0	0	0	0	0	0	0	0
0	0	0	0	0	0	0	0	0	0
0	0	0	0	0	0	0	0	1940	0
0	0	0	0	0	0	0	0	0	0
0	214	231	376	169	382	0	0	126	0
0	0	0	0	0	0	0	0	0	0
0	0	0	102	232	137	0	0	0	0
0	0	0	0	0	0	0	0	0	0
0	0	0	0	0	31	0	0	0	0
0	0	0	0	0	0	0	0	0	0
0	0	0	30	0	42	1189	0	116	0
0	0	0	0	0	0	0	0	0	0
0	0	0	0	0	33	0	0	0	0
0	0	0	0	0	0	0	0	0	0
0	0	0	0	0	0	0	0	0	0
0	0	0	0	0	0	0	0	0	0
0	0	0	0	0	0	0	0	0	0
0	0	0	0	0	55	0	0	0	0
0	0	0	0	0	0	0	0	0	0
0	0	0	17	0	43	749	0	116	0
0	0	0	0	0	0	0	0	0	0
0	0	0	0	0	0	96	0	815	0
0	0	0	0	0	0	0	0	831	0
0	7	7	7	4	751	0	4	0	0
0	0	0	0	0	0	672	0	96	0
0	0	0	0	0	29	0	0	0	0
0	10	7	140	540	17	10	6	11	0
0	0	0	0	0	0	738	0	0	0
38	46	8	0	0	8	0	22	0	0
0	6	2	222	307	107	5	5	9	0
0	0	0	0	0	0	0	0	0	0
0	221	113	11	25	58	11	40	11	0
0	0	0	0	0	0	0	0	660	0
0	0	0	0	0	13	0	0	0	0
0	295	336	0	0	0	0	0	0	0
0	379	203	0	0	0	0	0	0	0
0	297	279	0	0	0	0	0	0	0
36	0	0	0	0	0	0	0	0	0
0	0	0	0	0	0	0	0	0	0
0	334	169	0	0	0	0	0	0	0
0	0	0	0	0	0	0	0	0	0
0	0	0	0	0	0	0	0	0	0
14	0	65	7	0	0	0	0	0	0
0	140	96	0	0	0	0	53	51	38
0	294	172	0	0	0	0	0	0	0
0	0	0	0	0	0	0	0	0	0
0	0	0	0	0	0	453	0	0	0
0	0	0	0	0	0	6	0	0	0
311	69	28	0	0	0	0	0	0	0
0	0	0	0	0	0	0	0	0	0

0	0	0	0	0	0	0	0	0	0
0	0	0	0	0	0	0	0	0	0
0	226	168	0	0	0	0	0	0	0
0	0	0	29	0	0	0	0	0	0
0	0	0	0	0	0	0	0	0	0
0	0	0	0	0	0	0	0	390	0
0	0	0	0	0	0	0	0	0	0
0	0	0	0	0	0	0	0	0	0
0	0	0	0	0	0	0	0	0	0
0	0	0	319	0	0	0	0	0	0
0	0	0	0	0	0	0	0	0	0
0	0	0	0	0	0	0	0	0	0
0	0	0	0	0	0	0	0	360	0
0	0	0	0	0	0	0	0	0	0
0	0	0	0	0	0	0	0	350	0
0	0	0	0	0	0	296	0	46	0
0	0	0	0	0	0	0	0	0	0
0	0	0	0	0	318	0	0	0	0
0	0	0	0	0	0	0	0	0	0
0	0	0	0	0	0	0	0	0	0
0	0	0	0	0	0	316	0	0	0
0	0	0	0	0	0	0	0	313	0
0	0	0	0	0	0	0	0	0	0
181	92	30	0	0	0	0	0	0	0
173	77	23	0	0	19	0	0	4	0
0	0	0	0	0	0	0	0	0	0
0	0	0	0	0	0	0	0	288	0
0	0	0	0	0	0	200	0	87	0
0	0	0	0	0	0	0	0	0	0
0	0	0	0	0	0	0	0	0	0
0	0	0	0	0	0	0	0	0	0
0	0	0	0	0	0	0	0	0	0
0	0	0	0	0	0	0	0	0	0
0	0	0	0	0	0	0	0	0	0
0	0	0	0	0	0	0	0	0	0
0	0	0	0	0	0	0	0	260	0
0	0	0	0	0	0	250	0	0	0
0	0	0	0	0	0	0	0	247	0
0	0	0	0	0	0	0	0	0	0
0	0	0	0	0	0	0	0	0	0
0	0	0	0	0	0	0	0	0	0
0	0	0	0	0	0	0	0	0	0
0	231	0	0	0	0	0	0	0	0
0	13	0	0	0	0	0	0	0	0
0	0	0	0	0	0	0	0	0	0
0	0	0	0	0	28	125	0	63	0
0	0	0	0	0	0	0	0	0	0
0	0	0	0	0	0	0	0	214	0
0	0	0	0	0	0	0	0	0	0
0	0	0	69	0	11	0	0	0	0

0	0	0	0	0	0	0	0	0	0
0	0	0	0	0	0	0	0	0	0
0	0	0	0	0	0	0	0	188	0
0	0	0	0	0	0	0	0	0	0
0	0	0	0	0	0	0	0	0	0
0	0	0	0	0	0	0	0	0	0
0	0	0	0	0	0	0	0	0	0
0	0	0	0	0	0	0	0	0	0
0	0	0	0	0	12	0	0	0	0
0	168	0	0	0	0	0	0	0	0
0	0	0	0	0	0	0	0	167	0
0	0	0	0	0	0	0	0	0	0
105	47	13	0	0	0	0	0	0	0
0	0	0	0	0	0	0	0	0	0
0	0	0	0	0	0	0	0	0	0
2	0	0	0	0	5	0	0	0	0
0	0	0	0	0	0	0	0	0	0
0	0	0	0	0	0	0	159	0	0
0	0	0	0	0	0	0	0	0	0
0	0	0	0	0	0	153	0	0	0
0	0	0	0	0	0	0	0	0	0
0	0	0	0	0	0	0	0	0	0
0	0	0	0	0	0	0	0	0	0
0	0	0	0	0	0	0	0	0	0
4	0	0	3	0	43	0	77	0	0
0	0	0	0	0	0	0	0	0	0
0	0	0	0	0	0	0	0	0	0
0	0	0	0	0	0	0	0	0	0
0	0	0	0	0	0	0	0	0	0
0	0	0	0	0	0	0	0	0	0
0	0	0	0	0	0	0	0	0	0
0	0	0	0	0	0	0	0	0	0
0	0	0	0	0	0	0	0	0	0
0	0	0	0	0	0	0	0	0	0
0	0	0	0	0	0	0	0	0	0
0	0	0	0	0	0	0	0	0	0
0	0	0	0	0	0	133	0	0	0
0	0	0	48	22	62	0	0	0	0
0	0	0	0	0	0	106	0	25	0
0	0	0	0	0	0	0	0	0	0
0	0	0	0	0	0	0	0	0	0
0	0	0	3	0	0	0	0	0	0
0	0	2	0	0	0	0	3	0	0
0	0	0	0	0	0	0	0	0	0
0	0	0	0	0	0	0	0	0	0
0	22	11	0	0	0	0	0	0	0
0	0	0	0	0	0	0	0	0	0
0	0	0	0	0	0	0	0	0	0
0	0	0	0	0	14	0	0	0	0
0	0	0	0	0	0	0	0	0	0

0	0	0	0	0	0	0	0	0	0
0	0	0	17	0	49	0	0	0	0
0	0	0	0	0	0	0	0	0	0
0	0	0	0	0	0	0	0	0	0
0	0	36	0	0	0	0	0	0	0
0	0	0	0	0	0	0	0	0	0
0	0	0	0	0	0	0	0	0	0
0	0	0	0	0	0	0	0	0	0
0	0	0	0	0	0	0	0	0	0
0	16	0	0	0	0	0	0	0	0
0	0	0	0	15	0	0	0	0	0
0	14	0	0	0	0	0	0	0	0
0	13	0	0	0	0	0	0	0	0
0	0	0	0	0	0	0	0	0	0
0	0	0	0	0	0	0	0	0	0
0	0	0	0	0	0	0	0	0	0
0	0	0	10	0	0	0	0	0	0
0	0	0	0	0	0	0	0	0	0
0	0	0	0	0	0	0	0	0	0
0	0	0	0	0	0	0	0	0	0
0	0	6	0	0	0	0	0	0	0
0	0	0	0	0	6	0	0	0	0
0	0	0	0	0	0	0	0	0	0
0	0	0	0	5	0	0	0	0	0
0	0	0	0	0	5	0	0	0	0
0	0	0	0	0	0	0	0	0	5
0	0	0	0	0	0	0	0	0	0
0	0	0	0	0	0	0	0	0	0
0	0	0	0	0	0	0	0	0	0
0	0	0	0	0	2	0	0	0	0
0	63	51	2	0	10	0	0	2	0
0	0	0	0	0	0	0	0	0	0
0	24	7	0	0	14	0	0	0	0
0	0	0	16	0	0	0	0	0	0
0	11	0	0	0	0	0	0	0	0
0	0	10	0	0	0	0	0	0	0
0	0	8	0	0	0	0	0	0	0
7	0	0	0	0	0	0	0	0	0
0	7	0	0	0	0	0	0	0	0
0	0	0	0	0	0	0	7	0	0
0	0	0	0	0	0	0	0	0	0
0	0	0	0	0	5	0	0	0	0
0	0	0	0	0	0	0	0	0	0
0	0	0	0	0	0	0	0	0	0
0	0	0	0	0	0	0	0	0	0
0	0	0	0	0	0	0	0	0	0
0	0	0	0	0	0	0	0	0	0
0	0	0	0	0	0	0	0	0	0
0	0	0	0	0	0	0	0	0	0
0	0	0	0	0	0	128	0	0	0

0	0	0	0	0	0	0	0	0	0
0	0	0	0	0	0	0	0	0	0
0	0	0	0	0	0	0	0	0	0
0	0	0	0	0	0	0	0	0	0
0	0	0	0	0	0	0	0	0	0
0	0	0	118	0	0	0	0	0	0
0	0	0	0	0	0	0	0	0	27
0	0	0	0	0	0	0	0	0	0
0	0	0	0	0	0	0	0	0	0
0	18	74	7	0	8	4	0	0	0
0	0	0	0	0	0	0	0	0	0
0	0	0	0	0	0	0	0	0	0
0	0	0	0	0	0	0	0	0	0
0	0	0	0	0	0	0	0	0	0
0	0	0	0	0	0	0	0	0	0
0	0	0	112	0	0	0	0	0	0
0	0	0	0	0	0	109	0	0	0
0	0	0	0	0	0	0	0	0	0
0	0	0	0	0	0	0	0	0	0
0	0	0	0	0	0	0	0	0	0
0	0	0	0	0	0	0	0	0	0
0	0	0	0	0	0	0	0	106	0
0	0	0	0	0	0	0	0	0	0
0	0	0	0	0	0	103	0	0	0
0	0	0	0	0	0	0	0	0	0
0	0	0	0	0	0	0	0	0	0
0	0	0	0	0	0	0	0	0	0
0	0	0	0	0	0	0	0	0	0
0	0	0	0	0	0	0	0	0	0
0	0	0	0	0	0	0	0	0	0
0	0	0	0	0	0	0	0	0	0
0	0	0	0	0	0	0	0	0	0
0	0	0	0	0	0	0	0	0	0
0	0	0	0	0	0	0	0	0	0
0	0	0	0	0	0	97	0	0	0
0	0	0	23	11	0	0	0	0	0
0	0	0	0	0	0	0	0	0	0
0	0	0	86	0	0	0	0	0	0
0	0	0	0	0	0	0	0	0	0
0	0	0	0	0	0	0	0	0	0
0	0	0	0	0	0	0	0	0	0
0	0	0	0	0	0	75	0	20	0
0	0	0	0	0	0	0	0	0	0
0	0	0	0	0	0	0	0	0	0
0	0	0	0	0	0	0	0	0	0
0	0	0	0	0	0	0	0	0	0
0	0	0	0	0	0	0	0	0	0
2	3	0	0	80	7	0	0	0	0
0	0	0	0	0	0	0	0	0	0
0	0	0	0	0	0	0	0	0	0

0	0	0	0	0	0	0	0	0	0
0	0	0	0	0	0	0	0	0	0
0	0	0	28	58	0	0	0	0	0
0	0	0	0	0	0	67	0	22	0
0	0	0	0	0	0	0	0	0	0
0	0	0	0	0	0	88	0	0	0
0	0	0	0	0	0	0	0	87	0
0	0	0	0	0	0	0	0	0	0
0	0	0	0	0	0	0	0	0	0
0	0	0	0	0	0	0	0	0	0
0	0	0	0	0	0	0	0	0	0
0	0	0	0	0	0	0	0	0	0
0	0	0	0	0	0	0	0	0	0
0	0	0	0	0	0	0	0	0	0
0	0	0	0	0	0	0	0	0	0
0	0	0	0	0	0	0	0	0	0
0	0	0	0	0	0	0	0	0	0
0	0	0	0	0	0	0	0	0	0
0	0	0	0	0	0	0	0	0	0
0	0	0	0	0	0	0	0	0	0
0	0	0	0	0	0	0	0	0	0
0	51	32	0	0	0	0	0	0	0
0	0	0	0	0	0	0	0	0	0
0	0	0	0	0	0	0	0	0	0
0	0	0	0	0	0	0	0	0	0
0	0	0	0	0	0	0	0	0	0
0	0	0	0	0	0	0	0	0	0
0	0	0	0	0	0	0	0	0	0
0	0	0	0	0	0	0	0	0	0
0	0	0	0	0	0	0	0	0	0
0	0	0	0	0	0	0	0	80	0
0	0	0	0	0	0	0	0	0	0
0	0	0	0	0	0	79	0	0	0
0	0	0	0	0	0	0	0	0	0
54	0	24	0	0	0	0	0	0	0
0	0	0	0	0	0	0	0	0	0
0	0	0	0	0	0	0	0	0	0
0	0	0	0	0	0	0	0	0	0
0	0	0	0	0	0	0	0	0	0
0	0	0	0	0	0	0	0	0	0
0	0	0	0	0	0	0	0	0	0
0	0	0	0	0	0	61	0	14	0
0	0	0	0	0	0	0	0	0	0
0	0	0	0	0	0	0	0	0	0
0	0	0	0	0	0	0	0	0	0
0	0	0	0	0	0	0	0	0	0
0	0	0	0	0	0	0	0	0	0
0	0	0	0	0	0	0	0	0	0
0	0	0	0	0	0	0	0	0	0
0	0	0	0	0	0	0	0	0	0

0	0	0	0	0	0	0	0	0	0
0	0	0	0	0	0	0	0	0	0
0	0	0	0	0	0	0	0	0	0
0	0	0	0	0	0	0	0	0	0
4	21	0	0	0	0	0	0	0	0
0	0	0	0	0	0	0	71	0	0
0	0	0	0	0	0	0	0	0	0
0	0	0	0	0	0	0	0	0	0
66	0	0	0	0	0	0	0	0	4
0	0	0	0	0	0	0	0	0	0
0	0	0	0	0	0	0	0	0	0
0	0	0	0	0	0	0	0	0	0
0	0	0	0	0	0	0	0	0	0
0	0	0	0	0	0	0	0	0	0
0	0	0	0	0	0	0	0	0	0
0	0	0	0	0	0	0	0	0	0
0	0	0	69	0	0	0	0	0	0
0	0	0	0	0	0	0	0	0	0
0	0	0	0	0	0	0	0	0	0
0	0	0	0	0	0	0	0	0	0
0	0	0	0	0	0	0	0	0	0
0	0	0	0	0	0	0	0	0	0
0	0	0	68	0	0	0	0	0	0
0	0	0	0	0	0	9	0	59	0
0	0	0	0	0	0	0	0	0	0
0	0	0	0	0	0	0	0	0	0
0	0	0	0	0	0	0	0	0	0
0	0	0	0	0	0	0	0	0	0
0	0	0	0	0	0	0	0	0	0
0	0	0	0	0	0	0	0	0	0
0	0	0	0	0	0	0	0	0	0
0	0	0	0	0	0	0	0	0	0
0	0	0	0	0	0	0	0	0	0
0	0	0	0	0	0	0	0	0	0
0	0	0	0	0	0	0	0	0	0
0	0	0	0	0	0	0	0	0	0
0	0	0	0	0	0	0	0	0	0
0	0	0	0	0	0	0	0	0	0
0	0	0	0	0	0	0	0	0	0
0	0	0	0	0	0	0	0	0	0
0	0	0	0	0	0	0	0	0	0
0	0	0	0	0	0	0	0	0	0
0	0	0	0	0	0	0	0	0	0
0	0	0	55	8	0	0	0	0	0
0	0	0	0	0	0	0	0	0	0
0	0	0	0	0	0	0	0	0	0
0	0	0	0	0	0	0	0	0	0
0	0	0	0	0	0	0	0	0	0
0	0	0	0	0	0	0	0	0	0

0	0	0	0	0	0	0	0	0	0
0	0	0	0	0	0	0	0	0	0
0	0	0	0	0	0	0	0	0	0
0	0	0	0	0	0	0	0	0	0
0	0	0	0	0	0	0	0	0	0
0	60	0	0	0	0	0	0	0	0
0	0	0	0	0	0	0	0	0	0
0	0	0	0	0	0	0	0	0	0
0	0	0	0	0	0	0	0	0	0
0	0	0	0	0	0	0	0	0	0
0	0	0	0	0	0	0	0	0	0
0	0	0	0	0	0	0	0	0	0
0	0	0	0	0	0	0	0	0	0
0	0	0	0	0	0	0	0	0	0
0	0	0	0	0	0	0	0	0	0
0	0	0	0	0	0	0	0	0	0
0	0	0	58	0	0	0	0	0	0
0	0	0	0	0	0	0	0	0	0
0	0	0	0	0	0	0	0	0	0
0	0	0	0	0	0	0	0	0	0
0	0	0	0	0	0	0	0	0	0
0	0	0	0	0	0	0	0	0	0
0	0	0	0	34	0	0	0	0	0
0	0	0	0	0	0	0	0	0	0
0	0	0	0	0	0	0	0	0	0
0	0	0	0	0	0	0	0	0	0
0	0	0	0	0	0	0	0	0	0
0	0	0	0	0	0	0	0	0	0
0	0	0	0	0	0	0	0	0	0
0	0	0	0	0	0	0	0	0	0
0	0	0	0	0	0	0	0	0	0
0	0	0	0	0	0	0	0	0	0
0	0	0	0	0	0	0	0	0	0
0	0	0	0	0	0	0	0	0	0
0	0	0	0	0	0	0	0	0	0
0	0	0	0	0	0	0	0	0	0
0	0	0	0	0	55	0	0	0	0
0	0	0	0	0	0	0	0	0	0
0	0	0	0	0	0	0	0	0	0
0	0	0	0	0	0	0	0	0	0
0	0	0	0	0	0	0	0	0	0
0	0	0	0	0	0	0	0	0	0
0	0	0	0	0	0	0	0	0	0
0	0	0	0	0	0	0	0	0	0
0	0	0	0	0	0	0	0	0	0
0	0	0	0	0	0	0	0	0	0
0	0	0	0	0	0	0	0	0	0
0	0	0	0	0	0	0	0	0	0
0	0	0	0	0	0	0	0	0	0
0	0	0	0	0	0	0	0	0	0

0	0	0	0	0	0	0	0	0	0
0	0	0	0	0	0	0	0	0	0
0	0	0	0	0	0	0	0	0	0
0	0	0	0	0	0	0	0	0	0
0	0	0	0	0	0	0	0	0	0
0	0	0	0	0	0	0	0	0	0
0	0	0	0	0	0	0	0	0	0
0	0	0	20	33	0	0	0	0	0
0	0	0	0	0	0	0	0	0	0
0	0	0	0	0	0	0	0	0	0
0	0	0	0	0	0	0	0	0	0
0	0	0	0	0	0	0	0	0	0
0	0	0	0	0	0	0	0	0	0
0	0	0	39	0	0	0	0	0	0
0	0	0	0	0	0	0	0	52	0
0	0	0	0	0	0	0	0	52	0
0	0	0	0	0	0	0	0	0	0
0	0	0	0	0	0	0	0	0	0
0	0	0	0	0	0	0	0	0	0
0	0	0	0	0	0	0	0	0	0
0	0	0	0	0	0	0	0	0	0
0	0	0	0	0	0	0	0	0	0
0	0	0	51	0	0	0	0	0	0
0	0	0	0	0	0	0	0	0	0
0	0	0	0	0	0	0	0	0	0
0	0	0	0	0	0	0	0	0	0
0	0	0	0	0	0	0	0	0	0
0	0	0	0	0	0	0	0	0	0
0	0	0	0	0	0	0	0	0	0
0	0	0	0	0	0	0	0	0	0
0	0	0	0	0	0	0	0	0	0
0	0	0	50	0	0	0	0	0	0
0	0	0	0	0	0	14	0	36	0
0	0	0	0	0	0	0	0	0	0
0	0	0	0	0	0	0	0	0	0
0	0	0	0	0	0	0	0	0	0
0	0	0	0	0	0	0	0	0	0
0	0	0	0	0	0	0	0	0	0
0	0	0	0	0	0	0	0	0	0
0	0	0	0	0	0	0	0	0	0
0	0	0	0	0	0	0	0	0	0
0	0	0	0	0	0	0	0	0	0
0	0	0	0	0	0	0	0	0	0
0	0	0	49	0	0	0	0	0	0
0	0	0	0	0	0	0	0	0	0
0	0	0	0	0	0	0	0	0	0
0	0	0	0	0	0	0	0	0	0
0	0	0	0	0	0	0	0	0	0
0	0	0	0	0	0	0	0	0	0
0	0	0	0	0	0	0	0	0	0

0	0	0	0	0	0	0	0	0	0
0	0	0	0	0	0	0	0	0	0
0	0	0	0	0	0	0	0	0	0
0	0	0	0	0	0	0	0	0	0
0	0	0	0	0	0	0	0	0	0
0	0	0	0	0	0	0	0	0	0
0	3	0	0	0	39	0	0	0	0
0	0	0	0	0	0	0	0	0	0
0	0	0	0	0	0	0	0	0	0
0	0	0	0	0	0	0	0	0	0
0	0	0	0	0	0	0	0	0	0
0	0	0	0	0	0	0	0	0	0
0	0	0	0	0	0	0	0	0	0
0	0	0	0	0	0	0	0	0	0
0	0	0	0	0	0	0	0	0	0
0	0	0	0	0	0	0	0	0	0
11	0	23	0	0	6	0	0	0	0
0	0	0	0	0	0	0	0	0	0
0	0	0	0	0	0	0	0	0	0
0	0	0	0	0	0	0	0	0	0
0	0	0	0	0	0	0	0	0	0
0	0	0	0	0	0	0	0	0	0
0	0	0	0	0	0	0	0	0	0
0	0	0	0	0	0	0	0	0	0
0	0	0	0	0	0	11	24	11	0
0	0	0	0	0	0	0	0	0	0
0	0	0	0	0	0	0	0	0	0
0	0	0	0	0	0	0	0	0	0
0	0	0	0	0	0	0	0	0	0
0	0	0	0	0	0	0	0	0	0
0	0	0	0	0	0	0	0	0	0
0	0	0	0	0	0	0	0	0	0
0	0	0	0	0	0	0	0	0	0
0	0	0	0	0	0	0	0	0	0
0	0	0	0	0	0	0	0	0	0
0	0	0	0	0	0	0	0	0	0
0	0	0	0	0	0	0	0	0	0
0	0	0	0	0	0	0	0	0	0
0	0	0	0	4	0	0	0	0	0
0	0	0	0	0	0	0	0	0	0
0	0	0	0	0	0	0	0	0	0
0	0	0	0	0	0	0	0	0	0
0	0	0	0	0	0	0	0	0	0
0	0	0	0	0	0	0	0	0	0
0	0	0	0	0	0	0	0	0	0
0	0	0	0	0	0	0	0	0	0
0	0	0	0	0	0	0	0	0	0
0	0	0	0	0	0	0	0	0	0
0	0	0	0	0	0	0	0	0	0
0	0	0	0	0	0	0	0	0	0

0	0	0	0	0	0	0	0	0	0
0	0	0	0	0	0	0	0	0	0
0	0	0	0	0	0	0	0	0	0
0	0	0	0	0	0	0	0	0	0
18	0	25	0	0	0	0	0	0	0
0	0	0	0	0	0	0	0	43	0
0	0	0	0	0	0	0	0	0	0
0	0	0	0	0	0	0	0	0	0
0	0	0	0	0	0	0	0	0	0
0	0	0	0	0	0	0	0	0	0
0	0	0	0	0	0	0	0	0	0
0	0	0	25	0	0	0	0	0	0
0	0	0	0	0	0	0	0	0	0
0	0	0	0	0	0	0	0	0	0
0	0	0	0	0	0	0	0	0	0
0	0	0	0	0	0	0	0	0	0
0	0	0	0	0	0	0	0	0	0
0	0	0	0	0	0	0	0	0	0
0	0	0	0	0	0	0	0	0	0
0	0	0	0	0	0	0	0	0	0
0	0	0	0	0	0	0	0	0	0
0	0	0	0	0	0	0	0	0	0
0	0	0	0	0	0	0	0	0	0
0	0	0	0	0	0	0	0	0	0
0	0	0	0	0	0	0	0	0	0
0	0	0	0	0	0	0	0	0	0
0	0	0	41	0	0	0	0	0	0
0	0	0	0	0	0	0	0	0	0
0	0	0	0	0	0	0	0	0	0
0	0	0	0	0	0	0	0	0	0
0	0	0	0	0	0	0	0	0	0
0	0	0	0	0	0	0	0	0	0
0	0	0	0	0	0	0	0	0	0
0	0	0	0	0	0	0	0	0	0
0	0	0	0	0	0	0	0	0	0
0	0	0	0	0	0	0	0	0	0
0	0	0	0	0	0	0	0	0	0
0	0	0	0	0	0	0	0	0	0
0	0	0	0	0	0	40	0	0	0
0	0	0	0	0	0	0	0	0	0
0	0	0	0	0	0	0	0	0	0
0	0	0	0	0	0	0	0	0	0
0	0	0	0	0	0	0	0	0	0
17	0	0	0	0	0	0	0	0	0
0	0	0	0	0	0	0	0	0	0
0	0	0	0	0	0	0	0	0	0
0	0	0	0	0	0	0	0	0	0
0	0	0	0	0	0	0	0	0	0
0	0	0	0	0	0	0	0	0	0
0	0	0	0	0	0	0	0	0	0

0	0	0	0	0	0	0	0	0	0
0	0	0	0	0	0	0	0	0	0
0	0	0	0	0	0	0	0	0	0
0	0	0	0	0	0	0	0	0	0
0	0	0	0	0	0	0	0	0	0
0	0	0	0	0	0	0	0	0	0
0	0	0	0	0	0	0	0	0	0
0	0	0	0	0	0	0	0	0	0
0	0	0	0	0	0	0	0	0	0
0	0	0	0	0	0	0	0	0	0
0	0	0	0	0	0	0	0	0	0
0	0	0	0	0	0	0	0	0	0
0	0	0	0	0	0	0	0	0	0
0	0	0	0	0	0	0	0	0	0
0	0	0	0	38	0	0	0	0	0
0	0	0	0	0	0	38	0	0	0
0	0	0	0	0	0	0	0	0	0
0	0	0	0	0	0	0	0	0	0
0	0	0	0	0	0	0	0	0	0
0	0	0	0	0	0	0	0	0	0
0	0	0	0	0	0	0	0	0	0
0	0	0	0	0	0	0	0	0	0
0	0	0	0	0	0	0	0	0	0
0	0	0	0	0	0	0	0	0	0
0	0	0	0	0	0	0	0	0	0
0	0	0	0	0	0	0	0	0	0
0	0	0	0	37	0	0	0	0	0
0	0	0	0	0	0	0	0	37	0
0	0	0	0	0	0	0	0	0	0
0	0	0	0	0	0	0	0	0	0
0	0	0	0	0	0	0	0	0	0
0	0	0	0	0	0	0	0	0	0
0	0	0	0	0	0	0	0	0	0
0	0	0	0	0	0	0	0	0	0
0	0	0	0	0	0	0	0	0	0
0	0	0	0	0	0	0	0	0	0
0	0	0	0	0	9	0	0	0	0
0	0	0	0	0	0	0	0	0	0
0	0	0	0	0	0	0	0	0	0
0	0	0	0	0	0	0	0	0	0
0	0	0	0	0	0	0	0	0	0
0	0	0	0	0	0	0	0	0	0
0	0	0	0	36	0	0	0	0	0
0	0	0	0	0	0	0	0	0	0
0	0	0	0	0	0	0	0	0	0
0	0	0	0	0	0	0	0	0	0
0	0	0	0	0	0	0	0	0	0
0	0	0	0	0	0	0	0	0	0
0	0	0	0	0	0	0	0	0	0
0	0	0	0	0	0	0	0	0	0

0	0	0	0	0	0	0	0	0	0
0	0	0	0	0	0	0	0	0	0
0	0	0	0	0	0	0	0	0	0
0	0	0	0	0	0	0	0	0	0
0	0	0	0	0	0	0	0	0	0
0	0	0	0	0	0	0	0	0	0
0	0	0	0	0	0	0	0	0	0
0	0	0	0	0	0	0	0	0	0
0	0	0	0	0	0	0	0	0	0
0	35	0	0	0	0	0	0	0	0
0	0	0	0	0	0	35	0	0	0
0	0	0	0	0	0	0	0	0	35
0	0	0	0	0	0	0	0	0	0
0	0	0	0	0	0	0	0	0	0
0	0	0	0	0	0	0	0	0	0
0	0	0	0	0	0	0	0	0	0
0	0	0	0	0	0	0	0	0	0
0	0	0	0	0	0	0	0	0	0
0	0	0	0	0	0	0	0	0	0
0	0	0	0	0	0	0	0	0	0
0	0	0	0	0	0	0	0	0	0
0	2	3	0	8	13	0	0	0	0
0	0	28	0	0	0	0	0	0	0
0	0	0	0	0	0	0	0	0	0
0	0	0	0	0	0	0	0	0	0
0	34	0	0	0	0	0	0	0	0
0	0	0	34	0	0	0	0	0	0
0	0	0	0	0	0	0	0	0	0
0	0	0	0	0	0	0	0	0	0
0	0	0	0	0	0	0	0	0	0
0	0	0	0	0	0	0	0	0	0
0	0	0	0	0	0	0	0	0	0
0	0	0	0	0	0	0	0	0	0
0	0	0	0	0	0	0	0	0	0
0	0	0	0	0	0	0	0	0	0
0	0	0	0	0	0	0	0	0	0
0	0	0	0	0	0	0	0	0	0
0	0	0	0	0	0	0	0	0	0
0	0	0	0	0	0	0	0	0	0
0	0	0	0	0	0	0	0	0	0
0	0	0	15	0	0	0	0	0	0
0	0	0	0	0	0	0	0	0	0
0	0	0	0	0	0	33	0	0	0
0	0	0	0	0	0	0	0	33	0
0	0	0	0	0	0	0	0	0	0
0	0	0	0	0	0	0	0	0	0
0	0	0	0	0	0	0	0	0	0
0	0	0	0	0	0	0	0	0	0
0	0	0	0	0	0	0	0	0	0

0	0	0	0	0	0	0	0	0	0
0	0	0	0	0	0	0	0	0	0
0	0	0	0	0	0	0	0	0	0
0	0	0	0	0	0	0	0	0	0
0	0	0	0	0	0	0	0	0	0
0	0	0	0	0	0	0	0	0	0
0	0	0	0	0	0	0	0	0	0
0	0	0	0	0	0	0	0	0	0
0	0	0	0	0	0	0	0	0	0
0	0	0	0	0	0	0	0	0	0
0	0	0	0	0	0	0	0	0	0
0	0	0	0	0	0	0	0	0	0
0	0	0	0	0	0	0	0	0	0
0	0	0	0	0	0	0	0	0	0
0	0	0	0	0	27	0	0	0	0
0	0	0	0	0	0	0	0	32	0
0	0	0	0	0	0	0	0	0	0
0	0	0	0	0	0	0	0	0	0
0	0	0	0	0	0	0	0	0	0
0	0	0	0	0	0	0	0	0	0
0	0	0	0	0	0	0	0	0	0
0	0	0	0	0	0	0	0	0	0
0	0	0	0	0	0	0	0	0	0
0	0	0	0	0	0	0	0	0	0
0	0	0	0	0	0	0	0	0	0
0	0	0	0	0	0	0	0	0	0
0	0	0	0	0	0	0	0	0	0
0	0	0	0	0	0	0	0	0	0
0	0	0	0	0	0	0	0	0	0
0	0	0	0	0	0	0	0	0	0
0	0	0	0	0	0	0	0	0	0
0	0	0	0	0	0	0	0	0	0
0	0	0	0	0	0	0	0	0	0
0	0	0	0	0	0	0	0	0	0
0	0	0	0	0	0	0	31	0	0
0	0	0	0	0	0	0	0	0	0
0	0	0	0	0	0	0	0	0	0
0	0	0	0	0	0	0	0	0	0
0	0	0	0	0	0	0	0	0	0
0	0	0	0	0	0	0	0	0	0
0	0	0	0	0	0	0	0	0	0
0	0	0	0	0	0	0	0	0	0
0	0	0	0	0	0	0	0	0	0
0	0	0	0	0	0	0	0	0	0
0	0	0	0	0	0	0	0	0	0
0	0	0	0	0	0	0	0	0	0
0	0	0	0	0	0	0	0	0	0
0	0	0	0	0	0	0	0	0	0
0	6	14	0	0	0	0	0	0	0
0	0	0	28	0	0	0	0	0	0

0	0	0	0	0	0	0	0	0	0
0	0	30	0	0	0	0	0	0	0
0	0	0	30	0	0	0	0	0	0
0	0	0	30	0	0	0	0	0	0
0	0	0	0	0	30	0	0	0	0
0	0	0	0	0	0	0	0	0	0
0	0	0	0	0	0	0	0	0	0
0	0	0	0	0	0	0	0	0	0
0	0	0	0	0	0	0	0	0	0
0	0	0	0	0	0	0	0	0	0
0	0	0	0	0	0	0	0	0	0
0	0	0	0	0	0	0	0	0	0
0	0	0	0	0	0	0	0	0	0
0	0	0	0	0	0	0	0	0	0
0	0	0	0	0	0	0	0	0	0
0	0	0	0	0	0	0	0	0	0
0	0	0	0	0	0	0	0	0	0
0	0	0	0	0	0	0	0	0	0
0	0	0	0	0	0	0	0	0	0
0	0	0	0	0	0	0	0	0	0
0	0	0	0	0	0	0	0	0	0
19	10	0	0	0	0	0	0	0	0
0	0	0	29	0	0	0	0	0	0
0	0	0	29	0	0	0	0	0	0
0	0	0	26	0	0	0	0	0	0
0	0	0	0	0	0	0	0	0	0
0	0	0	0	0	0	0	0	0	0
0	0	0	0	0	0	0	0	0	0
0	0	0	0	0	0	0	0	0	0
0	0	0	0	0	0	0	0	0	0
0	0	0	0	0	0	0	0	0	0
0	0	0	0	0	0	0	0	0	0
0	0	0	0	0	0	0	0	0	0
0	0	0	0	0	0	0	0	0	0
0	0	0	0	0	0	0	0	0	0
0	0	0	0	0	0	0	0	0	0
0	0	0	0	0	0	0	0	0	0
0	0	0	0	0	0	0	0	0	0
0	0	0	0	0	0	0	0	0	0
0	0	0	0	0	0	0	0	0	0
0	0	0	0	0	0	0	0	0	0
0	0	0	0	0	0	0	0	0	0
0	0	0	0	0	0	0	0	0	0
0	0	0	28	0	0	0	0	0	0
0	0	0	28	0	0	0	0	0	0
0	0	0	28	0	0	0	0	0	0
0	0	0	0	0	28	0	0	0	0
0	0	0	0	0	28	0	0	0	0
0	0	0	0	0	0	0	0	0	0
0	0	0	0	0	0	0	0	0	0

0	0	0	0	0	0	0	0	0	0
0	0	0	0	0	0	0	0	0	0
0	0	0	0	0	0	0	0	0	0
0	0	0	0	0	0	0	0	0	0
0	0	0	0	0	0	0	0	0	0
0	0	0	0	0	0	0	0	0	0
0	0	0	0	0	0	0	0	0	0
0	0	0	0	0	0	0	0	0	0
0	0	0	0	0	0	0	0	0	0
0	0	0	0	0	0	0	0	0	0
0	0	0	0	0	0	0	0	0	0
0	0	0	0	0	0	0	0	0	0
0	0	0	0	0	0	0	0	0	0
0	0	0	0	0	0	0	0	0	0
0	0	0	0	0	0	0	0	0	0
0	0	0	0	0	0	0	0	0	0
0	0	0	0	0	0	0	0	0	0
0	0	0	0	0	0	0	0	0	0
0	0	0	0	0	0	0	0	0	0
0	0	0	0	0	0	0	0	0	0
0	0	0	0	0	0	0	0	0	0
0	0	0	0	0	0	0	0	0	0
0	0	0	0	0	0	0	0	0	0
0	0	0	0	0	0	0	0	0	0
0	0	0	27	0	0	0	0	0	0
0	0	0	27	0	0	0	0	0	0
0	0	0	0	0	0	0	0	0	0
0	0	0	0	0	0	0	0	0	0
0	0	0	0	0	0	0	0	0	0
0	0	0	0	0	0	0	0	0	0
0	0	0	0	0	0	0	0	0	0
0	0	0	0	0	0	0	0	0	0
0	0	0	0	0	0	0	0	0	0
0	0	0	0	0	0	0	0	0	0
0	0	0	0	0	0	0	0	0	0
0	0	0	0	0	0	0	0	0	0
0	0	0	0	0	0	0	0	0	0
0	0	0	0	0	0	0	0	0	0
0	0	0	0	0	0	0	0	0	0
0	0	0	0	0	0	0	0	0	0
0	0	0	0	0	0	0	0	0	0
0	0	0	0	0	0	0	0	0	0
0	0	0	0	0	0	0	0	0	0
0	0	0	0	0	0	0	0	0	0
0	0	0	0	0	0	0	0	0	0
0	0	0	0	0	0	0	0	0	0
0	0	0	0	0	0	0	0	0	0
0	0	0	0	0	0	0	0	0	0
0	0	0	0	0	0	0	0	0	0
0	0	0	0	0	0	0	0	0	0
0	0	0	0	0	0	0	0	0	0

0	0	0	0	0	0	0	0	0	0
0	0	0	26	0	0	0	0	0	0
0	0	0	0	0	0	0	0	0	0
0	0	0	0	0	0	0	0	0	0
0	0	0	0	0	0	0	0	0	0
0	0	0	0	0	0	0	0	0	0
0	0	0	0	0	0	0	0	0	0
0	0	0	0	0	0	0	0	0	0
0	0	0	0	0	0	0	0	0	0
0	0	0	0	0	0	0	0	0	0
0	0	0	0	0	0	0	0	0	0
0	0	0	0	0	0	0	0	0	0
0	0	0	0	0	0	0	0	0	0
0	0	0	0	0	0	0	0	0	0
0	0	0	0	0	0	0	0	0	0
0	0	0	0	0	0	0	0	0	0
0	0	0	0	0	0	0	0	0	0
0	0	0	0	0	0	0	0	0	0
0	0	0	0	0	0	0	0	0	0
0	0	0	0	0	0	0	0	0	0
0	0	0	0	0	0	0	0	0	0
0	0	0	0	0	0	0	0	0	0
0	0	0	0	0	0	0	0	0	0
0	0	0	0	0	0	0	0	0	0
0	20	5	0	0	0	0	0	0	0
0	0	0	25	0	0	0	0	0	0
0	0	0	25	0	0	0	0	0	0
0	0	0	0	0	0	0	0	0	0
0	0	0	0	0	0	0	0	0	0
0	0	0	0	0	0	0	0	0	0
0	0	0	0	0	0	0	0	0	0
0	0	0	0	0	0	0	0	0	0
0	0	0	0	0	0	0	0	0	0
0	0	0	0	0	0	0	0	0	0
0	0	0	0	0	0	0	0	0	0
0	0	0	0	0	0	0	0	0	0
0	0	0	0	0	0	0	0	0	0
0	0	0	0	0	0	0	0	0	0
0	0	0	0	0	0	0	0	0	0
0	0	0	0	0	0	0	0	0	0
0	0	0	0	0	0	0	0	0	0
0	0	0	0	0	0	0	0	0	0
0	0	0	0	0	0	0	0	0	0
0	0	0	0	0	0	0	0	0	0
0	0	0	0	0	0	0	0	0	0
0	0	0	0	0	0	0	0	0	0
0	0	0	0	0	0	0	0	0	0
0	0	0	0	0	0	0	0	0	0
0	0	0	0	0	0	0	0	0	0
0	0	0	0	0	0	0	0	0	0
0	0	0	0	0	0	0	0	0	0

0	0	0	0	0	0	0	0	0	0
0	0	0	0	0	0	0	0	0	0
0	0	0	0	0	0	0	0	0	0
0	0	0	0	0	0	0	0	0	0
0	0	0	0	0	0	0	0	0	0
4	0	0	20	0	0	0	0	0	0
0	0	0	0	0	0	0	0	0	0
0	0	0	0	0	0	0	0	0	0
0	0	0	0	0	0	0	0	0	0
0	0	0	0	0	0	0	0	0	0
0	0	0	0	0	0	0	0	0	0
0	0	0	0	0	0	0	0	0	0
0	0	0	0	0	0	0	0	0	0
0	0	0	0	0	0	0	0	0	0
0	0	0	0	0	0	0	0	0	0
0	0	0	0	0	0	0	0	0	0
0	0	0	0	0	0	0	0	0	0
0	0	0	0	0	0	0	0	0	0
0	0	0	0	0	0	0	0	0	0
0	0	0	0	0	0	0	0	0	0
0	0	0	0	0	0	0	0	0	0
0	0	0	0	0	0	0	0	0	0
0	0	0	0	0	0	0	0	0	0
0	0	0	0	0	0	0	0	0	0
0	0	0	0	0	0	0	0	0	0
0	0	0	0	0	0	0	0	0	0
0	0	0	0	0	0	0	0	0	0
0	0	0	0	0	0	0	0	0	0
0	0	0	0	0	0	0	0	0	0
0	0	0	0	0	0	0	0	0	0
0	0	0	0	0	0	0	0	0	0
0	0	0	0	0	0	0	0	0	0
0	0	0	0	0	0	0	0	0	0
0	0	0	0	0	0	0	0	0	0
0	0	0	0	0	0	0	0	0	0
0	0	0	23	0	0	0	0	0	0
0	0	0	0	0	0	0	0	0	0
0	0	0	0	0	0	0	0	0	0
0	0	0	0	0	0	0	0	0	0
0	0	0	0	0	0	0	0	0	0
0	0	0	0	0	0	0	0	0	0
0	0	0	0	0	0	0	0	0	0
0	0	0	0	0	0	0	0	0	0
0	0	0	0	0	0	0	0	0	0
0	0	0	0	0	0	0	0	0	0
0	0	0	0	0	0	0	0	0	0
0	0	0	0	0	0	0	0	0	0
0	0	0	0	0	0	0	0	0	0
0	0	0	0	0	0	0	0	0	0
0	0	0	0	0	0	0	0	0	0

0	0	0	0	0	0	0	0	0	0
0	0	0	0	0	14	0	0	0	0
0	0	0	0	0	0	0	0	0	0
0	0	0	0	0	0	0	0	0	0
0	0	0	0	0	0	0	0	0	0
0	0	0	0	0	0	0	0	0	0
0	0	0	0	0	0	0	0	0	0
0	0	0	0	0	0	0	0	0	0
0	0	0	0	0	0	0	0	0	0
0	0	0	0	0	0	0	0	0	0
0	0	0	0	0	0	0	0	0	0
0	0	0	0	0	0	0	0	0	0
0	0	0	0	0	0	0	0	0	0
0	0	0	0	0	0	0	0	0	0
6	6	10	0	0	0	0	0	0	0
0	0	0	22	0	0	0	0	0	0
0	0	0	22	0	0	0	0	0	0
0	0	0	0	0	22	0	0	0	0
0	0	0	0	0	0	22	0	0	0
0	0	0	0	0	0	0	0	0	0
0	0	0	0	0	0	0	0	0	0
0	0	0	0	0	0	0	0	0	0
0	0	0	0	0	0	0	0	0	0
0	0	0	0	0	0	0	0	0	0
0	0	0	0	0	0	0	0	0	0
0	0	0	0	0	0	0	0	0	0
0	0	0	0	0	0	0	0	0	0
0	0	0	0	0	0	0	0	0	0
0	0	0	0	0	0	0	0	0	0
0	0	0	0	0	0	0	0	0	0
0	0	0	0	0	0	0	0	0	0
0	0	0	0	0	0	0	0	0	0
0	0	0	0	0	0	0	0	0	0
0	0	0	0	0	0	0	0	0	0
0	0	0	0	0	0	0	0	0	0
0	0	0	0	0	0	0	0	0	0
0	0	0	0	0	0	0	0	0	0
0	0	7	0	0	0	0	0	0	0
0	0	0	0	0	0	0	0	0	0
0	0	0	0	0	0	0	0	0	0
0	0	0	0	0	0	0	0	0	0
0	0	0	0	0	0	0	0	0	0
0	0	0	0	0	0	0	0	0	0
0	0	0	0	0	0	0	0	0	0
0	0	0	0	0	0	0	0	0	0
21	0	0	0	0	0	0	0	0	0
0	21	0	0	0	0	0	0	0	0
0	0	0	21	0	0	0	0	0	0
0	0	0	0	0	0	0	0	0	0
0	0	0	0	0	0	0	0	0	0

0	0	0	0	0	0	0	0	0	0
0	0	0	0	0	0	0	0	0	0
0	0	0	0	0	0	0	0	0	0
0	0	0	0	0	0	0	0	0	0
0	0	0	0	0	0	0	0	0	0
0	0	0	0	0	0	0	0	0	0
0	0	0	0	0	0	0	0	0	0
0	0	0	0	0	0	0	0	0	0
0	0	0	0	0	0	0	0	0	0
0	0	0	0	0	0	0	0	0	0
0	0	0	0	0	0	0	0	0	0
0	0	0	0	0	0	0	0	0	0
0	0	0	0	0	0	0	0	0	0
0	0	0	0	0	0	0	0	0	0
0	0	0	0	0	0	0	0	0	0
0	0	0	0	0	0	0	0	0	0
0	0	0	0	0	0	0	0	0	0
0	0	0	0	0	0	0	0	0	0
0	0	0	0	0	0	0	0	0	0
0	0	0	0	0	0	0	0	0	0
0	0	0	0	0	0	0	0	0	0
0	0	0	0	0	0	0	0	0	0
0	0	0	0	0	0	0	0	0	0
0	0	0	0	0	0	0	0	0	0
0	0	0	0	0	0	0	0	0	0
0	0	0	0	0	0	0	0	0	0
0	0	0	0	0	0	0	0	0	0
0	0	0	0	0	0	0	0	0	0
0	0	0	0	0	0	0	0	0	0
0	0	0	0	0	0	0	0	0	0
0	0	0	0	0	0	0	0	0	0
0	0	0	20	0	0	0	0	0	0
0	0	0	20	0	0	0	0	0	0
0	0	0	20	0	0	0	0	0	0
0	0	0	20	0	0	0	0	0	0
0	0	0	20	0	0	0	0	0	0
0	0	0	0	0	0	0	0	0	0
0	0	0	0	0	0	0	0	0	0
0	0	0	0	0	0	0	0	0	0
0	0	0	0	0	0	0	0	0	0
0	0	0	0	0	0	0	0	0	0
0	0	0	0	0	0	0	0	0	0
0	0	0	0	0	0	0	0	0	0
0	0	0	0	0	0	0	0	0	0
0	0	0	0	0	0	0	0	0	0
0	0	0	0	0	0	0	0	0	0
0	0	0	0	0	0	0	0	0	0
0	0	0	0	0	0	0	0	0	0
0	0	0	0	0	0	0	0	0	0
0	0	0	0	0	0	0	0	0	0

0	0	0	0	0	0	0	0	0	0
0	0	0	0	0	0	0	0	0	0
0	0	0	0	0	0	0	0	0	0
0	0	0	0	0	0	0	0	0	0
0	0	0	0	0	0	0	0	0	0
0	0	0	0	0	0	0	0	0	0
0	0	0	0	0	0	0	0	0	0
0	0	0	0	0	0	0	0	0	0
0	0	0	0	0	0	0	0	0	0
0	0	0	0	0	0	0	0	0	0
0	0	0	0	0	0	0	0	0	0
0	0	0	19	0	0	0	0	0	0
0	0	0	19	0	0	0	0	0	0
0	0	0	0	0	0	0	0	0	0
0	0	0	0	0	0	0	0	0	0
0	0	0	0	0	0	0	0	0	0
0	0	0	0	0	0	0	0	0	0
0	0	0	0	0	0	0	0	0	0
0	0	0	0	0	0	0	0	0	0
0	0	0	0	0	0	0	0	0	0
0	0	0	0	0	0	0	0	0	0
0	0	0	0	0	0	0	0	0	0
0	0	0	0	0	0	0	0	0	0
0	0	0	0	0	0	0	0	0	0
0	0	0	0	0	0	0	0	0	0
0	0	0	0	0	0	0	0	0	0
0	0	0	0	0	0	0	0	0	0
0	0	0	0	0	0	0	0	0	0
0	0	0	0	0	0	0	0	0	0
0	0	0	0	0	0	0	0	0	0
0	0	0	0	0	0	0	0	0	0
0	0	0	0	0	0	0	0	0	0
0	0	0	0	0	0	0	0	0	0
0	0	0	0	0	0	0	0	0	0
0	0	0	0	0	0	0	0	0	0
0	0	0	0	0	0	0	0	0	0
0	0	0	0	0	0	0	6	0	0
0	0	0	18	0	0	0	0	0	0
0	0	0	18	0	0	0	0	0	0
0	0	0	16	0	2	0	0	0	0
0	0	0	14	0	4	0	0	0	0
0	0	0	0	18	0	0	0	0	0
0	0	0	0	0	0	0	0	0	0
0	0	0	0	0	0	0	0	0	0
0	0	0	0	0	0	0	0	0	0
0	0	0	0	0	0	0	0	0	0
0	0	0	0	0	0	0	0	0	0
0	0	0	0	0	0	0	0	0	0
0	0	0	0	0	0	0	0	0	0
0	0	0	0	0	0	0	0	0	0

0	0	0	0	0	0	0	0	0	0
0	0	0	0	0	0	0	0	0	0
0	0	0	0	0	0	0	0	0	0
0	0	0	0	0	0	0	0	0	0
0	0	0	0	0	0	0	0	0	0
0	0	0	0	0	0	0	0	0	0
0	0	0	0	0	0	0	0	0	0
0	0	0	0	0	0	0	0	0	0
0	0	0	0	0	0	0	0	0	0
0	0	0	0	0	0	0	0	0	0
0	0	0	0	0	0	0	0	0	0
0	0	0	0	0	0	0	0	0	0
0	0	0	0	0	0	0	0	0	0
0	0	0	0	0	0	0	0	0	0
0	0	0	0	0	0	0	0	0	0
0	0	0	0	0	0	0	0	0	0
0	0	0	0	0	0	0	0	0	0
0	0	0	0	0	0	0	0	0	0
0	0	0	0	0	0	0	0	0	0
0	0	0	0	0	0	0	0	0	0
0	0	0	0	0	0	0	0	0	0
0	0	0	17	0	0	0	0	0	0
0	0	0	17	0	0	0	0	0	0
0	0	0	0	0	17	0	0	0	0
0	0	0	0	0	0	0	17	0	0
0	0	0	0	0	0	0	0	0	17
0	0	0	0	0	0	0	0	0	0
0	0	0	0	0	0	0	0	0	0
0	0	0	0	0	0	0	0	0	0
0	0	0	0	0	0	0	0	0	0
0	0	0	0	0	0	0	0	0	0
0	0	0	0	0	0	0	0	0	0
0	0	0	0	0	0	0	0	0	0
0	0	0	0	0	0	0	0	0	0
0	0	0	0	0	0	0	0	0	0
0	0	0	0	0	0	0	0	0	0
0	0	0	0	0	0	0	0	0	0
0	0	0	0	0	0	0	0	0	0
0	0	0	0	0	0	0	0	0	0
0	0	0	0	0	0	0	0	0	0
0	0	0	0	0	0	0	0	0	0
0	0	0	0	0	0	0	0	0	0
0	0	0	0	0	0	0	0	0	0
0	0	0	0	0	0	0	0	0	0
0	0	0	0	0	0	0	0	0	0
0	0	0	0	0	0	0	0	0	0
0	0	0	0	0	0	0	0	0	0
0	0	0	0	9	0	0	0	0	0
0	0	0	0	0	0	0	0	0	0
0	0	0	0	0	0	0	0	0	0

0	0	0	0	0	0	0	0	0	0
0	0	0	0	0	0	0	0	0	0
0	0	0	0	0	0	0	0	0	0
0	0	0	0	0	0	0	0	0	0
0	0	0	0	0	0	0	0	0	0
0	0	0	0	0	0	0	0	0	0
0	0	0	0	0	0	0	0	0	0
0	0	0	0	0	0	0	0	0	0
0	2	0	0	0	14	0	0	0	0
0	0	16	0	0	0	0	0	0	0
0	0	0	16	0	0	0	0	0	0
0	0	0	0	0	16	0	0	0	0
0	0	0	0	0	0	0	0	0	0
0	0	0	0	0	0	0	0	0	0
0	0	0	0	0	0	0	0	0	0
0	0	0	0	0	0	0	0	0	0
0	0	0	0	0	0	0	0	0	0
0	0	0	0	0	0	0	0	0	0
0	0	0	0	0	0	0	0	0	0
0	0	0	0	0	0	0	0	0	0
0	0	0	0	0	0	0	0	0	0
0	0	0	0	0	0	0	0	0	0
0	0	0	0	0	0	0	0	0	0
0	0	0	0	0	0	0	0	0	0
0	0	0	0	0	0	0	0	0	0
0	0	0	0	0	0	0	0	0	0
0	0	0	0	0	0	0	0	0	0
0	0	0	0	0	0	0	0	0	0
0	0	0	0	0	0	0	0	0	0
0	0	0	0	0	0	0	0	0	0
0	0	0	0	0	0	0	0	0	0
0	0	0	0	0	0	0	0	0	0
0	0	0	0	0	0	0	0	0	0
0	0	6	0	0	0	0	0	0	0
0	15	0	0	0	0	0	0	0	0
0	0	0	15	0	0	0	0	0	0
0	0	0	11	0	0	4	0	0	0
0	0	0	0	0	0	0	0	0	0
0	0	0	0	0	0	0	0	0	0
0	0	0	0	0	0	0	0	0	0
0	0	0	0	0	0	0	0	0	0
0	0	0	0	0	0	0	0	0	0
0	0	0	0	0	0	0	0	0	0
0	0	0	0	0	0	0	0	0	0
0	0	0	0	0	0	0	0	0	0
0	0	0	0	0	0	0	0	0	0
0	0	0	0	0	0	0	0	0	0
0	0	0	0	0	0	0	0	0	0
0	0	0	0	0	0	0	0	0	0
0	0	0	0	0	0	0	0	0	0

0	0	0	4	0	0	0	0	0	0
0	0	8	0	0	0	0	0	0	0
0	0	0	0	0	0	0	0	0	0
0	0	0	0	0	0	0	0	0	0
0	0	0	0	0	0	0	0	0	0
0	0	0	0	0	0	0	0	0	0
0	0	0	0	0	0	0	0	0	0
0	0	0	0	0	0	0	0	0	0
0	0	0	0	0	0	0	0	0	0
0	0	0	0	0	0	0	0	0	0
0	0	0	0	0	0	0	0	0	0
0	0	0	0	0	0	0	0	0	0
0	0	0	0	0	0	0	0	0	0
14	0	0	0	0	0	0	0	0	0
6	0	0	0	0	8	0	0	0	0
4	0	10	0	0	0	0	0	0	0
0	14	0	0	0	0	0	0	0	0
0	11	0	0	3	0	0	0	0	0
0	0	0	14	0	0	0	0	0	0
0	0	0	14	0	0	0	0	0	0
0	0	0	14	0	0	0	0	0	0
0	0	0	14	0	0	0	0	0	0
0	0	0	14	0	0	0	0	0	0
0	0	0	0	0	0	0	0	0	0
0	0	0	0	0	0	0	0	0	0
0	0	0	0	0	0	0	0	0	0
0	0	0	0	0	0	0	0	0	0
0	0	0	0	0	0	0	0	0	0
0	0	0	0	0	0	0	0	0	0
0	0	0	0	0	0	0	0	0	0
0	0	0	0	0	0	0	0	0	0
0	0	0	0	0	0	0	0	0	0
0	0	0	0	0	0	0	0	0	0
0	0	0	0	0	0	0	0	0	0
0	0	0	0	0	0	0	0	0	0
0	0	0	0	0	0	0	0	0	0
0	0	0	0	0	0	0	0	0	0
0	13	0	0	0	0	0	0	0	0
0	13	0	0	0	0	0	0	0	0
0	13	0	0	0	0	0	0	0	0
0	4	6	0	0	0	0	0	0	0
0	0	2	11	0	0	0	0	0	0
0	0	0	13	0	0	0	0	0	0
0	0	0	13	0	0	0	0	0	0
0	0	0	13	0	0	0	0	0	0
0	0	0	13	0	0	0	0	0	0
0	0	0	13	0	0	0	0	0	0
0	0	0	0	13	0	0	0	0	0
0	0	0	0	0	13	0	0	0	0
0	0	0	0	0	0	0	0	0	0

0	0	0	0	0	0	0	0	0	0
0	0	0	0	0	0	0	0	0	0
0	0	0	0	0	0	0	0	0	0
0	0	0	0	0	0	0	0	0	0
0	0	0	0	0	0	0	0	0	0
0	0	0	0	0	0	0	0	0	0
0	0	0	0	0	0	0	0	0	0
0	0	0	0	0	0	0	0	0	0
0	0	0	0	0	0	0	0	0	0
0	0	0	0	0	0	0	0	0	0
0	0	0	0	0	0	0	0	0	0
0	0	0	0	0	0	0	0	0	0
0	0	0	0	0	0	0	0	0	0
0	0	0	0	0	0	0	0	0	0
0	0	0	0	0	0	0	0	0	0
0	0	0	0	0	0	0	0	0	0
0	0	0	0	0	0	0	0	0	0
0	0	0	0	0	0	0	0	0	0
0	0	0	0	0	0	0	0	0	0
0	0	0	6	0	0	0	0	0	0
0	0	0	0	0	0	0	0	0	0
0	12	0	0	0	0	0	0	0	0
0	12	0	0	0	0	0	0	0	0
0	12	0	0	0	0	0	0	0	0
0	7	5	0	0	0	0	0	0	0
0	0	12	0	0	0	0	0	0	0
0	0	12	0	0	0	0	0	0	0
0	0	12	0	0	0	0	0	0	0
0	0	0	12	0	0	0	0	0	0
0	0	0	12	0	0	0	0	0	0
0	0	0	12	0	0	0	0	0	0
0	0	0	12	0	0	0	0	0	0
0	0	0	3	9	0	0	0	0	0
0	0	0	0	0	12	0	0	0	0
0	0	0	0	0	12	0	0	0	0
0	0	0	0	0	0	4	0	8	0
0	0	0	0	0	0	0	0	0	0
0	0	0	0	0	0	0	0	0	0
0	0	0	0	0	0	0	0	0	0
0	0	0	0	0	0	0	0	0	0
0	0	0	0	0	0	0	0	0	0
0	0	0	0	0	0	0	0	0	0
0	0	0	0	0	0	0	0	0	0
0	0	0	0	0	0	0	0	0	0
0	0	0	0	0	0	0	0	0	0
0	0	0	0	0	0	0	0	0	0
0	0	0	0	0	0	0	0	0	0
0	0	0	0	0	0	0	0	0	0
0	0	0	0	0	0	0	0	0	0
0	0	0	0	0	0	0	0	0	0

0	0	0	0	0	0	0	0	0	0
0	0	0	0	0	0	0	0	0	0
0	0	0	0	0	0	0	0	0	0
11	0	0	0	0	0	0	0	0	0
0	11	0	0	0	0	0	0	0	0
0	0	11	0	0	0	0	0	0	0
0	0	11	0	0	0	0	0	0	0
0	0	2	0	0	9	0	0	0	0
0	0	0	11	0	0	0	0	0	0
0	0	0	11	0	0	0	0	0	0
0	0	0	7	0	4	0	0	0	0
0	0	0	0	0	11	0	0	0	0
0	0	0	0	0	11	0	0	0	0
0	0	0	0	0	0	0	0	11	0
0	0	0	0	0	0	0	0	0	0
0	0	0	0	0	0	0	0	0	0
0	0	0	0	0	0	0	0	0	0
0	0	0	0	0	0	0	0	0	0
0	0	0	0	0	0	0	0	0	0
0	0	0	0	0	0	0	0	0	0
0	0	0	0	0	0	0	0	0	0
0	0	0	0	0	0	0	0	0	0
0	0	0	0	0	0	0	0	0	0
0	0	0	0	0	0	0	0	0	0
0	0	0	0	0	0	0	0	0	0
0	0	0	0	0	0	0	0	0	0
0	0	0	0	0	0	0	0	0	0
0	0	0	0	0	0	0	0	0	0
0	0	0	0	0	0	0	0	0	0
0	0	0	0	0	0	0	0	0	0
0	0	0	0	0	0	0	0	0	0
0	0	0	0	0	0	0	0	0	0
0	0	0	0	0	0	0	0	0	0
0	0	0	0	0	0	0	0	0	0
0	10	0	0	0	0	0	0	0	0
0	10	0	0	0	0	0	0	0	0
0	10	0	0	0	0	0	0	0	0
0	10	0	0	0	0	0	0	0	0
0	5	0	5	0	0	0	0	0	0
0	0	0	10	0	0	0	0	0	0
0	0	0	10	0	0	0	0	0	0
0	0	0	10	0	0	0	0	0	0
0	0	0	10	0	0	0	0	0	0
0	0	0	0	0	10	0	0	0	0
0	0	0	0	0	0	0	10	0	0
0	0	0	0	0	0	0	0	0	0
0	0	0	0	0	0	0	0	0	0
0	0	0	0	0	0	0	0	0	0
0	0	0	0	0	0	0	0	0	0
0	0	0	0	0	0	0	0	0	0

0	0	0	0	0	0	0	0	0	0
0	0	0	0	0	0	0	0	0	0
0	0	0	0	0	0	0	0	0	0
0	0	0	0	0	0	0	0	0	0
0	0	0	0	0	0	0	0	0	0
0	0	0	0	0	0	0	0	0	0
0	0	0	0	0	0	0	0	0	0
0	0	0	0	0	0	0	0	0	0
0	0	0	0	0	0	0	0	0	0
0	0	0	0	0	0	0	0	0	0
0	0	0	0	0	0	0	0	0	0
0	0	0	0	0	0	0	0	0	0
0	0	0	6	0	0	0	0	0	0
0	0	0	0	0	0	0	0	0	0
0	0	0	0	0	0	0	0	0	0
0	0	0	0	0	0	0	0	0	0
0	0	0	0	0	0	0	0	0	0
0	0	0	0	0	0	0	0	0	0
0	0	0	5	0	0	0	0	0	0
0	9	0	0	0	0	0	0	0	0
0	9	0	0	0	0	0	0	0	0
0	9	0	0	0	0	0	0	0	0
0	9	0	0	0	0	0	0	0	0
0	9	0	0	0	0	0	0	0	0
0	0	9	0	0	0	0	0	0	0
0	0	9	0	0	0	0	0	0	0
0	0	9	0	0	0	0	0	0	0
0	0	0	9	0	0	0	0	0	0
0	0	0	9	0	0	0	0	0	0
0	0	0	0	0	9	0	0	0	0
0	0	0	0	0	9	0	0	0	0
0	0	0	0	0	6	3	0	0	0
0	0	0	0	0	0	0	9	0	0
0	0	0	0	0	0	0	0	0	0
0	0	0	0	0	0	0	0	0	0
0	0	0	0	0	0	0	0	0	0
0	0	0	0	0	0	0	0	0	0
0	0	0	0	0	0	0	0	0	0
0	0	0	0	0	0	0	0	0	0
0	0	0	0	0	0	0	0	0	0
0	0	0	0	0	0	0	0	0	0
0	0	0	0	0	0	0	0	0	0
0	0	0	0	0	0	0	0	0	0
0	0	0	0	0	0	0	0	0	0
0	0	0	0	0	0	0	0	0	0
0	0	0	0	0	0	0	0	0	0
0	0	0	0	0	0	0	0	0	0
0	0	0	0	0	0	0	0	0	0
0	0	0	0	6	0	0	0	0	0
5	0	0	0	0	0	0	0	0	0

0	8	0	0	0	0	0	0	0	0
0	8	0	0	0	0	0	0	0	0
0	0	8	0	0	0	0	0	0	0
0	0	8	0	0	0	0	0	0	0
0	0	8	0	0	0	0	0	0	0
0	0	8	0	0	0	0	0	0	0
0	0	8	0	0	0	0	0	0	0
0	0	0	8	0	0	0	0	0	0
0	0	0	8	0	0	0	0	0	0
0	0	0	8	0	0	0	0	0	0
0	0	0	6	0	2	0	0	0	0
0	0	0	0	8	0	0	0	0	0
0	0	0	0	5	0	0	0	3	0
0	0	0	0	0	8	0	0	0	0
0	0	0	0	0	8	0	0	0	0
0	0	0	0	0	4	0	0	2	0
0	0	0	0	0	0	0	0	0	0
0	0	0	0	0	0	0	0	0	0
0	0	0	0	0	0	0	0	0	0
0	0	0	0	0	0	0	0	0	0
0	0	0	0	0	0	0	0	0	0
0	0	0	0	0	0	0	0	0	0
0	0	0	0	0	0	0	0	0	0
0	0	0	0	0	0	0	0	0	0
0	0	0	0	0	0	0	0	0	0
0	0	0	0	0	0	0	0	0	0
0	0	0	0	0	0	0	0	0	0
0	0	0	0	0	0	0	0	0	0
0	0	0	0	0	0	0	0	0	0
0	0	0	0	0	0	0	0	0	0
0	0	0	0	0	0	0	0	0	0
0	0	0	0	0	0	0	0	0	0
7	0	0	0	0	0	0	0	0	0
7	0	0	0	0	0	0	0	0	0
7	0	0	0	0	0	0	0	0	0
7	0	0	0	0	0	0	0	0	0
7	0	0	0	0	0	0	0	0	0
0	7	0	0	0	0	0	0	0	0
0	0	7	0	0	0	0	0	0	0
0	0	7	0	0	0	0	0	0	0
0	0	7	0	0	0	0	0	0	0
0	0	0	7	0	0	0	0	0	0
0	0	0	7	0	0	0	0	0	0
0	0	0	7	0	0	0	0	0	0
0	0	0	7	0	0	0	0	0	0
0	0	0	7	0	0	0	0	0	0
0	0	0	7	0	0	0	0	0	0
0	0	0	7	0	0	0	0	0	0
0	0	0	0	7	0	0	0	0	0
0	0	0	0	0	7	0	0	0	0

0	0	0	0	0	7	0	0	0	0
0	0	0	0	0	7	0	0	0	0
0	0	0	0	0	0	0	0	0	0
0	0	0	0	0	0	0	0	0	0
0	0	0	0	0	0	0	0	0	0
0	0	0	0	0	0	0	0	0	0
0	0	0	0	0	0	0	0	0	0
0	0	0	0	0	0	0	0	0	0
0	0	0	0	0	0	0	0	0	0
0	0	0	0	0	0	0	0	0	0
0	0	0	0	0	0	0	0	0	0
6	0	0	0	0	0	0	0	0	0
3	0	0	0	0	3	0	0	0	0
0	6	0	0	0	0	0	0	0	0
0	6	0	0	0	0	0	0	0	0
0	6	0	0	0	0	0	0	0	0
0	6	0	0	0	0	0	0	0	0
0	6	0	0	0	0	0	0	0	0
0	3	3	0	0	0	0	0	0	0
0	0	6	0	0	0	0	0	0	0
0	0	6	0	0	0	0	0	0	0
0	0	6	0	0	0	0	0	0	0
0	0	6	0	0	0	0	0	0	0
0	0	0	6	0	0	0	0	0	0
0	0	0	6	0	0	0	0	0	0
0	0	0	6	0	0	0	0	0	0
0	0	0	6	0	0	0	0	0	0
0	0	0	6	0	0	0	0	0	0
0	0	0	0	6	0	0	0	0	0
0	0	0	0	6	0	0	0	0	0
0	0	0	0	0	6	0	0	0	0
0	0	0	0	0	6	0	0	0	0
0	0	0	0	0	6	0	0	0	0
0	0	0	0	0	0	0	6	0	0
0	0	0	0	0	0	0	6	0	0
0	0	0	0	0	0	0	0	0	0
0	0	0	0	0	0	0	0	0	0
0	0	0	0	0	0	0	0	0	0
0	0	0	0	0	0	0	0	0	0
0	0	0	0	0	0	0	0	0	0
0	0	0	0	0	0	0	0	0	0
0	0	0	0	0	0	0	0	0	0
0	0	0	0	0	0	0	0	0	0
0	0	0	0	0	0	0	0	0	0
0	0	0	0	0	0	0	0	0	0
0	0	0	0	0	0	0	0	0	0
0	0	0	0	0	0	0	0	0	0
0	0	0	0	0	0	0	0	0	0
0	0	0	0	0	0	0	0	0	0
0	0	0	0	0	0	0	0	0	0

3	0	0	0	0	2	0	0	0	0
2	0	0	3	0	0	0	0	0	0
0	5	0	0	0	0	0	0	0	0
0	5	0	0	0	0	0	0	0	0
0	0	5	0	0	0	0	0	0	0
0	0	5	0	0	0	0	0	0	0
0	0	5	0	0	0	0	0	0	0
0	0	5	0	0	0	0	0	0	0
0	0	0	5	0	0	0	0	0	0
0	0	0	3	0	0	0	0	0	0
0	0	0	3	0	2	0	0	0	0
0	0	0	0	5	0	0	0	0	0
0	0	0	0	5	0	0	0	0	0
0	0	0	0	0	5	0	0	0	0
0	0	0	0	0	5	0	0	0	0
0	0	0	0	0	5	0	0	0	0
0	0	0	0	0	0	5	0	0	0
0	0	0	0	0	0	0	0	0	0
0	0	0	0	0	0	0	0	0	0
0	0	0	0	0	0	0	0	0	0
0	0	0	0	0	0	0	0	0	0
0	0	0	0	0	0	0	0	0	0
0	0	0	0	0	2	0	0	0	0
0	0	0	0	0	0	0	0	0	0
0	0	0	0	0	0	0	0	0	0
0	0	0	0	0	0	0	0	0	0
0	0	0	0	0	0	0	0	0	0
0	0	0	0	0	0	0	0	0	0
0	0	0	0	0	0	0	0	0	0
4	0	0	0	0	0	0	0	0	0
4	0	0	0	0	0	0	0	0	0
0	4	0	0	0	0	0	0	0	0
0	4	0	0	0	0	0	0	0	0
0	0	4	0	0	0	0	0	0	0
0	0	4	0	0	0	0	0	0	0
0	0	0	4	0	0	0	0	0	0
0	0	0	4	0	0	0	0	0	0
0	0	0	0	4	0	0	0	0	0
0	0	0	0	4	0	0	0	0	0
0	0	0	0	0	4	0	0	0	0
0	0	0	0	0	4	0	0	0	0
0	0	0	0	0	4	0	0	0	0
0	0	0	0	0	4	0	0	0	0
0	0	0	0	0	0	0	4	0	0
0	0	0	0	0	0	0	0	4	0
0	0	0	0	0	0	0	0	4	0
0	0	0	0	0	0	0	0	0	0
0	0	0	0	0	0	0	0	0	0
0	0	0	0	0	0	0	0	0	0
0	0	0	0	0	0	0	0	0	0

0	0	0	0	0	0	0	0	0	0
0	0	0	0	0	0	0	0	0	0
0	0	0	0	0	0	0	0	0	0
0	0	0	0	0	0	0	0	0	0
0	0	0	0	0	0	0	0	0	0
0	0	0	0	0	0	0	0	0	0
0	0	0	0	0	0	0	0	0	0
0	0	0	0	0	0	0	0	0	0
0	0	0	0	0	0	0	0	0	0
0	0	0	0	0	0	0	0	0	0
0	0	0	0	0	0	0	0	0	0
0	0	0	0	0	0	0	0	0	0
0	0	0	0	0	0	0	0	0	0
0	0	0	0	0	0	0	0	0	0
0	0	0	0	0	0	0	0	0	0
0	0	0	0	0	0	0	0	0	0
0	0	0	0	0	0	0	0	0	0
0	0	0	0	0	0	0	0	0	0
0	0	0	0	0	0	0	0	0	0
0	0	0	0	0	0	0	0	0	0
0	0	0	0	0	0	0	0	0	0
0	0	0	0	0	0	0	0	0	0
0	0	0	0	0	0	0	0	0	0
0	0	0	0	0	0	0	0	0	0
0	0	0	0	0	0	0	0	0	0
0	0	0	0	0	0	0	0	0	0
0	0	0	0	0	0	0	0	0	0
0	0	0	0	0	0	0	0	0	0
3	0	0	0	0	0	0	0	0	0
3	0	0	0	0	0	0	0	0	0
3	0	0	0	0	0	0	0	0	0
0	3	0	0	0	0	0	0	0	0
0	3	0	0	0	0	0	0	0	0
0	3	0	0	0	0	0	0	0	0
0	3	0	0	0	0	0	0	0	0
0	0	3	0	0	0	0	0	0	0
0	0	3	0	0	0	0	0	0	0
0	0	3	0	0	0	0	0	0	0
0	0	3	0	0	0	0	0	0	0
0	0	3	0	0	0	0	0	0	0
0	0	0	3	0	0	0	0	0	0
0	0	0	3	0	0	0	0	0	0
0	0	0	3	0	0	0	0	0	0
0	0	0	3	0	0	0	0	0	0
0	0	0	3	0	0	0	0	0	0
0	0	0	3	0	0	0	0	0	0
0	0	0	3	0	0	0	0	0	0
0	0	0	3	0	0	0	0	0	0
0	0	0	3	0	0	0	0	0	0
0	0	0	3	0	0	0	0	0	0

0	0	0	3	0	0	0	0	0	0
0	0	0	0	3	0	0	0	0	0
0	0	0	0	3	0	0	0	0	0
0	0	0	0	3	0	0	0	0	0
0	0	0	0	3	0	0	0	0	0
0	0	0	0	3	0	0	0	0	0
0	0	0	0	3	0	0	0	0	0
0	0	0	0	0	3	0	0	0	0
0	0	0	0	0	3	0	0	0	0
0	0	0	0	0	3	0	0	0	0
0	0	0	0	0	3	0	0	0	0
0	0	0	0	0	3	0	0	0	0
0	0	0	0	0	3	0	0	0	0
0	0	0	0	0	3	0	0	0	0
0	0	0	0	0	3	0	0	0	0
0	0	0	0	0	3	0	0	0	0
0	0	0	0	0	3	0	0	0	0
0	0	0	0	0	0	0	3	0	0
0	0	0	0	0	0	0	3	0	0
0	0	0	0	0	0	0	0	3	0
0	0	0	0	0	0	0	0	3	0
0	0	0	0	0	0	0	0	3	0
0	0	0	0	0	0	0	0	0	0
0	0	0	0	0	0	0	0	0	0
0	0	0	0	0	0	0	0	0	0
0	0	0	0	0	0	0	0	0	0
0	0	0	0	0	0	0	0	0	0
0	0	0	0	0	0	0	0	0	0
0	0	0	0	0	0	0	0	0	0
0	0	0	0	0	0	0	0	0	0
0	0	0	0	0	0	0	0	0	0
0	0	0	0	0	0	0	0	0	0
0	0	0	0	0	0	0	0	0	0
0	0	0	0	0	0	0	0	0	0
0	0	0	0	0	0	0	0	0	0
0	0	0	0	0	0	0	0	0	0
0	0	0	0	0	0	0	0	0	0
0	0	0	0	0	0	0	0	0	0
0	0	0	0	0	0	0	0	0	0
0	0	0	0	0	0	0	0	0	0
0	0	0	0	0	0	0	0	0	0
0	0	0	0	0	0	0	0	0	0
0	0	0	0	0	0	0	0	0	0
0	0	0	0	0	0	0	0	0	0
0	0	0	0	0	0	0	0	0	0
0	0	0	0	0	0	0	0	0	0
0	0	0	0	0	0	0	0	0	0
0	0	0	0	0	0	0	0	0	0
0	0	0	0	0	0	0	0	0	0
0	0	0	0	0	0	0	0	0	0

0	0	0	0	0	0	0	0	0	0
0	0	0	0	0	0	0	0	0	0
0	0	0	0	0	0	0	0	0	0
0	0	0	0	0	0	0	0	0	0
0	0	0	0	0	0	0	0	0	0
0	0	0	0	0	0	0	0	0	0
0	0	0	0	0	0	0	0	0	0
0	0	0	0	0	0	0	0	0	0
0	0	0	0	0	0	0	0	0	0
0	0	0	0	0	0	0	0	0	0
0	0	0	0	0	0	0	0	0	0
0	0	0	0	0	0	0	0	0	0
0	0	0	0	0	0	0	0	0	0
0	0	0	0	0	0	0	0	0	0
0	0	0	0	0	0	0	0	0	0
2	0	0	0	0	0	0	0	0	0
2	0	0	0	0	0	0	0	0	0
0	2	0	0	0	0	0	0	0	0
0	2	0	0	0	0	0	0	0	0
0	2	0	0	0	0	0	0	0	0
0	0	2	0	0	0	0	0	0	0
0	0	2	0	0	0	0	0	0	0
0	0	2	0	0	0	0	0	0	0
0	0	2	0	0	0	0	0	0	0
0	0	2	0	0	0	0	0	0	0
0	0	2	0	0	0	0	0	0	0
0	0	2	0	0	0	0	0	0	0
0	0	2	0	0	0	0	0	0	0
0	0	0	2	0	0	0	0	0	0
0	0	0	2	0	0	0	0	0	0
0	0	0	2	0	0	0	0	0	0
0	0	0	2	0	0	0	0	0	0
0	0	0	2	0	0	0	0	0	0
0	0	0	2	0	0	0	0	0	0
0	0	0	2	0	0	0	0	0	0
0	0	0	2	0	0	0	0	0	0
0	0	0	2	0	0	0	0	0	0
0	0	0	2	0	0	0	0	0	0
0	0	0	0	2	0	0	0	0	0
0	0	0	0	2	0	0	0	0	0
0	0	0	0	2	0	0	0	0	0
0	0	0	0	2	0	0	0	0	0
0	0	0	0	2	0	0	0	0	0
0	0	0	0	2	0	0	0	0	0
0	0	0	0	0	2	0	0	0	0
0	0	0	0	0	2	0	0	0	0
0	0	0	0	0	2	0	0	0	0
0	0	0	0	0	2	0	0	0	0
0	0	0	0	0	2	0	0	0	0
0	0	0	0	0	2	0	0	0	0

0	0	0	0	0	2	0	0	0	0
0	0	0	0	0	2	0	0	0	0
0	0	0	0	0	2	0	0	0	0
0	0	0	0	0	2	0	0	0	0
0	0	0	0	0	2	0	0	0	0
0	0	0	0	0	2	0	0	0	0
0	0	0	0	0	2	0	0	0	0
0	0	0	0	0	2	0	0	0	0
0	0	0	0	0	2	0	0	0	0
0	0	0	0	0	0	0	2	0	0
0	0	0	0	0	0	0	2	0	0
0	0	0	0	0	0	0	2	0	0
0	0	0	0	0	0	0	2	0	0
0	0	0	0	0	0	0	2	0	0
0	0	0	0	0	0	0	0	2	0
0	0	0	0	0	0	0	0	2	0
0	0	0	0	0	0	0	0	2	0
0	0	0	0	0	0	0	0	2	0
0	0	0	0	0	0	0	0	2	0
0	0	0	0	0	0	0	0	0	0
0	0	0	0	0	0	0	0	0	0
0	0	0	0	0	0	0	0	0	0
0	0	0	1	0	0	0	0	0	0

SF1697	SF1753
72963	79829
1060	726
244	231
112	119
449	910
36476	137
170	203
139	151
86	185
564	959
203	139
62	38
13	0
1821	2133
92	95
94	83
119	99
525	236
152	1925
62	221
124	67
59	72
77	51
53	47
80	92
10	15
114	37
1231	2451
451	98
0	29
33	0
0	0
12	817
0	0
30	0
0	0
25	16
0	0
0	34
102	42
0	0
31	47
0	0
22	39
0	32
4	0
0	0
0	35

0	0
14	26
94	197
0	106
58	0
0	0
0	27
0	0
0	0
16	8
8	24
0	0
18	29
11	16
19	0
0	0
0	166
0	0
0	0
0	0
1048	13
0	0
63	0
350	64
62	85
0	8
302	28
0	0
0	0
0	0
24	0
37	0
0	15
0	0
34	47
0	29
0	41
47	0
0	0
38	0
0	0
0	0
0	0
0	0
0	0
0	0
0	0
0	0
0	0
11	0

0	0
0	987
0	0
137	0
0	0
11	0
0	0
0	0
0	0
0	0
0	0
6	0
0	0
0	0
0	0
0	0
0	0
0	0
0	0
0	0
0	0
0	0
0	0
0	0
196	53
0	0
3	4
15	0
105	0
0	38
0	0
0	0
0	0
0	11
0	0
4	11
0	0
8	0
0	0
0	0
0	71
0	18
0	0
577	0
0	0
0	251
0	0
9	23
0	0
0	0

0	0
9	12
0	0
0	0
0	0
0	0
0	0
0	0
4	0
7	18
0	0
0	0
6	13
0	9
0	0
0	0
0	0
0	52
0	0
0	0
0	0
8	14
0	0
0	0
0	0
0	0
0	0
0	0
0	0
13	44
0	0
0	0
83	0
0	0
0	0
0	0
0	126
0	0
0	0
0	0
0	0
0	0
230	86
0	0
0	0
0	0
0	0
0	0
0	0
0	5

0	0
0	0
0	0
0	0
0	0
6	13
3	0
0	0
0	0
128	0
0	0
0	0
0	0
0	15
0	0
0	0
0	0
0	0
0	0
0	0
0	0
115	87
0	0
0	0
0	0
13	0
0	0
0	0
0	0
0	0
0	0
22	0
0	0
0	0
0	0
0	0
0	0
0	0
12	4
0	0
0	0
48	0
0	0
0	0
0	0
0	0
0	0
7	11
0	0
0	0

0	0
0	0
11	26
0	0
0	0
0	0
0	0
0	0
0	13
0	0
0	0
0	0
0	0
0	222
0	223
0	16
0	0
0	0
16	3
0	0
0	4
0	49
185	0
0	0
0	0
0	0
0	0
0	0
0	0
0	0
0	0
0	0
0	7
0	0
12	6
0	0
0	0
0	5
0	0
0	0
0	0
0	0
0	0
0	0
0	0
0	0
0	0
0	0
0	5
0	0

0	0
0	0
0	0
0	0
0	0
0	0
0	0
0	0
0	7
0	0
0	0
0	0
0	0
0	0
0	0
0	0
0	0
0	0
0	0
0	0
0	0
0	37
0	0
0	0
5	0
0	0
0	0
46	0
0	0
0	0
0	0
0	0
0	0
0	6
0	0
0	0
0	0
0	0
0	0
0	0
0	0
0	0
0	0
0	0
0	0
0	0
0	47
0	0
0	0
0	0

0	0
0	0
0	0
0	0
0	0
0	0
0	0
19	0
0	0
0	0
4	0
0	0
0	0
0	0
0	79
4	0
0	0
0	0
0	0
0	0
0	5
0	0
0	0
0	0
0	0
0	0
0	100
0	0
0	0
0	0
0	0
0	0
0	98
0	0
0	97
0	0
0	0
0	0
0	0
0	0
0	0
0	0
0	0
0	0
0	0
0	0
0	0
0	0
0	0
0	0

0	0
0	0
0	0
0	0
0	0
0	0
0	0
0	0
0	0
0	0
0	0
0	0
0	0
0	0
0	0
0	0
0	0
0	0
0	0
0	0
0	0
0	0
0	0
0	0
0	0
0	0
4	0
0	0
0	0
0	0
0	0
0	0
0	0
0	0
0	0
4	0
74	0
0	0
0	0
0	0
0	0
0	0
0	0
0	0
0	0
0	0
0	0
0	0
0	0
0	0

0	0
0	0
0	0
0	0
0	0
0	5
0	0
0	0
0	0
0	0
0	0
0	0
0	0
0	0
0	0
0	66
0	0
0	0
0	0
0	0
0	0
0	0
0	0
0	0
0	0
0	0
0	0
0	4
0	0
0	0
0	0
0	0
0	0
0	0
2	0
0	0
0	0
0	0
0	0
0	0
0	0
0	0
0	0
0	0
0	0
0	0
0	0
58	0
54	4
0	0

0	35
0	0
0	0
0	0
0	0
0	0
56	0
0	0
0	0
0	0
0	0
0	0
0	45
0	0
0	0
0	6
0	0
0	0
0	0
8	0
0	0
0	0
0	0
0	0
0	0
0	0
0	0
0	0
53	0
0	0
0	0
0	0
13	3
0	0
0	0
0	0
0	0
0	0
0	0
0	0
0	0
0	0
0	0
50	0
0	0
0	0
0	0
0	0
0	0
0	0

0	0
0	0
0	0
0	0
0	0
0	0
0	0
0	0
0	0
0	0
0	0
0	0
0	0
0	0
0	0
0	0
0	46
0	0
0	0
0	0
0	0
0	0
0	0
0	0
0	0
0	0
0	0
0	0
5	26
0	0
0	0
0	0
0	0
0	0
0	0
0	0
0	0
0	0
0	0
0	0
0	0
0	0
0	0
0	0
0	0
0	0
0	0
0	0
0	0
4	0

0	0
0	0
0	0
0	0
0	3
0	0
0	0
0	0
0	0
0	0
0	3
0	0
0	0
0	0
0	0
0	0
0	0
0	0
38	0
0	0
0	0
0	0
0	0
0	0
0	0
0	0
0	0
0	0
0	0
0	0
0	0
0	0
0	0
0	0
0	0
0	0
0	0
0	0
0	0
0	0
0	0
0	0
0	0
0	0
0	0
0	0
0	0
0	0
0	0
0	0

0	0
0	0
0	0
0	0
0	0
0	0
0	0
0	0
33	0
0	0
0	0
0	0
0	0
0	0
0	0
0	12
0	0
0	0
0	0
0	0
0	0
0	0
0	0
0	0
0	0
0	0
0	0
0	0
0	0
0	0
0	0
0	0
0	0
0	0
0	0
0	0
0	0
0	0
0	0
0	0
0	0
0	0
0	0
0	0
0	0
29	0
0	0
0	0
0	0
0	0

0	0
0	0
0	0
0	0
0	0
0	0
0	0
0	4
0	0
0	0
0	0
0	0
0	0
0	0
0	0
0	0
0	0
0	0
0	0
0	0
0	0
0	15
27	0
0	0
0	0
0	0
0	0
0	0
0	0
0	0
0	0
0	0
0	0
0	0
0	0
0	0
0	0
0	0
0	0
0	0
0	0
0	0
0	0
0	0
0	0
0	0
0	0
0	0
0	0
0	0

0	0
25	0
0	0
0	0
0	0
0	0
0	0
0	0
0	0
0	0
0	0
0	0
0	0
0	0
0	0
0	0
0	0
0	0
0	0
0	0
0	0
0	0
0	0
0	0
0	0
0	0
0	0
0	0
0	0
0	0
0	0
0	0
0	0
0	0
0	0
0	0
0	0
22	0
0	0
0	0
0	0
0	0
0	9
0	0
0	0
0	0
0	0
0	0
0	0
0	0

0	0
0	0
0	0
0	0
0	0
0	0
0	0
0	0
0	21
0	21
0	0
0	0
0	0
0	0
0	0
0	0
0	0
0	0
0	0
0	0
0	0
0	0
0	0
0	0
3	0
0	0
0	0
0	0
0	0
0	0
0	0
0	0
0	0
0	0
0	0
0	0
0	0
0	0
0	0
0	0
0	0
0	0
0	0
0	0
0	0
0	0
0	0
0	0
0	0
0	0

0	0
0	0
0	0
0	0
0	0
0	0
0	0
0	0
0	0
0	0
0	0
0	0
0	0
0	0
0	0
0	0
0	0
0	0
0	0
0	0
0	0
0	0
0	0
18	0
0	18
0	0
0	0
0	0
0	0
0	0
0	0
0	0
0	0
0	0
0	0
0	0
0	0
0	0
0	0
0	0
0	0
0	0
0	0
0	0
2	0
0	0
0	0
0	0
0	0
17	0

17	0
0	0
0	0
0	0
0	0
0	0
0	0
0	0
0	0
0	0
0	0
0	0
0	0
0	0
0	0
0	0
0	0
0	0
0	0
0	0
0	0
0	0
0	2
0	0
16	0
16	0
0	16
0	0
0	0
0	0
0	0
0	0
0	0
0	0
0	0
0	0
0	0
0	7
0	0
0	0
0	0
0	0
0	0
0	0
0	0
0	0
0	0
0	0
0	0
0	0

0	0
0	0
0	0
0	0
0	0
0	0
0	0
0	0
0	0
0	0
0	0
0	0
0	0
0	4
0	0
0	0
0	0
0	0
0	0
0	0
0	0
0	0
0	0
0	0
0	0
0	0
0	0
0	0
0	0
0	0
0	0
0	0
0	0
0	0
0	0
0	0
0	0
0	0
0	0
0	0
0	0
0	0
0	0
0	0
0	0
0	0
14	0
0	0
0	0
0	0

0	0
0	0
0	0
0	0
0	0
0	0
0	0
0	0
0	0
0	0
0	0
0	0
0	0
0	0
0	0
0	0
0	0
0	0
0	0
0	0
0	0
0	0
0	0
0	0
0	0
0	0
0	0
0	13
0	0
0	0
0	0
0	0
0	0
0	0
0	0
0	0
0	0
0	0
0	0
0	0
0	0
0	0
0	0
0	0
0	0
0	0
0	0
0	0
0	0
0	0

0	0
0	0
0	0
0	0
0	0
0	0
0	0
12	0
0	12
0	12
0	0
0	0
0	0
0	0
0	0
0	2
0	0
0	0
0	0
0	0
0	0
0	0
0	5
0	0
0	0
0	0
0	0
0	0
0	0
0	0
0	0
0	0
0	0
0	0
0	0
0	0
0	0
0	0
0	0
0	0
0	11
0	11
0	0
0	0
0	0
0	0
0	0
0	0
0	0

0	0
0	0
0	0
0	0
0	0
0	0
0	0
0	0
0	0
0	0
0	0
0	0
0	0
0	0
0	0
0	0
0	0
0	0
0	0
0	0
0	0
0	0
0	0
0	0
0	0
0	0
0	0
0	0
0	3
0	0
0	0
0	0
0	0
0	0
0	0
0	0
0	0
0	0
0	0
0	0
0	10
0	0
0	0
0	0
0	0
0	0
0	0
0	0
0	0
0	0

0	0
0	0
0	0
0	0
0	0
0	0
0	0
0	0
0	0
0	0
0	0
0	3
0	0
0	0
0	0
0	0
0	0
0	0
0	0
0	0
0	0
0	0
0	0
0	0
0	0
0	0
9	0
0	9
0	9
0	0
0	0
0	0
0	0
0	0
0	0
0	0
0	0
0	0
0	0
0	0
0	0
0	0
0	0
0	0
0	0
0	0
0	0
0	0
0	0
0	0

0	0
0	0
0	0
0	0
0	0
0	0
0	0
0	0
0	0
0	0
0	0
0	0
0	0
0	0
0	0
0	0
0	0
0	0
0	0
0	0
8	0
8	0
8	0
0	0
0	0
0	0
0	0
0	0
0	0
0	0
0	0
0	0
0	0
0	0
0	0
0	0
0	0
0	0
0	0
0	0
0	0
0	0
0	0
0	0
0	0
0	0
0	0
0	0
0	0
0	0

0	0
0	0
0	0
0	0
0	0
0	0
0	0
0	0
0	0
0	0
0	0
0	0
0	0
0	0
0	0
0	0
0	0
0	0
0	0
0	0
0	0
0	0
0	0
0	0
0	0
0	0
0	0
0	0
0	0
0	0
0	7
0	7
0	0
0	0
0	0
0	0
0	0
0	0
0	0
0	0
0	0
0	0
0	0
0	0
0	0
0	0
0	0
0	0
0	0
0	0

0	0
0	0
0	0
0	0
0	0
0	0
0	0
0	0
0	0
0	0
0	0
0	0
0	0
0	0
0	0
0	0
0	0
0	0
0	0
0	0
0	0
0	0
0	0
0	0
0	0
0	0
0	0
0	0
0	0
0	0
0	0
0	0
0	0
0	0
0	0
0	0
0	0
0	0
0	0
0	0
6	0
0	6
0	0
0	0
0	0
0	0
0	0
0	0
0	0
0	0

0	0
0	0
0	0
0	0
0	0
0	0
0	0
0	0
0	0
0	0
0	0
0	0
0	0
0	0
0	0
0	0
0	0
0	0
0	0
0	0
0	0
0	0
0	0
0	0
0	0
0	0
0	0
0	0
0	0
0	0
0	0
0	0
0	0
0	0
0	0
0	0
0	0
0	0
0	0
0	0
0	0
0	0
0	0
0	0
0	0
0	0
0	0
0	0
0	0
0	0

0	0
0	0
0	0
0	0
0	0
0	0
0	0
0	0
0	0
0	0
0	0
0	0
0	0
0	0
0	0
0	0
0	0
0	0
0	0
0	0
0	5
0	0
0	0
0	0
0	0
0	0
0	0
0	0
0	0
0	0
0	0
0	0
0	0
0	0
0	0
0	0
0	0
0	0
0	0
0	0
0	0
0	0
0	0
0	0
0	0
0	0
0	0
0	0
0	0
0	0

0	0
0	0
0	0
0	0
0	0
0	0
0	0
0	0
0	0
0	0
0	0
0	0
0	0
0	0
0	0
0	0
0	0
0	0
0	0
0	0
0	0
0	0
0	0
0	0
0	0
0	0
0	0
0	0
0	0
0	0
0	0
0	0
0	0
0	0
0	0
0	0
0	0
0	0
0	0
0	0
0	0
0	0
0	0
0	0
0	0
0	0
0	0
0	0
0	0
0	0

0	0
0	0
0	0
4	0
4	0
0	4
0	4
0	0
0	0
0	0
0	0
0	0
0	0
0	0
0	0
0	0
0	0
0	0
0	0
0	0
0	0
0	0
0	0
0	0
0	0
0	0
0	0
0	0
0	0
0	0
0	0
0	0
0	0
0	0
0	0
0	0
0	0
0	0
0	0
0	0
0	0
0	0
0	0
0	0
0	0
0	0
0	0
0	0
0	0
0	0

0	0
0	0
0	0
0	0
0	0
0	0
0	0
0	0
0	0
0	0
0	0
0	0
0	0
0	0
0	0
0	0
0	0
0	0
0	0
0	0
0	0
0	0
0	0
0	0
0	0
0	0
0	0
0	0
0	0
0	0
0	0
0	0
0	0
0	0
0	0
0	0
0	0
0	0
0	0
0	0
0	0
0	0
0	0
0	0
0	0
0	0
0	0
0	0
0	0
0	0

0	0
0	0
0	0
0	0
0	0
0	0
0	0
0	0
0	0
0	0
0	0
0	0
0	0
0	0
0	0
0	0
0	0
0	0
0	0
0	0
0	0
0	0
3	0
3	0
3	0
3	0
3	0
3	0
0	3
0	3
0	3
0	3
0	3
0	3
0	3
0	3
0	3
0	0
0	0
0	0
0	0
0	0
0	0
0	0
0	0
0	0
0	0
0	0
0	0
0	0

0	0
0	0
0	0
0	0
0	0
0	0
0	0
0	0
0	0
0	0
0	0
0	0
0	0
0	0
0	0
0	0
0	0
0	0
0	0
0	0
0	0
0	0
0	0
0	0
0	0
0	0
0	0
0	0
0	0
0	0
0	0
0	0
0	0
0	0
0	0
0	0
0	0
0	0
0	0
0	0
0	0
0	0
0	0
0	0
0	0
0	0
0	0
0	0
0	0
0	0

0	0
0	0
0	0
0	0
0	0
0	0
0	0
0	0
0	0
0	0
0	0
0	0
0	0
0	0
0	0
0	0
0	0
0	0
0	0
0	0
0	0
0	0
0	0
0	0
0	0
0	0
0	0
0	0
0	0
0	0
0	0
0	0
0	0
0	0
0	0
0	0
0	0
0	0
0	0
0	0
0	0
0	0
0	0
0	0
0	0
0	0
0	0
0	0
0	0
0	0

0	0
0	0
0	0
0	0
0	0
0	0
0	0
0	0
0	0
0	0
0	0
0	0
0	0
0	0
0	0
0	0
0	0
2	0
2	0
2	0
0	2
0	2
0	2
0	2
0	2
0	2
0	2
0	2
0	2
0	2
0	0
0	0
0	0
0	0
44	52
39	40
0	0
12	24
20	22
0	0
0	0
14	10
0	0
0	0
5	7
0	0
0	0
11	0
0	0
0	0

0	0
0	5
10	0
15	0
4	0
0	0
0	0
0	0
0	0
0	3
0	0
0	0
0	0
0	3
0	0
0	0
0	0
0	0
0	0
0	0
0	0
0	0
0	0
0	0
5	0
0	0
0	0
0	0
0	0
0	0
0	0
0	0
0	0
0	0
0	0
0	0
0	0
0	0
0	0
0	0
0	0
9	0
0	0
0	0
0	0
0	0
0	0
0	0
0	0
0	0

0	0
0	0
0	0
0	0
0	0
0	0
0	0
0	0
0	0
0	0
0	0
0	0
0	0
0	0
0	0
0	0
0	0
0	0
0	0
0	0
0	0
0	0
0	0
0	0
0	0
0	0
0	0
0	0
0	0
0	0
0	0
0	0
0	0
0	0
0	2
0	0
0	0
0	0
0	0
0	0
0	0
0	0
0	0
0	0
0	0
0	0
0	0
0	0
0	0
0	0

0	0
0	0
0	0
0	0
0	0
0	0
0	0
0	0
0	0
0	0
0	0
0	0
0	0
0	0
0	0
0	0
0	0
0	0
0	0
0	0
0	0
0	0
0	0
0	0
0	0
0	0
0	0
0	0
0	0
0	0
0	0
0	0
0	0
0	0
0	0
0	0
0	0
0	0
0	0
0	0
0	0
0	0
0	0
0	0
0	4
0	0
0	0
0	0
0	0
0	0

0	0
0	0
0	0
0	0
0	0
0	0
0	0
0	0
0	0
0	0
0	0
0	0
0	0
0	0
0	0
0	0
0	0
0	0
0	0
0	0
0	0
0	0
0	0
0	0
0	0
0	0
0	0
0	0
0	0
0	0
0	0
0	0
0	0
0	0
0	0
0	0
0	0
0	0
0	0
0	0
0	0
0	0
0	0
0	0
0	0
0	0
0	0
0	0
0	0
0	0

0	0
0	0
0	0
0	0
0	0
0	0
0	0
0	0
0	0
0	0
0	0
0	6
0	0
0	0
0	0
0	0
0	0
0	0
0	0
0	0
0	0
0	0
0	0
0	0
0	0
0	0
0	0
0	0
0	0
0	0
0	0
0	0
0	0
0	0
0	0
0	0
0	0
0	0
0	0
0	0
0	0
0	0
0	0
0	0
0	0
0	0
0	0
0	0
0	0
0	0

0	0
0	0
0	0
4	0
0	4
0	0
0	0
0	0
0	0
0	0
0	0
0	0
0	0
0	0
0	0
0	0
0	0
0	0
0	0
0	0
0	0
0	0
0	0
0	0
0	0
0	0
0	0
0	0
0	0
0	0
0	0
0	0
0	0
0	0
0	0
0	0
0	0
0	0
0	0
0	0
0	0
0	0
0	0
0	0
0	0
0	0
0	0
0	0
0	0
0	0

0	0
0	0
0	0
2	0
0	2
0	2
0	0
0	0
0	0
0	0
0	0
0	0
0	0
0	0
0	0
0	0
0	0
0	0
0	0
0	0
0	0
0	0
0	0
0	0
921	1110
69	43
0	0
0	0
0	0
93	146
72	129
60	0
0	0
0	0
28	26
0	0
23	43
0	0
21	41
0	0
0	0
0	0
0	0
0	0
0	0
0	0
14	0
0	0
0	0
0	0

0	0
0	0
0	0
0	0
104	75
0	0
0	0
0	0
0	0
0	0
0	0
0	0
0	0
0	0
0	0
0	0
0	0
0	0
0	0
0	0
0	0
0	0
0	0
6	0
0	0
0	0
6	0
0	0
4	0
0	0
0	0
16	33
0	0
0	0
0	0
0	0
0	0
0	0
0	0
0	0
0	0
0	0
0	0
51	57
0	0
0	0
0	0
0	0
0	0
0	0

0	0
0	0
0	0
0	0
0	0
0	0
0	0
0	0
0	0
0	0
0	0
0	0
0	0
0	0
0	0
0	0
0	0
0	0
0	0
0	0
0	0
0	0
0	0
0	0
0	0
0	0
0	0
0	0
0	0
0	0
0	0
0	0
0	0
0	0
0	0
0	0
0	0
0	0
0	0
0	0
0	0
0	0
0	0
5	0
0	0
0	0
0	0
0	0
0	0
0	0

0	0
0	0
0	0
0	0
0	0
92	85
0	0
0	0
0	0
0	0
0	0
0	0
0	0
0	0
0	0
0	0
0	0
0	0
0	0
0	0
0	0
0	0
0	0
0	0
0	0
0	0
0	0
0	0
0	0
0	0
0	0
0	0
0	0
0	0
0	0
0	0
0	0
0	0
0	0
0	0
0	0
0	0
0	0
0	0
0	0
0	0
0	0
0	0
0	0
0	0

0	0
0	0
0	0
0	0
0	0
0	0
0	0
0	0
0	0
0	0
0	0
0	0
0	0
0	0
0	0
0	0
0	0
0	0
0	0
0	0
0	0
0	0
0	0
0	0
0	0
0	0
0	0
0	0
3	0
0	0
0	9
0	0
0	0
0	0
0	0
0	0
0	0
0	0
0	0
0	0
0	0
0	0
0	0
0	0
0	0
0	0
0	0
0	0
0	0
0	0

0	0
0	0
0	0
0	0
0	0
0	0
45	44
0	0
0	0
0	0
0	0
0	0
0	0
0	0
0	0
0	0
0	0
0	0
0	0
0	0
0	0
0	0
0	0
0	0
0	0
0	0
0	0
0	0
0	0
0	0
0	0
0	0
0	0
0	0
0	0
0	0
0	0
0	0
0	0
0	0
0	0
0	0
0	0
0	0
0	0
0	0
0	0
0	0
0	0
0	0

0	0
0	0
0	0
0	0
0	0
0	0
0	0
0	0
0	0
0	0
0	0
0	0
0	0
0	0
0	0
0	0
0	0
0	0
0	0
0	0
0	0
0	0
0	0
0	0
0	0
0	0
0	0
0	0
0	0
0	0
0	0
0	0
0	0
0	0
0	0
0	0
0	0
0	0
0	0
0	0
0	0
0	0
0	0
0	0
0	0
0	0
0	0
0	0
0	0
0	0

0	0
0	0
0	0
0	0
10	0
0	0
0	0
0	0
0	0
0	0
0	0
0	0
0	0
0	0
0	0
0	0
0	0
0	0
0	0
0	0
0	0
0	0
0	0
0	0
0	0
0	0
0	0
0	0
0	0
0	0
0	0
0	0
0	0
0	0
0	0
0	0
0	0
0	0
0	0
0	0
0	0
0	0
0	0
0	0
0	0
0	0
0	0
0	0
0	0
0	0

0	0
0	0
0	0
0	0
0	0
0	0
0	0
0	0
0	0
0	0
0	0
0	0
0	0
0	0
0	0
0	0
0	0
0	0
0	0
0	0
0	0
0	0
0	0
0	0
0	0
0	0
0	0
0	0
0	0
0	0
0	0
0	0
0	0
0	0
0	0
0	0
0	0
0	0
0	0
0	0
0	0
0	0
0	0
0	0
0	0
0	0
0	0
0	0
0	0
0	0

0	0
0	0
0	0
0	0
0	0
0	0
0	0
0	0
0	0
0	0
0	0
0	0
0	0
0	0
0	0
0	0
0	0
0	0
0	0
0	0
0	0
0	0
0	0
0	0
0	0
0	0
0	0
0	0
0	0
0	0
0	0
0	0
0	0
0	0
0	0
0	0
0	0
0	0
0	0
0	0
0	0
0	0
0	0
0	0
0	0
0	0
0	0
0	0
0	0
0	0

0	0
0	0
0	0
0	0
0	0
0	0
0	6
0	0
0	0
0	0
0	0
0	0
0	0
0	0
0	0
0	0
0	7
0	0
0	0
0	0
0	0
0	0
0	0
0	0
0	0
0	0
0	0
0	0
0	0
0	0
0	0
0	0
0	0
0	0
0	0
0	0
0	0
0	0
0	0
0	0
0	0
0	0
0	0
0	0
0	0
0	0
0	0
0	0
0	0
0	0

0	0
0	0
0	0
0	0
0	0
0	0
0	0
0	0
0	0
0	0
0	0
0	0
0	42
0	0
0	0
0	0
0	0
0	0
0	0
0	0
0	0
0	0
0	0
0	0
0	0
0	0
0	0
0	0
0	0
0	0
0	0
0	0
0	0
0	0
0	0
0	0
0	0
0	0
0	0
0	0
0	0
0	0
0	0
0	6
0	0
0	0
0	0
0	0
0	0
0	0

0	0
0	0
0	0
0	0
0	0
0	0
0	0
0	0
0	0
0	0
0	0
0	0
0	0
0	0
0	0
0	0
0	0
0	0
0	0
0	0
0	0
0	0
0	0
0	0
0	0
0	0
0	0
0	0
0	0
0	0
0	0
0	0
0	0
0	0
0	0
0	0
0	0
0	0
0	0
0	0
0	0
0	0
0	0
0	0
0	0
0	0
0	0
0	0
0	0
0	0

0	0
0	0
0	0
0	0
0	0
0	0
0	0
0	0
0	0
0	0
0	0
0	0
0	0
0	0
0	0
0	0
0	0
0	0
0	0
0	0
0	0
0	0
0	0
0	0
0	0
0	0
0	0
0	0
0	0
0	0
0	0
0	0
0	0
0	0
0	0
0	0
0	0
0	0
0	0
0	0
0	0
0	0
0	0
0	0
0	0
0	33
0	0
0	0
0	0
0	0

0	0
0	0
0	0
0	0
0	0
0	0
0	0
0	0
0	0
0	0
0	0
0	0
0	0
0	0
0	0
0	0
0	0
0	0
0	0
0	0
0	0
0	0
0	0
0	0
0	0
0	0
0	0
0	0
0	0
0	0
0	0
0	0
0	0
0	0
0	0
0	31
0	0
0	0
0	0
0	0
0	0
0	0
0	0
0	0
0	0
0	0
0	0
0	0
0	0
0	0

0	0
0	0
0	0
0	0
0	0
0	0
0	0
0	0
0	0
0	0
0	0
0	0
0	0
0	0
0	0
0	0
0	0
0	0
0	0
0	0
0	0
0	0
0	0
0	0
0	3
0	0
0	0
0	0
0	0
0	0
0	0
0	0
0	0
0	0
0	0
0	0
0	0
0	0
0	0
0	0
0	0
0	0
0	0
0	0
0	0
0	0
0	0
0	0
0	0
0	0

0	0
0	0
0	0
0	0
0	0
0	0
0	0
0	0
0	0
0	0
0	0
0	0
0	0
0	0
0	0
0	0
0	0
0	0
0	0
0	0
0	0
0	0
0	0
0	0
0	0
0	0
0	27
0	0
0	0
0	0
0	0
0	0
0	0
0	0
0	0
0	0
0	0
0	0
0	0
0	0
0	0
0	0
0	0
0	0
0	0
0	0
0	0
0	0
0	0
0	0

0	0
0	0
0	0
0	0
0	0
0	0
0	0
0	0
0	0
0	0
0	0
0	0
0	0
0	0
0	0
0	0
0	0
0	0
0	0
0	0
0	0
0	0
0	0
0	0
0	0
0	0
0	0
0	0
0	0
0	0
0	0
0	0
0	0
0	0
0	0
0	0
0	0
0	0
0	0
0	0
0	0
0	0
0	0
0	0
0	0
0	0
0	0
0	0
0	0
0	0

0	0
0	0
0	0
0	0
0	0
0	0
0	0
0	0
0	0
0	0
0	0
0	0
0	0
0	0
0	0
0	0
0	0
0	0
0	0
0	0
0	0
0	0
0	0
0	0
0	0
0	0
0	0
0	0
0	0
0	0
0	0
0	0
0	0
0	0
0	0
0	0
0	0
0	0
0	0
0	0
0	0
0	0
0	0
0	0
0	0
0	0
0	0
0	0
0	0
0	0

0	0
0	0
0	0
0	0
0	0
0	0
0	0
0	0
0	0
0	0
0	0
0	0
0	0
0	0
0	0
0	0
0	0
0	0
0	0
0	0
0	0
0	0
0	0
0	0
0	0
0	0
0	0
0	0
0	0
0	0
0	0
0	0
0	0
0	0
0	0
0	0
0	0
0	0
0	0
0	0
0	0
0	0
0	0
0	0
0	0
0	0
0	0
0	0
0	0
0	0

0	0
0	0
0	0
0	0
0	0
0	0
0	0
0	0
0	0
0	0
0	0
0	0
0	0
0	0
0	0
0	0
0	0
0	0
0	0
0	0
0	0
0	0
0	0
0	0
0	0
0	0
0	0
0	0
0	0
0	0
0	0
0	0
0	0
0	0
0	0
0	0
0	0
0	0
0	0
0	0
0	0
0	0
0	0
0	0
0	0
0	0
0	0
0	0
0	0
0	0

0	0
0	0
0	0
0	0
0	0
0	0
0	0
0	0
0	0
0	0
0	0
0	0
0	0
0	0
0	0
0	0
0	0
0	0
0	0
0	0
0	0
0	0
0	0
0	0
0	0
0	0
0	0
0	0
0	0
0	0
0	0
0	0
0	0
0	0
0	0
0	0
0	0
0	0
0	0
0	0
0	0
0	0
0	0
0	0
0	0
0	0
0	0
0	0
0	0
0	0

0	0
0	0
0	0
0	0
0	0
0	0
0	0
0	0
0	0
0	0
0	0
0	0
0	0
0	0
0	0
0	0
0	0
0	0
0	0
0	0
0	0
0	0
0	0
0	0
0	0
0	0
0	0
0	0
0	0
0	0
0	0
0	0
0	0
0	0
0	0
0	0
0	0
0	0
0	0
0	0
0	0
0	0
0	0
0	0
0	0
0	0
0	0
0	0
0	0
0	0

0	0
0	0
0	0
0	0
0	0
0	0
0	0
0	0
0	0
0	0
0	0
0	0
0	0
0	0
0	0
0	0
0	0
0	0
0	0
0	0
0	0
0	0
0	0
0	0
0	0
0	0
0	0
0	0
0	0
0	0
0	0
0	0
0	0
0	0
0	0
0	0
0	0
0	15
0	0
0	0
0	0
0	0
0	0
0	0
0	0
0	0
0	0
0	0
0	0
0	0

0	0
0	0
0	0
0	0
0	0
0	0
0	0
0	0
0	0
0	0
0	0
0	0
0	0
0	0
0	0
0	0
0	0
0	0
0	0
0	0
0	0
0	0
0	0
0	0
0	0
0	0
0	0
0	0
0	0
0	0
0	0
0	0
0	0
0	0
0	0
0	0
0	0
0	0
0	0
0	0
0	3
0	0
0	0
0	0
0	0
0	0
0	0
0	0
0	0
0	0

0	0
0	0
0	0
0	0
0	0
0	0
0	0
0	0
0	0
0	0
0	0
0	0
0	0
0	0
0	0
0	0
0	0
0	0
0	0
0	0
0	0
0	0
0	0
0	0
0	0
0	0
0	0
0	0
0	0
0	0
0	0
0	0
0	0
0	0
0	0
0	0
0	0
0	0
0	0
0	0
0	0
0	0
0	0
0	0
0	0
0	0
0	0
0	0
0	0
0	0

0	0
0	0
0	0
0	0
0	0
0	0
0	0
0	0
0	0
0	0
0	0
0	0
0	0
0	0
0	0
0	0
0	0
0	0
0	0
0	0
0	0
0	0
0	0
0	0
0	0
0	0
0	0
0	0
0	0
0	0
0	0
0	0
0	0
0	0
0	0
0	0
0	0
0	0
0	0
0	0
0	0
0	0
0	0
0	0
0	0
0	0
0	0
0	0
0	0
0	0

0	0
0	0
0	0
0	0
0	0
0	0
0	0
0	0
0	0
0	0
0	0
0	0
0	0
0	0
0	0
0	0
0	0
0	0
0	0
0	0
0	0
0	0
0	0
0	0
0	0
0	0
0	0
0	0
0	0
0	0
0	0
0	0
0	0
0	0
0	0
0	0
0	0
0	0
0	0
0	0
0	0
0	0
0	0
0	0
0	0
0	0
0	0
0	0
0	0
3	0

0	0
0	0
0	0
0	0
0	0
0	0
0	0
0	0
0	0
0	0
0	0
0	0
0	0
0	0
0	0
0	2
8	0
0	0
0	0
0	0
0	0
0	0
0	0
0	0
0	0
0	0
0	0
0	0
0	0
0	0
0	0
0	0
0	0
0	0
0	0
0	0
0	0
0	0
0	0
0	0
0	0
0	0
0	0
0	0
0	0
0	0
0	0
0	0
0	0
0	0

0	0
0	0
0	0
0	0
0	0
0	0
0	0
0	0
0	0
0	0
0	0
0	0
0	0
0	0
0	0
0	0
0	0
0	0
0	0
0	0
0	0
0	0
0	0
0	0
0	0
0	0
0	0
0	0
0	0
0	0
0	0
0	0
0	0
0	0
0	0
0	0
0	0
0	0
0	0
0	0
0	0
0	0
0	0
0	0
0	0
0	0
0	0
0	0
0	0
0	0

0	0
0	0
0	0
0	0
0	0
0	0
0	0
0	0
0	0
0	2
0	0
0	0
0	0
0	0
0	0
0	0
0	0
0	5
0	0
0	0
0	0
0	0
0	0
0	0
0	0
0	0
0	0
0	0
0	0
0	0
0	0
0	0
0	0
0	0
0	0
0	0
0	0
0	0
0	0
0	0
0	0
0	0
0	0
0	0
0	0
0	0
4	0
0	0
0	0
0	0

0	0
0	0
0	0
0	0
0	0
0	0
0	0
0	0
0	0
0	0
0	0
0	0
0	0
0	0
0	0
0	0
0	0
0	0
0	0
0	0
0	0
0	0
0	0
0	0
0	0
0	0
0	0
0	0
0	0
0	0
0	0
0	0
0	0
0	0
0	0
0	0
0	0
0	0
0	0
0	0
0	0
0	0
0	0
0	0
0	0
0	0
0	0
0	0
0	0
0	0

0	0
0	0
0	0
0	0
0	0
0	0
0	0
0	0
0	0
0	0
0	0
0	0
0	0
0	0
0	0
0	0
0	0
0	0
0	0
0	0
0	0
0	0
0	3
0	3
0	3
0	3
0	3
0	3
0	0
0	0
0	0
0	0
0	0
0	0
0	0
0	0
0	0
0	0
0	0
0	0
0	0
0	0
0	0
0	0
0	0
0	0
0	0
0	0
0	0
0	0

0	0
0	0
0	0
0	0
0	0
0	0
0	0
0	0
0	0
0	0
0	0
0	0
0	0
0	0
0	0
0	0
0	0
0	0
0	0
0	0
0	0
0	0
0	0
0	0
0	0
0	0
0	0
0	0
0	0
0	0
0	0
0	0
0	0
0	0
0	0
0	0
0	0
0	0
0	0
0	0
0	0
0	0
0	0
0	0
0	0
0	0
0	0
0	0
0	0
0	0

0	0
0	0
0	0
0	0
0	0
0	0
0	0
0	0
0	0
0	0
0	0
0	0
0	0
0	0
0	0
0	0
0	0
0	0
0	0
0	2
0	2
0	2
0	0
